# Supplementary material for: A recyclable polyester library from reversible alternating copolymerization of aldehyde and cyclic anhydride
Source: Nat Commun. 2023 Sep 5;14:5423. doi: 10.1038/s41467-023-41136-6 (PMC10480228; doi:10.1038/s41467-023-41136-6)
Supplement: Supplementary file 1 — Supplementary Information [file 41467_2023_41136_MOESM1_ESM.pdf]

Supplementary information

A recyclable polyester library from reversible alternating copolymerization of aldehyde and cyclic anhydride

Xun Zhang,[1] Wenqi Guo,[1] Chengjian Zhang,[1] and Xinghong Zhang[1,2]

[1]National Key Laboratory of Biobased Transportation Fuel Technology, International Research Center for X Polymers, Department of Polymer Science and Engineering, Zhejiang University, Hangzhou 310027 (China)

[2]Center of Chemistry for Frontier Technologies, Zhejiang University, Hangzhou 310027 (China)

Corresponding authors: chengjian.zhang@zju.edu.cn (Chengjian Zhang); xhzhang@zju.edu.cn (Xinghong Zhang).

Table of Contents

Supplementary Table 1. Copolymerization of cyclic anhydride with acetaldehyde . 3

Supplementary Fig. 1 (a) ^{1}H, (b) ^{13}C, and (c) ^{1}H-^{13}C HSQC NMR spectra of **P1A** in $CDCl_3$. 4

Supplementary Fig. 2 The reaction of **P1A** with diisocyanate.......... 6

Supplementary Table 2. Copolymerization of various cyclic anhydrides with aldehydes 7

Supplementary Figs. 3-61 NMR spectra of the obtained copolymers in $CDCl_3$....... 8

Supplementary Table 3. Determination of equilibrium monomer concentration for the copolymerization of **A** and **1**..........68

Supplementary Table 4. Determination of equilibrium monomer concentration for the copolymerization of **A** and **21**..........69

Supplementary Fig. 62 Thermodynamics of **21** and **A** copolymerization..........70

Supplementary Figs. 63-122 (a) TGA and (b) DSC curves of the obtained copolymers. 71

Supplementary Table 5. Copolymerization of **36** with **A** with $InBr_3$..........131

Supplementary Fig. 123 GPC curves of the original and recovered **P36A**..........132

Supplementary Figs. 124-129 Representative GPC curves of the obtained copolymers 133

Supplementary Table 1. Copolymerization of cyclic anhydride with acetaldehyde using various catalysts [a]

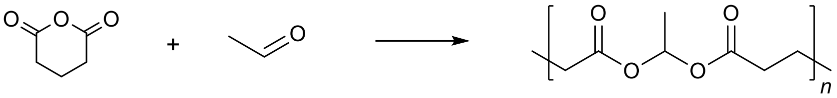

entry	aldehyde	cyclic anhydride	Cat.	*T* (°C)	t (s)	Conv.[b]	M_n (kDa)[c]	$Đ^c$
1	**1**	**A**	BF_3•Et_2O	25	180	89	19.7	1.3
2	**1**	**A**	$B(C_6F_5)_3$	25	180	90	22.0	1.2
3	**1**	**A**	Bu_2BOTf	25	180	88	11.0	1.2
4	**1**	**A**	$SnCl_4$	25	180	87	13.1	1.2
5	**1**	**A**	$InBr_3$	25	180	90	16.3	1.3
6	**1**	**A**	$NH(OTf)_2$	25	180	87	15.7	1.4
7	**1**	**A**	MeOTf	25	180	83	13.1	1.3
8	**1**	**A**	$^tBuP_4$	25	3600	0	-	-
9	**1**	**A**	(Salen)CrCl /PPNCl	25	3600	0	-	-
10	**1**	**A**	TEB /PPNCl	25	3600	0	-	-
11	**1**	**A**	(Salen)CoCl /PPNCl	25	3600	0	-	-
12	**1**	-	BF_3•Et_2O	25	3600	0	-	-
13	-	**A**	BF_3•Et_2O	25	3600	0	-	-

[a] The copolymerization was performed in 0.2 ml CH_2Cl_2, $[\mathbf{A}]_0$ = 0.0023 M, $[\mathbf{A}]_0$:$[\mathbf{1}]_0$:[catalyst] = 100:100:1; [b] Conversion of **1** or **A**, determined by ^1H NMR spectroscopy; [c] Determined by GPC in THF, calibrated with polystyrene standards.

a

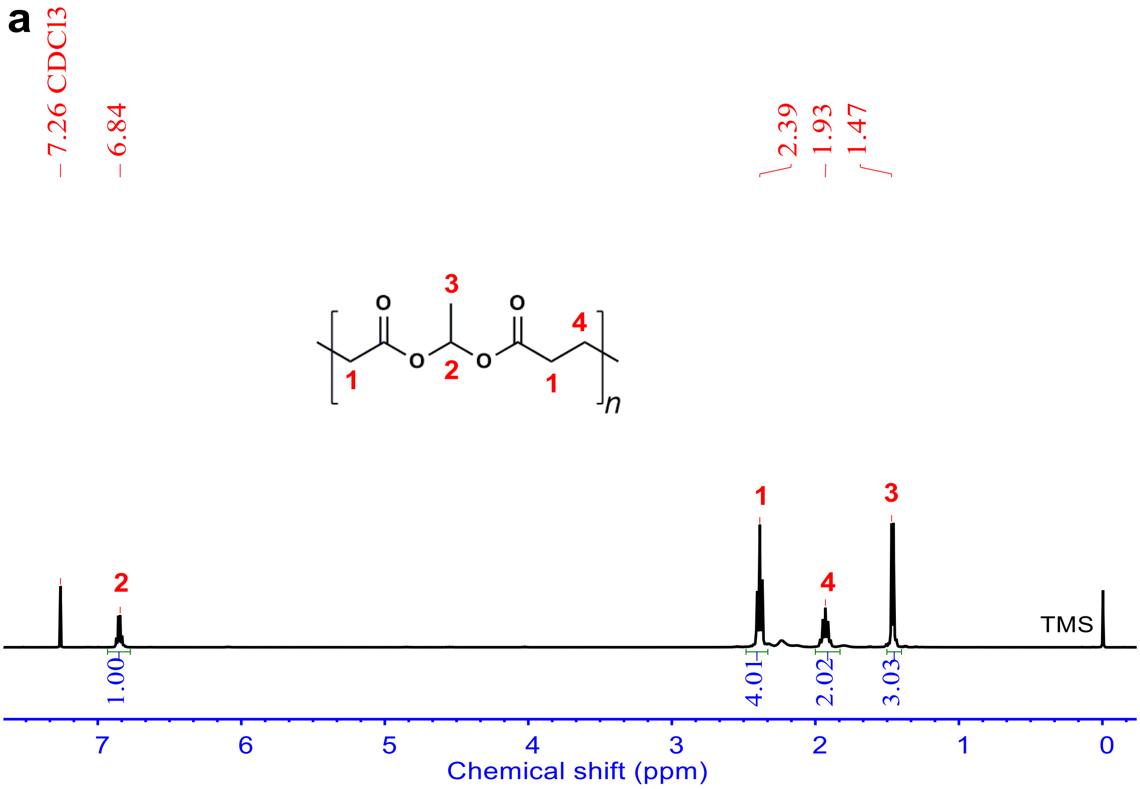

7.26 CDCl3
6.84
2.39
1.93
1.47
1
2
3
4
n
TMS
1.00
4.01
2.02
3.03
7
6
5
4
3
2
1
0
Chemical shift (ppm)

b

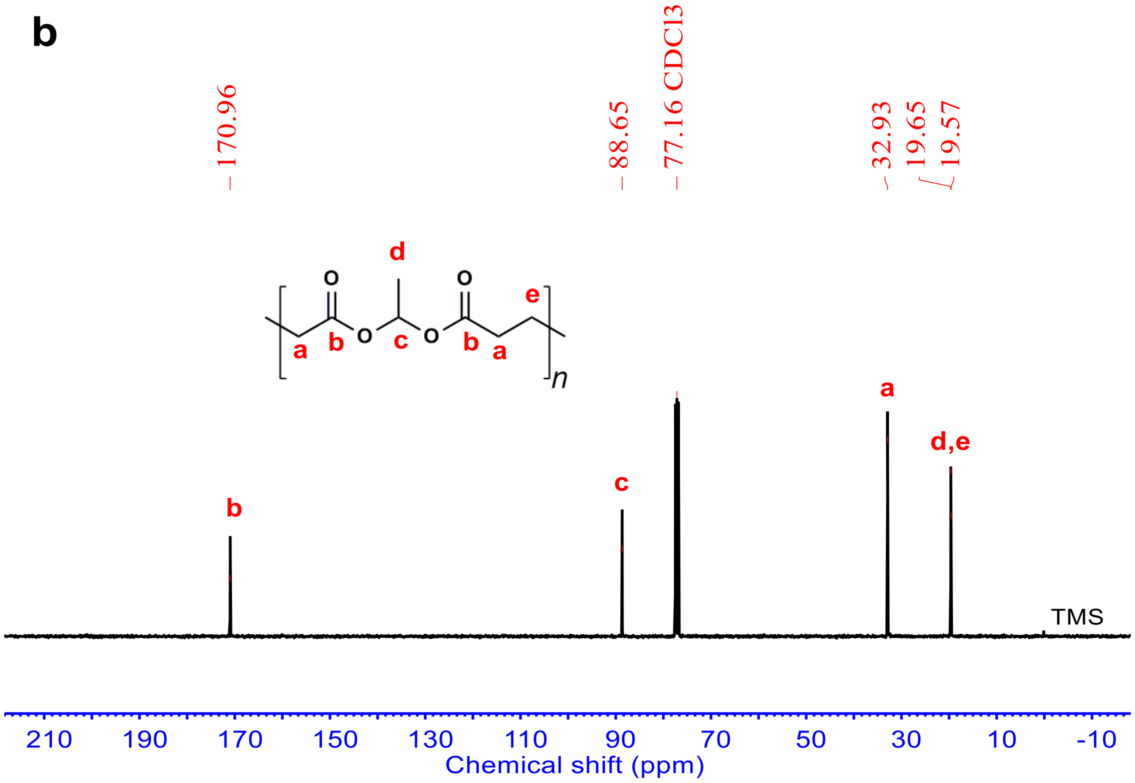

170.96
88.65
77.16 CDCl3
32.93
19.65
19.57
a
b
c
d
e
d,e
n
TMS
210
190
170
150
130
110
90
70
50
30
10
-10
Chemical shift (ppm)

c

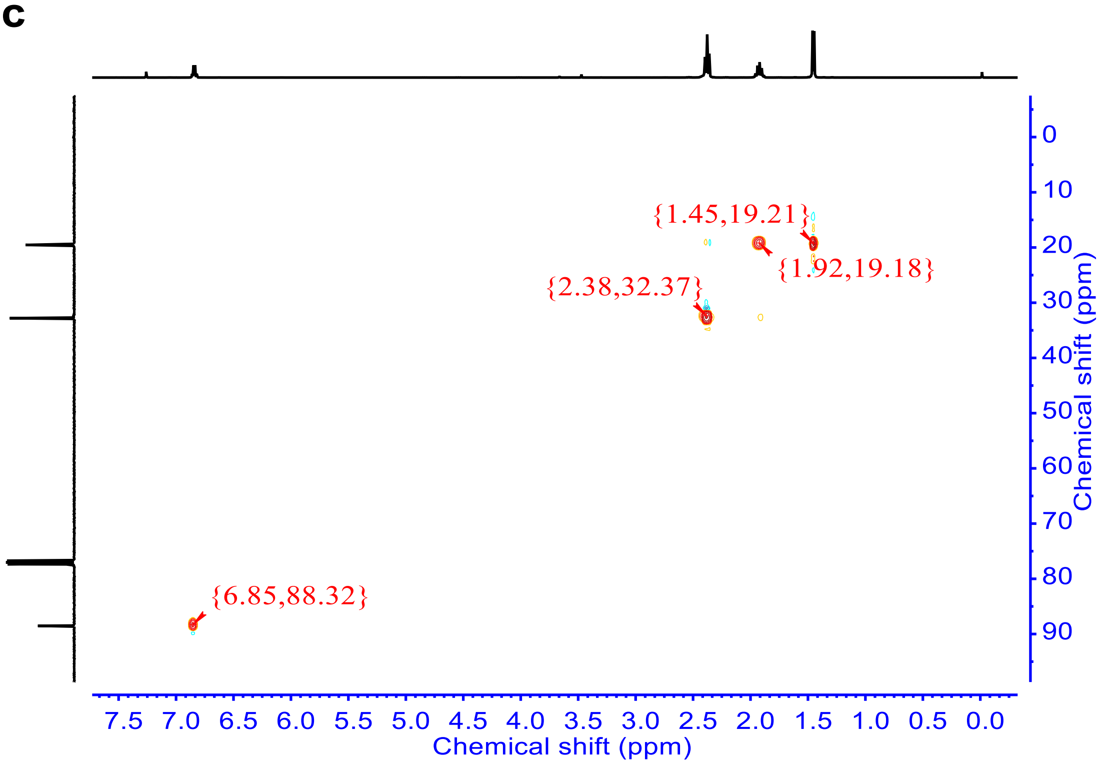

Supplementary Fig. 1 (a) ^{1}H, (b) ^{13}C, and (c) ^{1}H-^{13}C HSQC NMR spectra of **P1A** in $CDCl_3$.

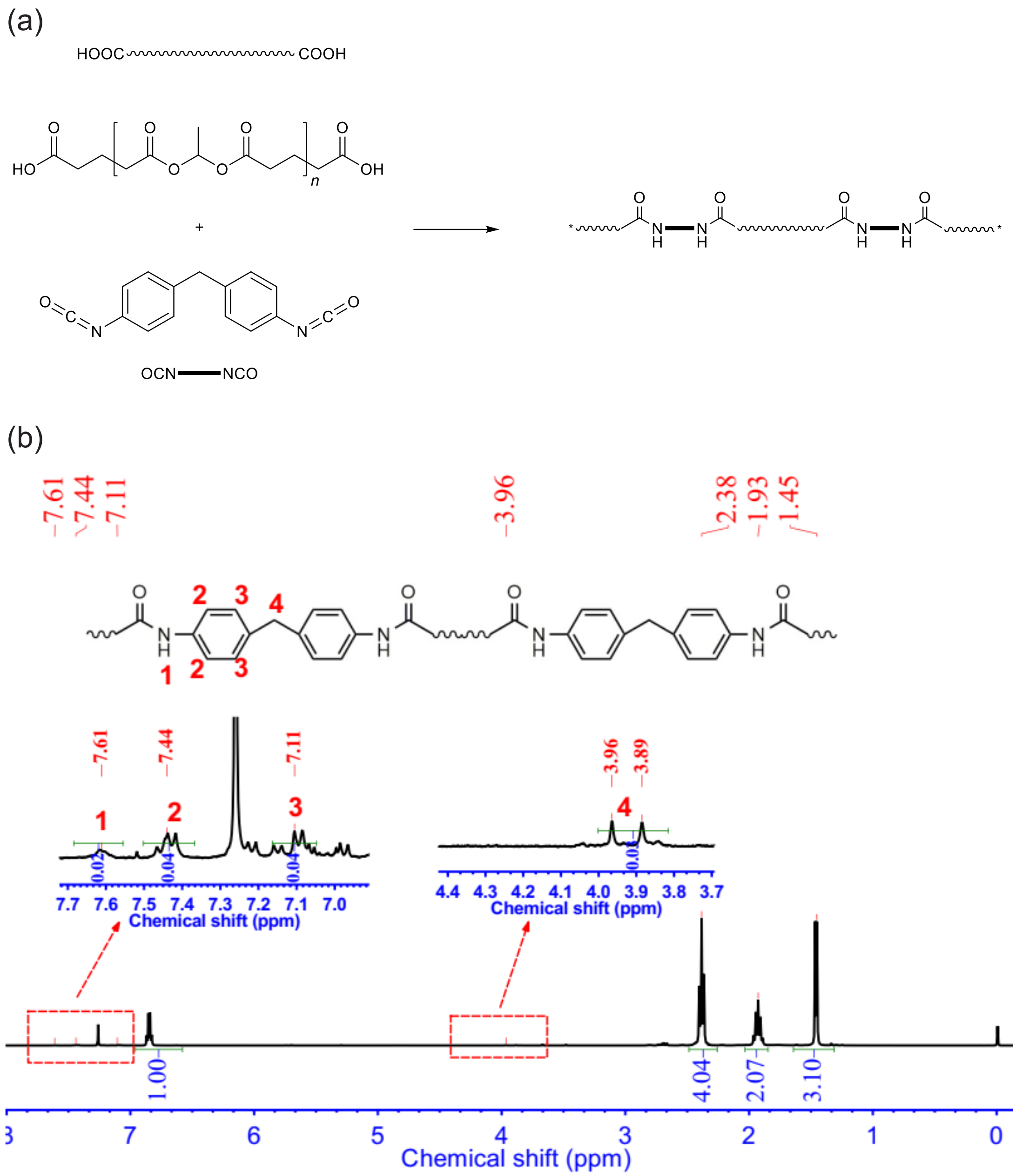

Supplementary Fig. 2 (a) Illustration of the reaction of **P1A** with diisocyanate and (b) the ^{1}H NMR spectrum of the resulting polyamide. Reaction conditions: room temperature, for 2 hours, 0.5 g of **P1A**, 2 ml of CH_2Cl_2, 10 mg of methylene diphenyl diisocyanate, 0.015 mmol P2-*t*-Bu.

Supplementary Table 2. Copolymerization of various cyclic anhydrides with aldehydes [a]

aldehyde	cyclic anhydride	polymer	Cat.	T (°C)	t (min)	Conv.[b]	M_n (kDa)[c]	$Đ$[c]	T_g (T_m)[d]	T_d[e]
**2**	**A**	**P2A**	BF_3•Et_2O	25	30	94	9.3	1.4	-40	185
**3**	**A**	**P3A**	$InBr_3$	25	30	94	5.6	1.3	-45	174
**4**	**A**	**P4A**	BF_3•Et_2O	25	30	95	7.6	1.3	-52	190
**5**	**A**	**P5A**	$InBr_3$	25	30	94	7.0	1.4	-52	206
**6**	**A**	**P6A**	$InBr_3$	25	30	81	8.6	1.4	-51	201
**7**	**A**	**P7A**	$InBr_3$	25	30	88	8.7	1.4	(-11)	201
**8**	**A**	**P8A**	$InBr_3$	25	30	85	6.1	1.4	(11)	211
**9**	**A**	**P9A**	BF_3•Et_2O	25	30	88	13.9	1.5	(20)	210
**10**	**A**	**P10A**	$InBr_3$	25	30	89	3.4	1.3	(37)	212
**11**	**A**	**P11A**	$InBr_3$	25	30	80	16.6	1.4	(42)	213
**12**	**A**	**P12A**	$InBr_3$	25	30	89	3.0	1.4	(53)	191
**13**	**A**	**P13A**	BF_3•Et_2O	25	30	85	7.5	1.2	1	218
**14**	**A**	**P14A**	BF_3•Et_2O	25	30	87	10.6	1.3	-33	225
**15**	**A**	**P15A**	$InBr_3$	25	30	90	15.9	1.4	-26	190
**16**	**A**	**P16A**	$InBr_3$	25	30	85	3.3	1.3	-57	200
**17**	**A**	**P17A**	$InBr_3$	25	30	88	5.5	1.5	-37	211
**18**	**A**	**P18A**	$InBr_3$	25	30	90	5.5	1.2	10	187
**19**	**A**	**P19A**	BF_3•Et_2O	25	30	96	3.1	1.4	56	183
**20**	**A**	**P20A**	BF_3•Et_2O	25	30	99	3.1	1.5	73	199
**21**	**A**	**P21A**	$InBr_3$	0	30	80	10.8	1.6	25	194
**22**	**A**	**P22A**	$InBr_3$	25	30	67	8.1	1.5	15	187
**23**	**A**	**P23A**	$InBr_3$	25	30	76	9.6	1.5	3	180
**24**	**A**	**P24A**	$InBr_3$	0	30	73	7.7	1.5	7	180
**25**	**A**	**P25A**	$InBr_3$	0	30	64	5.4	1.4	0	178
**26**	**A**	**P26A**	$InBr_3$	25	30	70	4.3	1.4	-2	171
**27**	**A**	**P27A**	$InBr_3$	25	30	70	5.3	1.4	-6	184
**28**	**A**	**P28A**	$InBr_3$	25	30	76	4.5	1.3	40	195
**29**	**A**	**P29A**	$InBr_3$	0	30	76	5.2	1.4	20	197
**30**	**A**	**P30A**	BF_3•Et_2O	25	30	76	11.3	1.4	32	186
**31**	**A**	**P31A**	BF_3•Et_2O	25	30	73	8.9	1.4	2	175
**32**	**A**	**P32A**	BF_3•Et_2O	25	30	74	13.7	1.4	28	193
**33**	**A**	**P33A**	$InBr_3$	25	30	65	7.7	1.3	25	174
**34**	**A**	**P34A**	$InBr_3$	25	30	63	5.1	1.4	16	195
**35**	**A**	**P35A**	$InBr_3$	0	30	75	3.8	1.3	31	186
**36**	**A**	**P36A**	$InBr_3$	25	30	71	5.8	1.5	50	190
**37**	**A**	**P37A**	$InBr_3$	25	30	67	5.1	1.4	40	182
**38**	**A**	**P38A**	$InBr_3$	25	30	66	6.8	1.6	58	184
**39**	**A**	**P39A**	$InBr_3$	0	30	65	3.2	1.3	33	184
**40**	**A**	**P40A**	$InBr_3$	0	30	79	3.5	1.4	35	221
**41**	**A**	**P41A**	$InBr_3$	25	30	72	7.1	1.6	44	188

**42**	**A**	**P42A**	$InBr_3$	25	30	66	12.0	1.6	43	191
**43**	**A**	**P43A**	$InBr_3$	25	30	71	9.4	1.6	10	180
**44**	**A**	**P44A**	$InBr_3$	0	30	69	8.9	1.5	32	173
**45**	**A**	**P45A**	BF_3•Et_2O	25	30	75	7.9	1.5	53	184
**46**	**A**	**P46A**	BF_3•Et_2O	25	30	69	9.3	1.6	48	185
**47**	**A**	**P47A**	BF_3•Et_2O	25	30	64	5.6	1.4	52	177
**48**	**A**	**P48A**	$InBr_3$	25	30	72	7.4	1.4	49	171
**49**	**A**	**P49A**	$InBr_3$	0	30	75	6.5	1.3	28	192
**50**	**A**	**P50A**	$InBr_3$	0	30	78	6.0	1.5	42	199
**51**	**A**	**P51A**	BF_3•Et_2O	25	30	69	5.7	1.6	44	172
**52**	**A**	**P52A**	$InBr_3$	25	30	69	8.0	1.5	60	175
**53**	**A**	**P53A**	$InBr_3$	25	30	61	4.7	1.5	22	170
**54**	**A**	**P54A**	$InBr_3$	0	30	73	4.5	1.3	26	179
**55**	**A**	**P55A**	$InBr_3$	25	30	67	4.2	1.3	35	214
**56**	**A**	**P56A**	$InBr_3$	25	30	78	2.2	1.3	43	193
**1**	**B**	**P1B**	BF_3•Et_2O	25	30	77	18.3	1.5	-6	185
**1**	**C**	**P1C**	$InBr_3$	25	30	63	2.8	1.5	0	180
**1**	**D**	**P1D**	$InBr_3$	25	30	70	2.5	1.4	-18	175
**1**	**E**	**P1E**	$InBr_3$	25	30	74	4.2	1.5	-25	230
**1**	**Maleic anhydride**	**-**	BF_3•Et_2O	25	30	0	-	-	-	-
**1**	**Succinic anhydride**	**-**	BF_3•Et_2O	25	30	0	-	-	-	-
**21**	**Maleic anhydride**	**-**	BF_3•Et_2O	25	30	0	-	-	-	-
**21**	**Succinic anhydride**	**-**	BF_3•Et_2O	25	30	0	-	-	-	-

[a] The copolymerization was performed in 0.2 ml CH_2Cl_2, $[\text{cyclic anhydride}]_0$ = 0.0023 M, $[\text{cyclic anhydride}]_0$:$[\text{aldehyde}]_0$:[catalyst] = 100:100:1; [b] Conversion of cyclic anhydride, determined by ^1H NMR spectroscopy; [c] Determined by GPC in THF, calibrated with polystyrene standards; [d] Determined by DSC; [e] Determined by TGA.

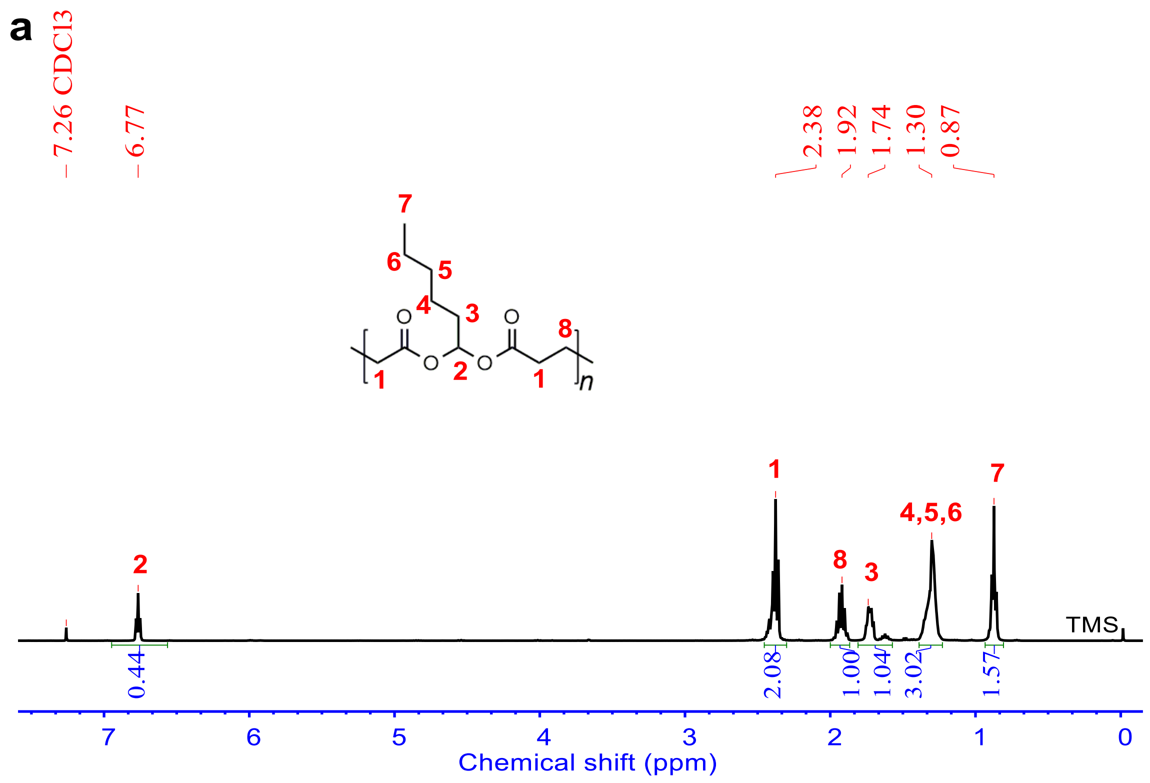

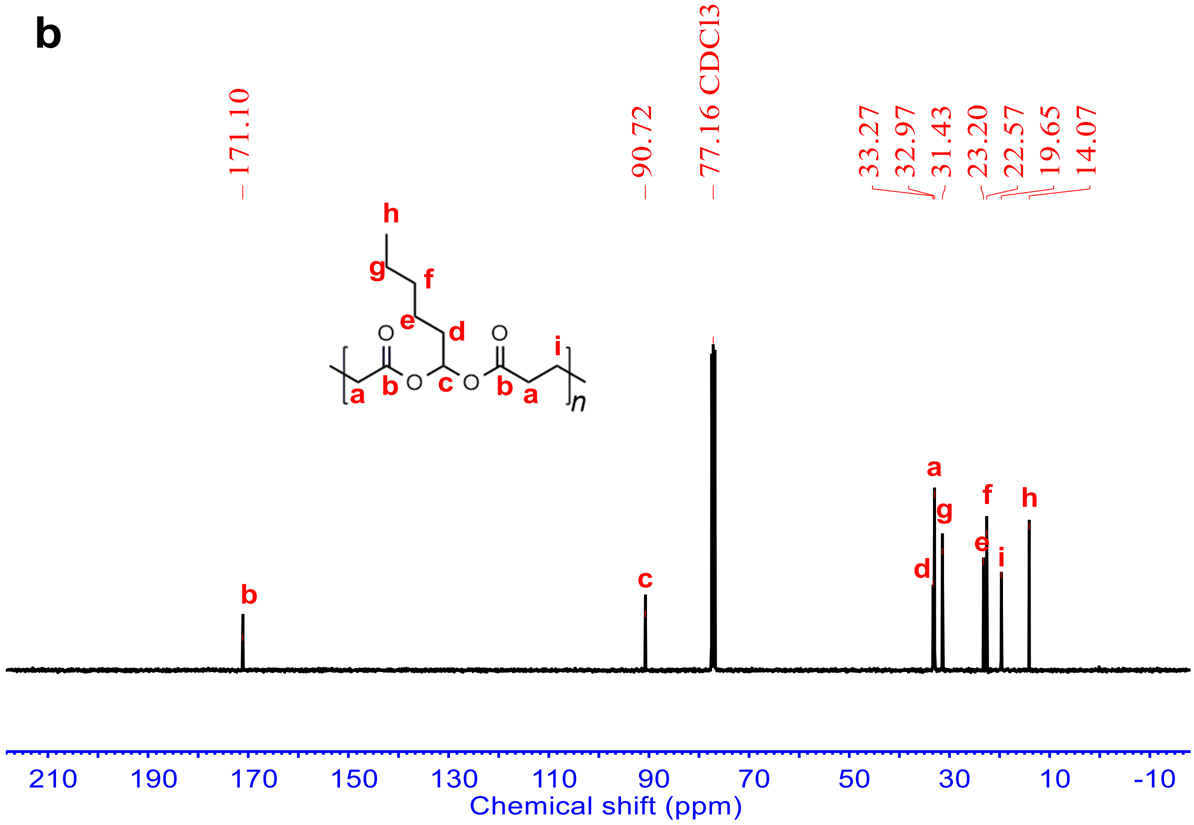

Supplementary Fig. 3 (a) ^{1}H and (b) ^{13}C NMR spectra of the obtained copolymer of **P2A** in $CDCl_3$.

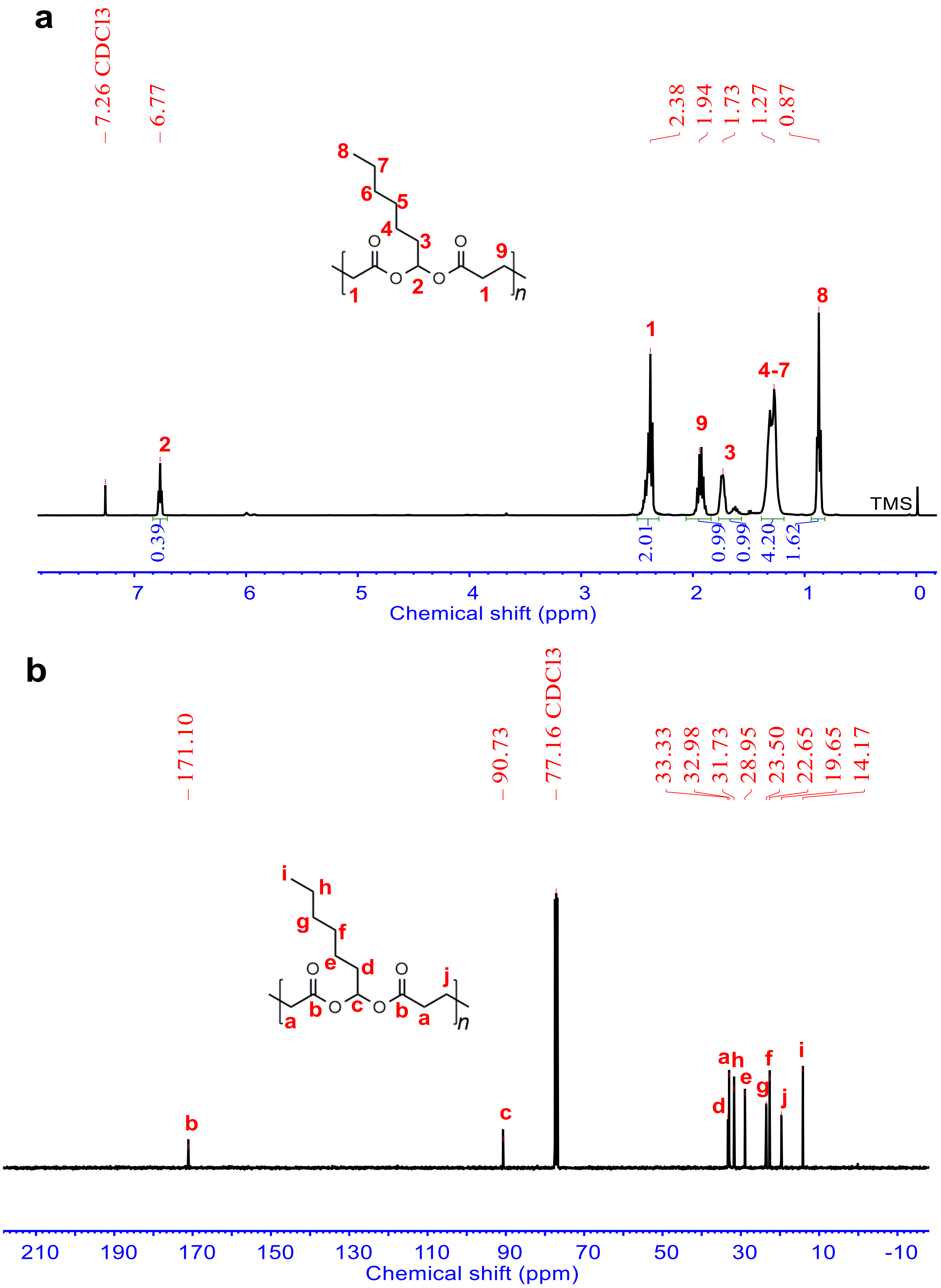

Supplementary Fig. 4 (a) ^{1}H and (b) ^{13}C NMR spectra of the obtained copolymer of **P3A** in $CDCl_3$.

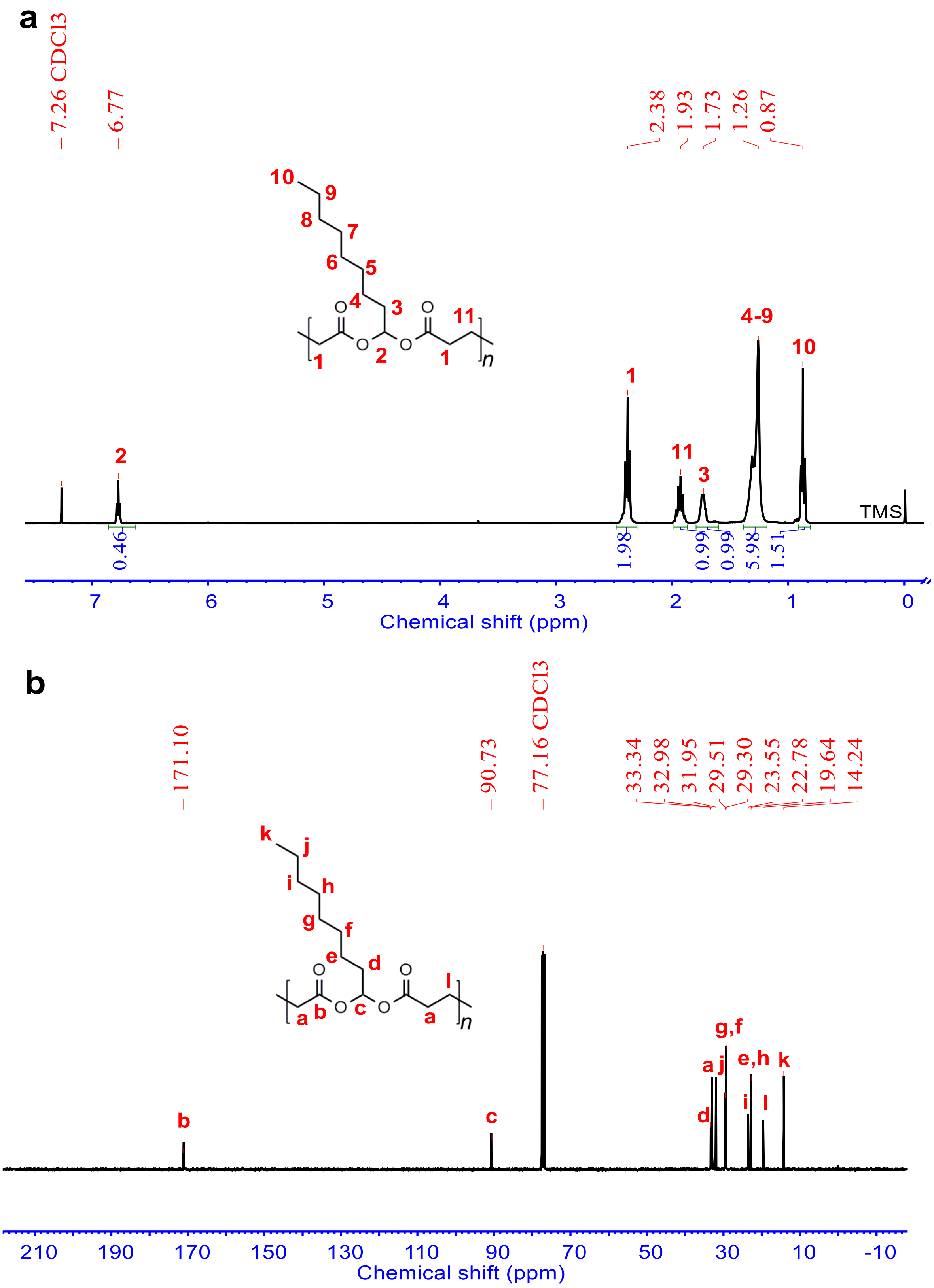

Supplementary Fig. 5 (a) ^{1}H and (b) ^{13}C NMR spectra of the obtained copolymer of **P4A** in $CDCl_3$.

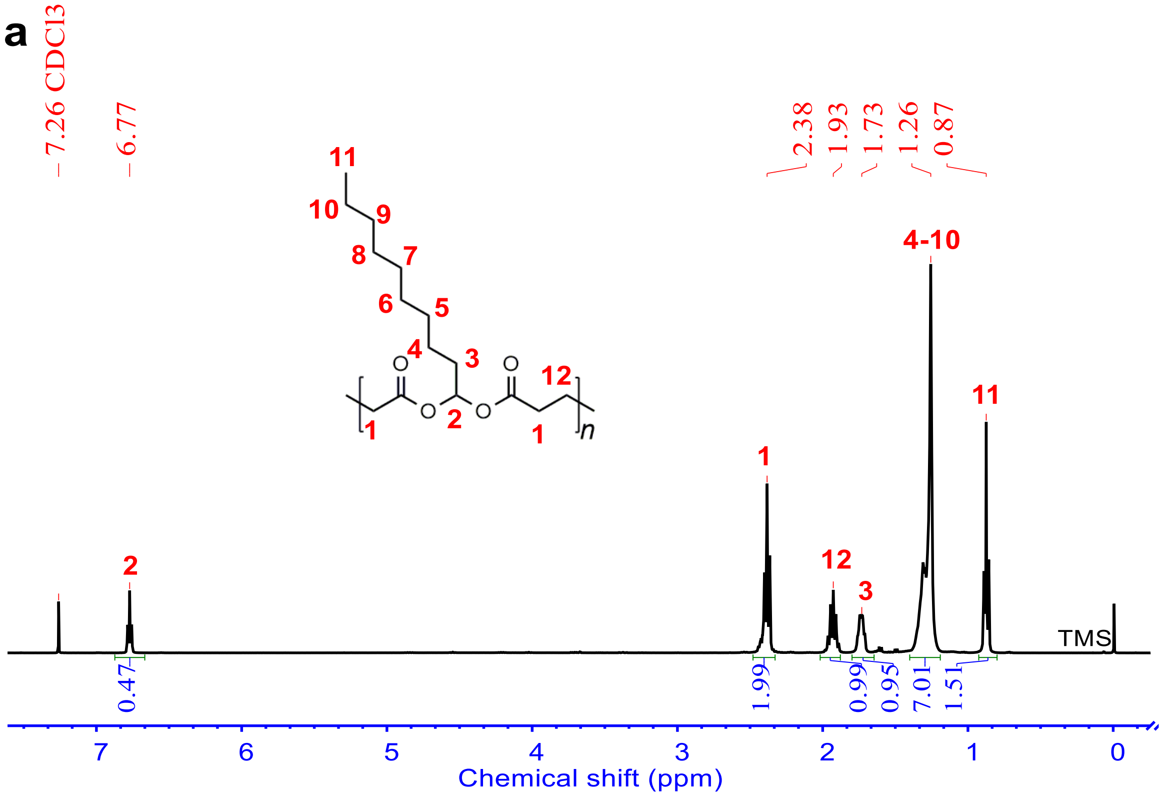

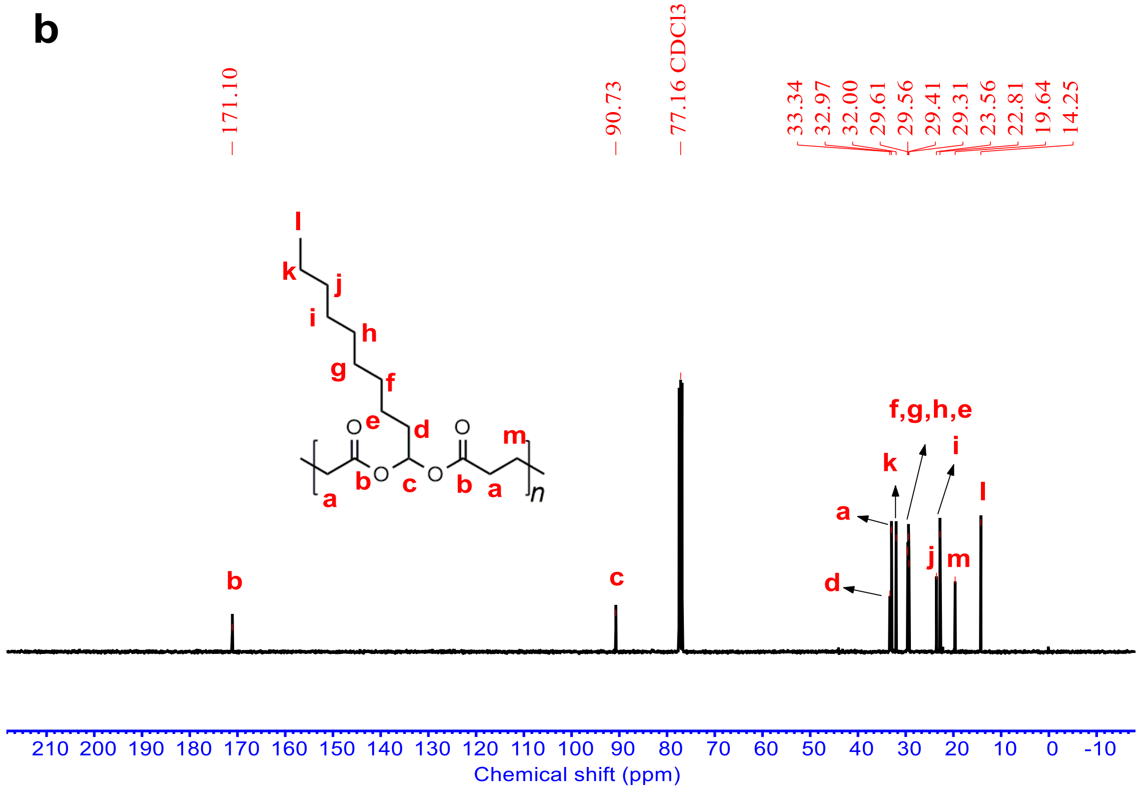

Supplementary Fig. 6 (a) ^{1}H and (b) ^{13}C NMR spectra of the obtained copolymer of **P5A** in $CDCl_3$.

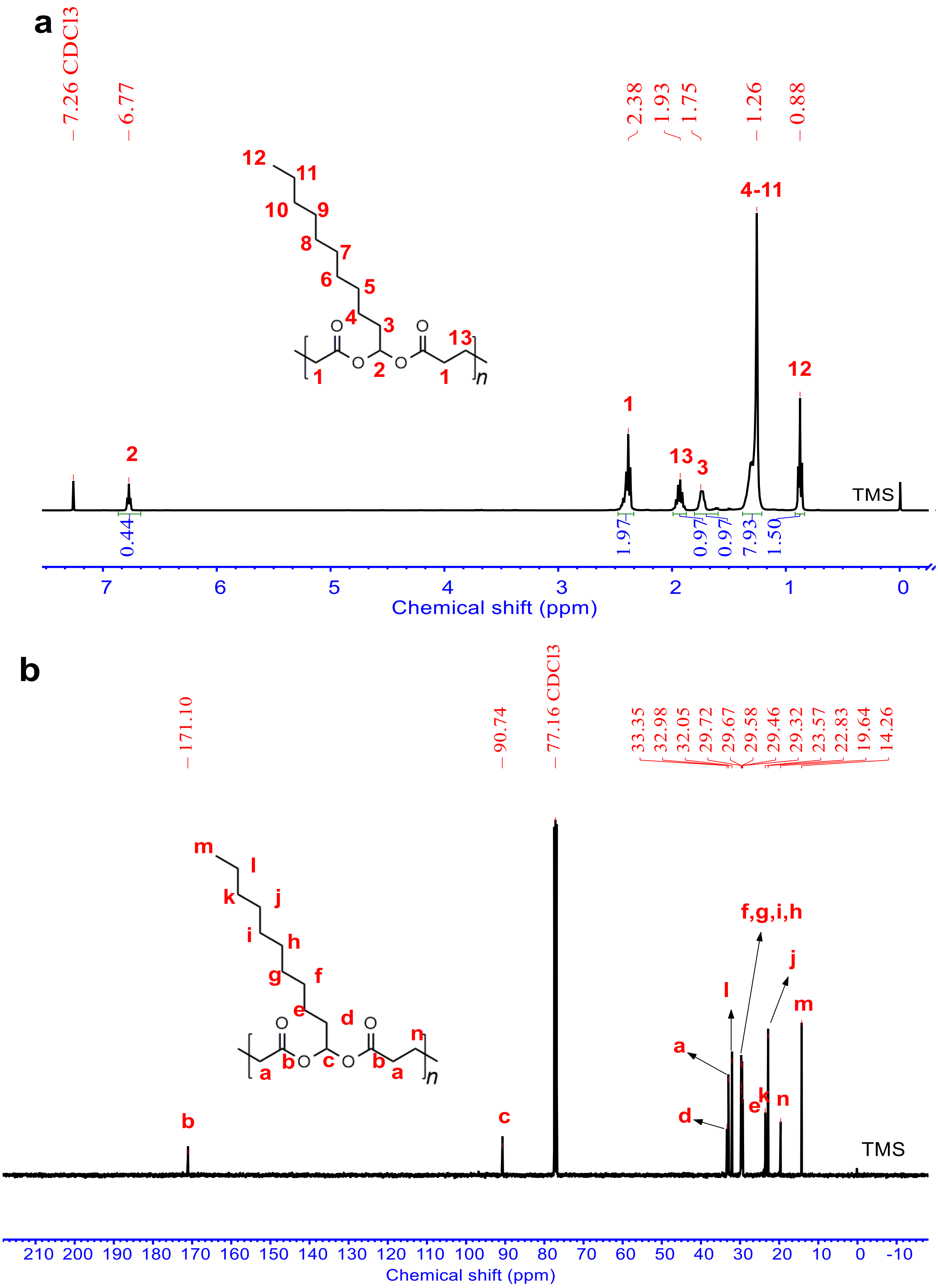

Supplementary Fig. 7 (a) ^{1}H and (b) ^{13}C NMR spectra of the obtained copolymer of **P6A** in $CDCl_3$.

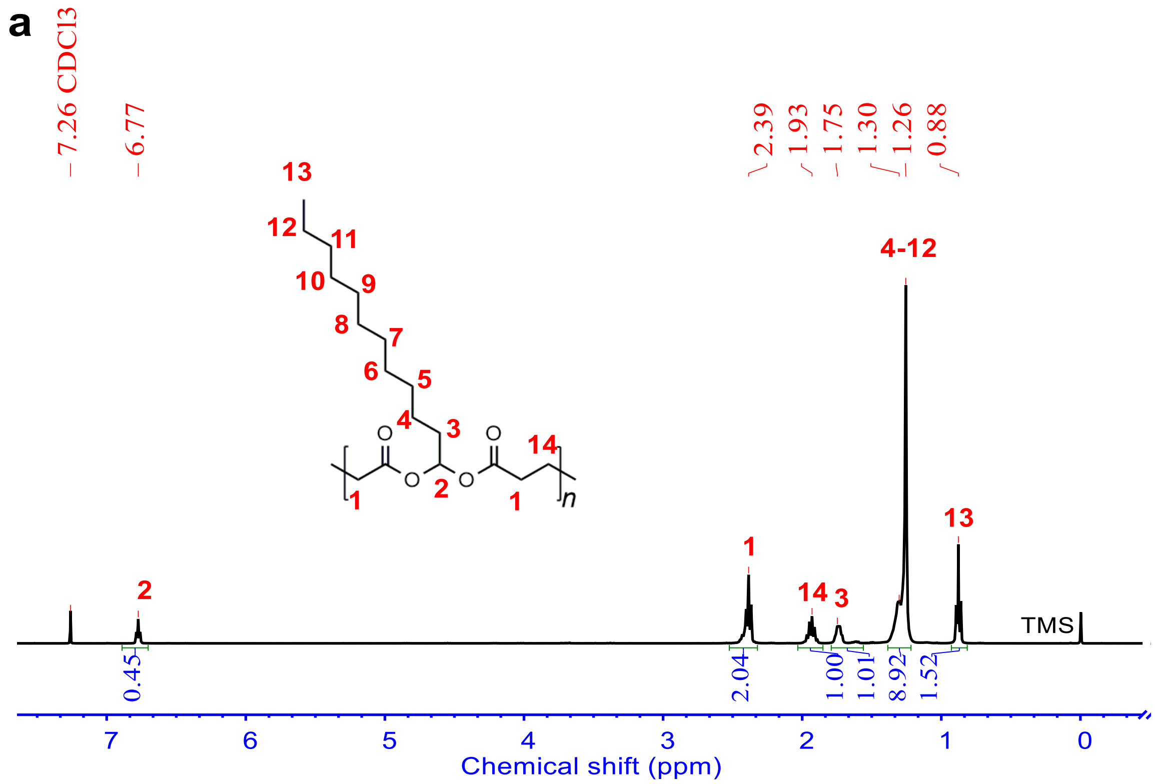

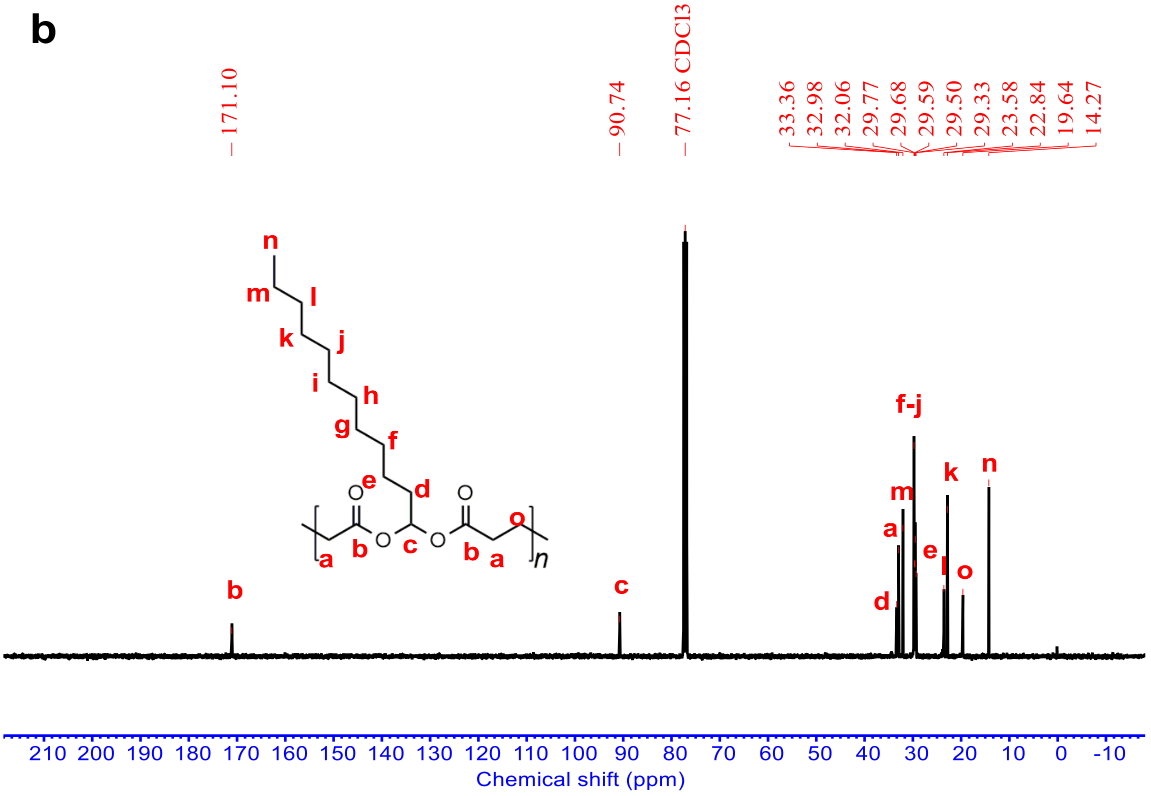

Supplementary Fig. 8 (a) ^{1}H and (b) ^{13}C NMR spectra of the obtained copolymer of **P7A** in $CDCl_3$.

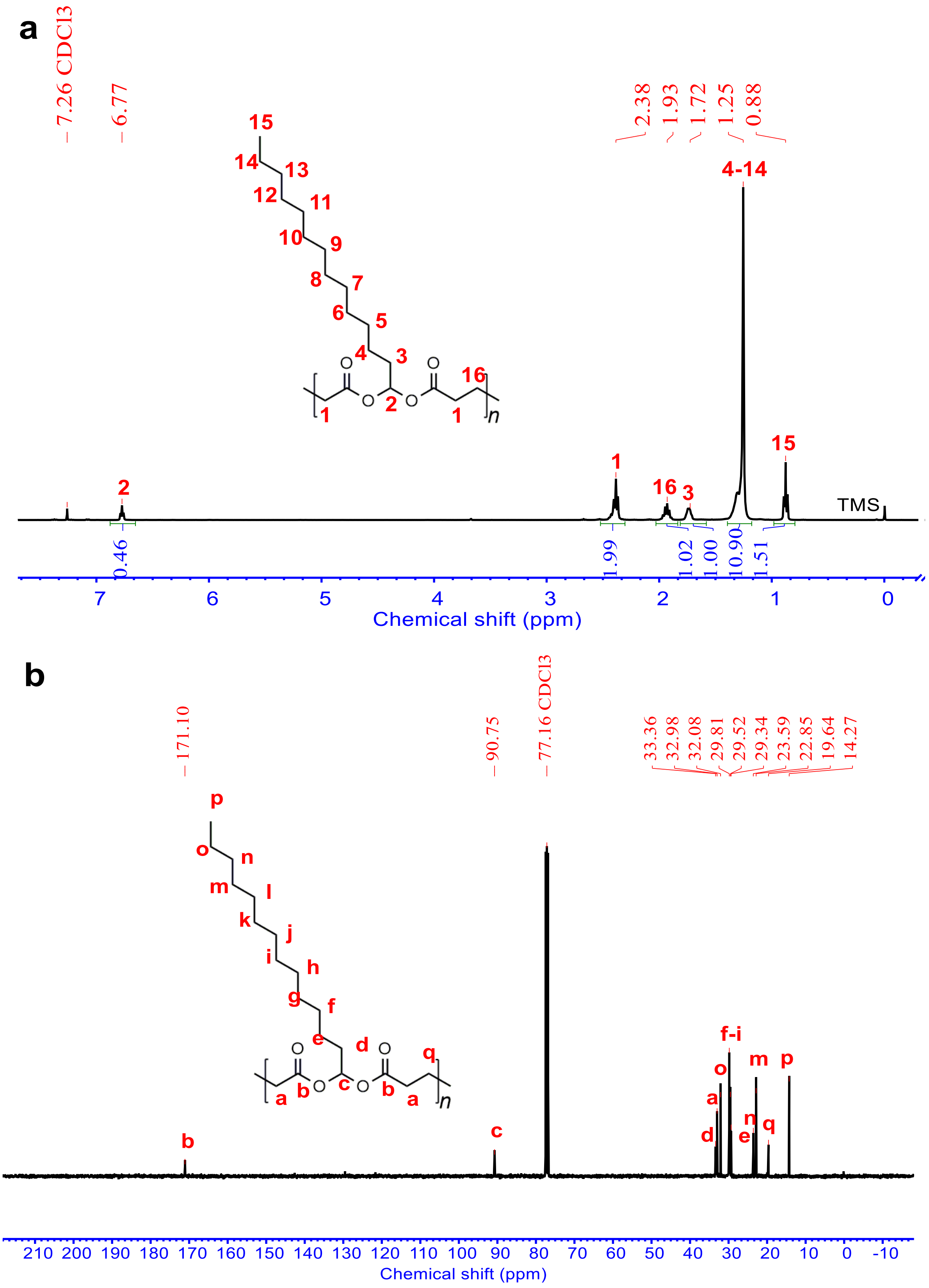

Supplementary Fig. 9 (a) ^{1}H and (b) ^{13}C NMR spectra of the obtained copolymer of **P8A** in $CDCl_3$.

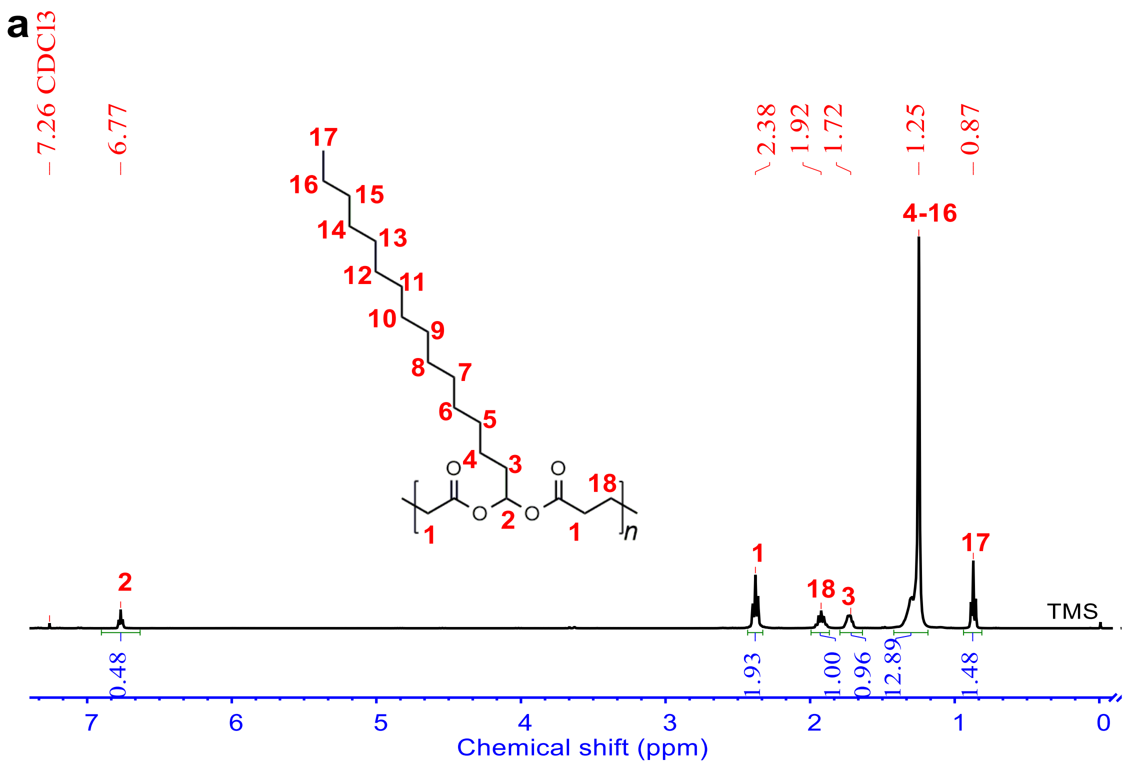

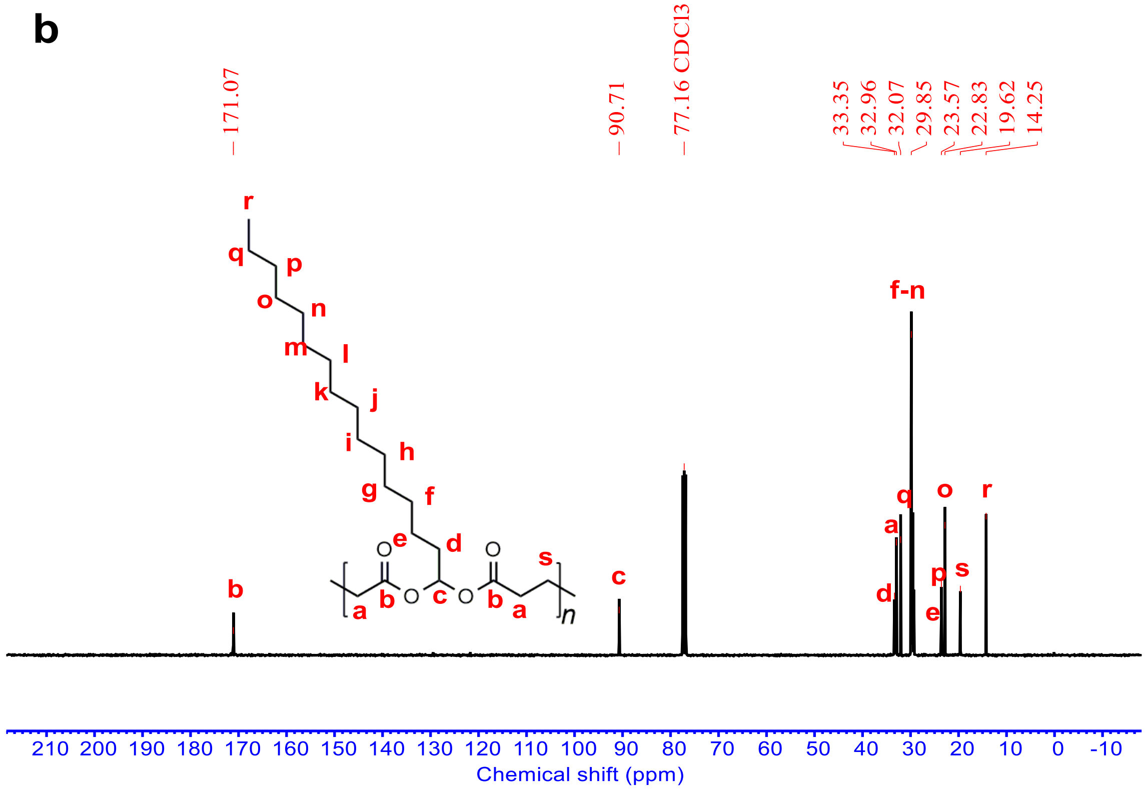

Supplementary Fig. 10 (a) ^{1}H and (b) ^{13}C NMR spectra of the obtained copolymer of **P9A** in $CDCl_3$.

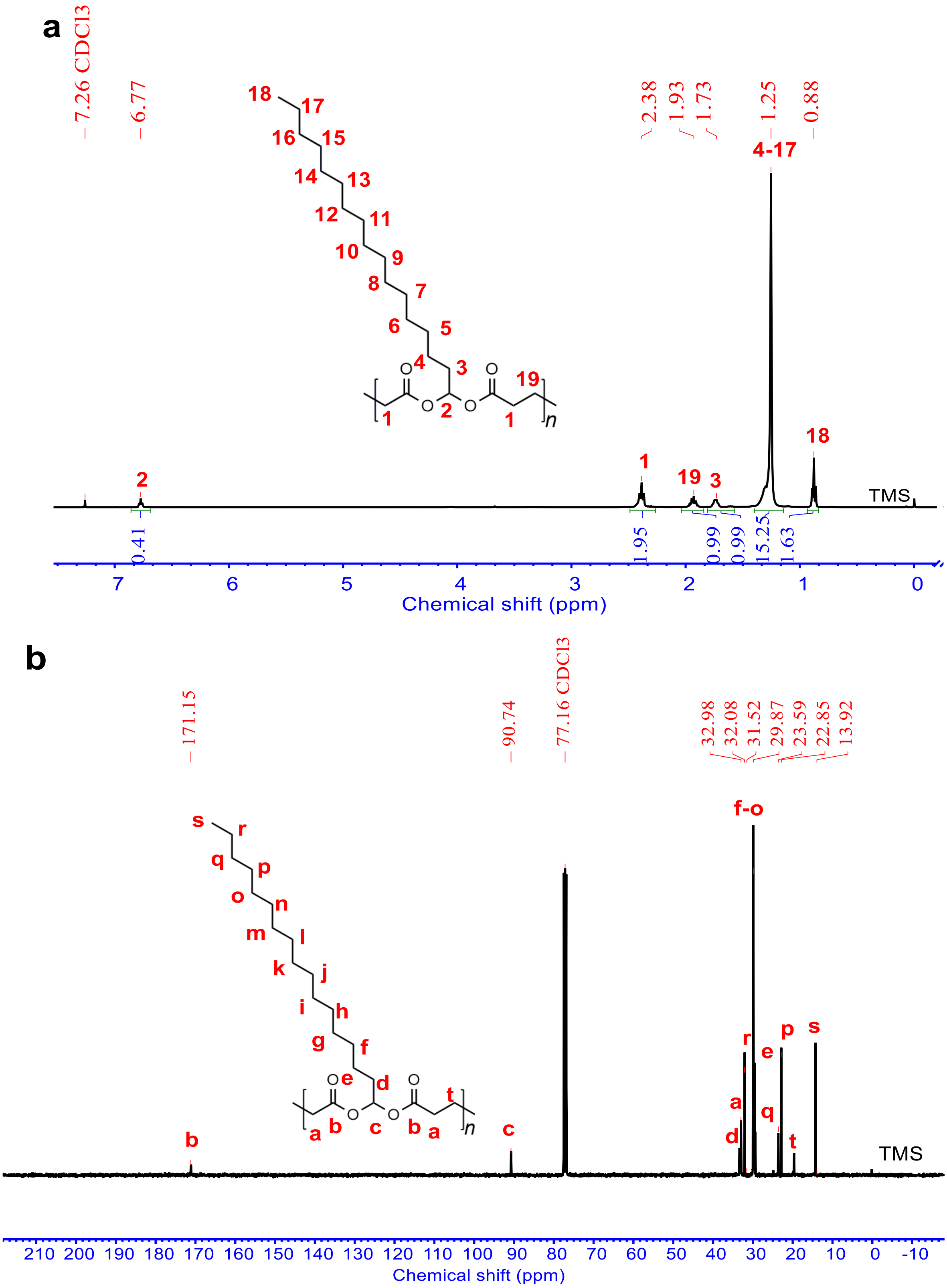

Supplementary Fig. 11 (a) ^{1}H and (b) ^{13}C NMR spectra of the obtained copolymer of **P10A** in $CDCl_3$.

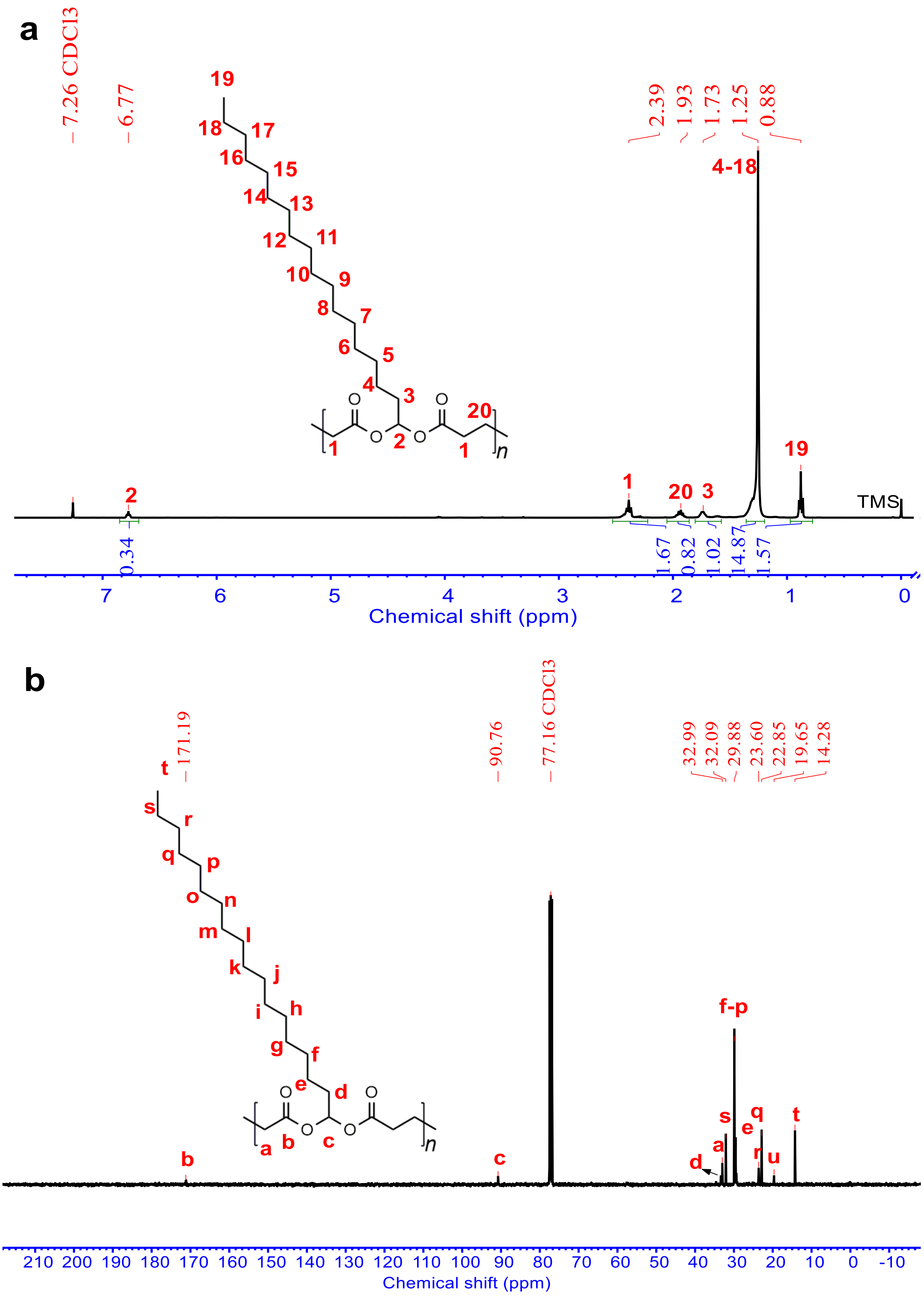

Supplementary Fig. 12 (a) ^{1}H and (b) ^{13}C NMR spectra of the obtained copolymer of **P11A** in $CDCl_3$.

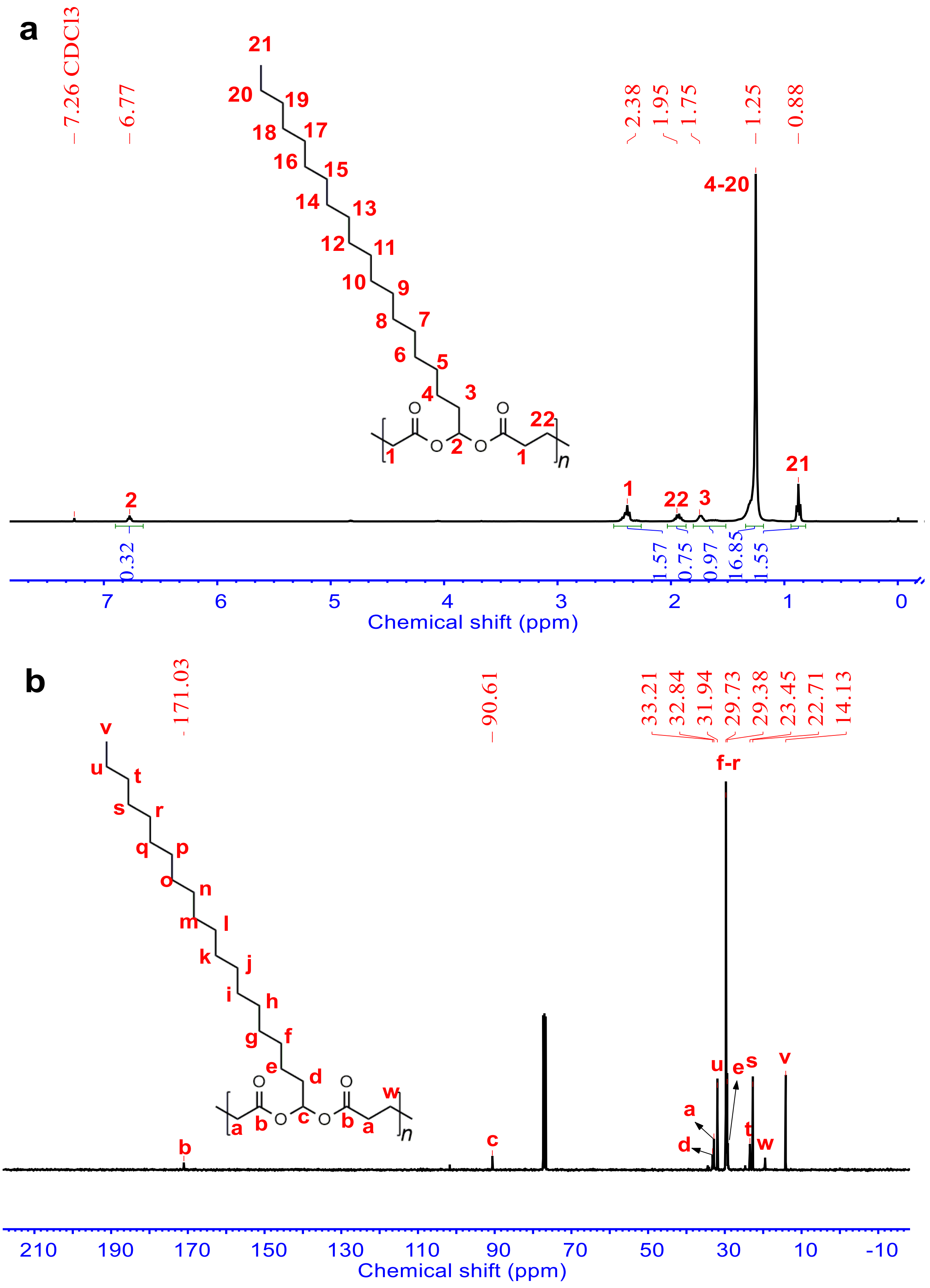

Supplementary Fig. 13 (a) ^{1}H and (b) ^{13}C NMR spectra of the obtained copolymer of **P12A** in $CDCl_3$.

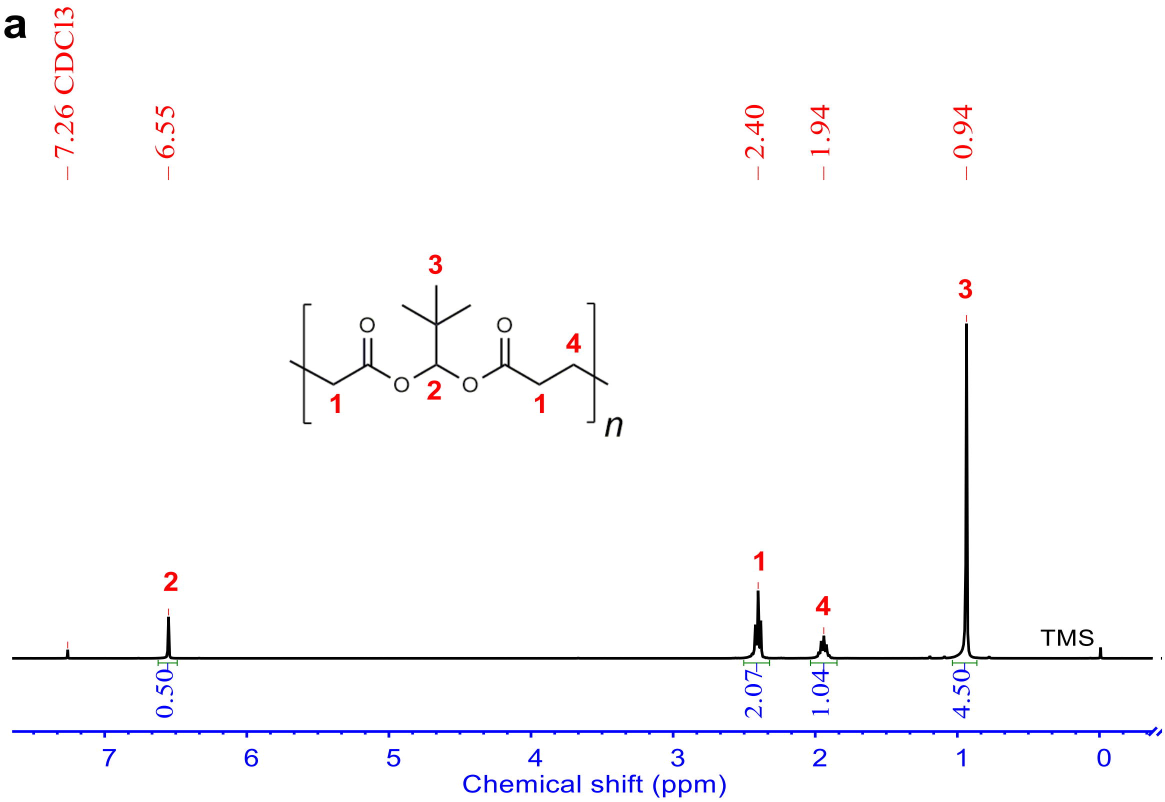

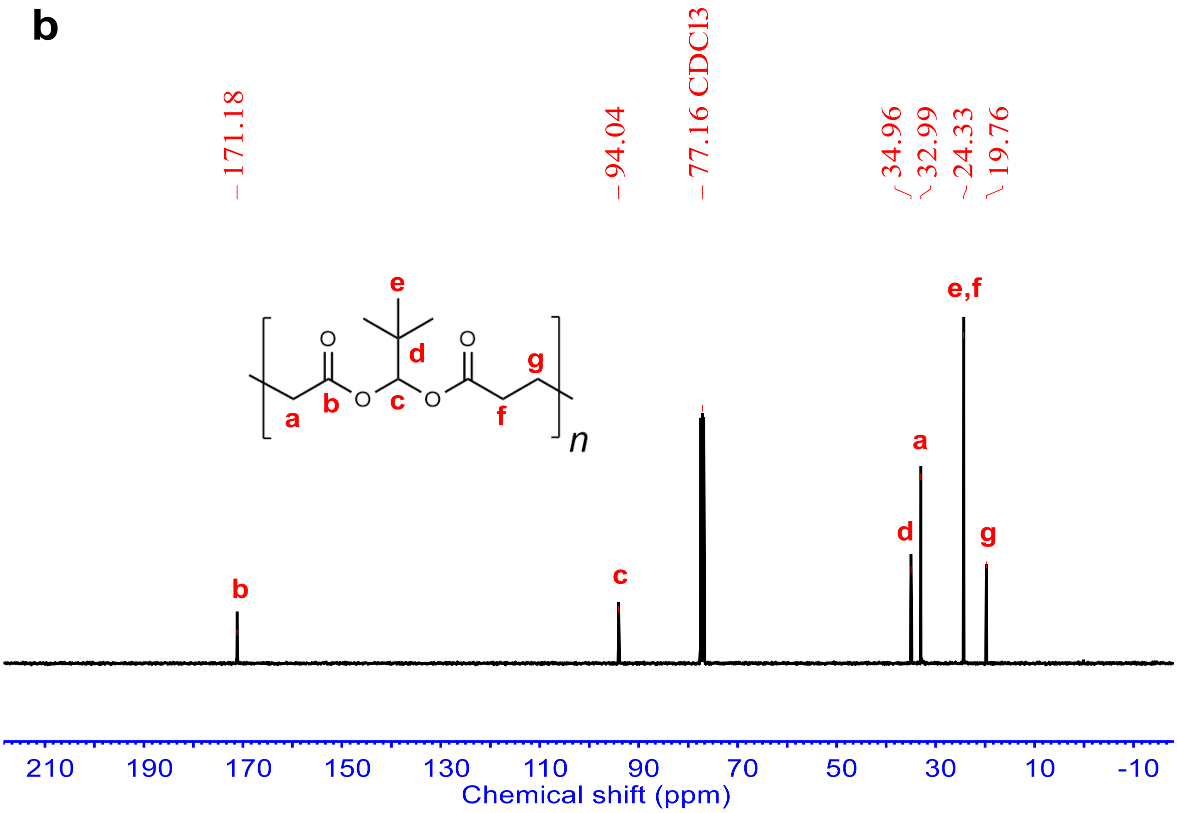

Supplementary Fig. 14 (a) ^{1}H and (b) ^{13}C NMR spectra of the obtained copolymer of **P13A** in $CDCl_3$.

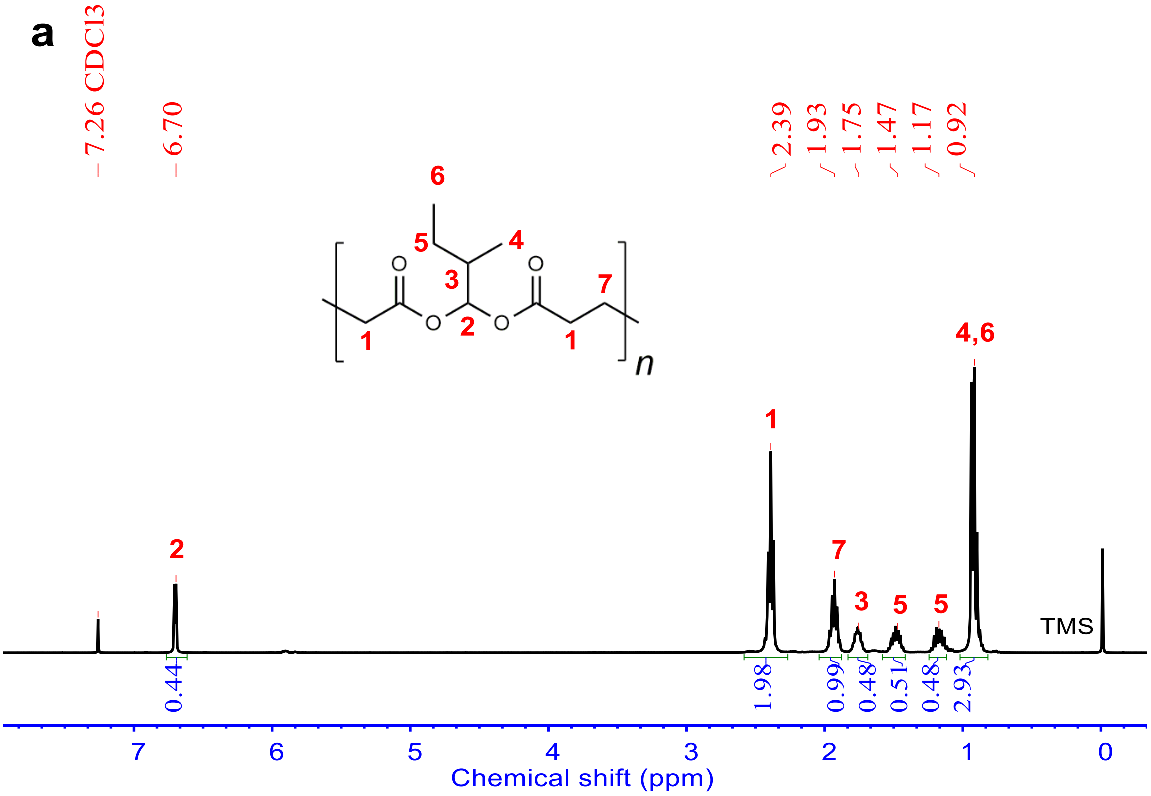

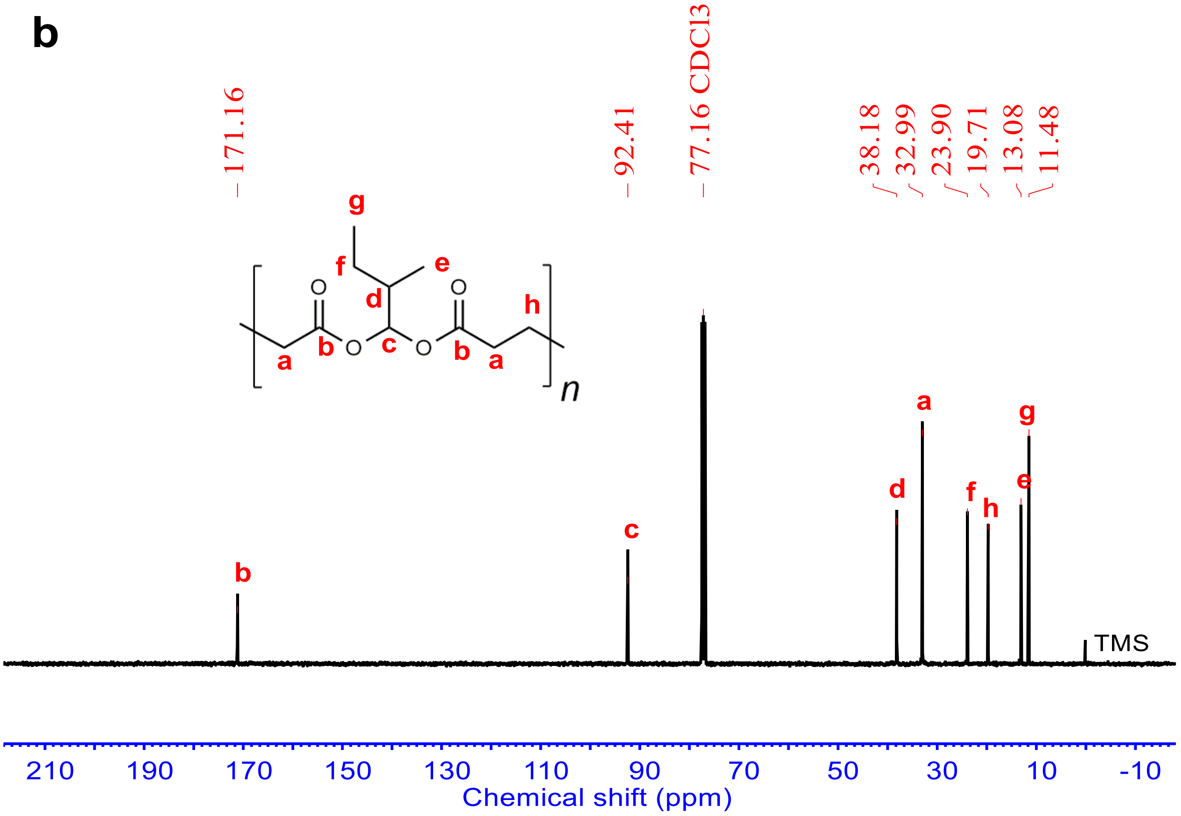

Supplementary Fig. 15 (a) ^{1}H and (b) ^{13}C NMR spectra of the obtained copolymer of **P14A** in $CDCl_3$.

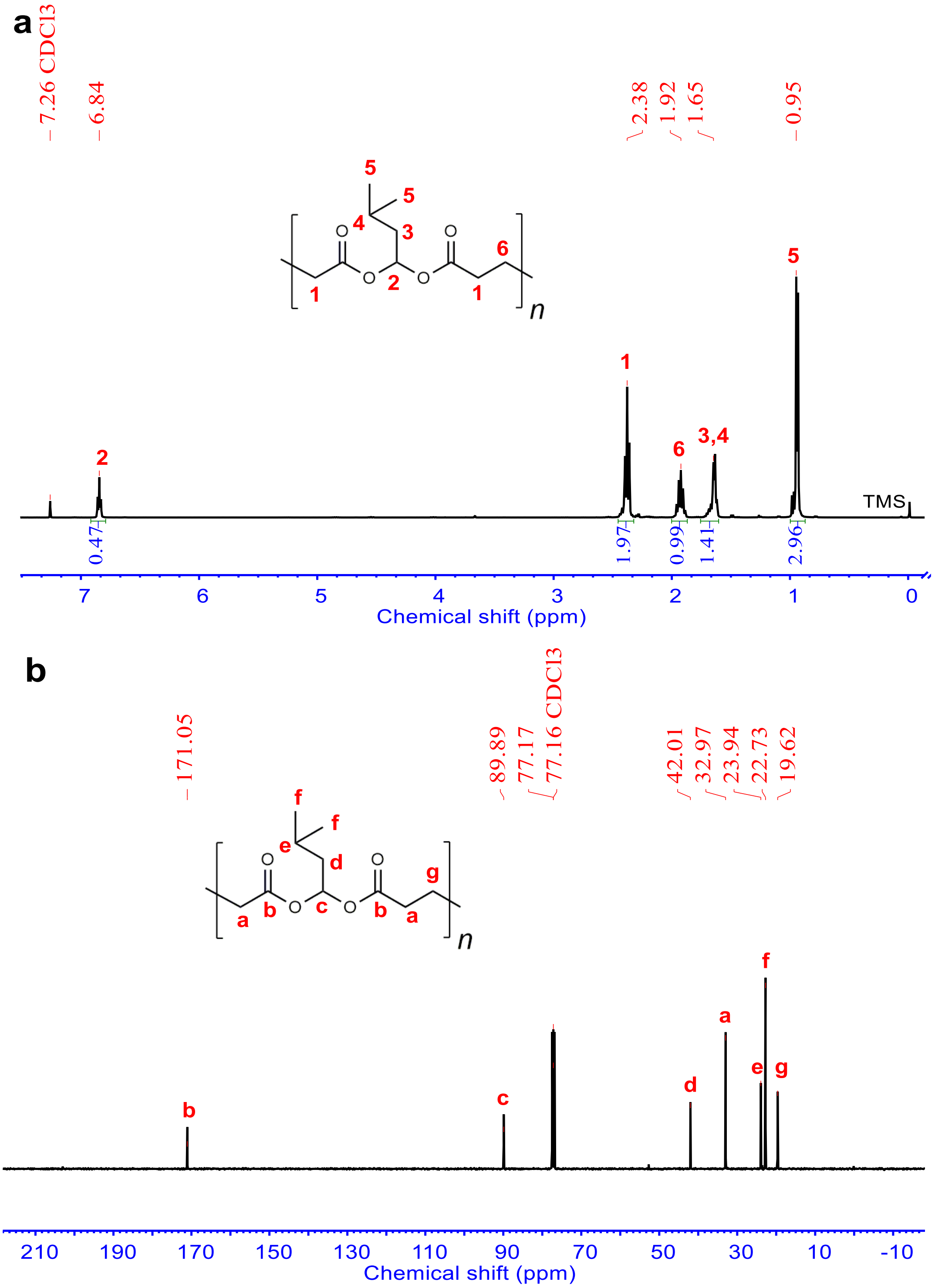

Supplementary Fig. 16 (a) ^{1}H and (b) ^{13}C NMR spectra of the obtained copolymer of **P15A** in $CDCl_3$.

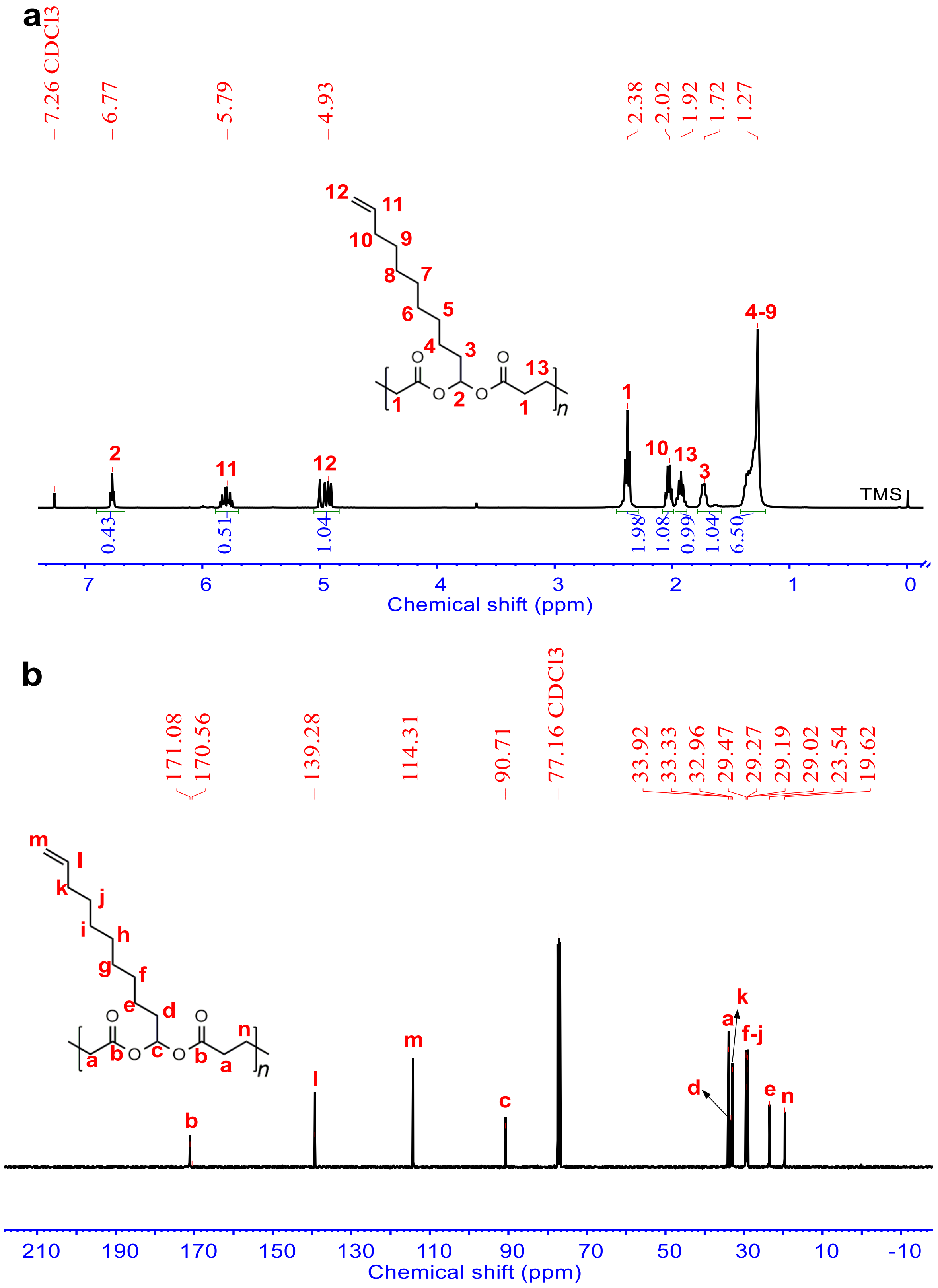

Supplementary Fig. 17 (a) ^{1}H and (b) ^{13}C NMR spectra of the obtained copolymer of **P16A** in $CDCl_3$.

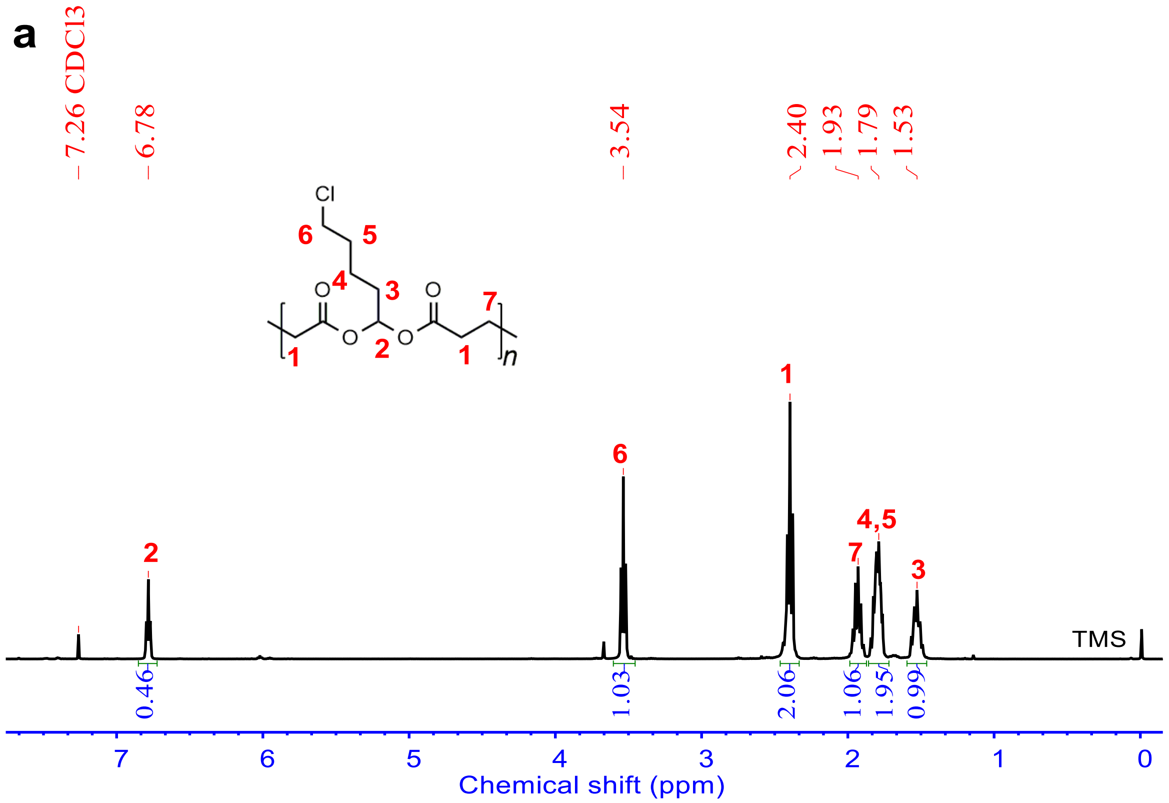

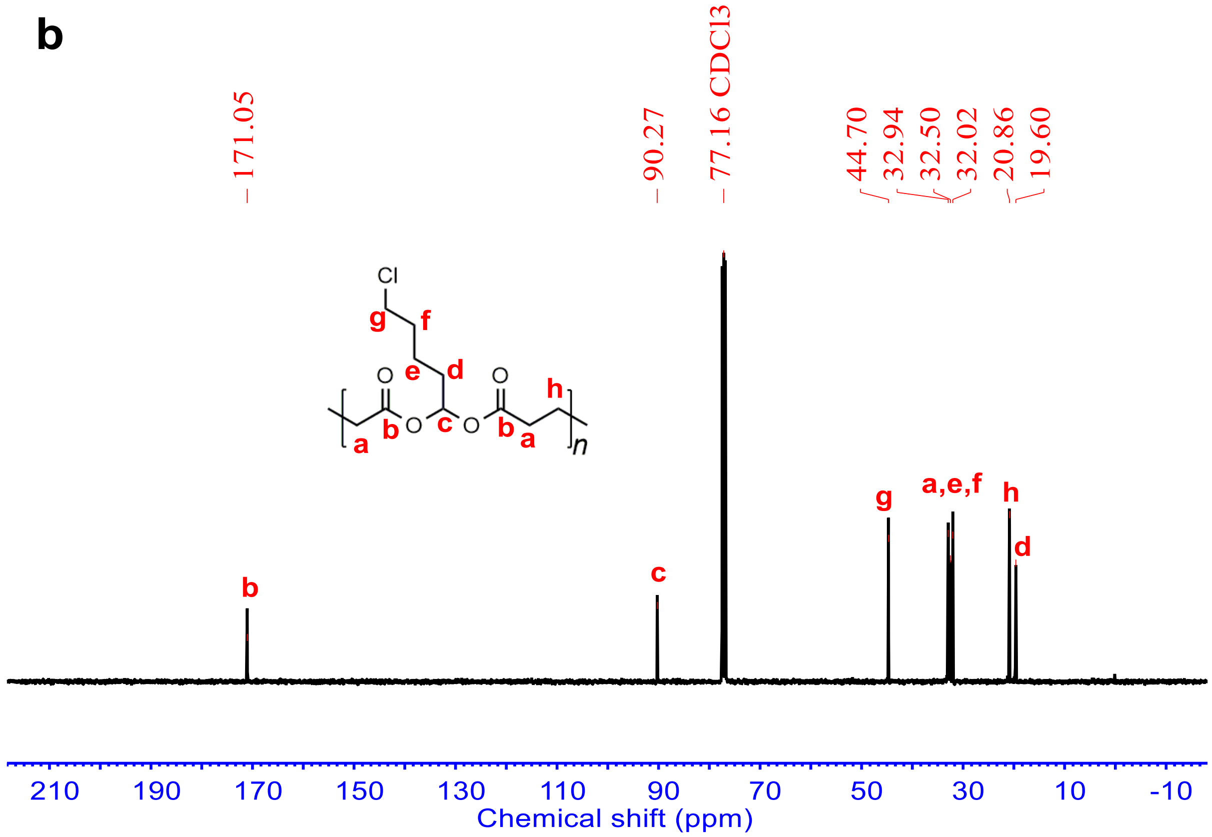

Supplementary Fig. 18 (a) ^{1}H and (b) ^{13}C NMR spectra of the obtained copolymer of **P17A** in $CDCl_3$.

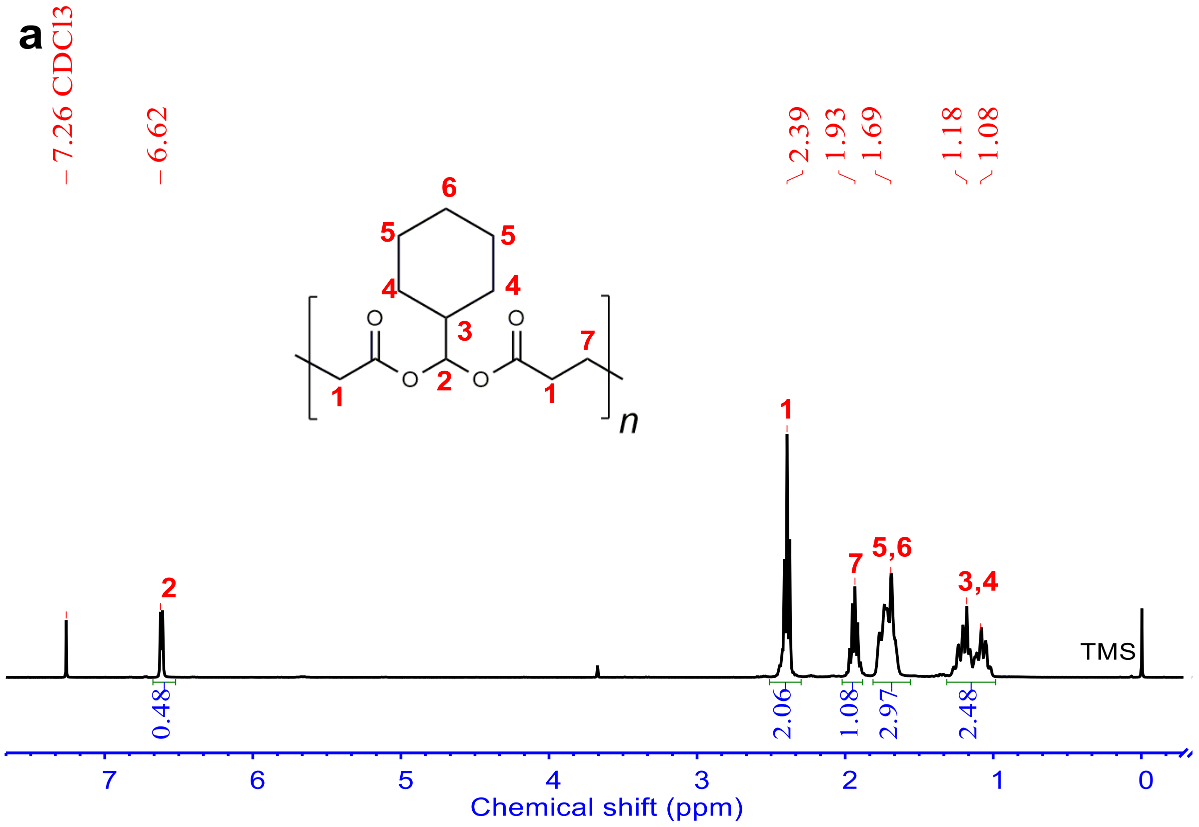

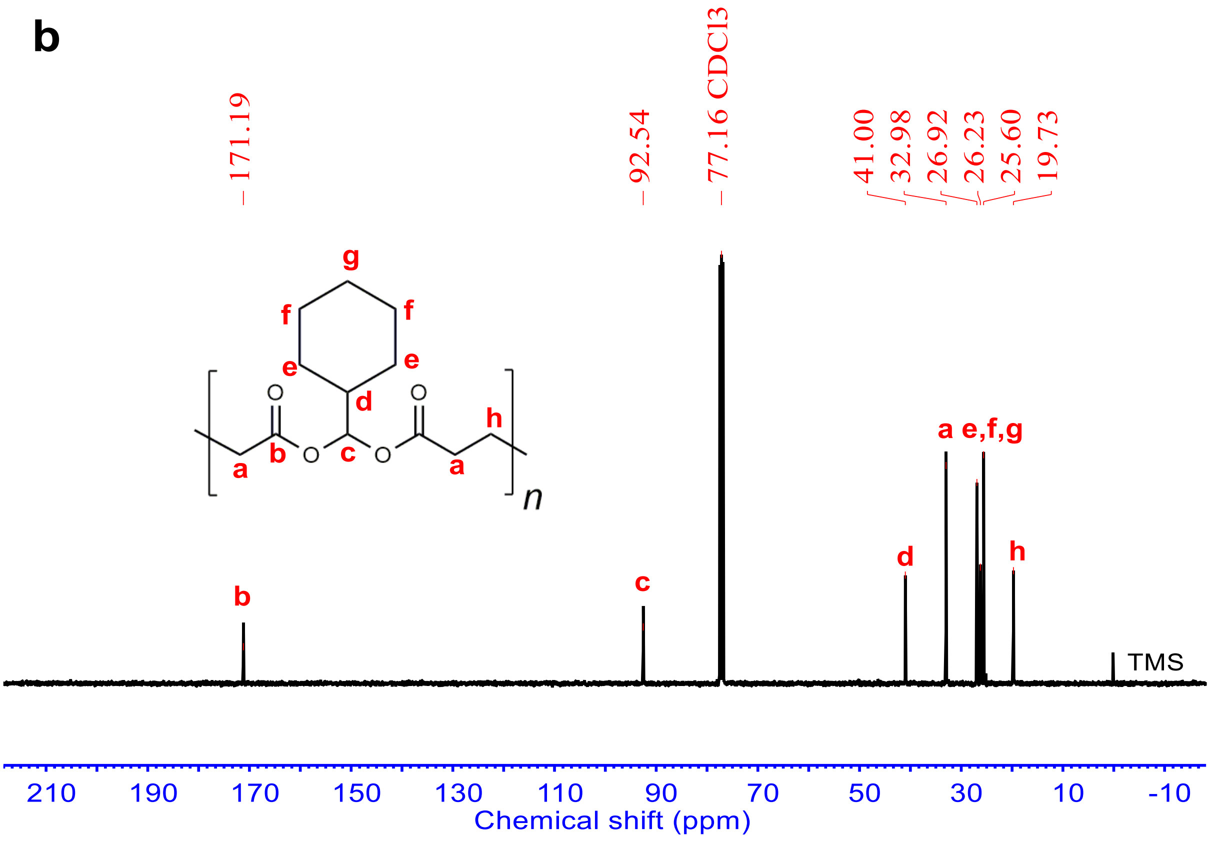

Supplementary Fig. 19 (a) ^{1}H and (b) ^{13}C NMR spectra of the obtained copolymer of **P18A** in $CDCl_3$.

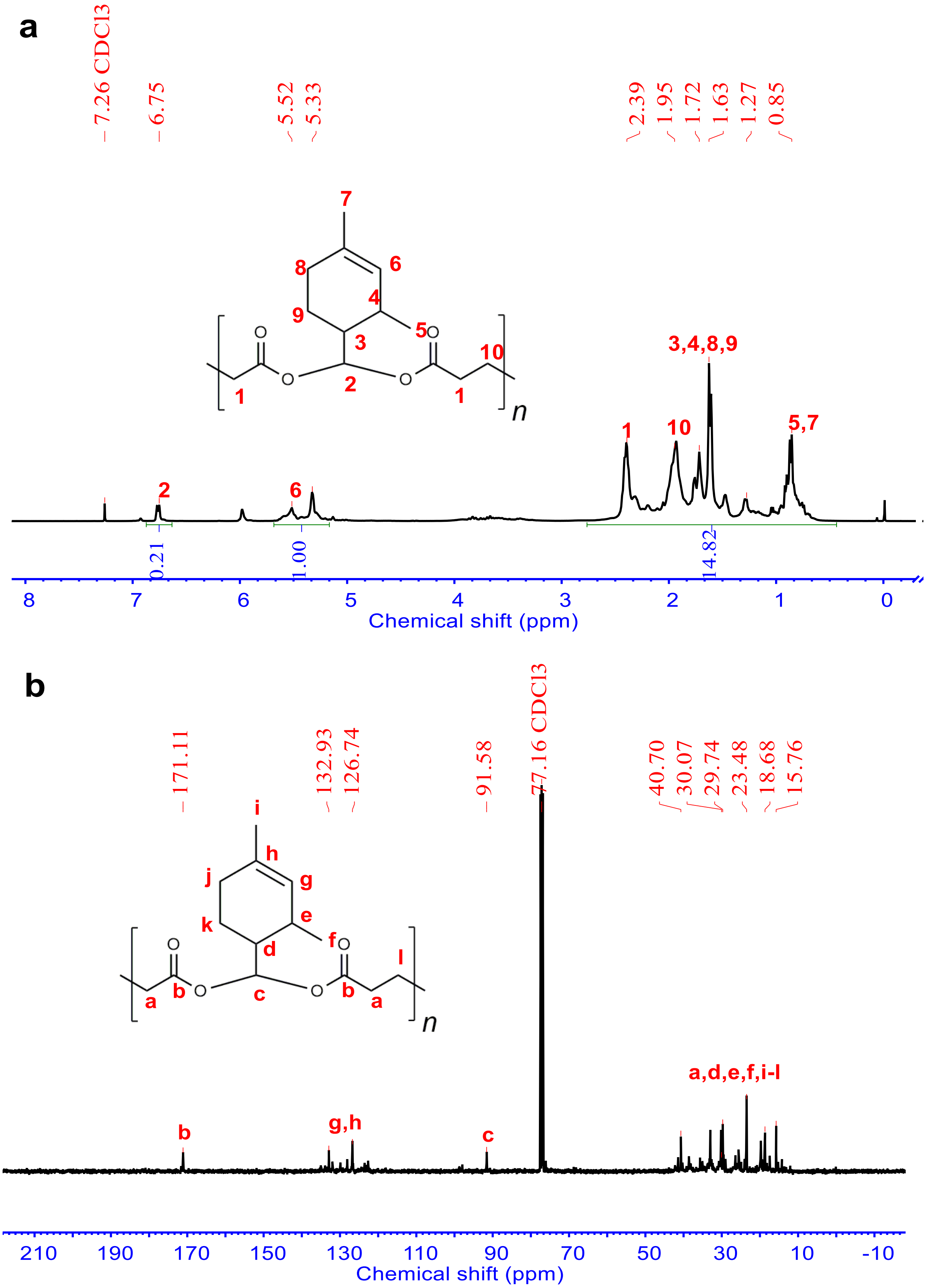

Supplementary Fig. 20 (a) ^{1}H and (b) ^{13}C NMR spectra of the obtained copolymer of **P19A** in $CDCl_3$.

a

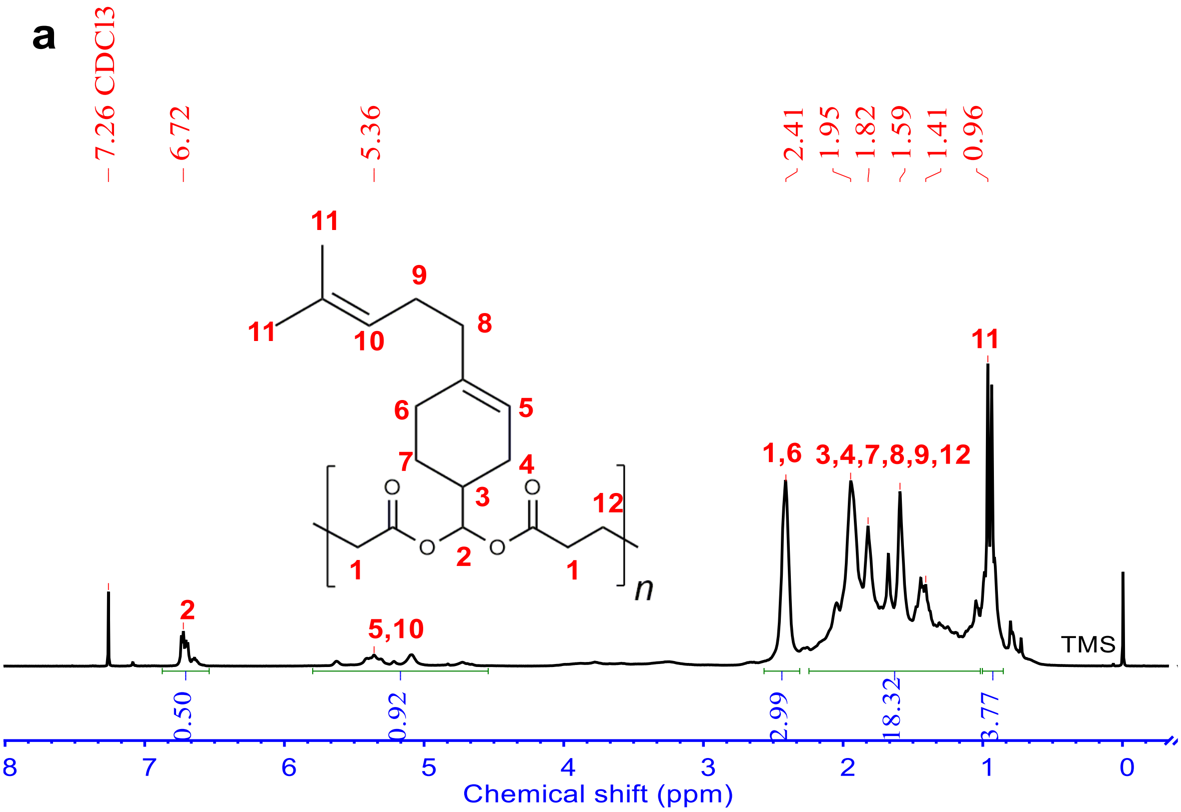

b

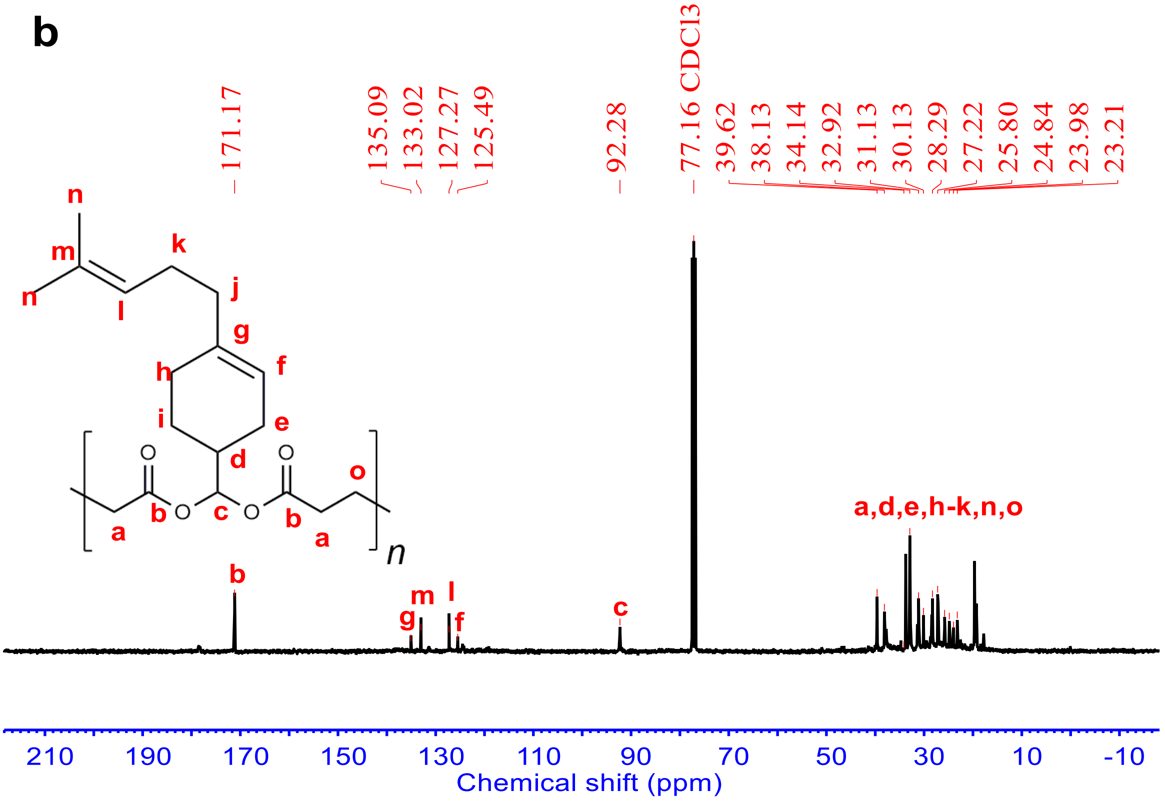

Supplementary Fig. 21 (a) ^{1}H and (b) ^{13}C NMR spectra of the obtained copolymer of **P20A** in $CDCl_3$.

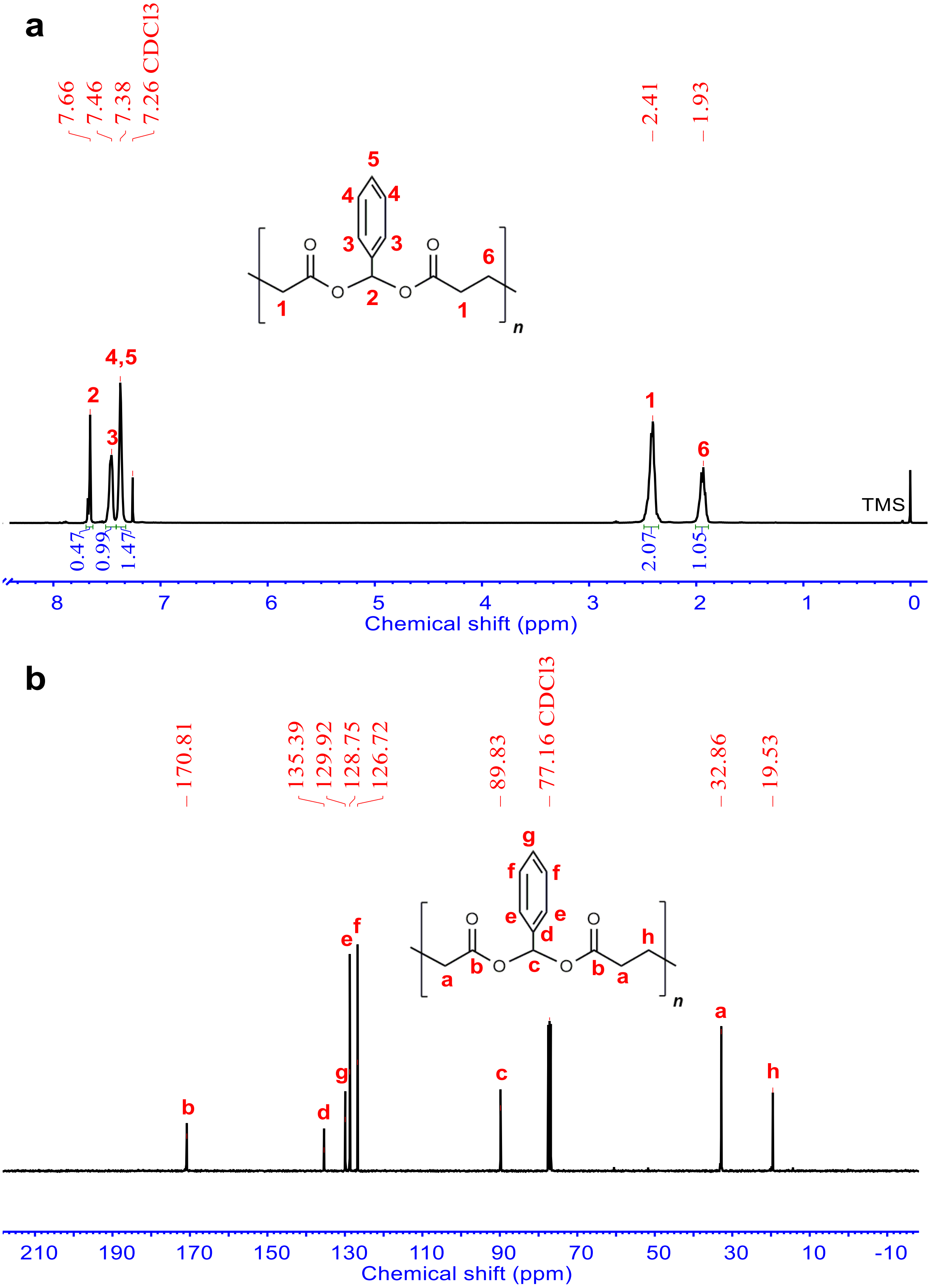

Supplementary Fig. 22 (a) ^{1}H and (b) ^{13}C NMR spectra of the obtained copolymer of **P21A** in $CDCl_3$.

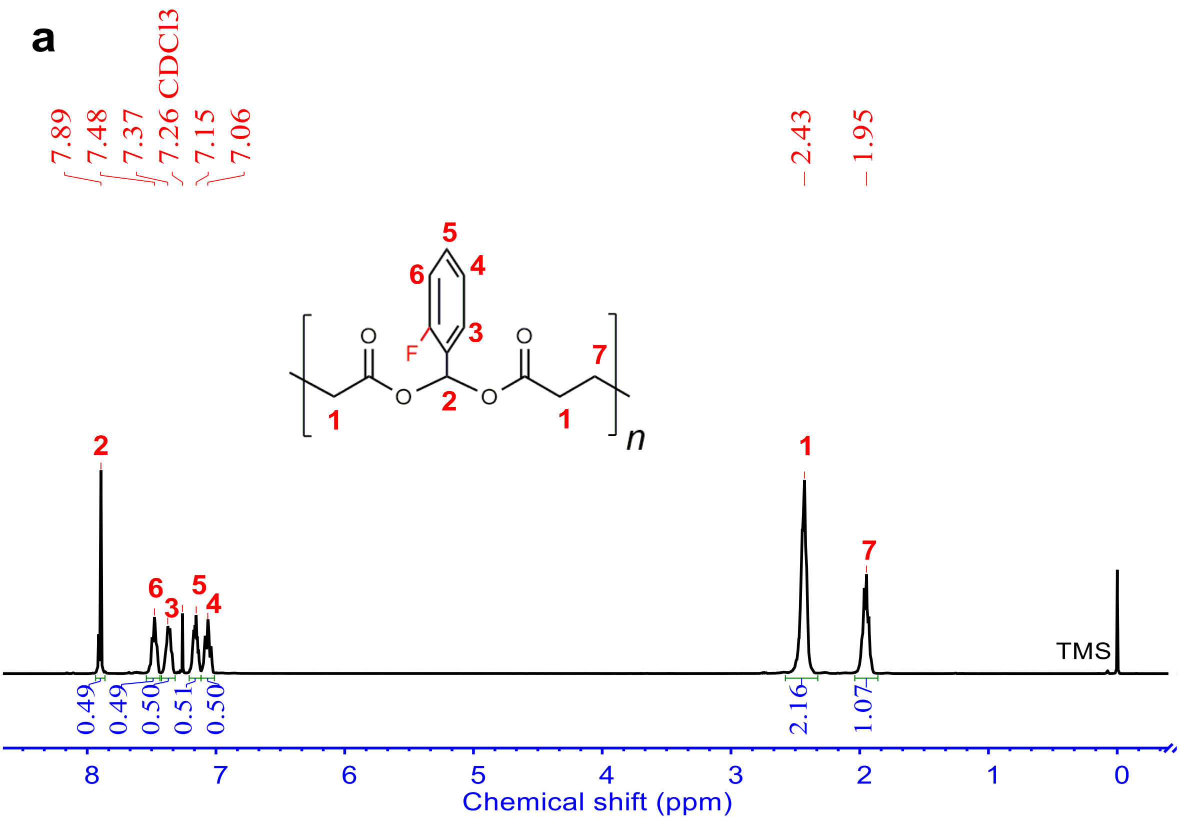

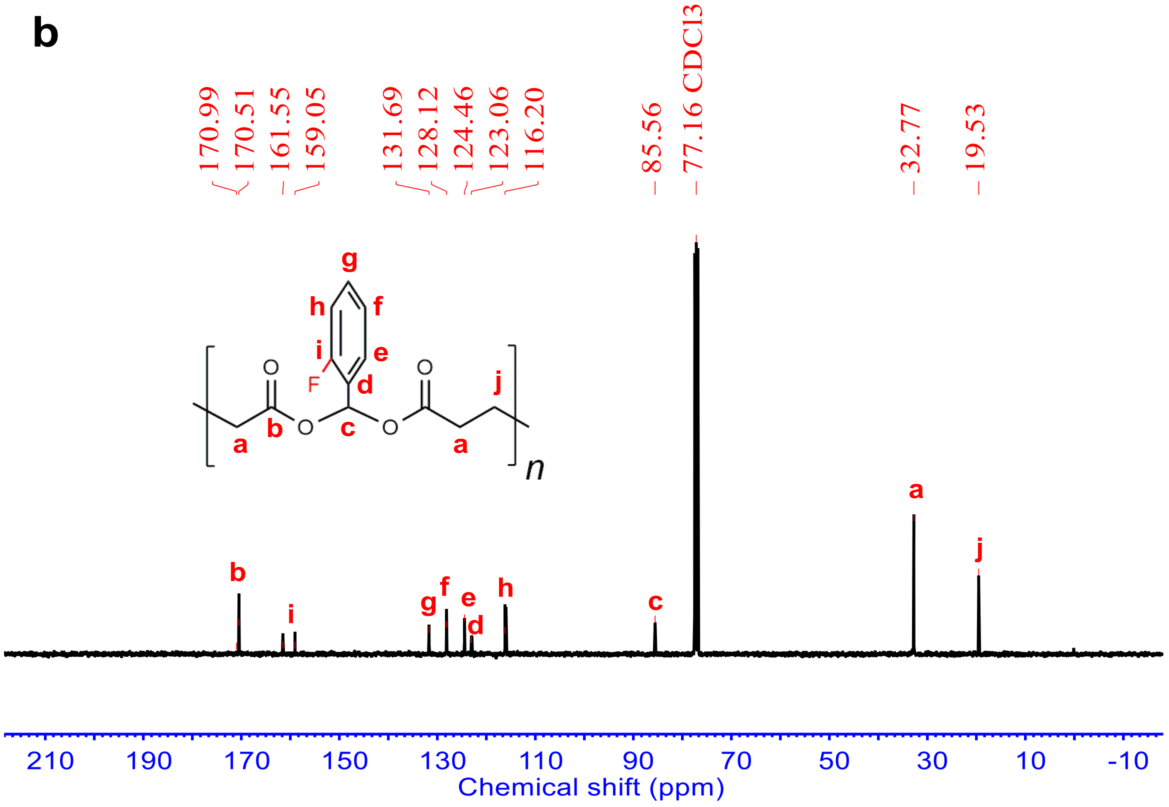

Supplementary Fig. 23 (a) ^{1}H and (b) ^{13}C NMR spectra of the obtained copolymer of **P22A** in $CDCl_3$.

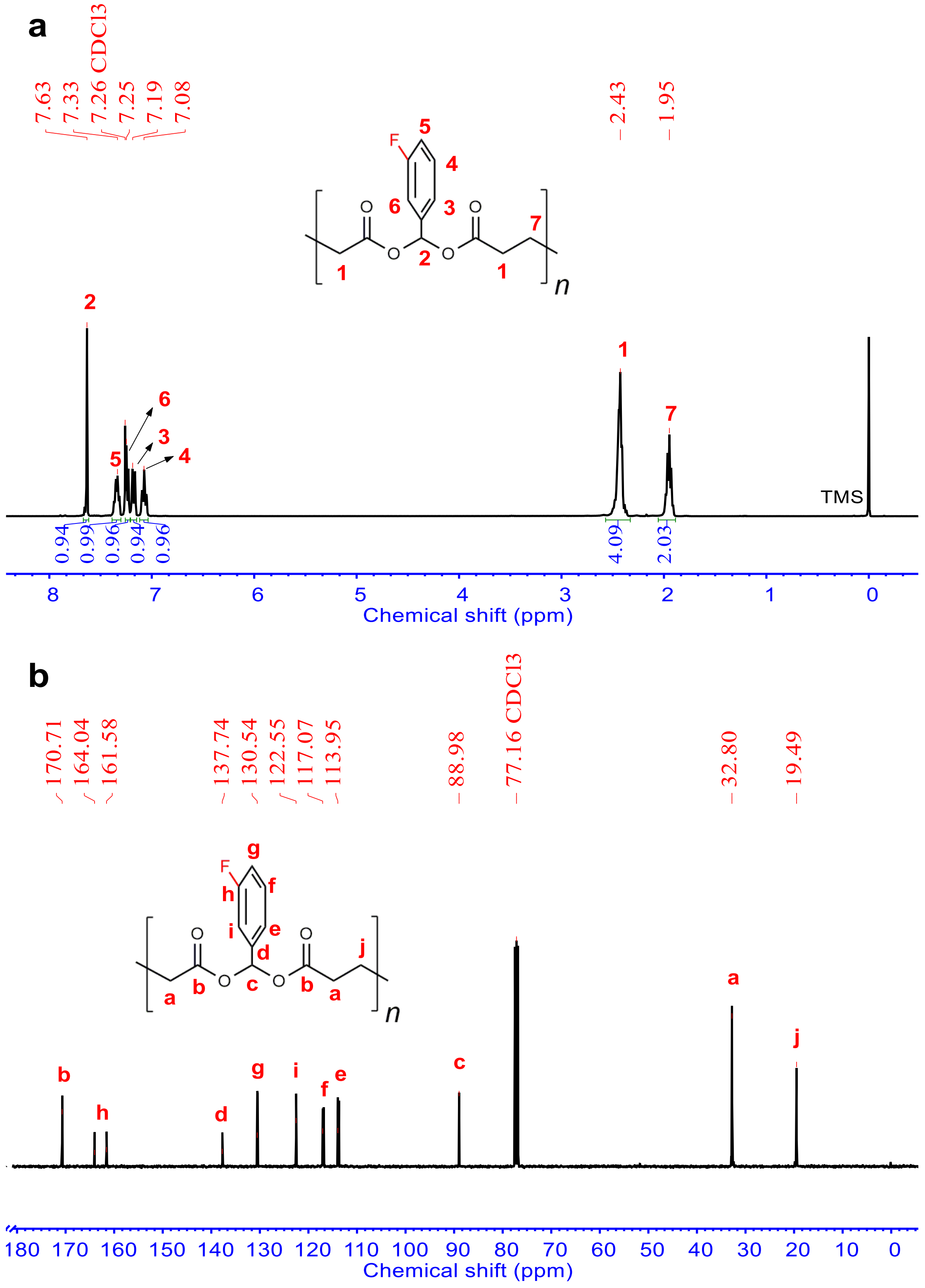

Supplementary Fig. 24 (a) ^{1}H and (b) ^{13}C NMR spectra of the obtained copolymer of **P23A** in $CDCl_3$.

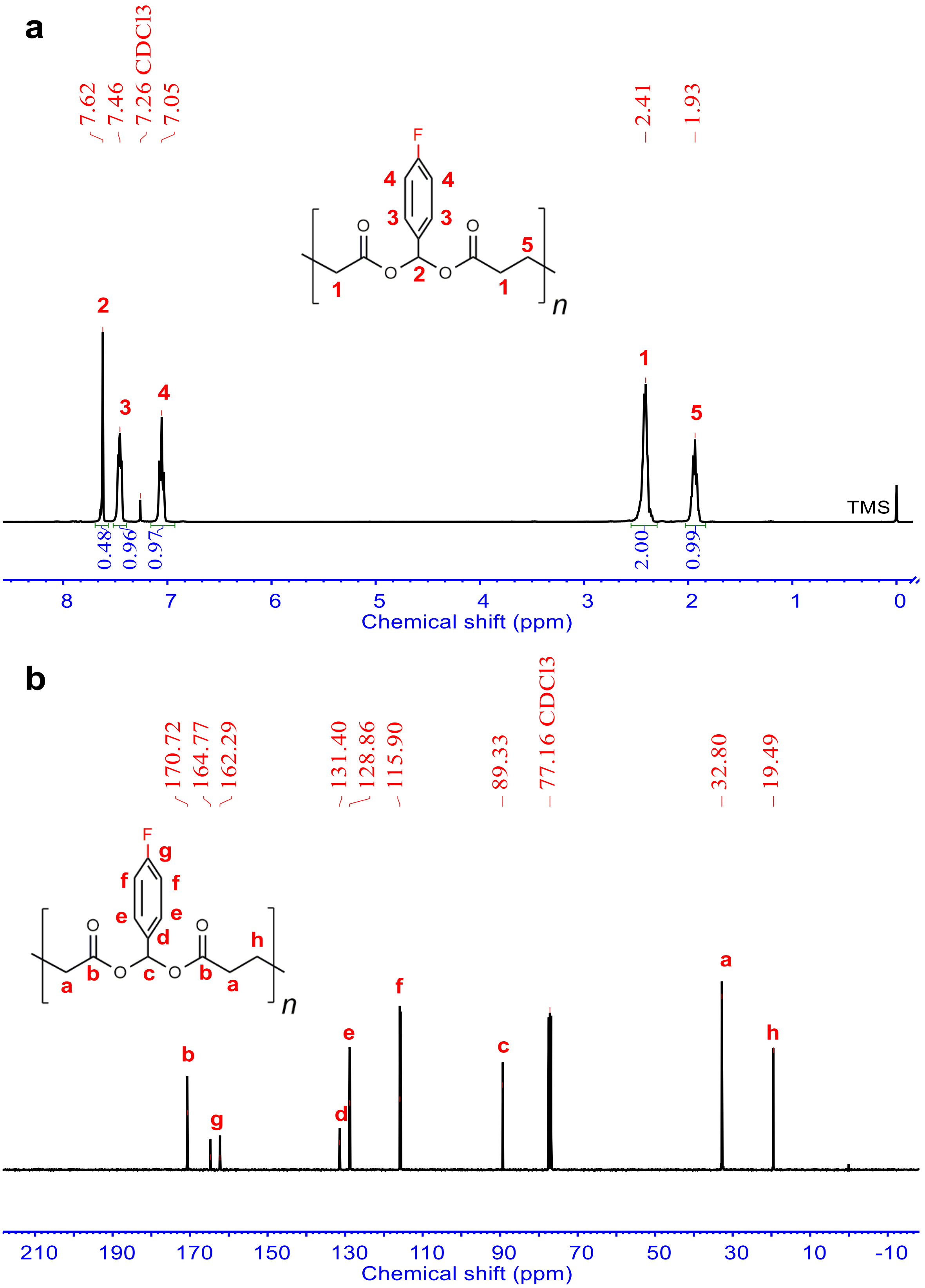

Supplementary Fig. 25 (a) ^{1}H and (b) ^{13}C NMR spectra of the obtained copolymer of **P24A** in $CDCl_3$.

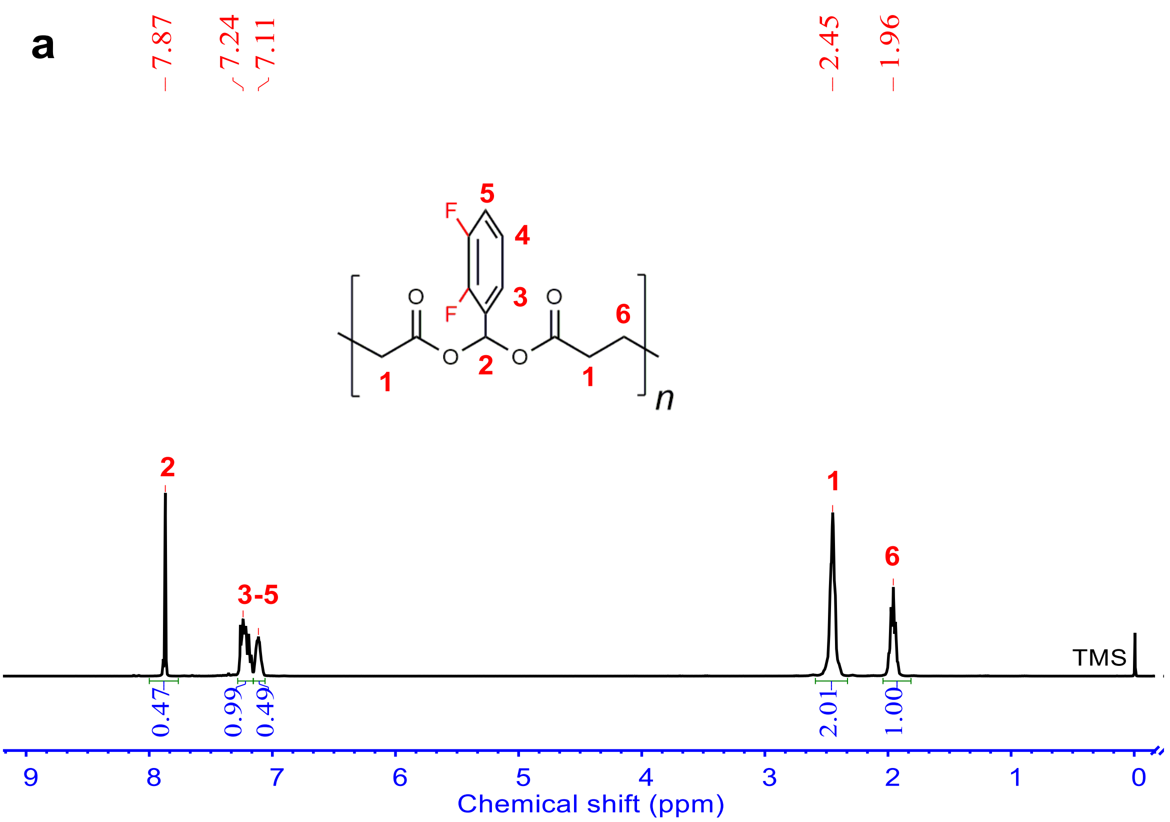

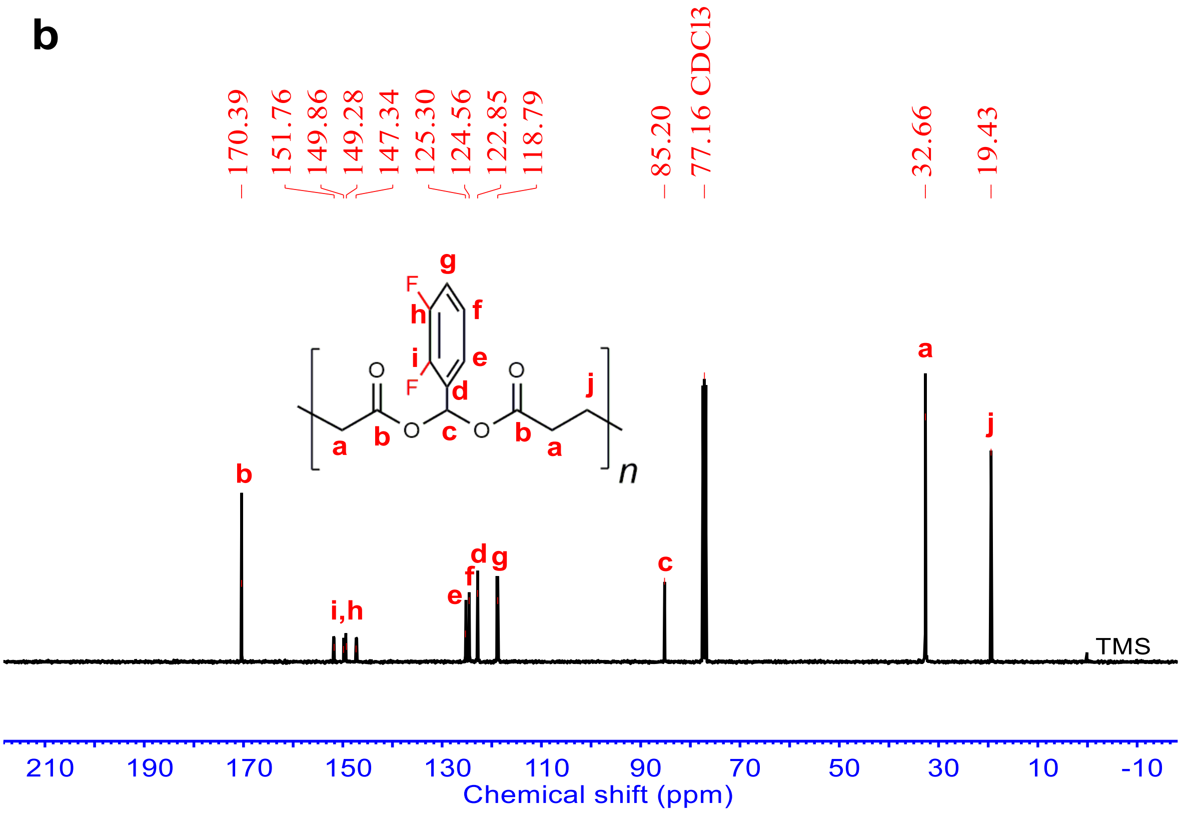

Supplementary Fig. 26 (a) ^{1}H and (b) ^{13}C NMR spectra of the obtained copolymer of **P25A** in $CDCl_3$.

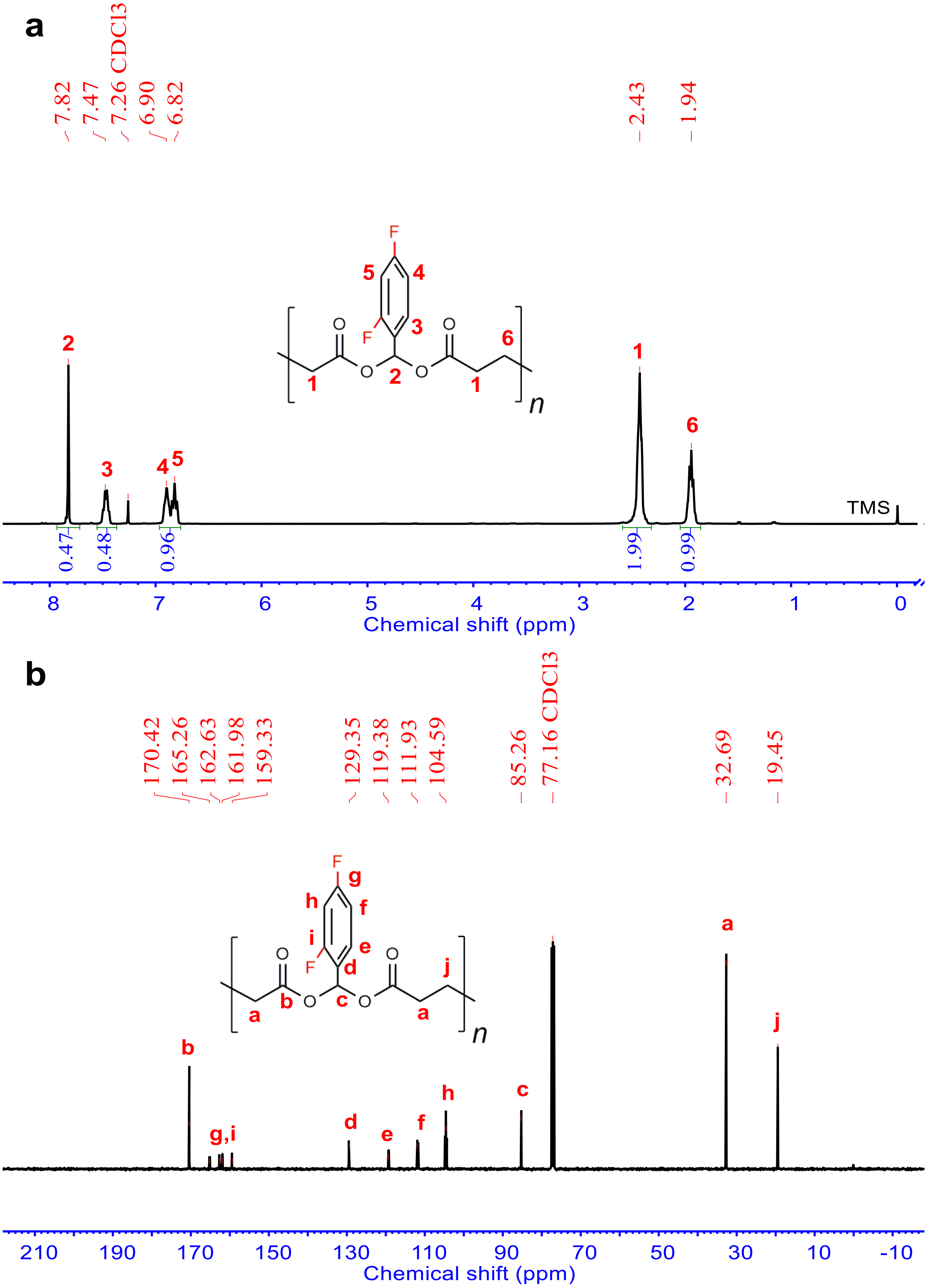

Supplementary Fig. 27 (a) ^{1}H and (b) ^{13}C NMR spectra of the obtained copolymer of **P26A** in $CDCl_3$.

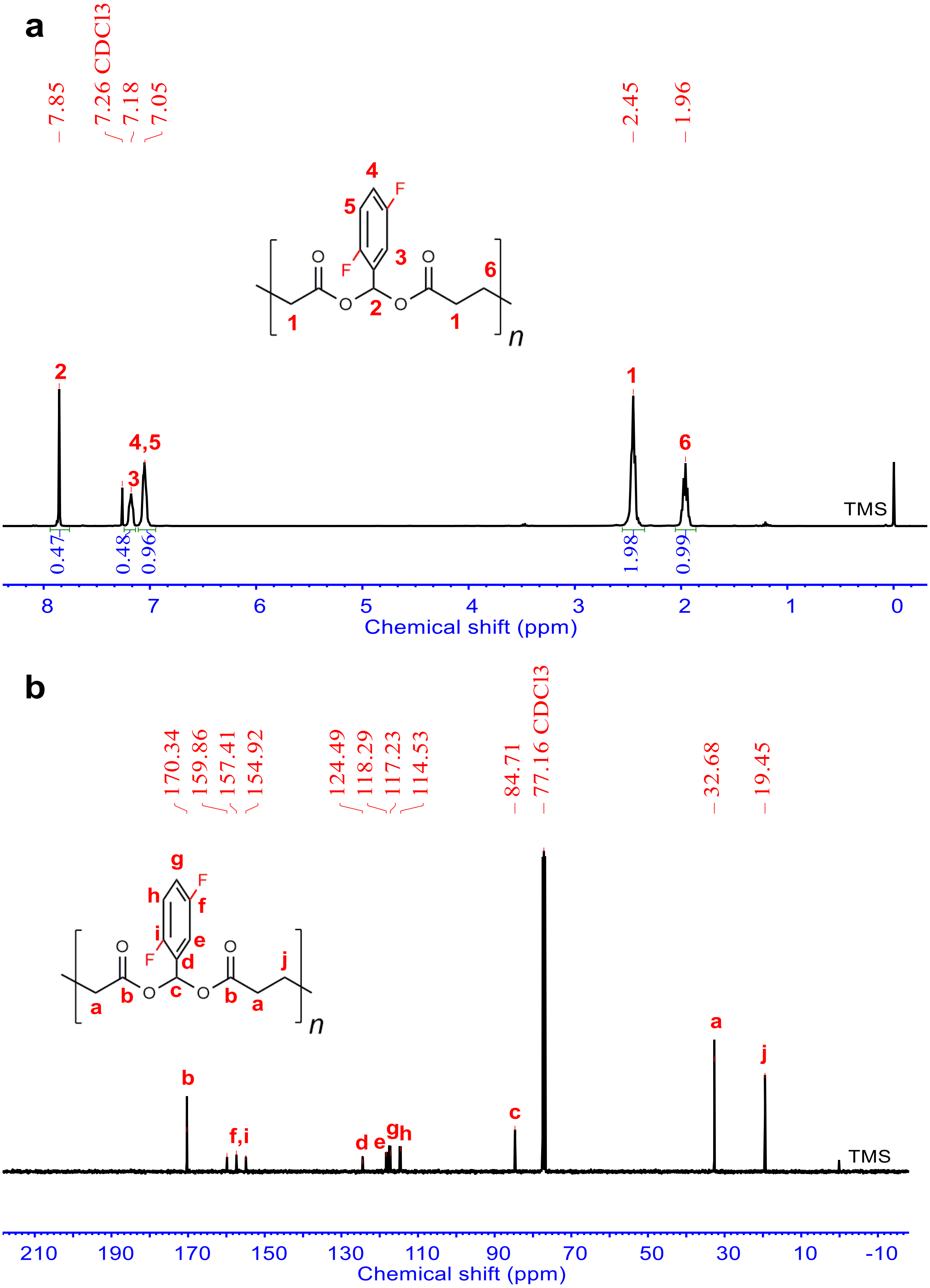

Supplementary Fig. 28 (a) ^{1}H and (b) ^{13}C NMR spectra of the obtained copolymer of **P27A** in $CDCl_3$.

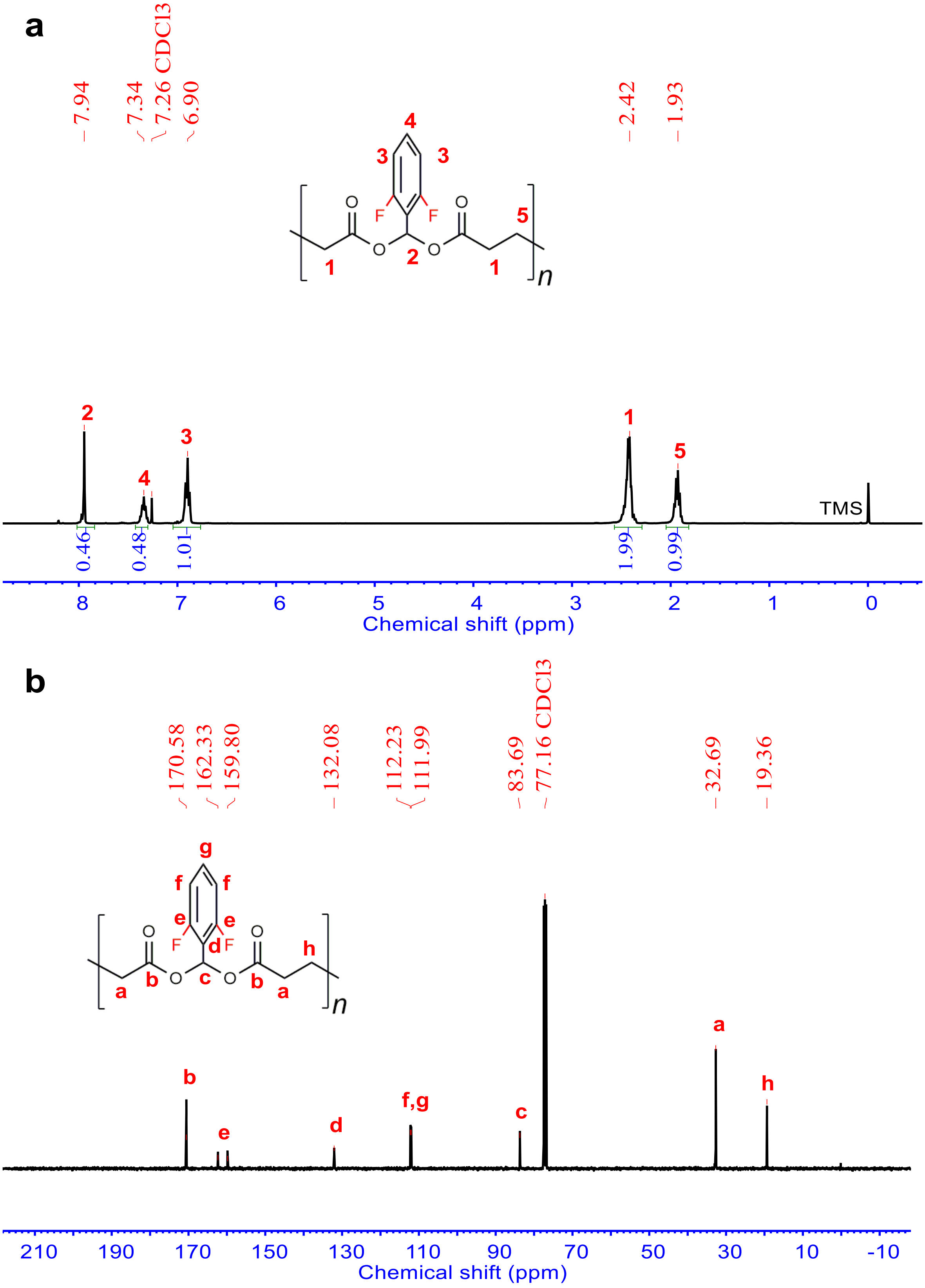

Supplementary Fig. 29 (a) ^{1}H and (b) ^{13}C NMR spectra of the obtained copolymer of **P28A** in $CDCl_3$.

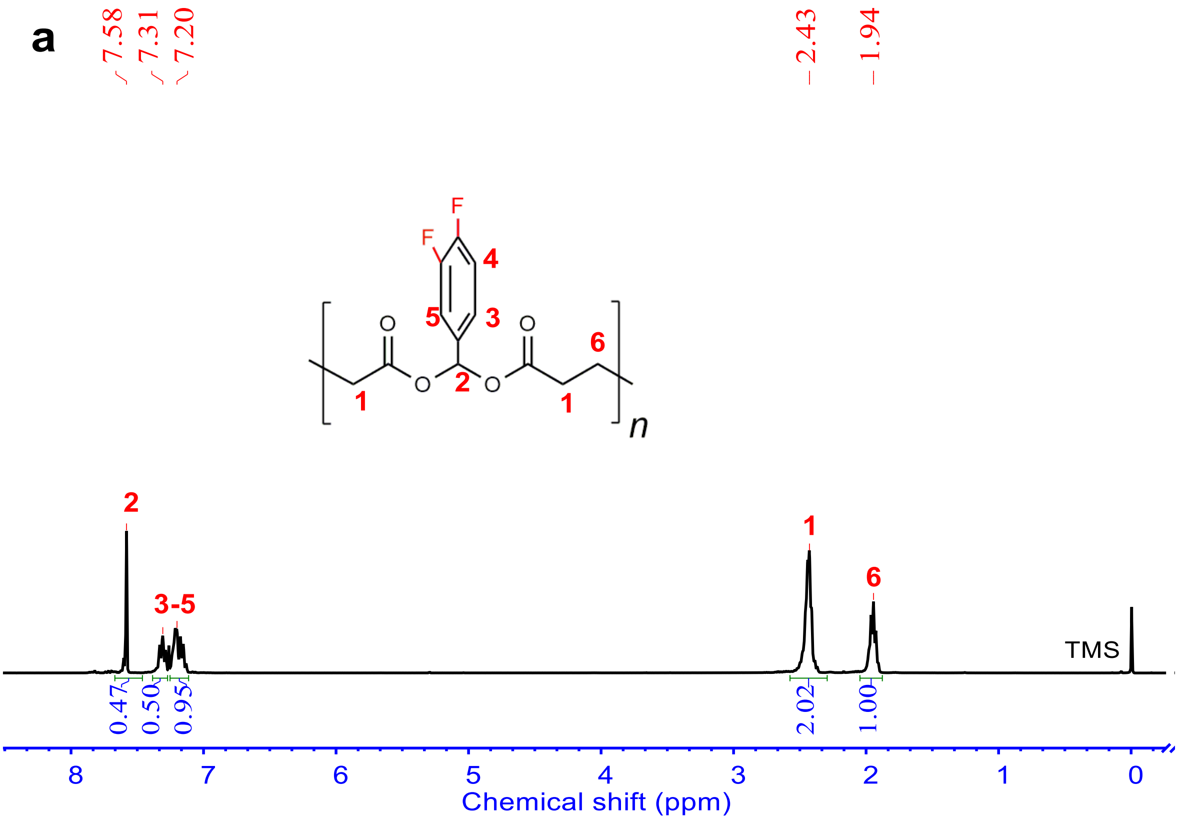

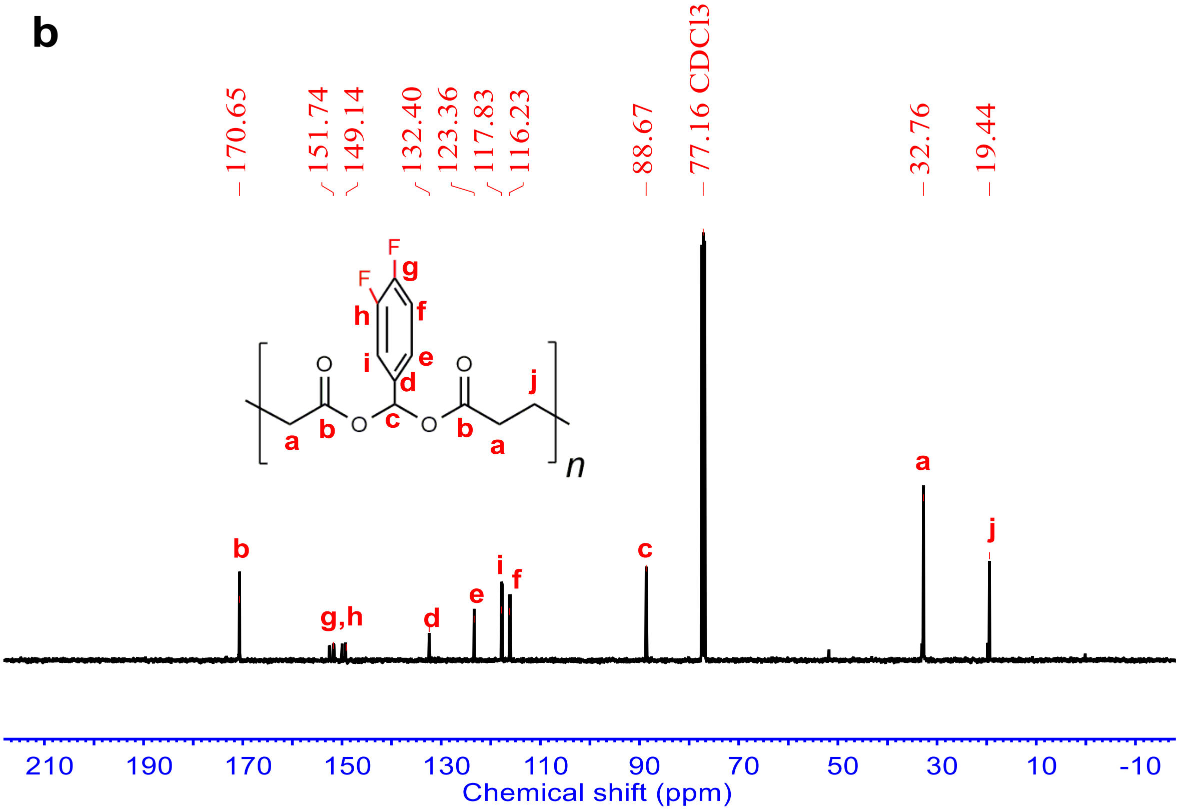

Supplementary Fig. 30 (a) ^{1}H and (b) ^{13}C NMR spectra of the obtained copolymer of **P29A** in $CDCl_3$.

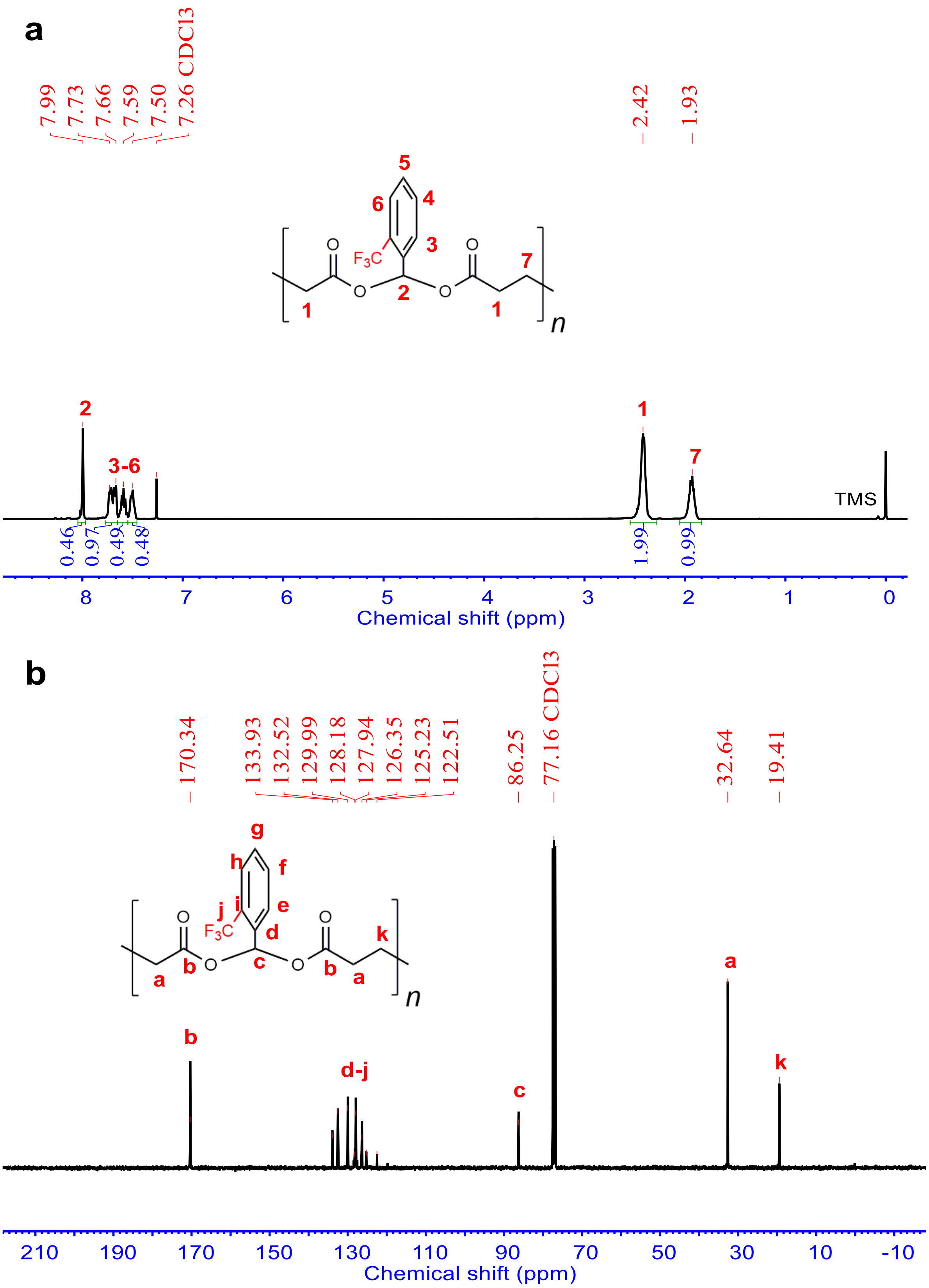

Supplementary Fig. 31 (a) ^{1}H and (b) ^{13}C NMR spectra of the obtained copolymer of **P30A** in $CDCl_3$.

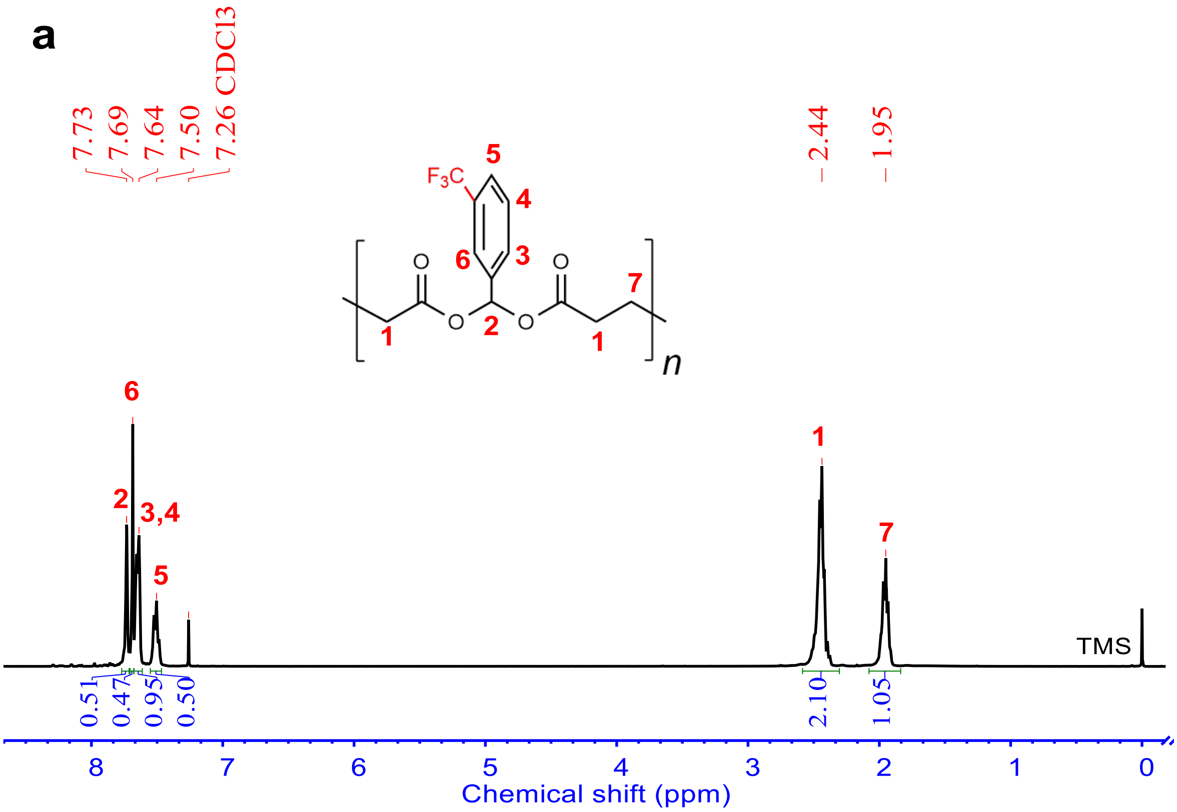

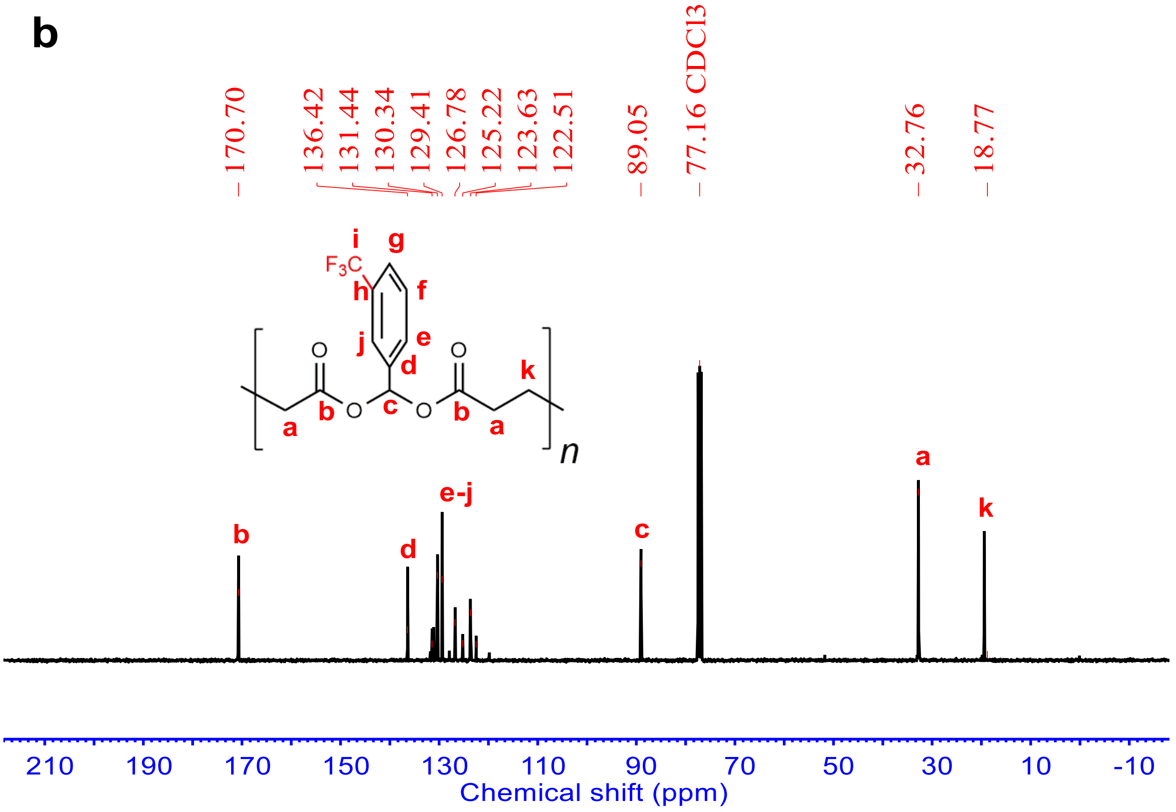

Supplementary Fig. 32 (a) ^{1}H and (b) ^{13}C NMR spectra of the obtained copolymer of **P31A** in $CDCl_3$.

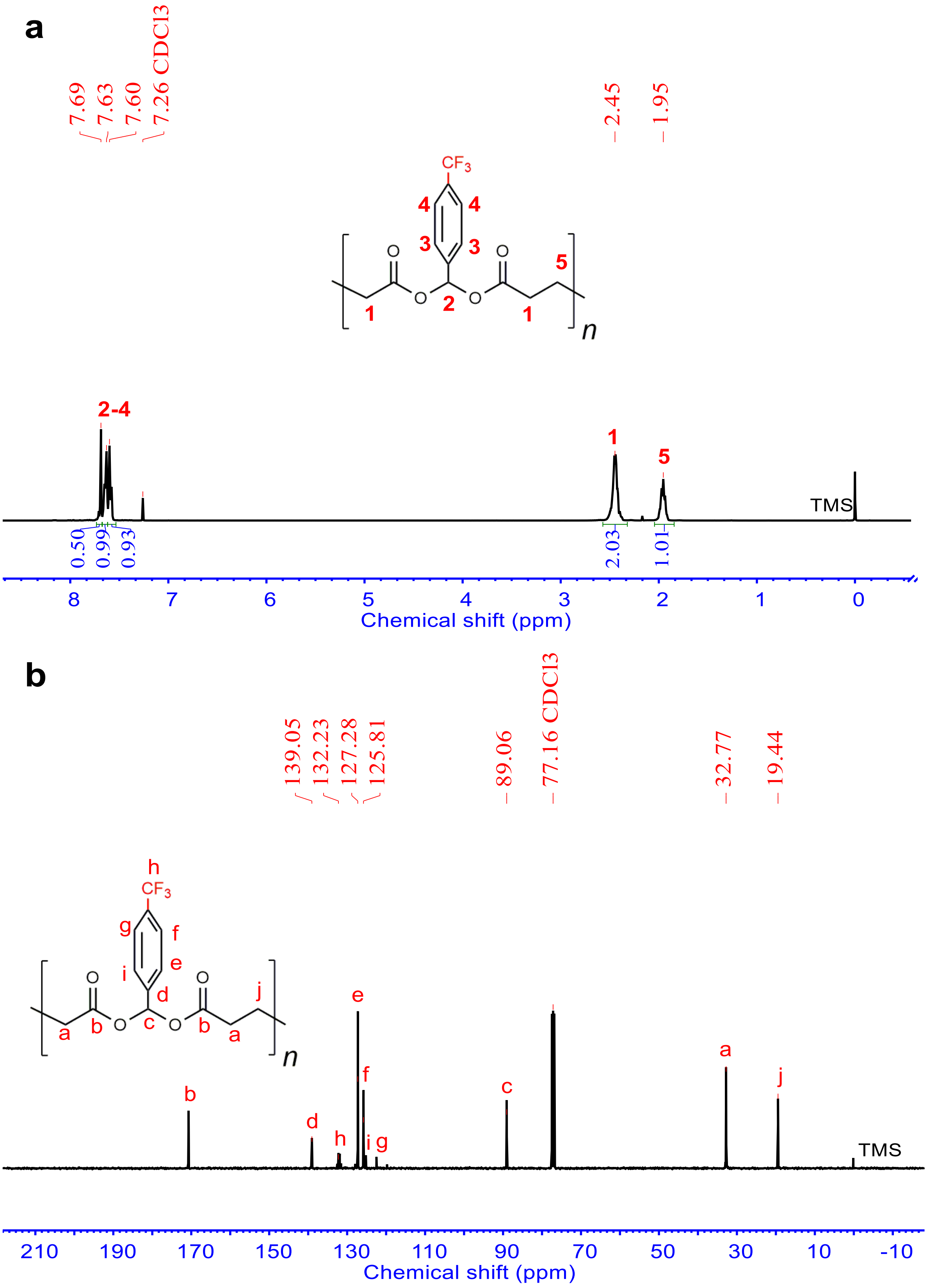

Supplementary Fig. 33 (a) ^{1}H and (b) ^{13}C NMR spectra of the obtained copolymer of **P32A** in $CDCl_3$.

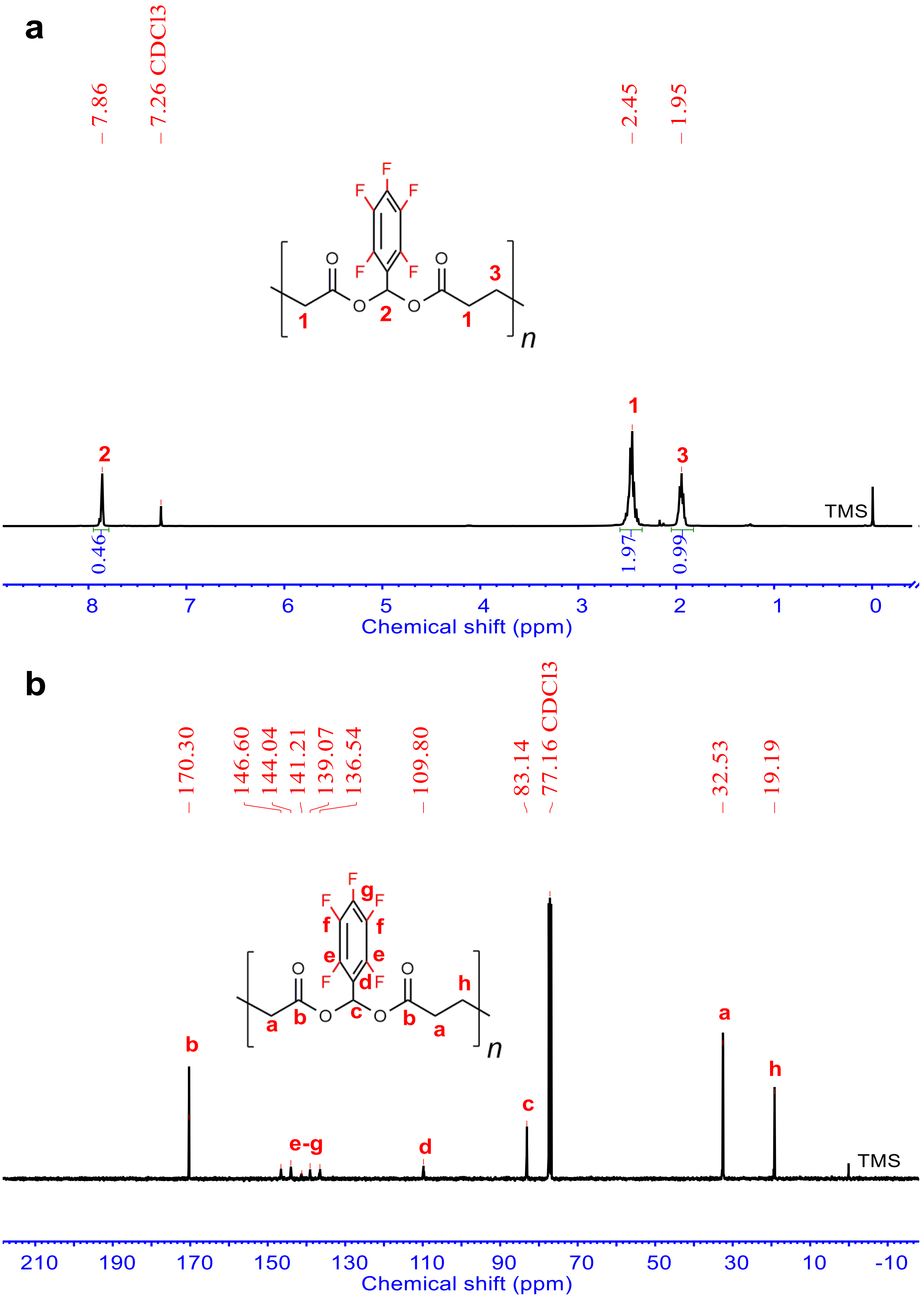

Supplementary Fig. 34 (a) ^{1}H and (b) ^{13}C NMR spectra of the obtained copolymer of **P33A** in $CDCl_3$.

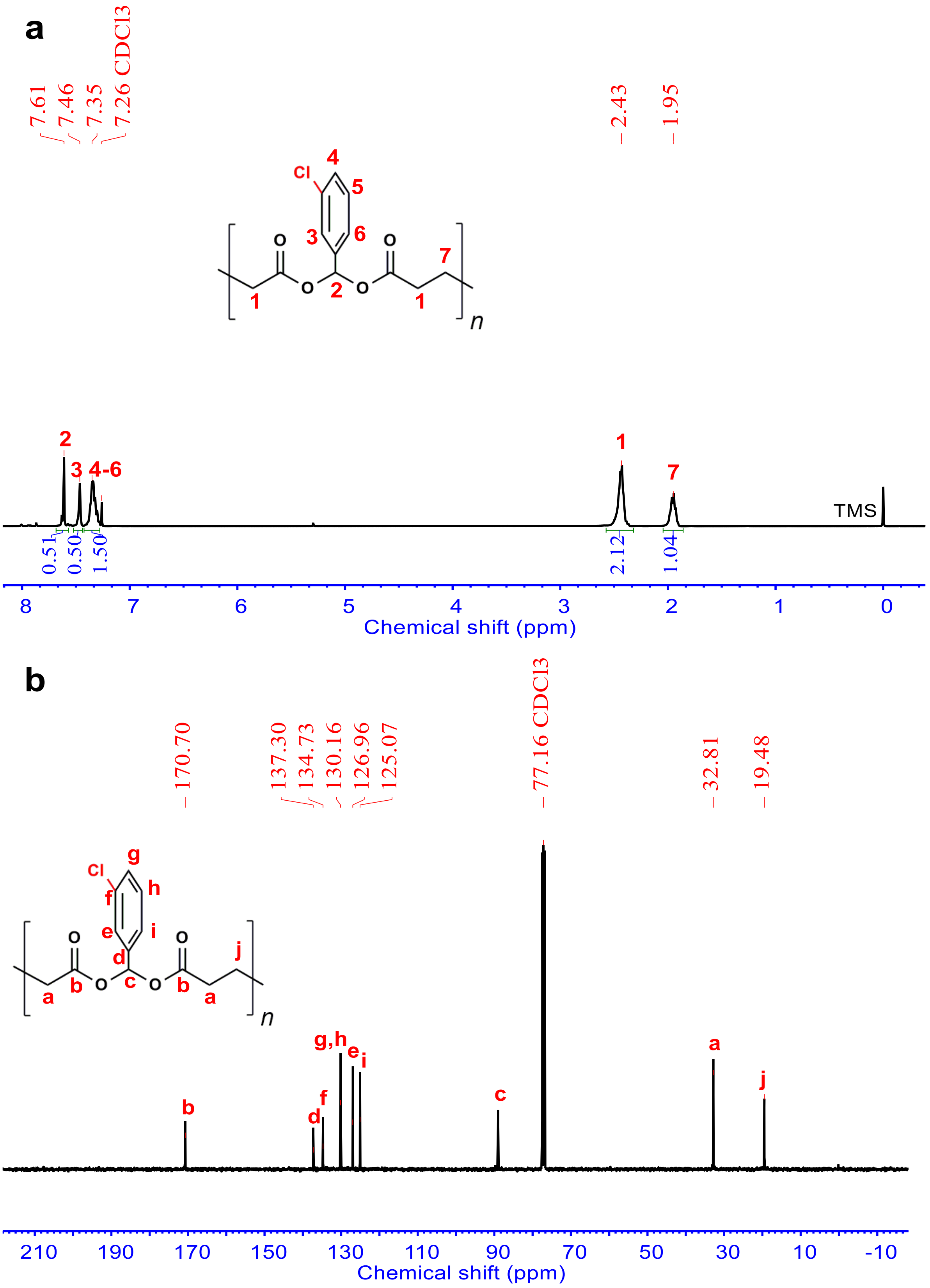

Supplementary Fig. 35 (a) ^{1}H and (b) ^{13}C NMR spectra of the obtained copolymer of **P34A** in $CDCl_3$.

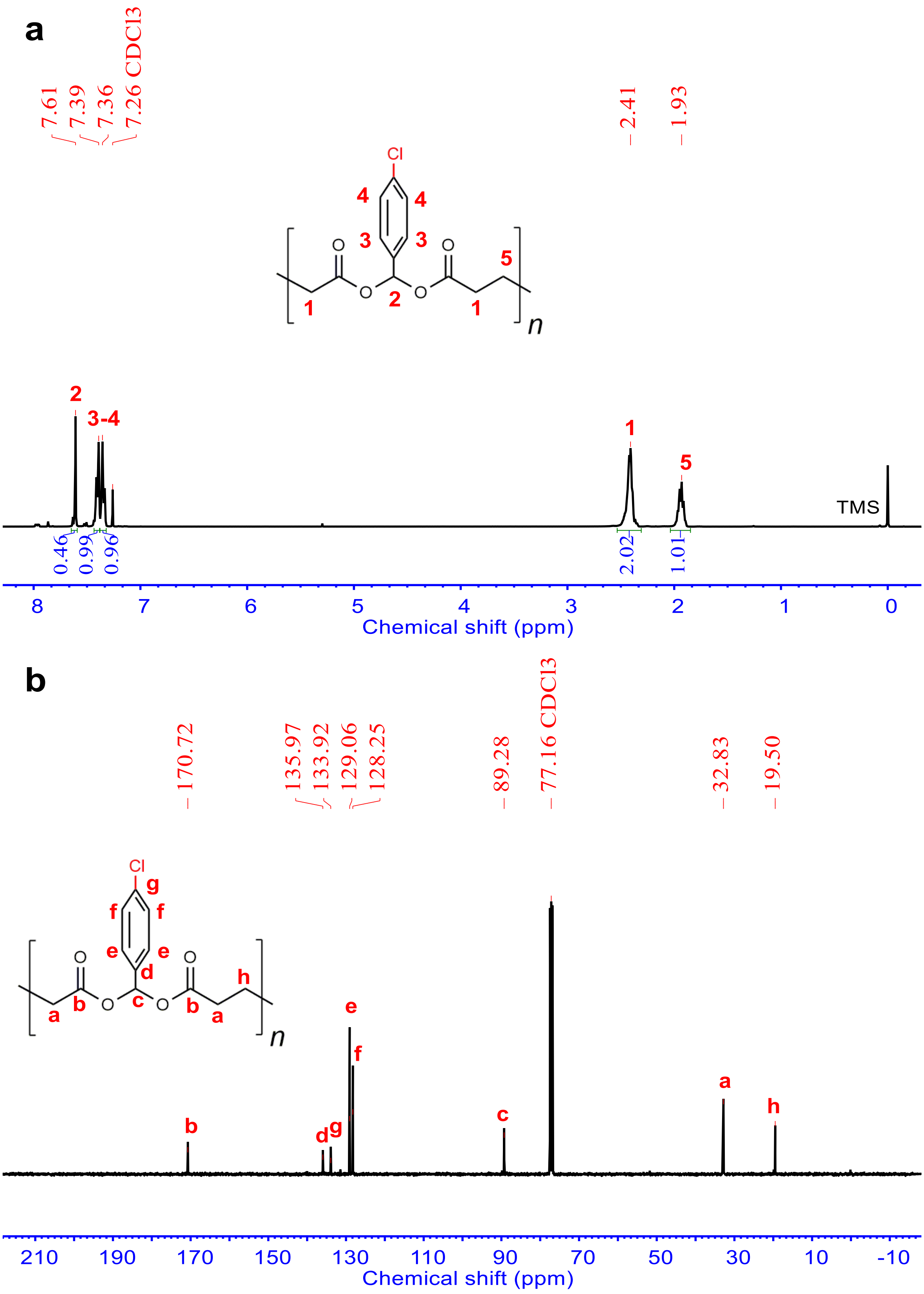

Supplementary Fig. 36 (a) ^{1}H and (b) ^{13}C NMR spectra of the obtained copolymer of **P35A** in $CDCl_3$.

a

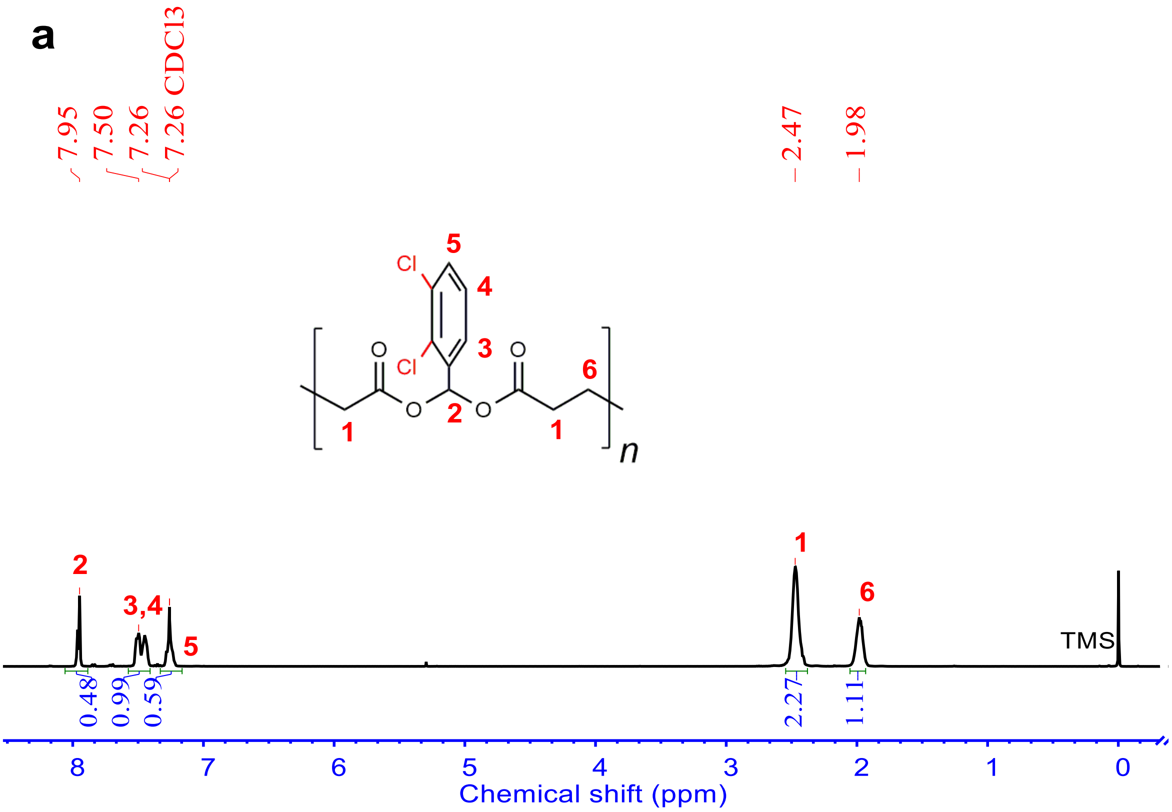

b

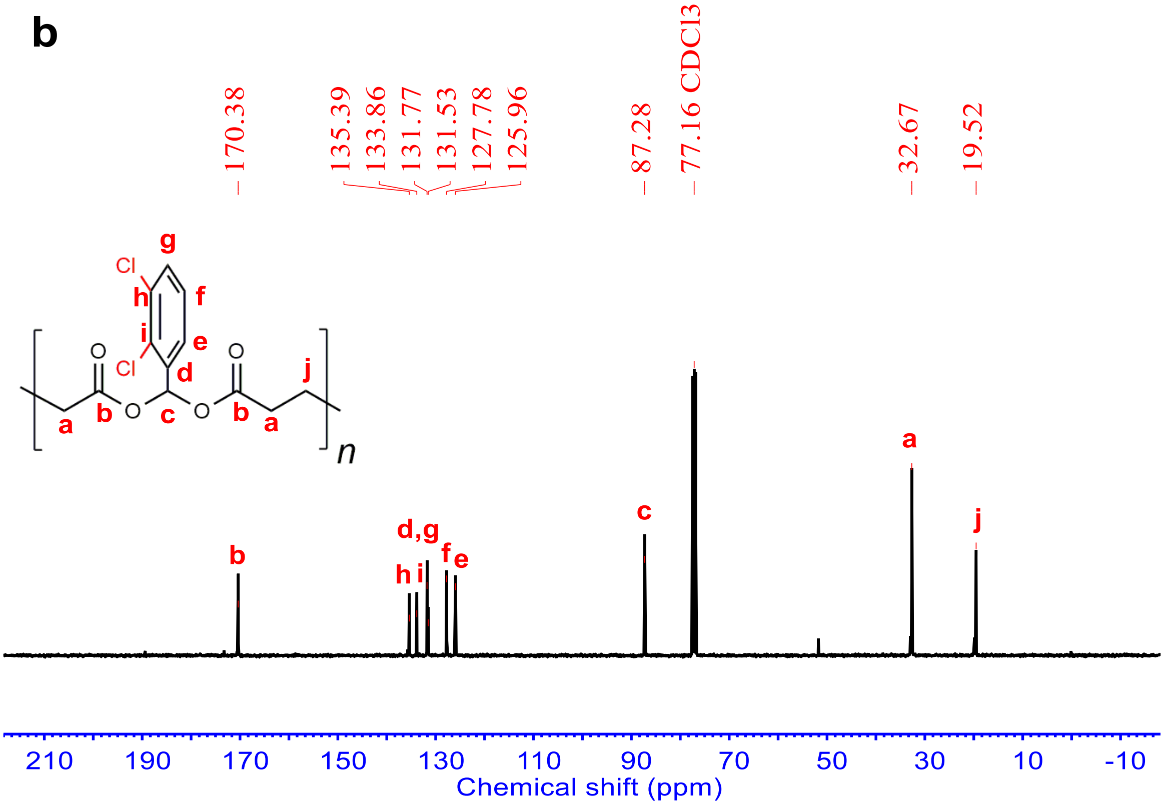

Supplementary Fig. 37 (a) ^{1}H and (b) ^{13}C NMR spectra of the obtained copolymer of **P36A** in $CDCl_3$.

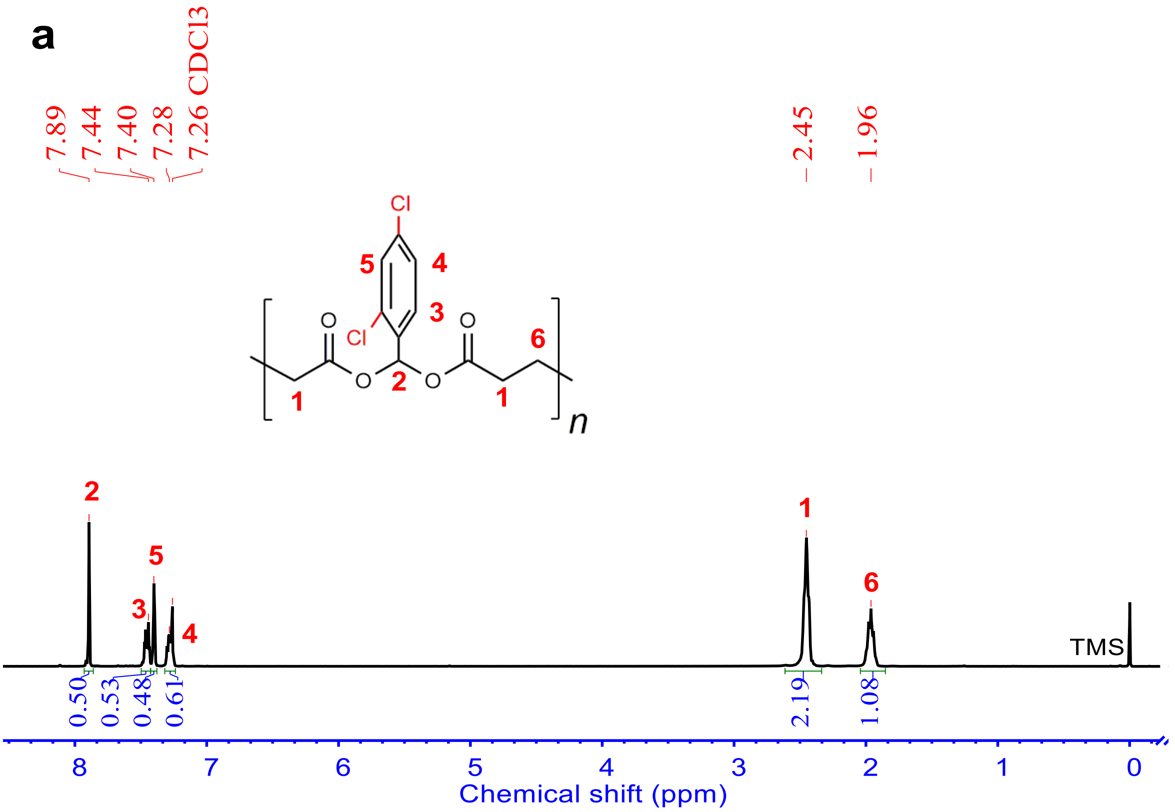

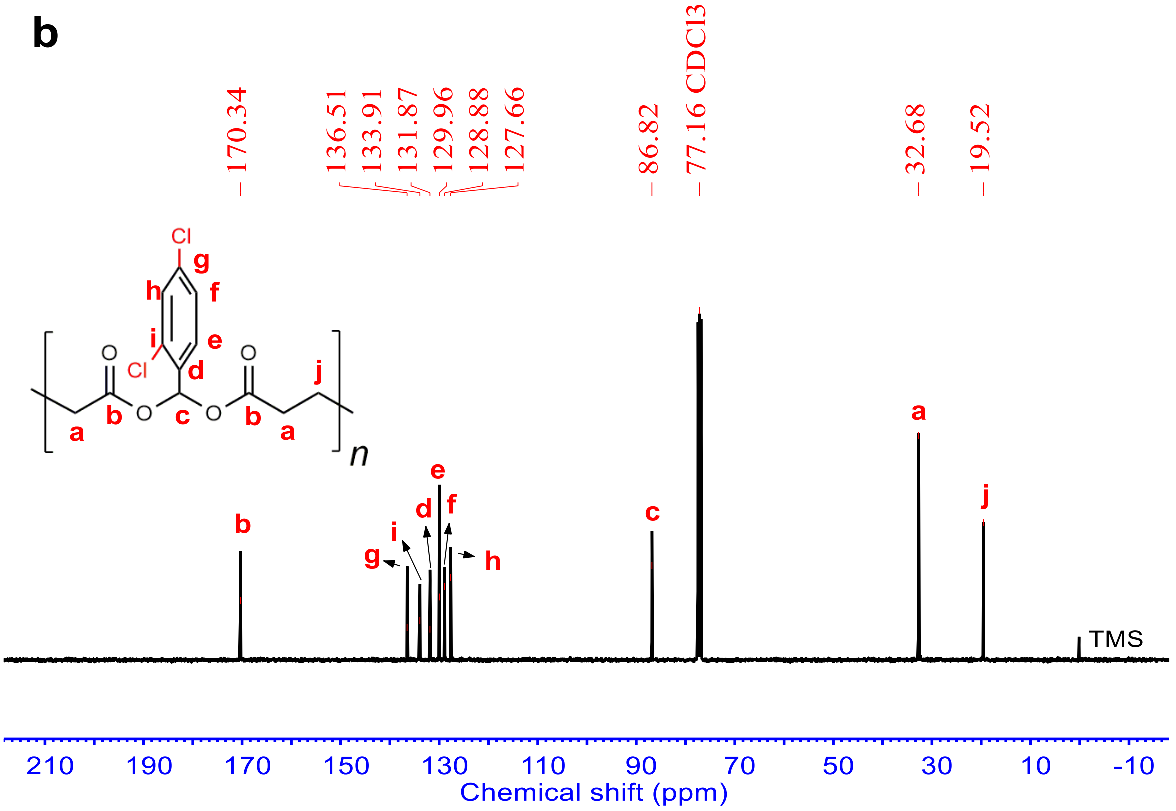

Supplementary Fig. 38 (a) ^{1}H and (b) ^{13}C NMR spectra of the obtained copolymer of **P37A** in $CDCl_3$.

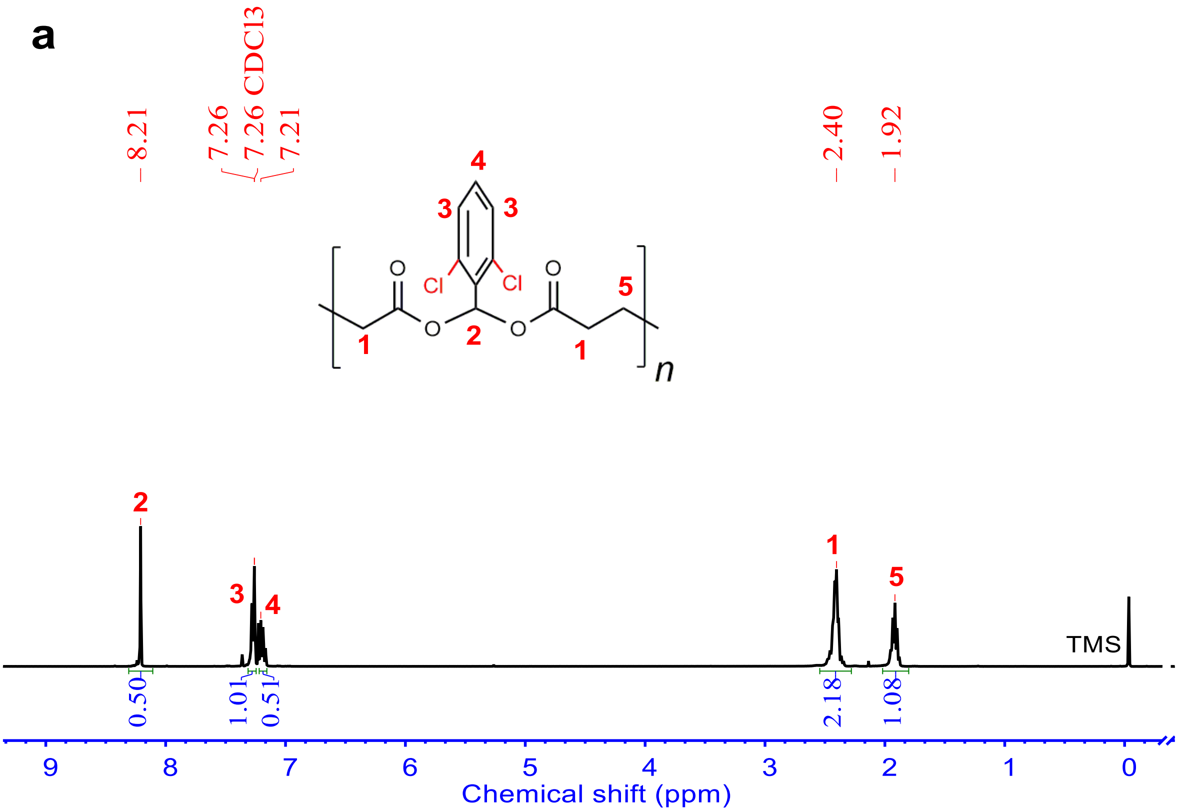

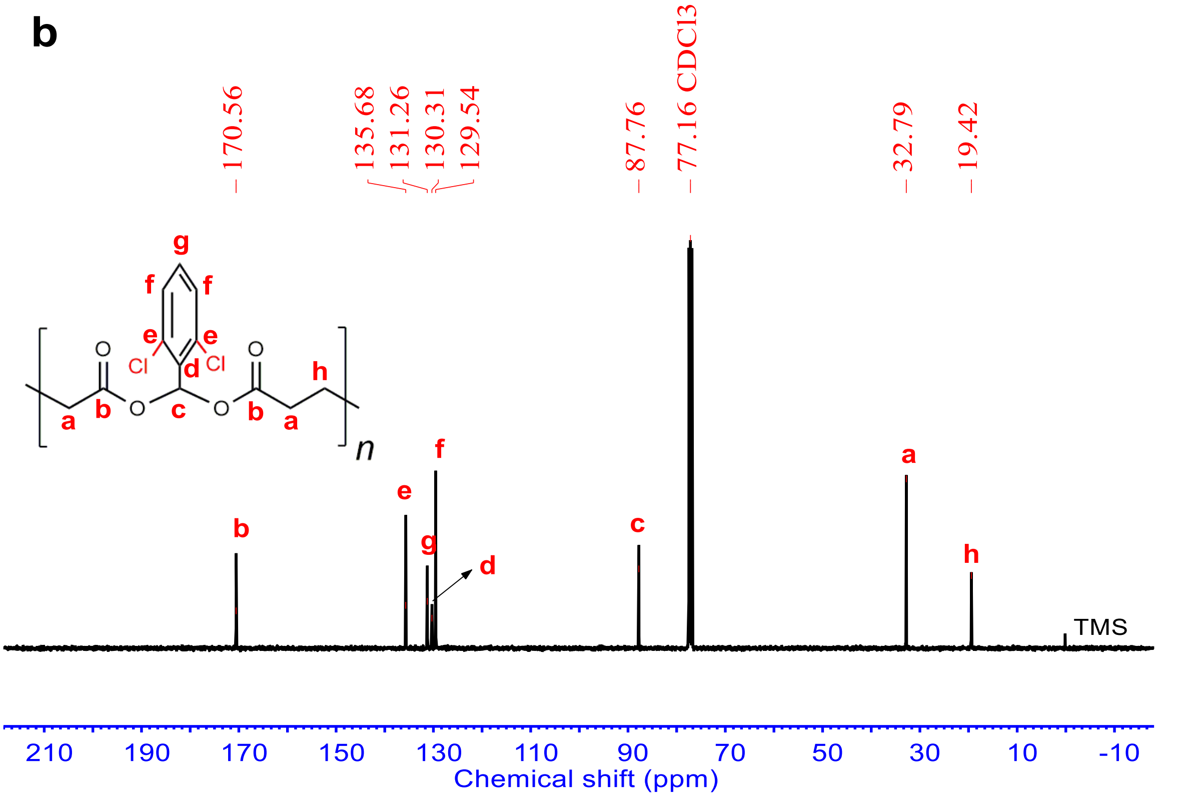

Supplementary Fig. 39 (a) ^{1}H and (b) ^{13}C NMR spectra of the obtained copolymer of **P38A** in $CDCl_3$.

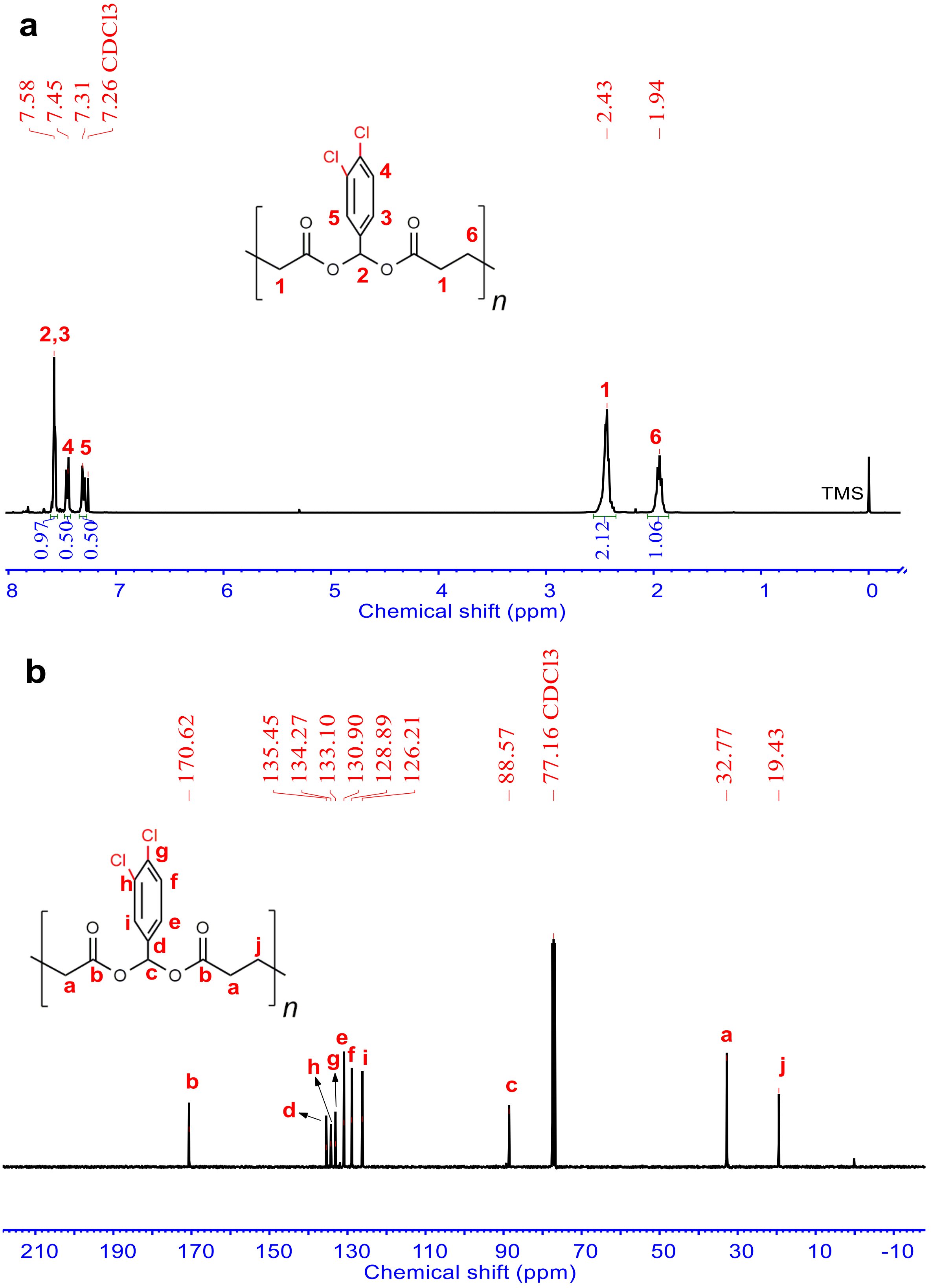

Supplementary Fig. 40 (a) ^{1}H and (b) ^{13}C NMR spectra of the obtained copolymer of **P39A** in $CDCl_3$.

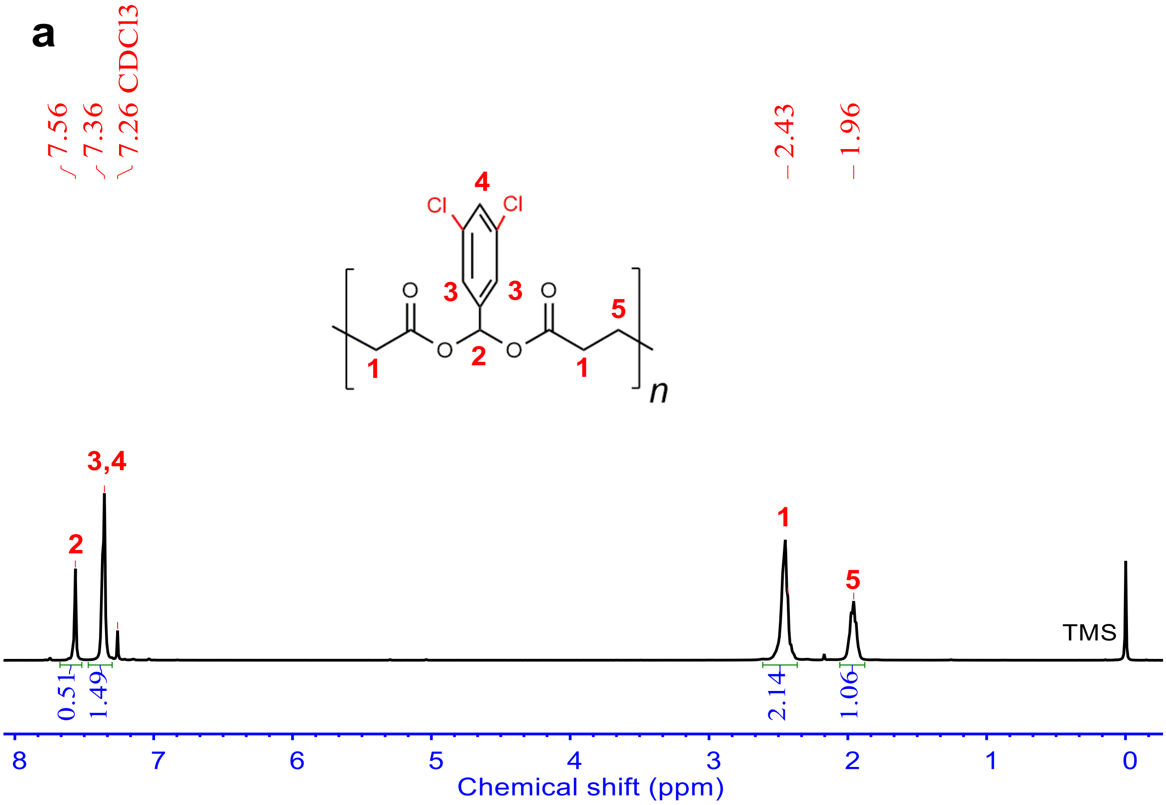

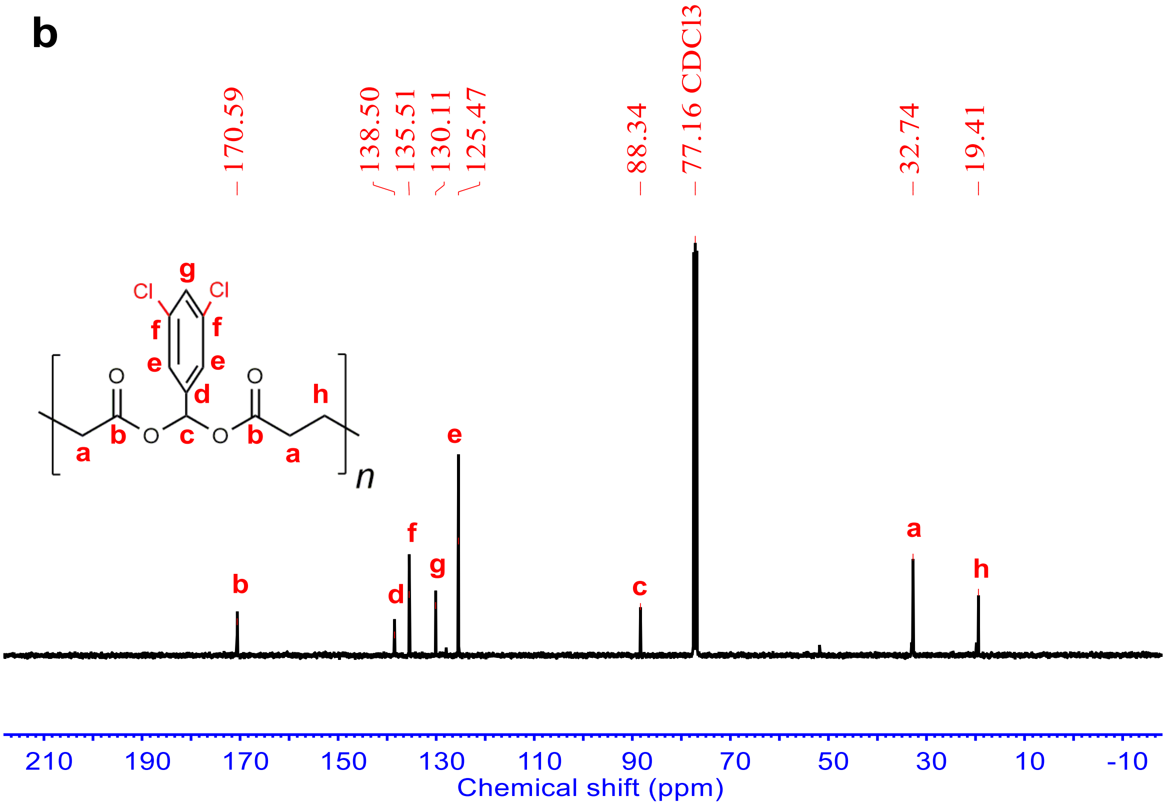

Supplementary Fig. 41 (a) ^{1}H and (b) ^{13}C NMR spectra of the obtained copolymer of **P40A** in $CDCl_3$.

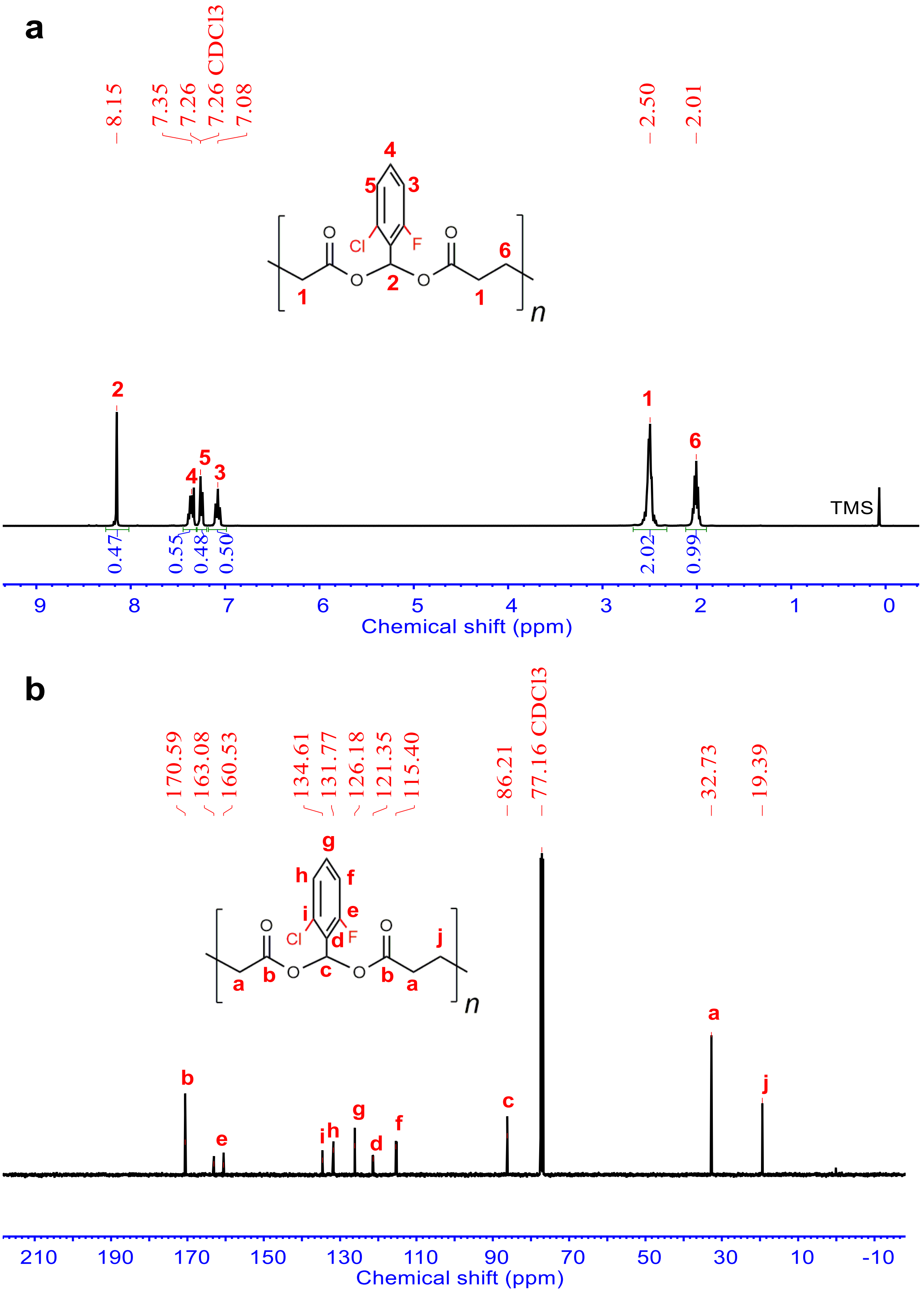

Supplementary Fig. 42 (a) ^{1}H and (b) ^{13}C NMR spectra of the obtained copolymer of **P41A** in $CDCl_3$.

a

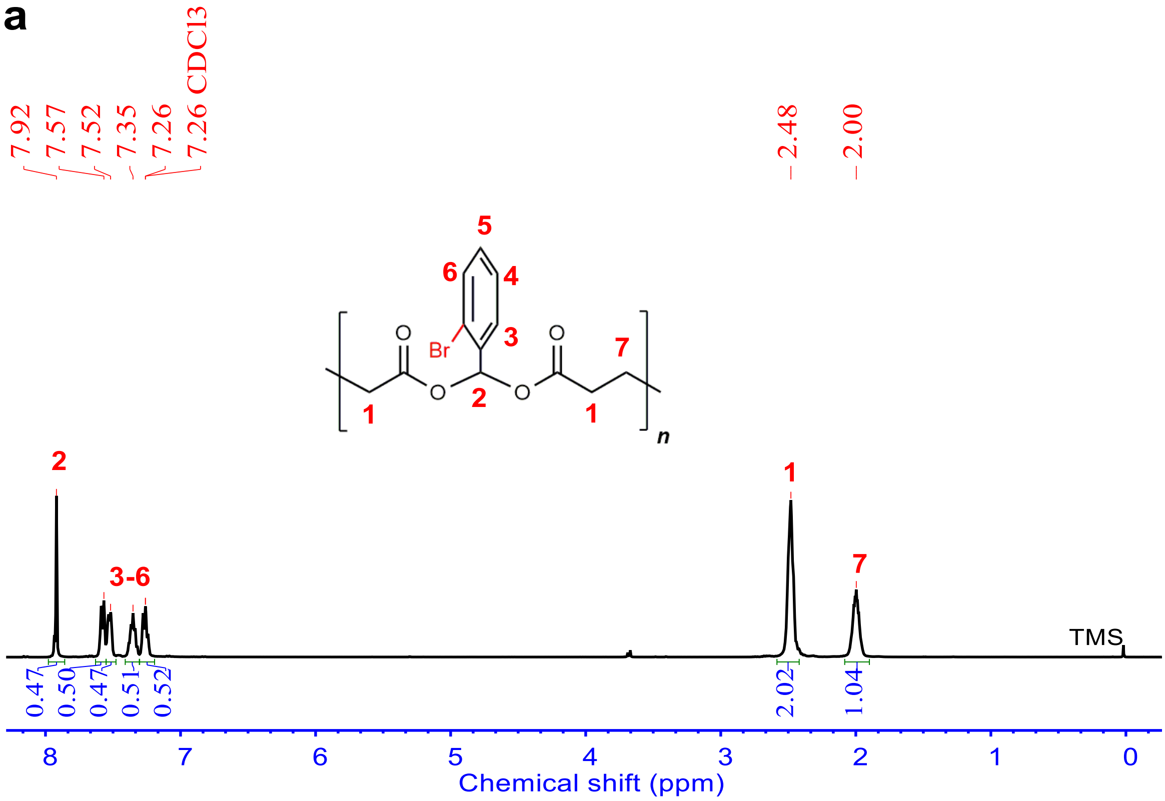

b

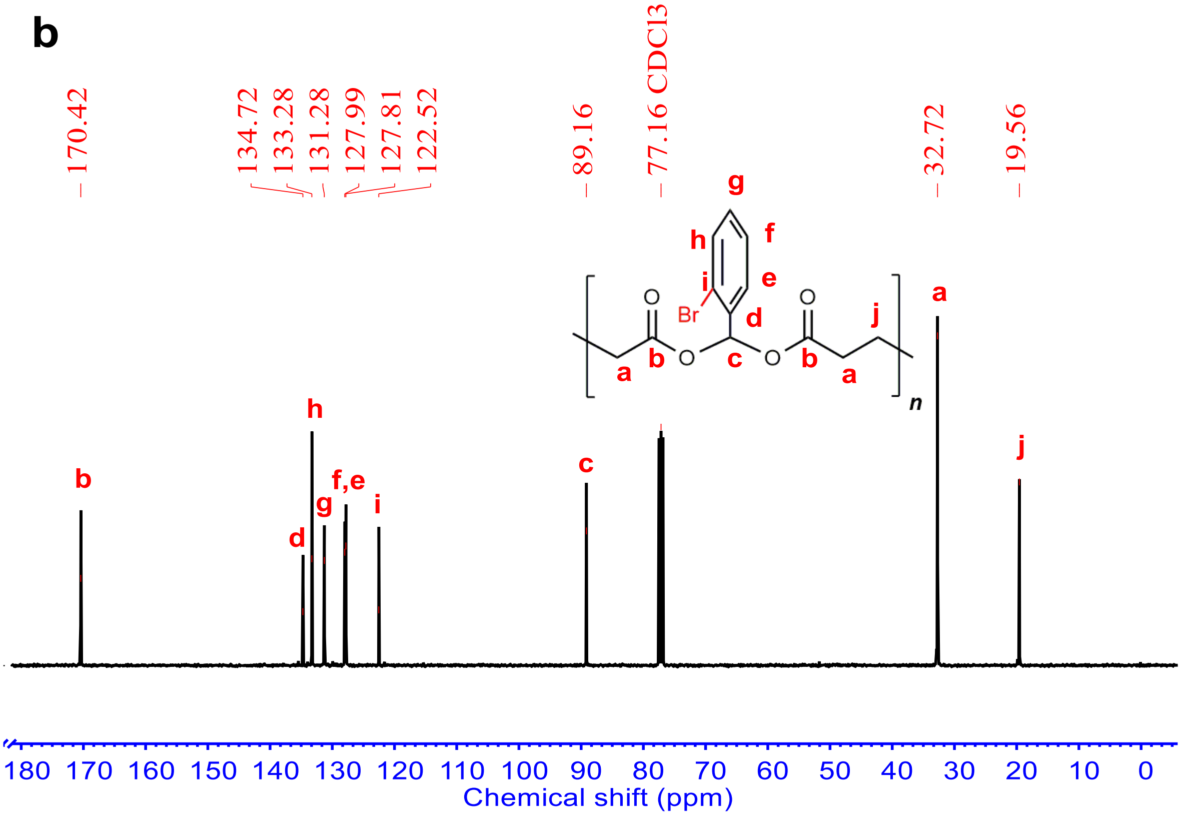

Supplementary Fig. 43 (a) ^{1}H and (b) ^{13}C NMR spectra of the obtained copolymer of **P42A** in $CDCl_3$.

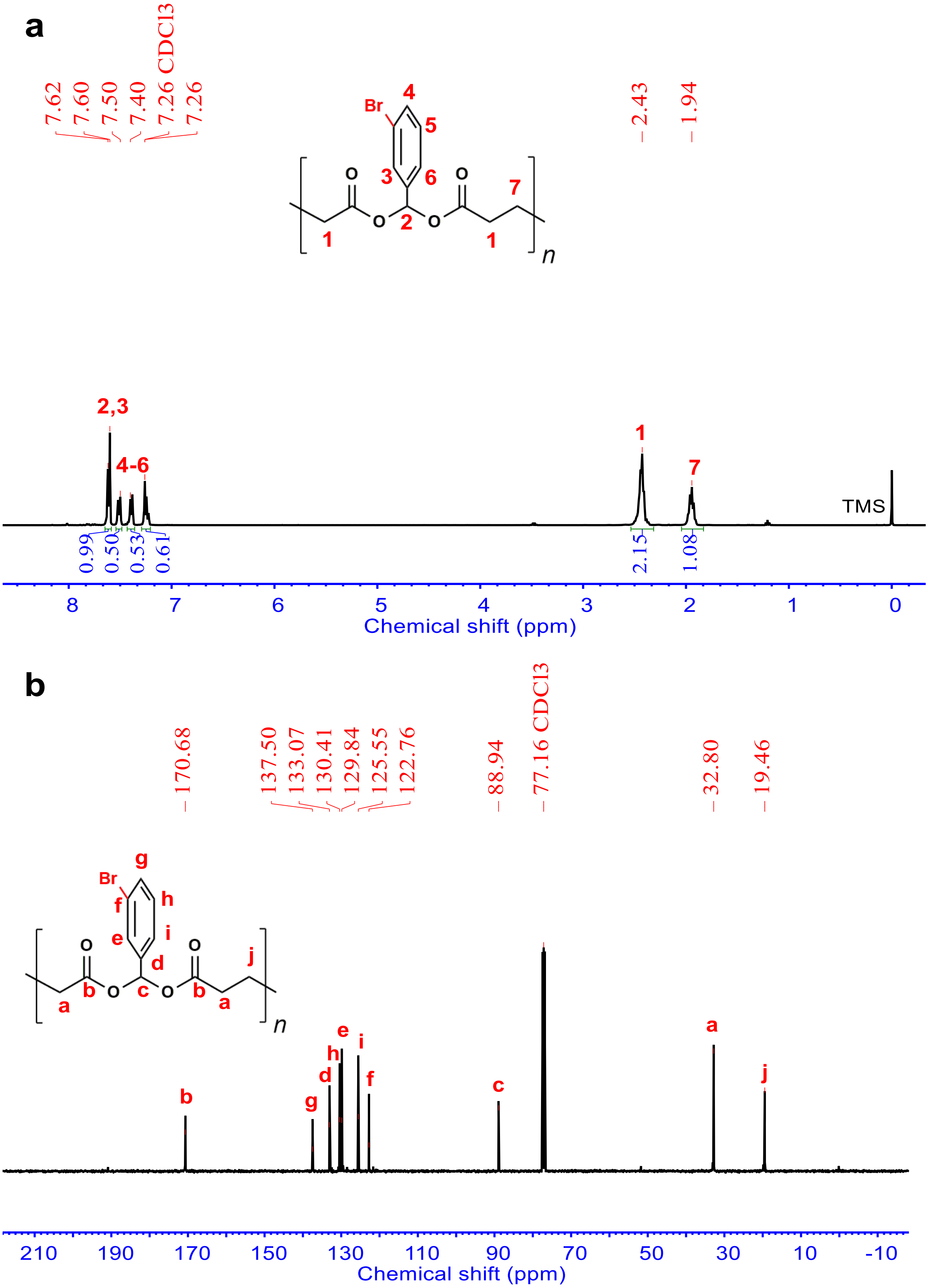

Supplementary Fig. 44 (a) ^{1}H and (b) ^{13}C NMR spectra of the obtained copolymer of **P43A** in $CDCl_3$.

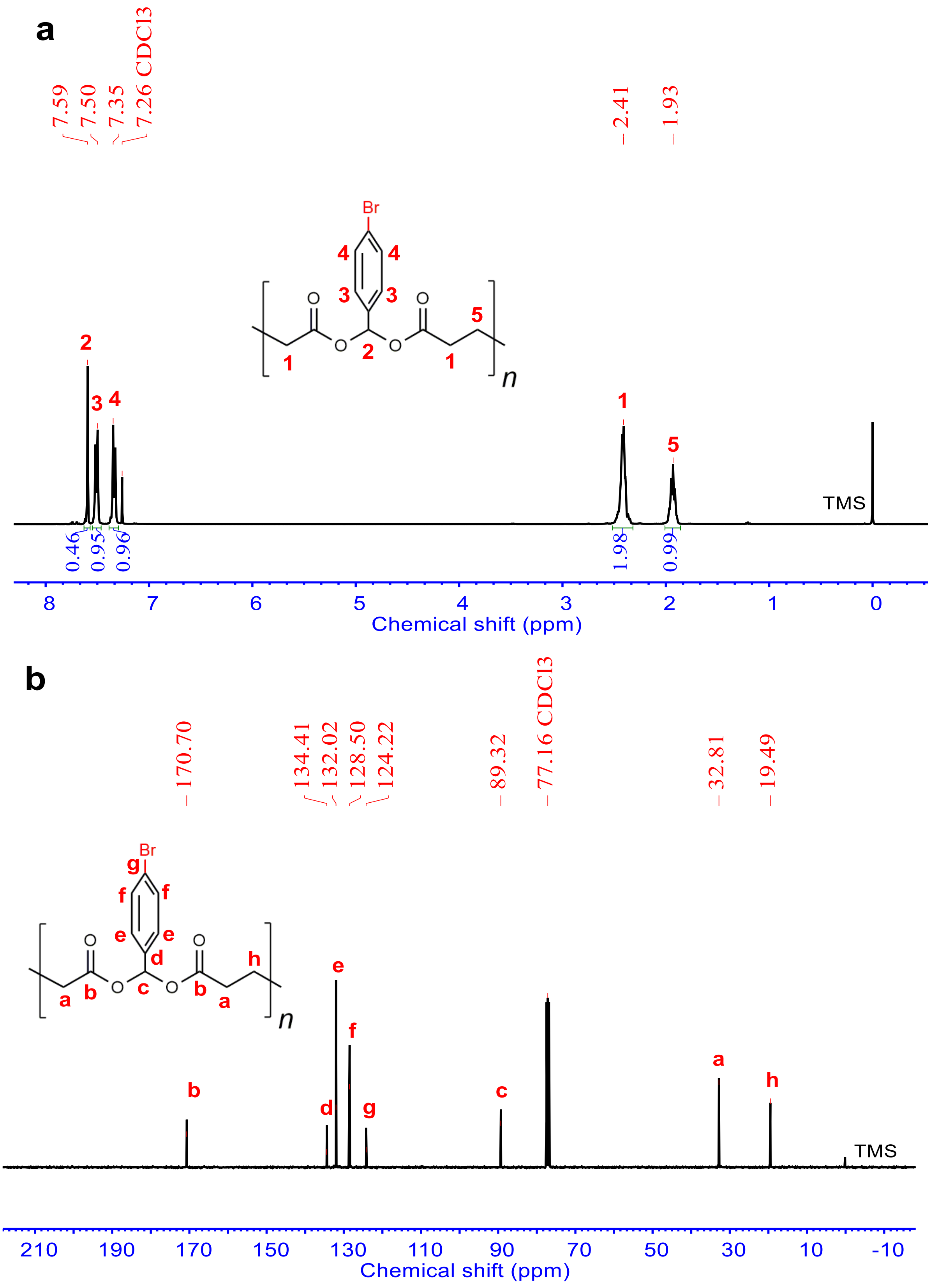

Supplementary Fig. 45 (a) ^{1}H and (b) ^{13}C NMR spectra of the obtained copolymer of **P44A** in $CDCl_3$.

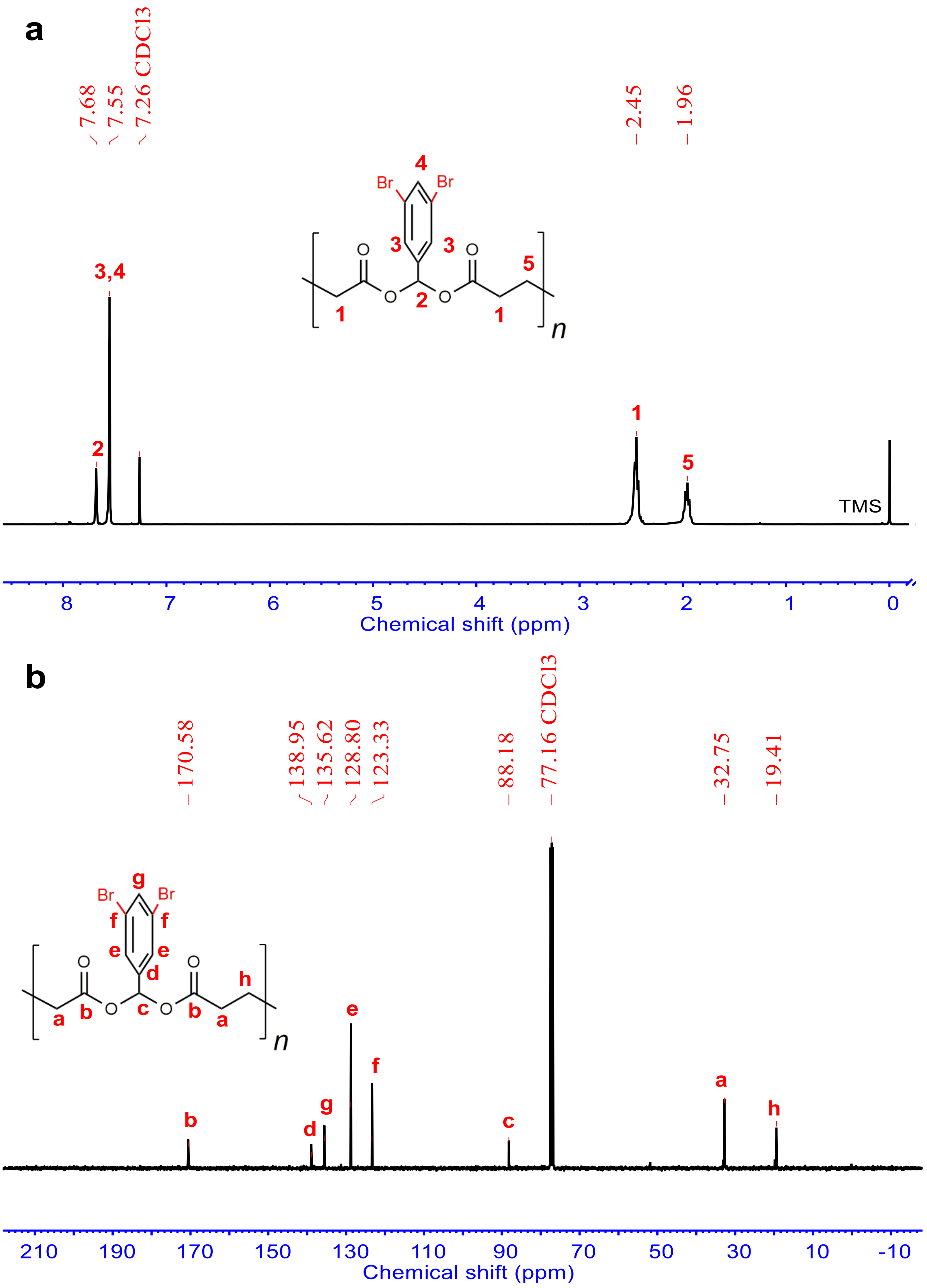

Supplementary Fig. 46 (a) ^{1}H and (b) ^{13}C NMR spectra of the obtained copolymer of **P45A** in $CDCl_3$.

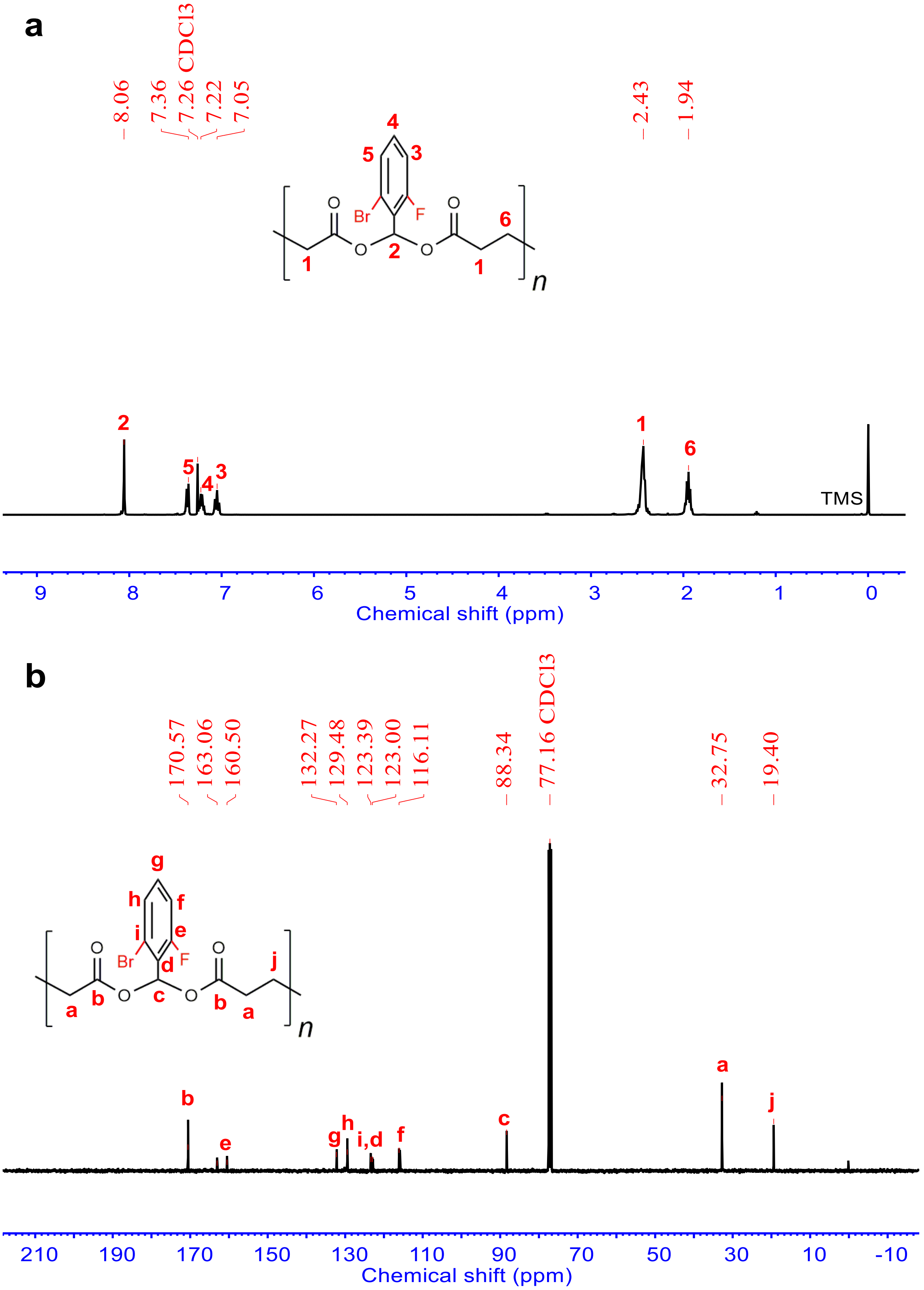

Supplementary Fig. 47 (a) ^{1}H and (b) ^{13}C NMR spectra of the obtained copolymer of **P46A** in $CDCl_3$.

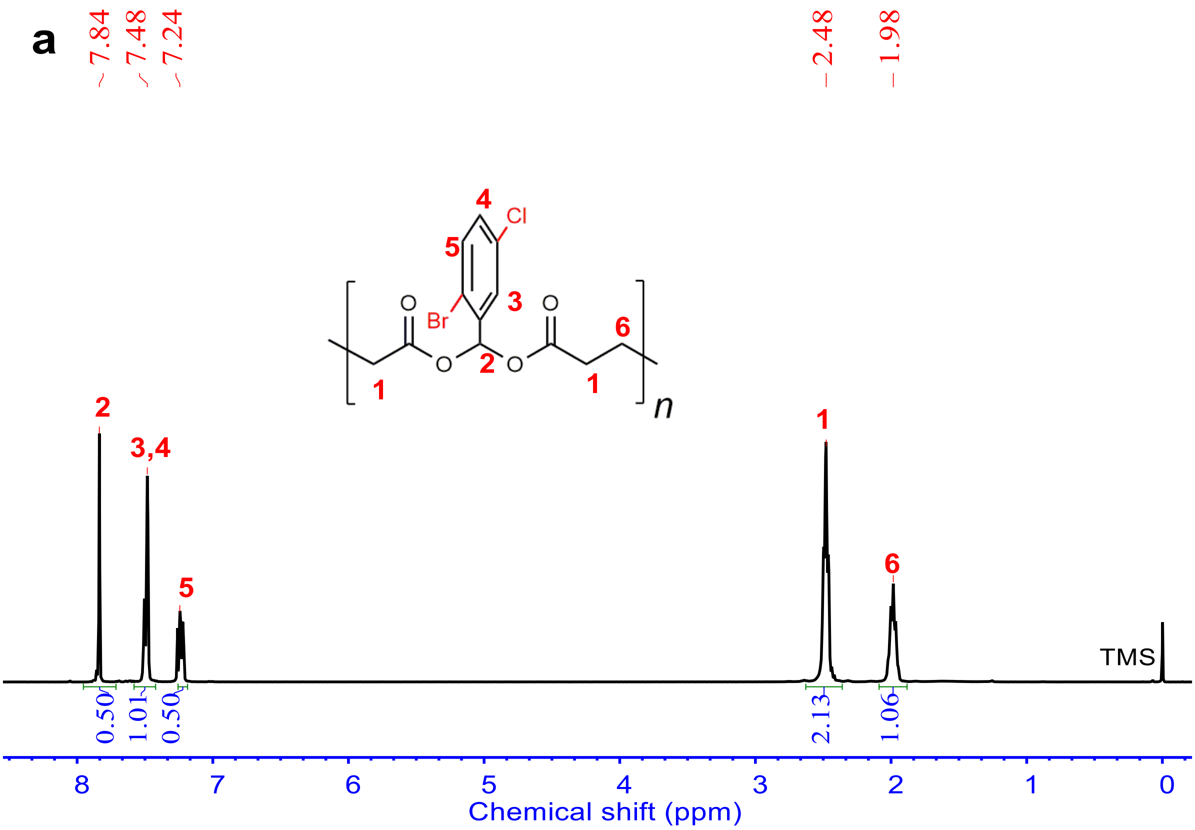

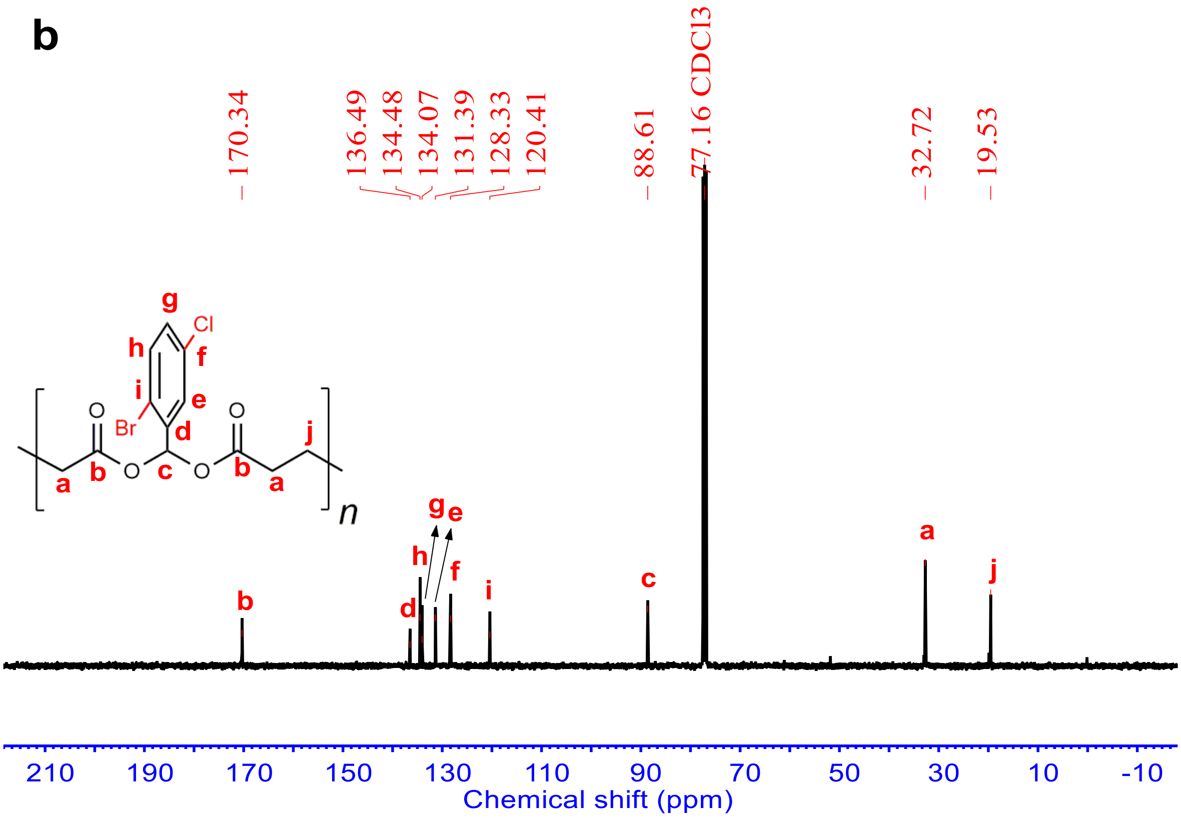

Supplementary Fig. 48 (a) ^{1}H and (b) ^{13}C NMR spectra of the obtained copolymer of **P47A** in $CDCl_3$.

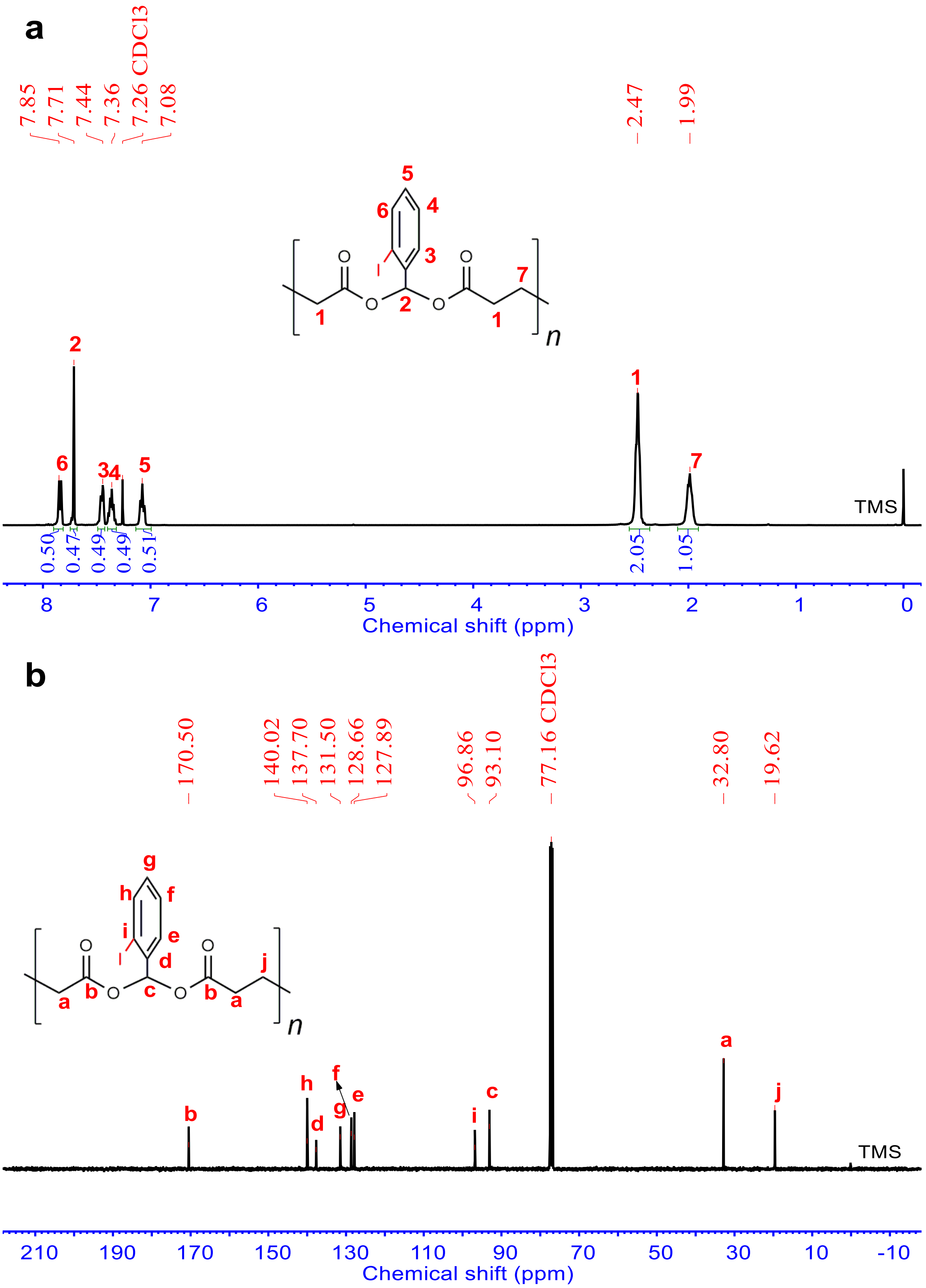

Supplementary Fig. 49 (a) ^{1}H and (b) ^{13}C NMR spectra of the obtained copolymer of **P48A** in $CDCl_3$.

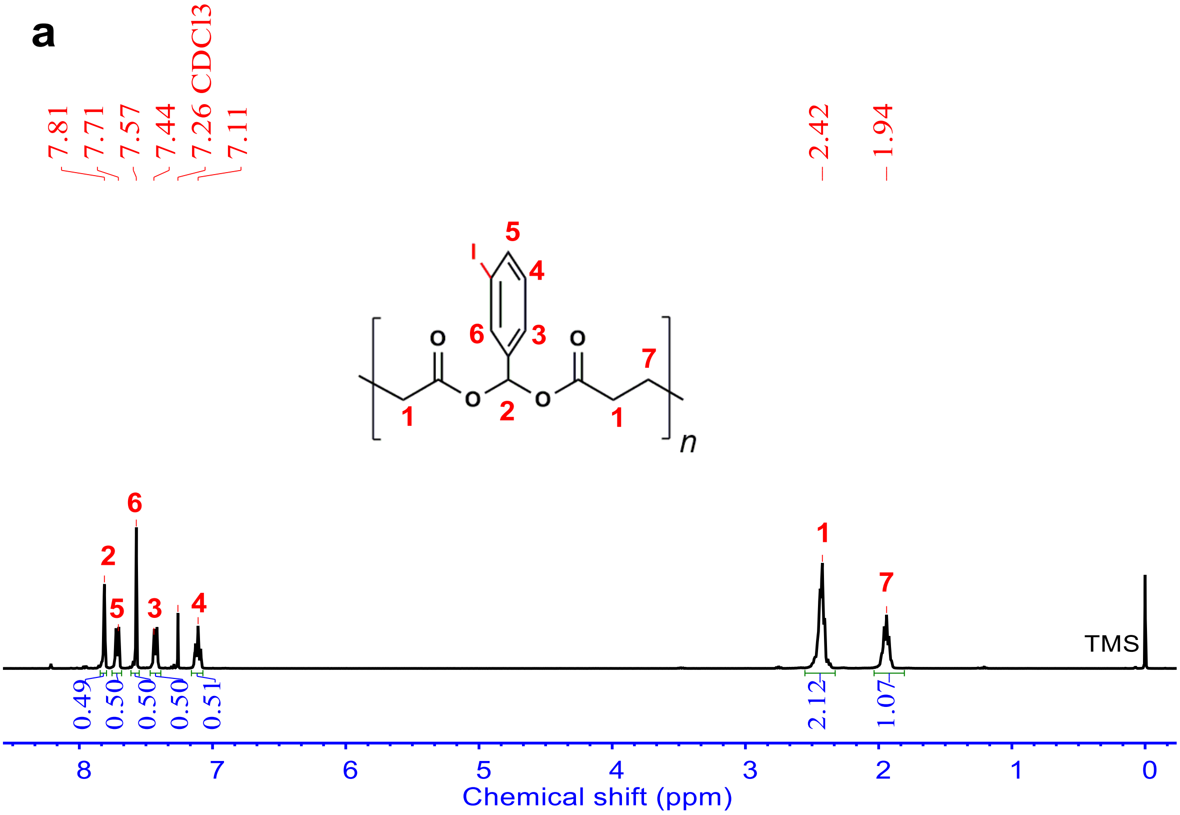

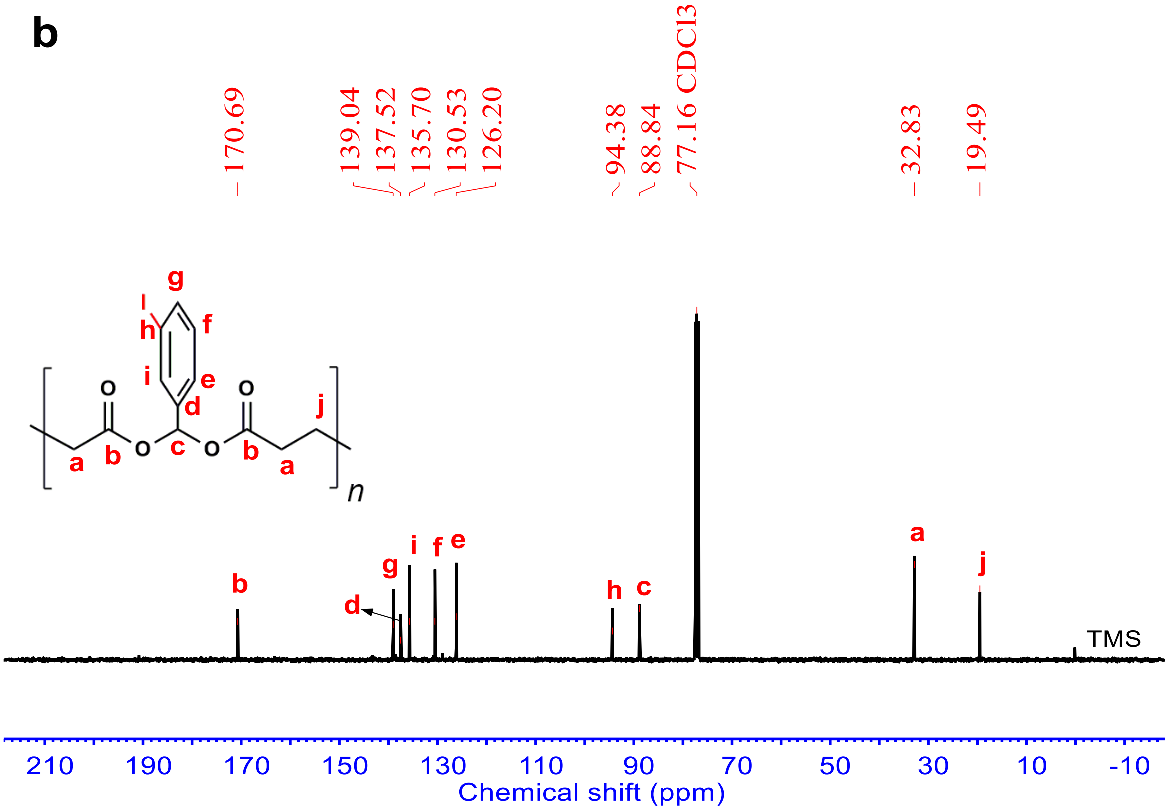

Supplementary Fig. 50 (a) ^{1}H and (b) ^{13}C NMR spectra of the obtained copolymer of **P49A** in $CDCl_3$.

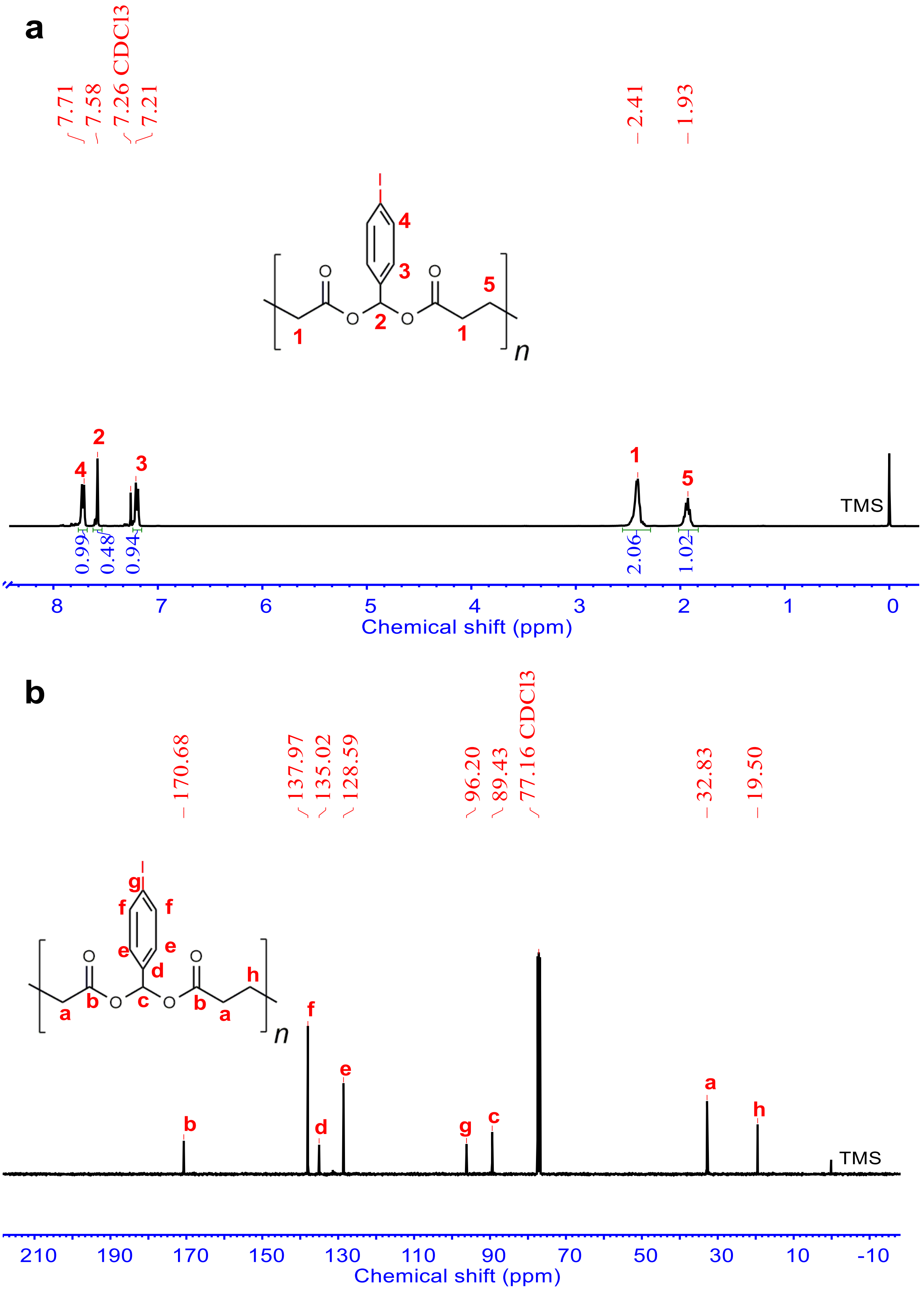

Supplementary Fig. 51 (a) ^{1}H and (b) ^{13}C NMR spectra of the obtained copolymer of **P50A** in $CDCl_3$.

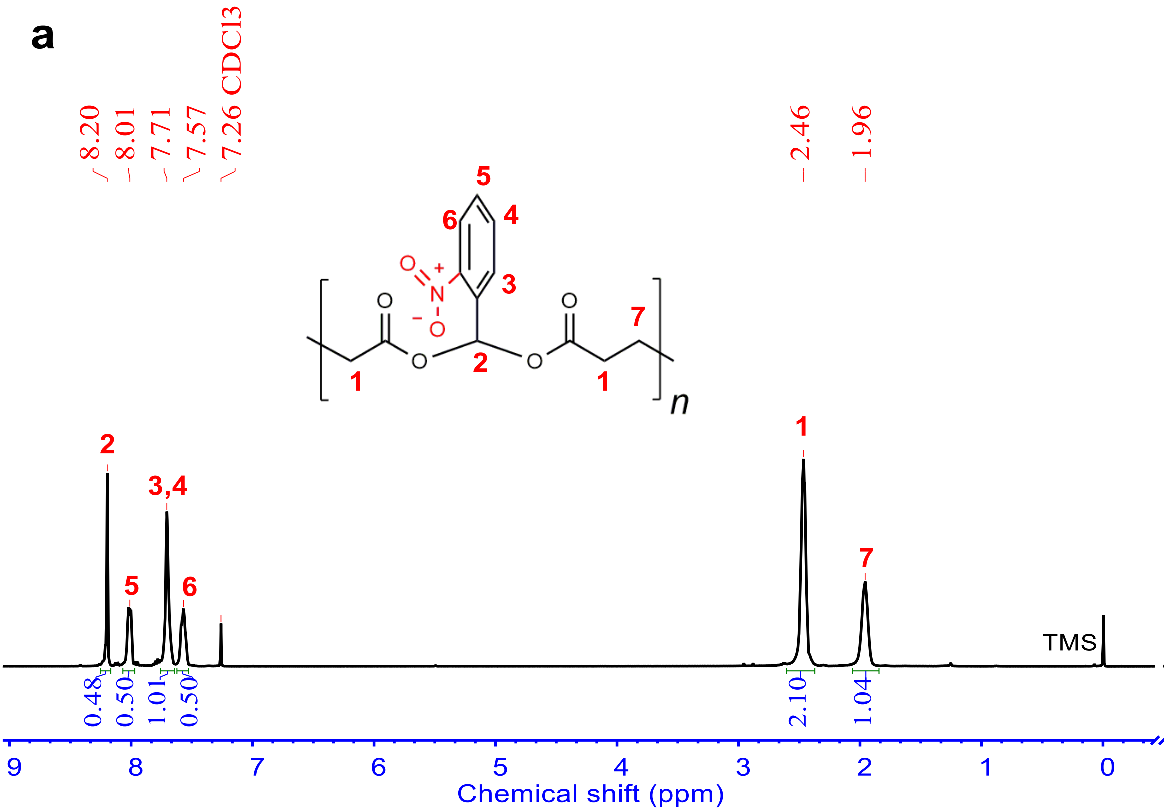

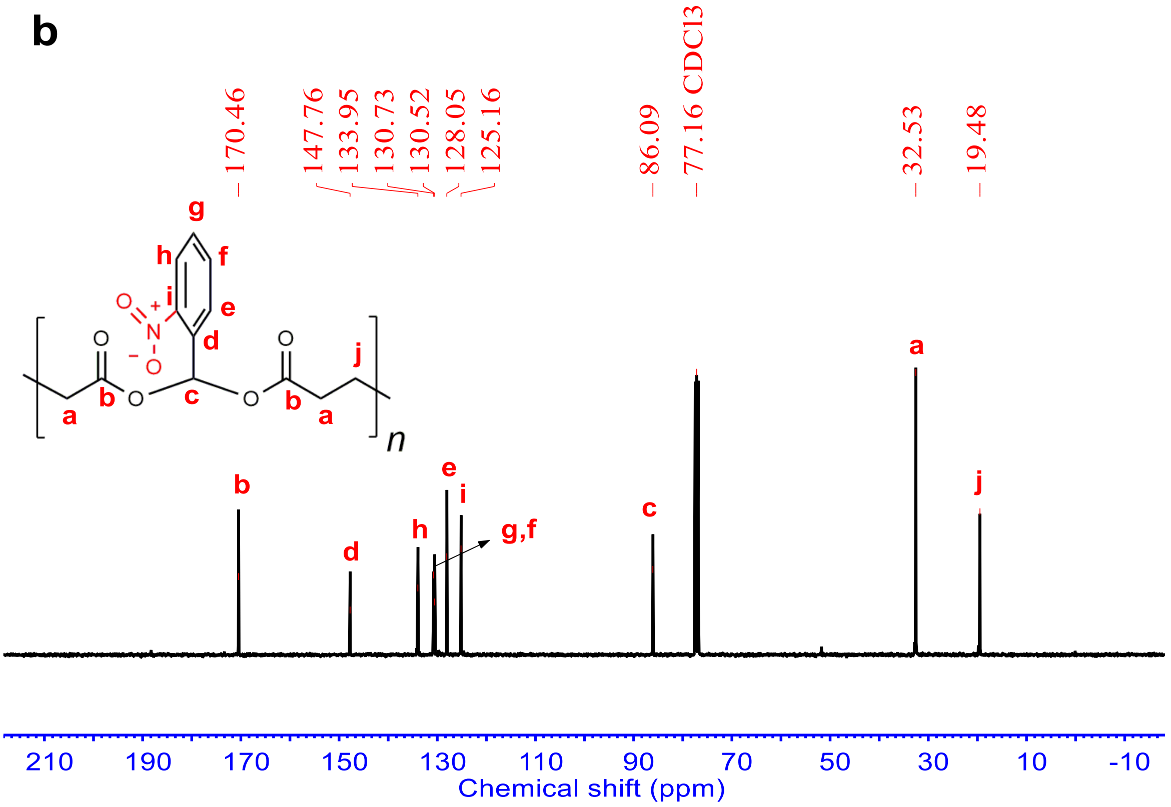

Supplementary Fig. 52 (a) ^{1}H and (b) ^{13}C NMR spectra of the obtained copolymer of **P51A** in $CDCl_3$.

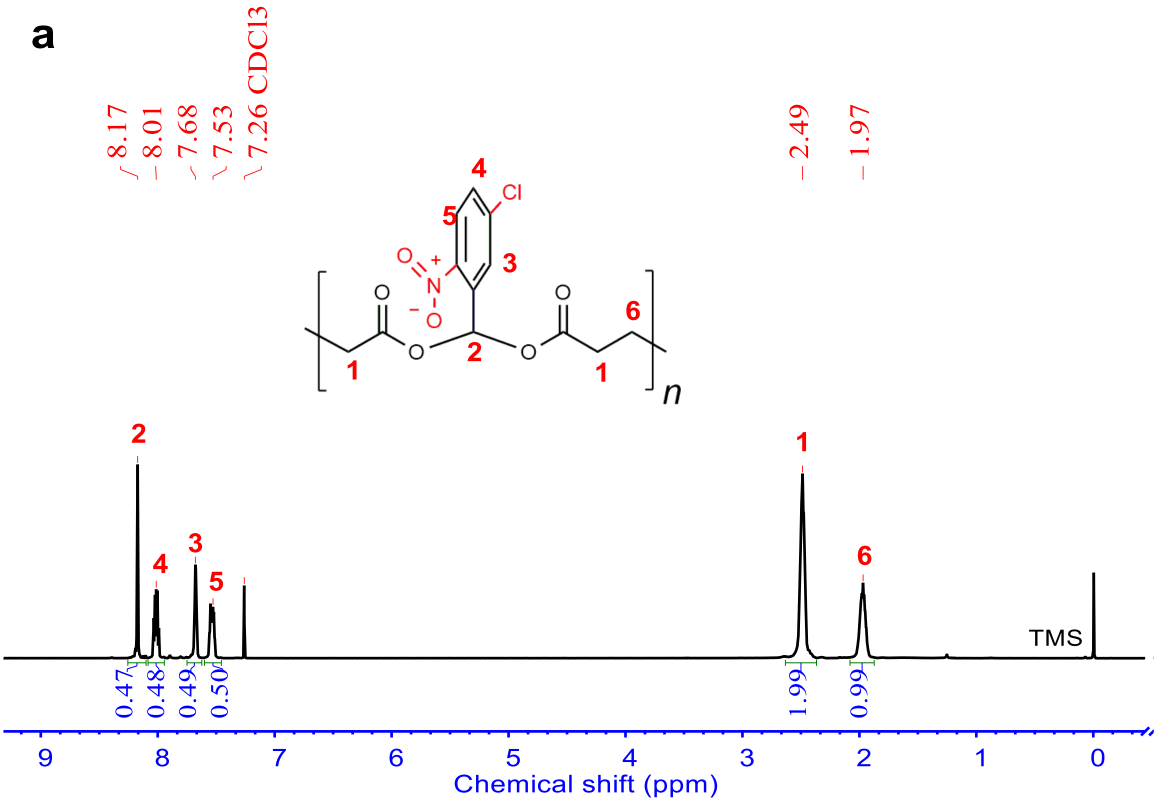

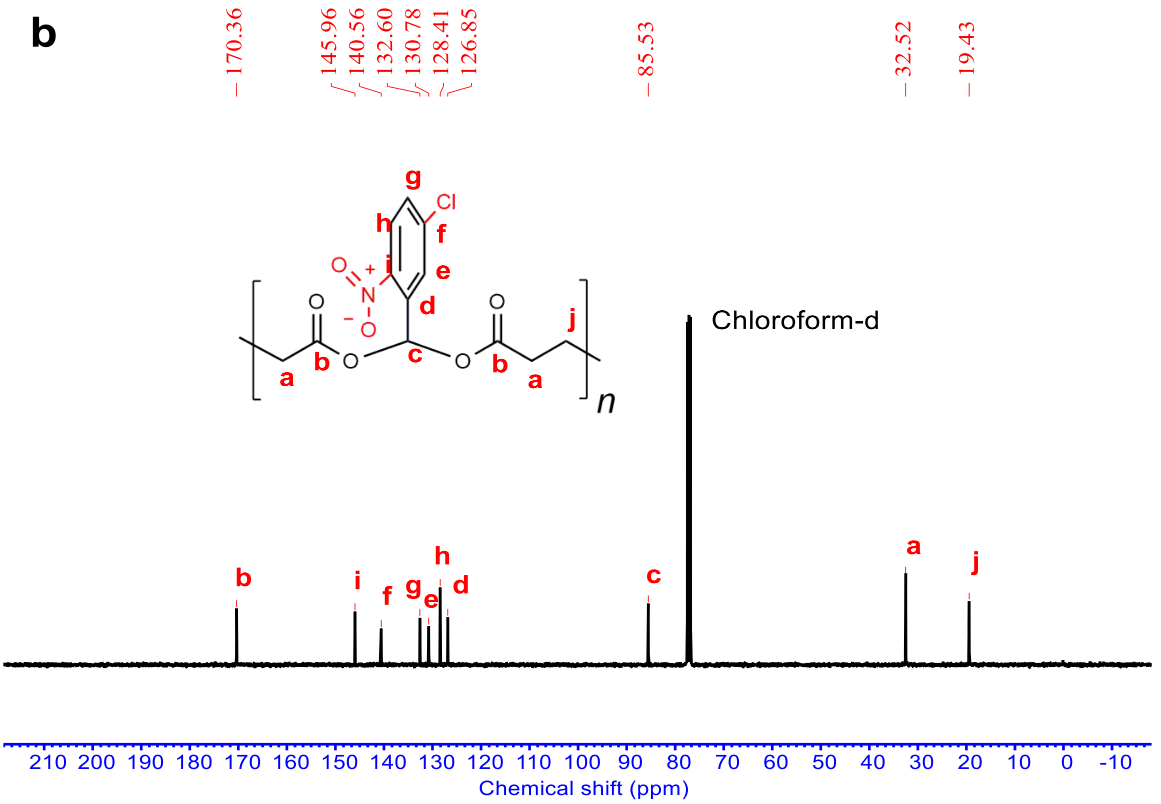

Supplementary Fig. 53 (a) ^{1}H and (b) ^{13}C NMR spectra of the obtained copolymer of **P52A** in $CDCl_3$.

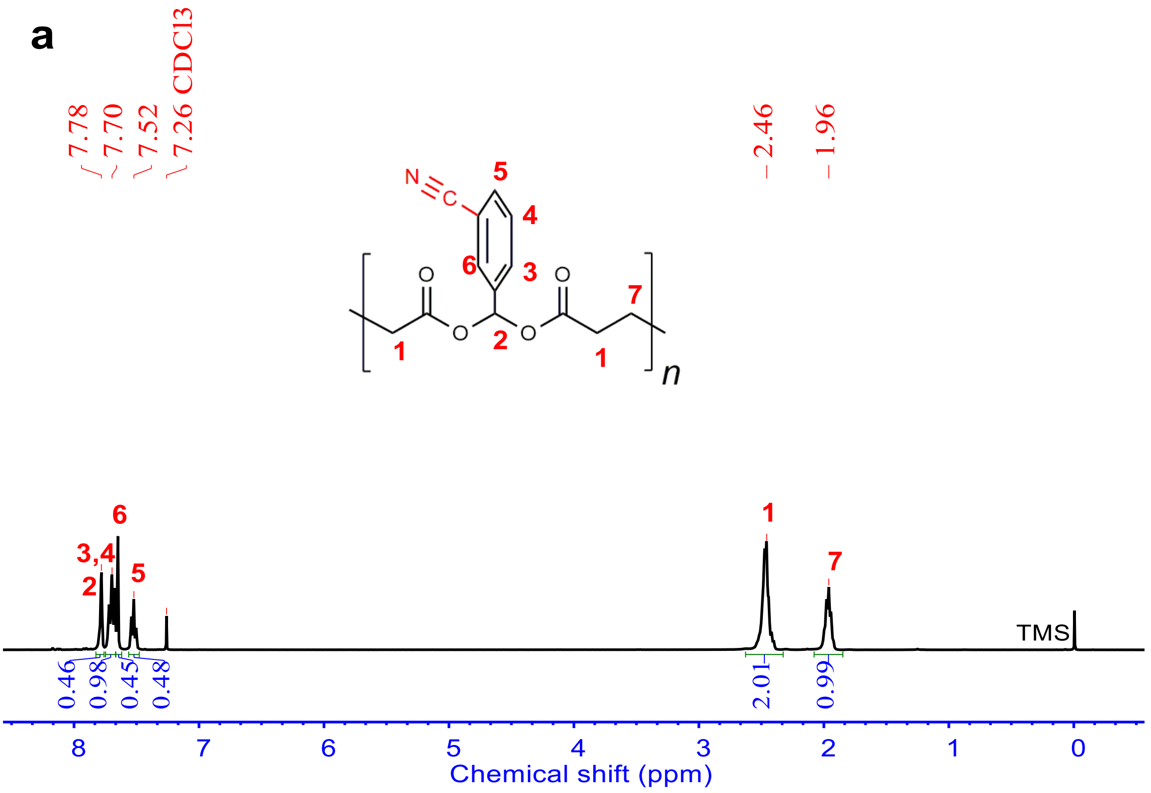

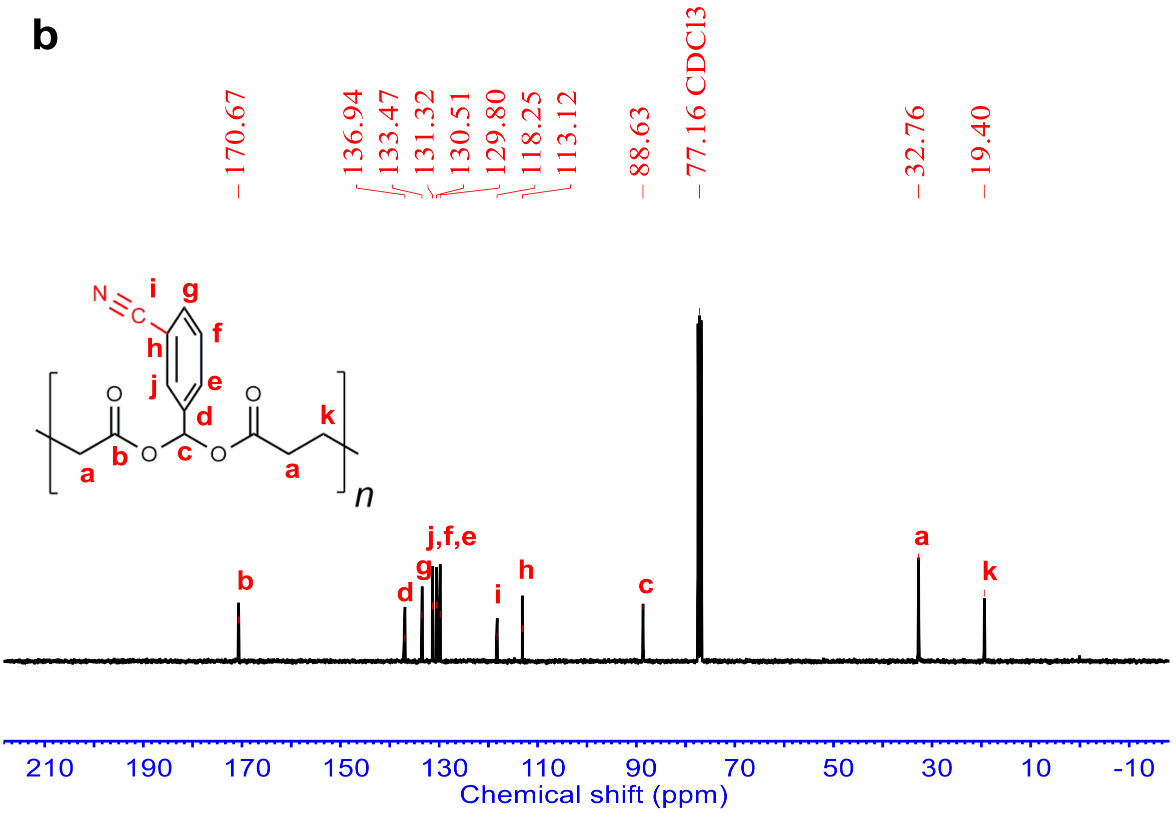

Supplementary Fig. 54 (a) ^{1}H and (b) ^{13}C NMR spectra of the obtained copolymer of **P53A** in $CDCl_3$.

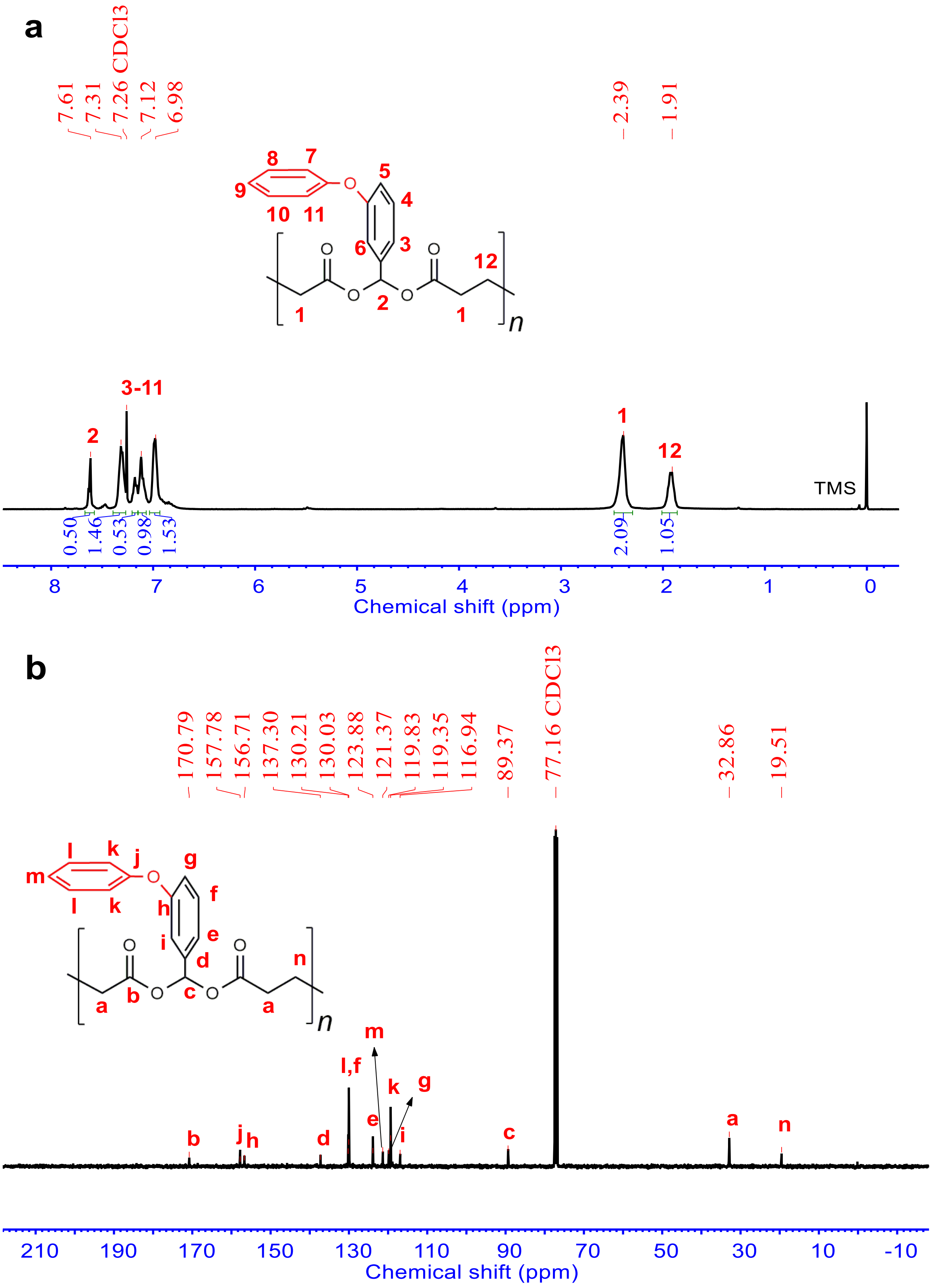

Supplementary Fig. 55 (a) ^{1}H and (b) ^{13}C NMR spectra of the obtained copolymer of **P54A** in $CDCl_3$.

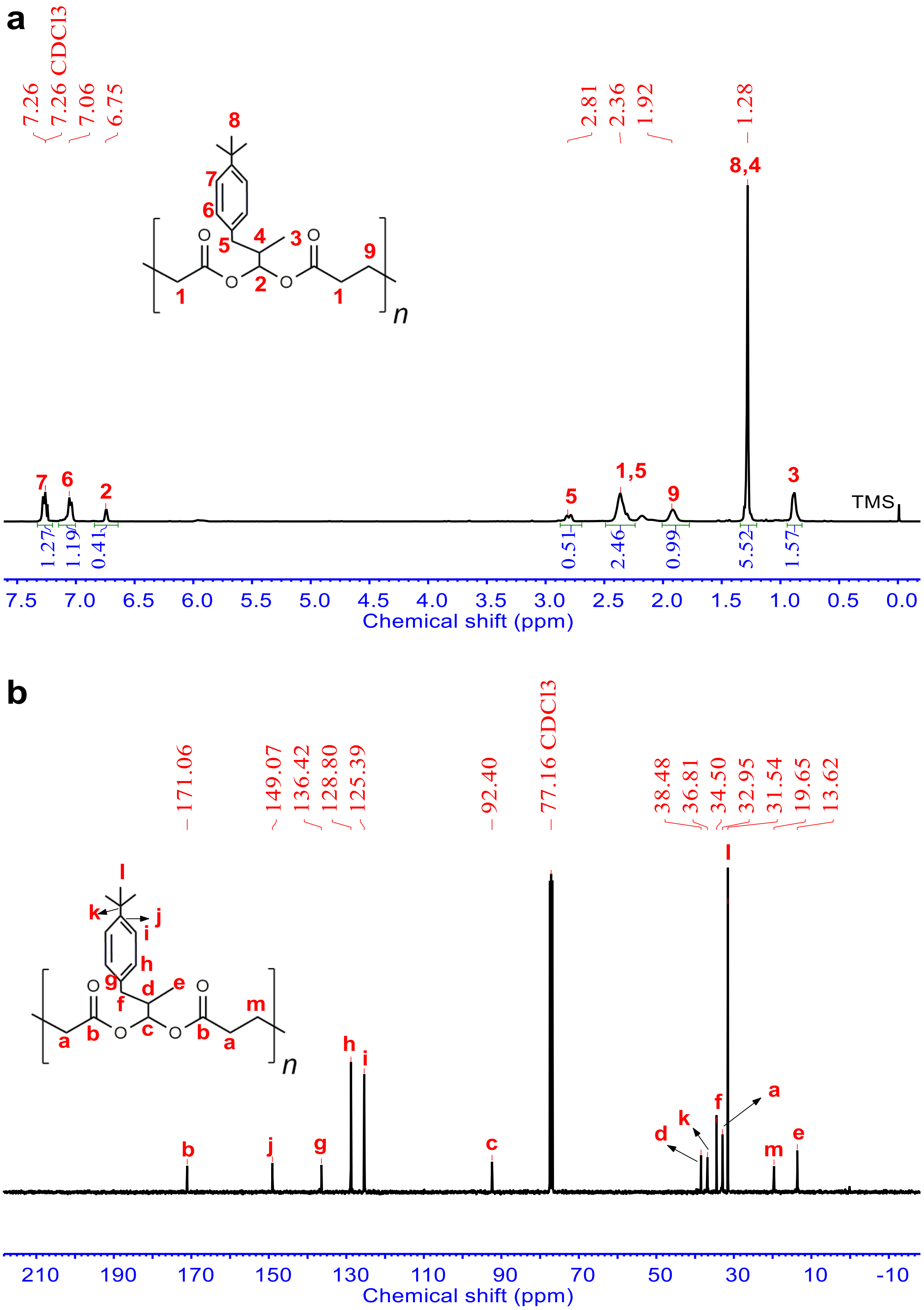

Supplementary Fig. 56 (a) ^{1}H and (b) ^{13}C NMR spectra of the obtained copolymer of **P55A** in $CDCl_3$.

a

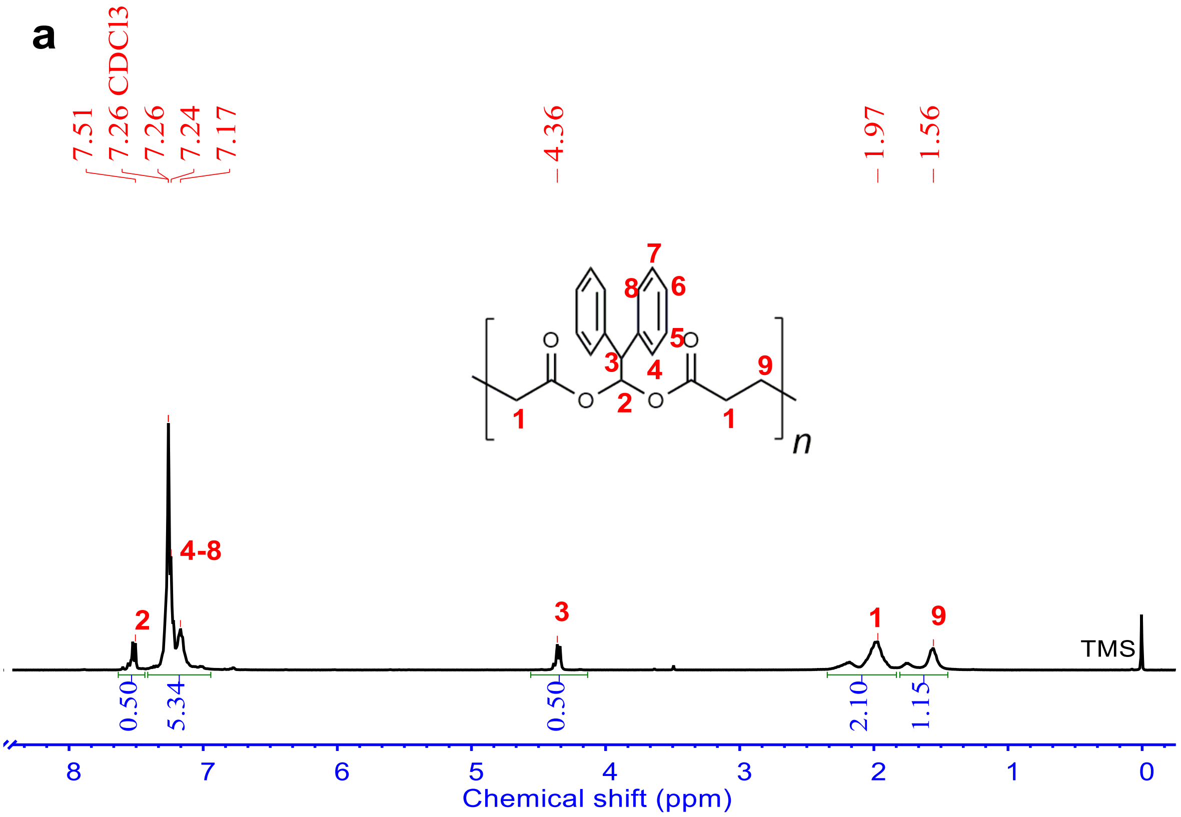

b

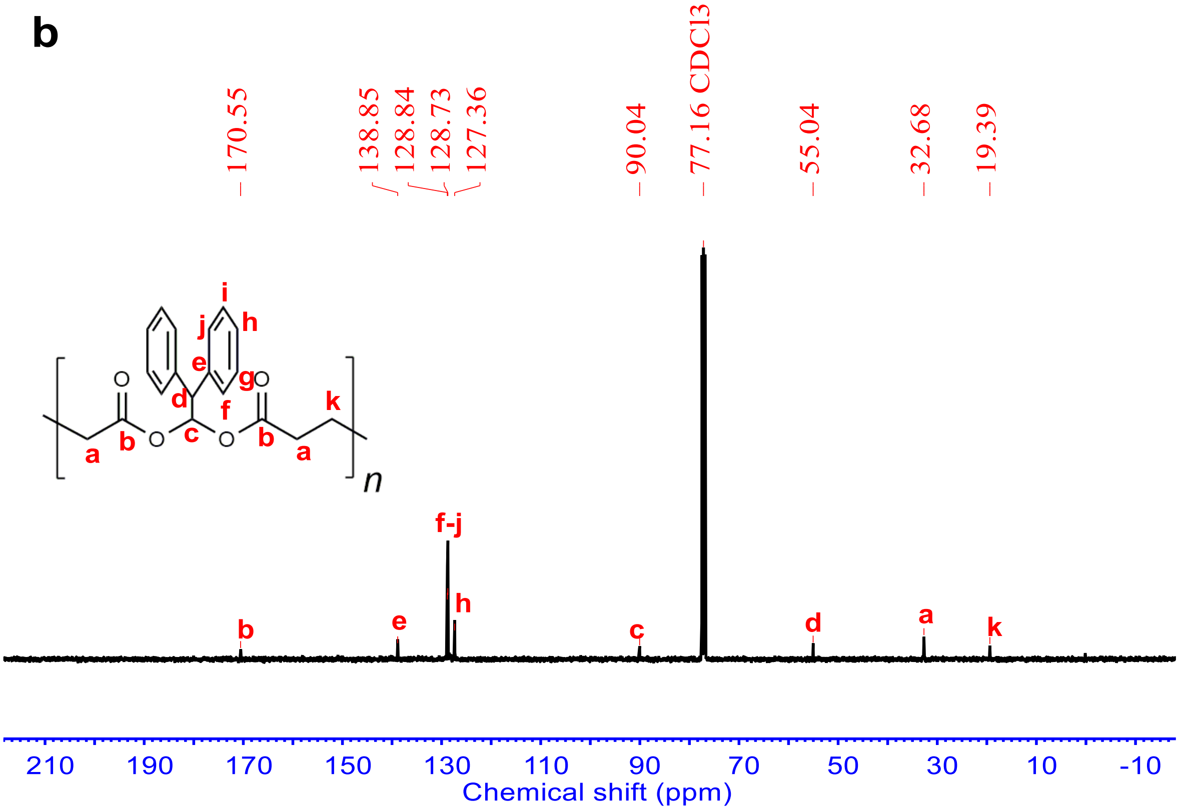

Supplementary Fig. 57 (a) ^{1}H and (b) ^{13}C NMR spectra of the obtained copolymer of **P56A** in $CDCl_3$.

a

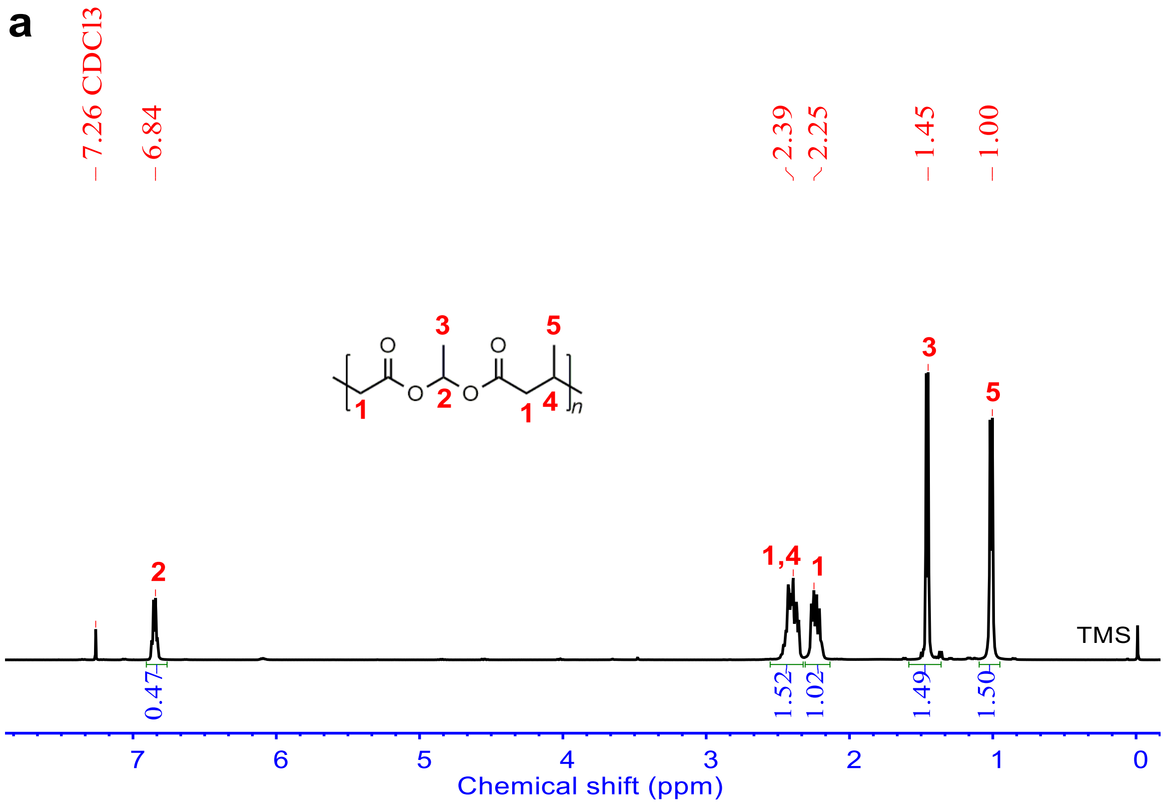

b

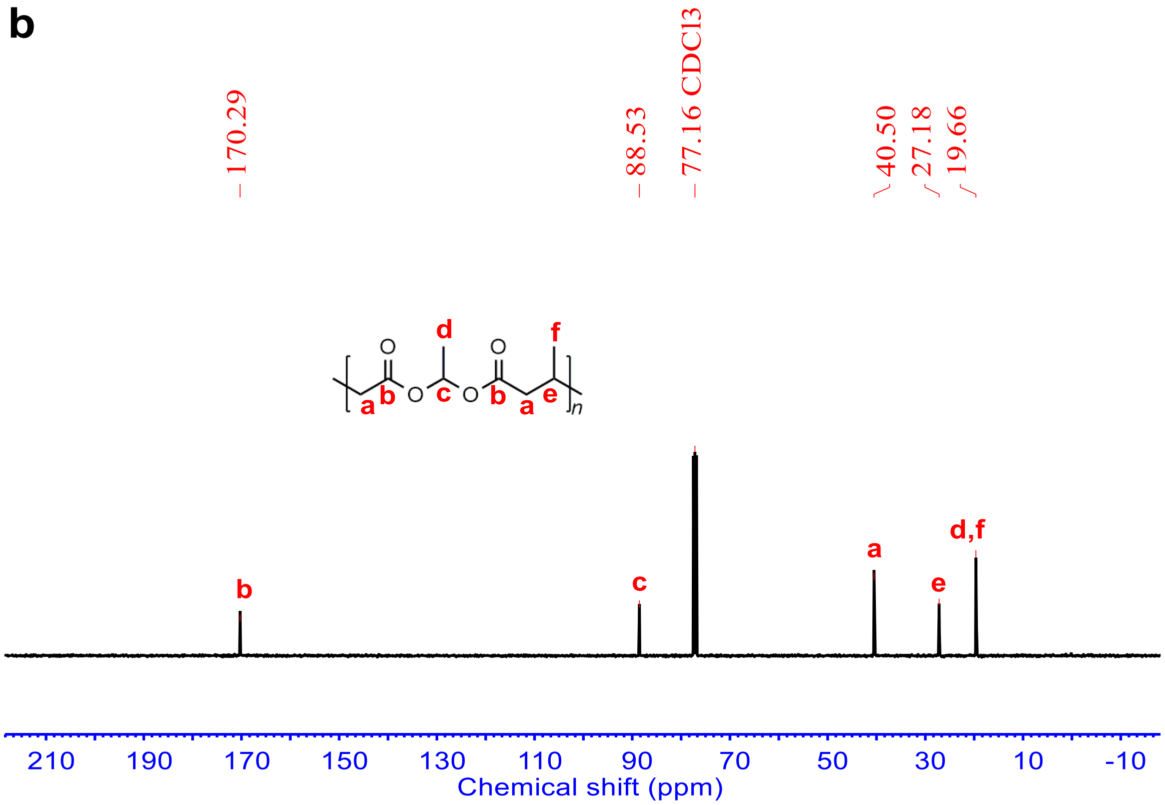

Supplementary Fig. 58 (a) ^{1}H and (b) ^{13}C NMR spectra of the obtained copolymer of **P1B** in $CDCl_3$.

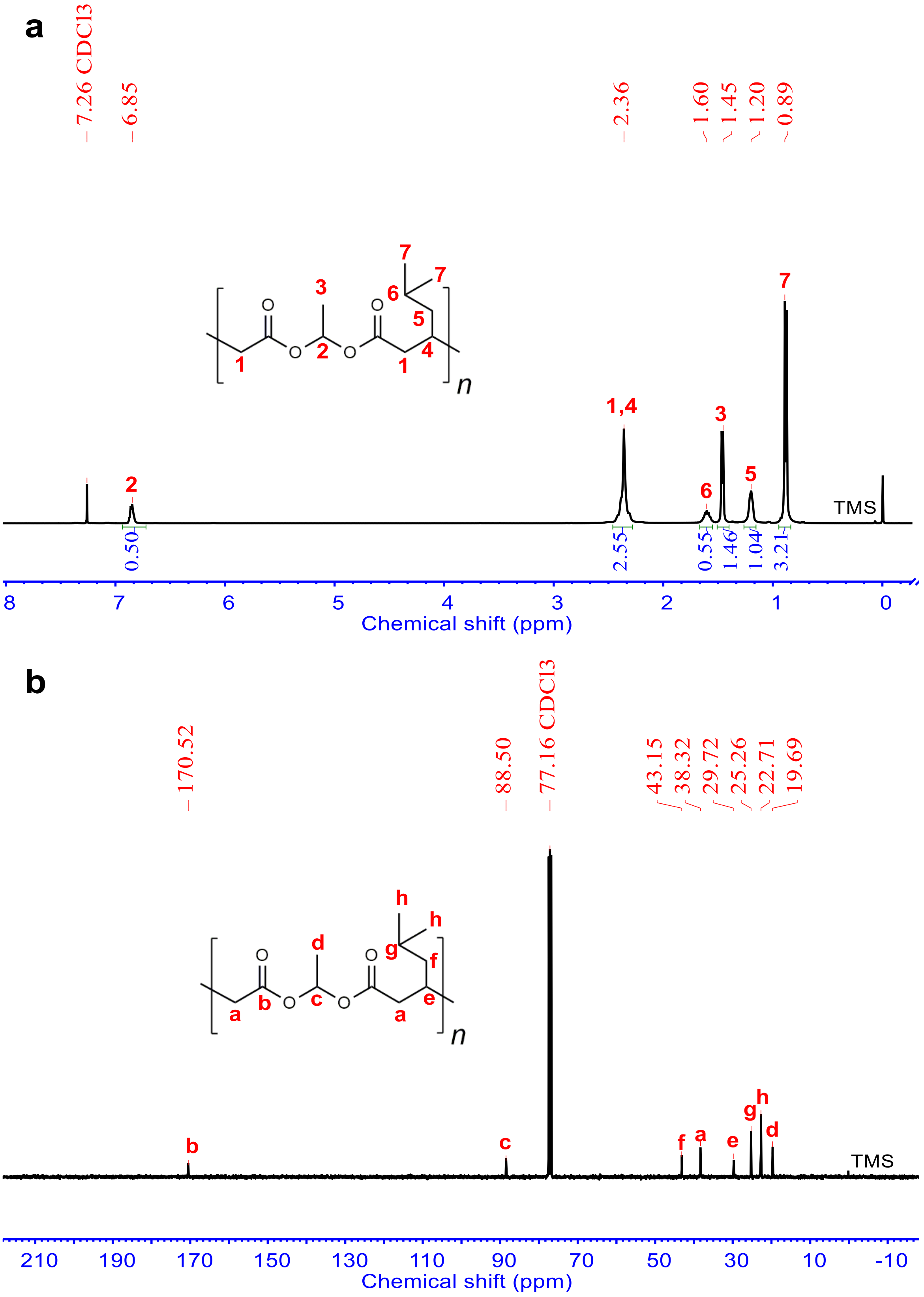

Supplementary Fig. 59 (a) ^{1}H and (b) ^{13}C NMR spectra of the obtained copolymer of **P1C** in $CDCl_3$.

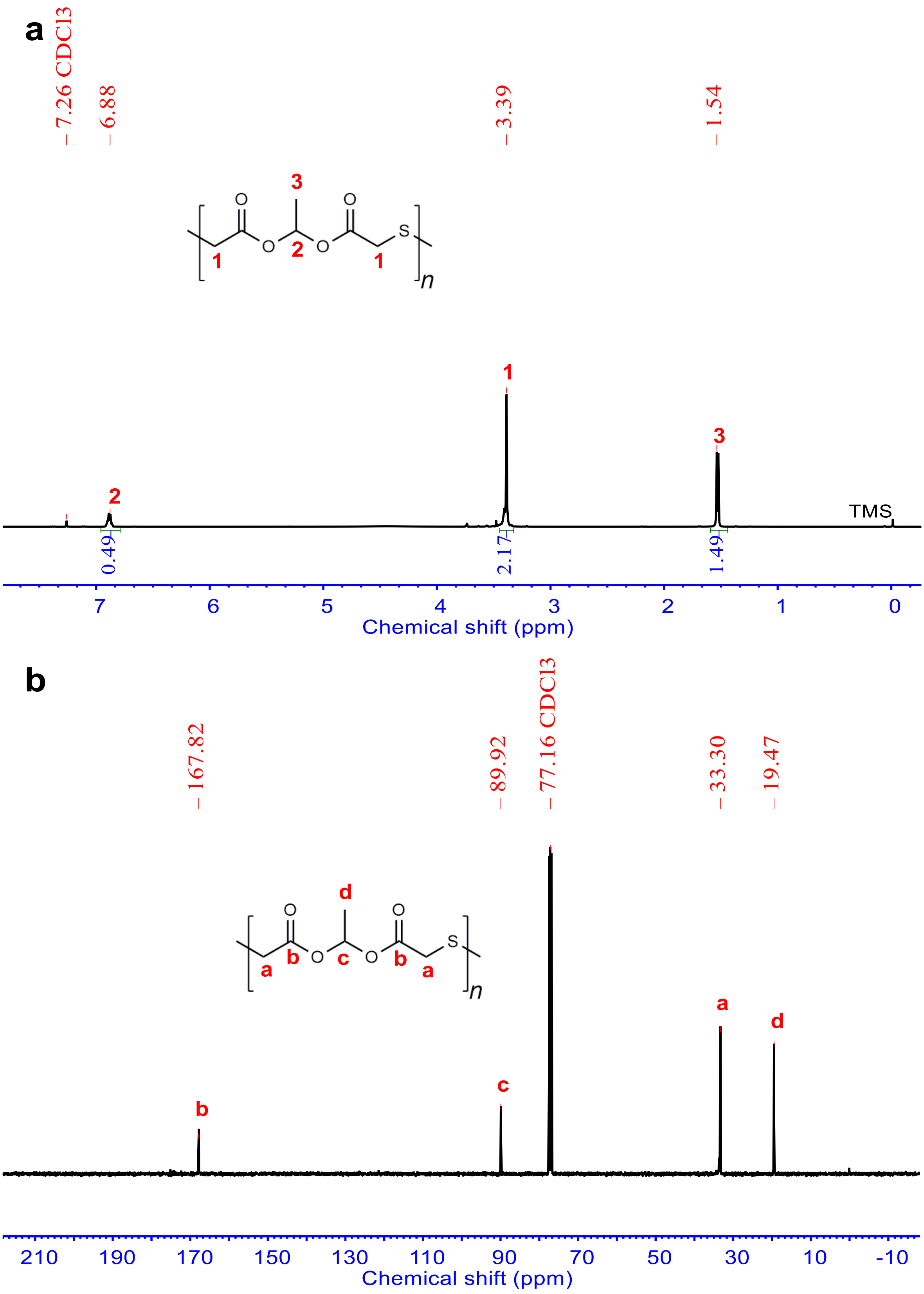

Supplementary Fig. 60 (a) ^1H and (b) ^{13}C NMR spectra of the obtained copolymer of **P1D** in $CDCl_3$.

a

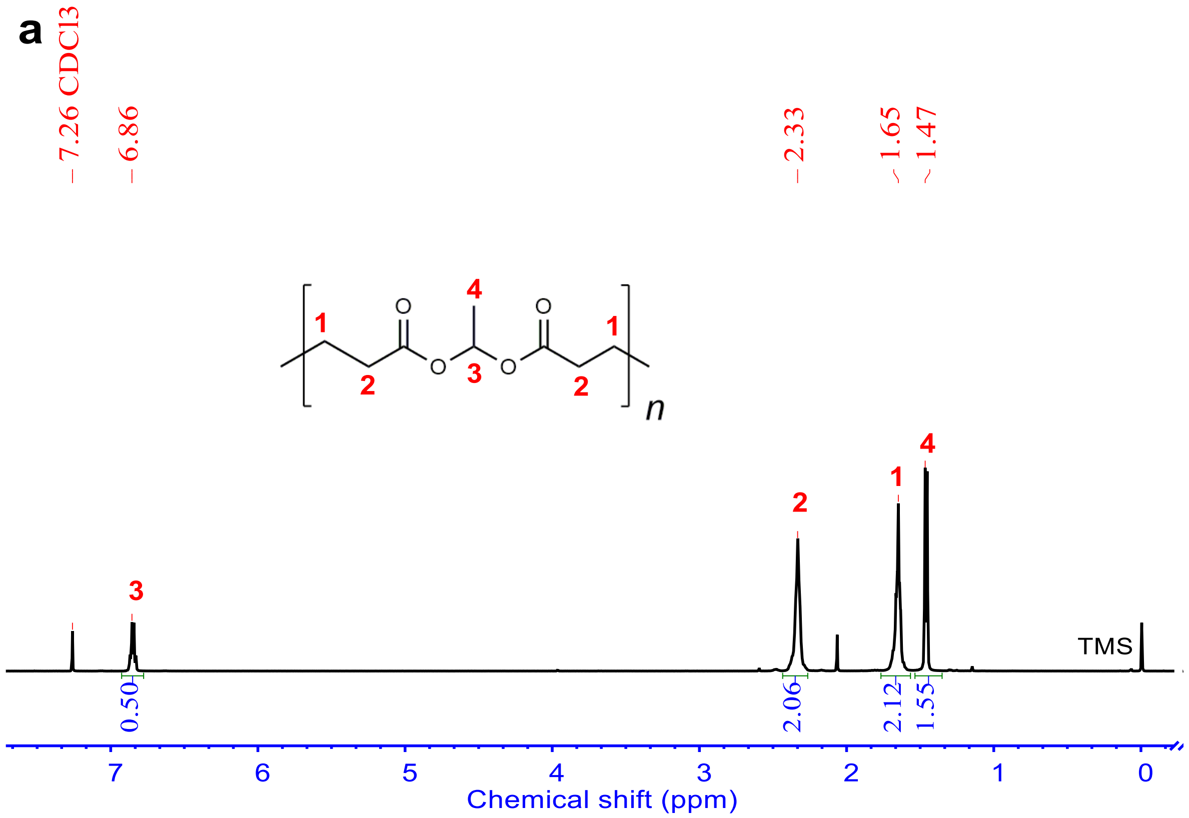

b

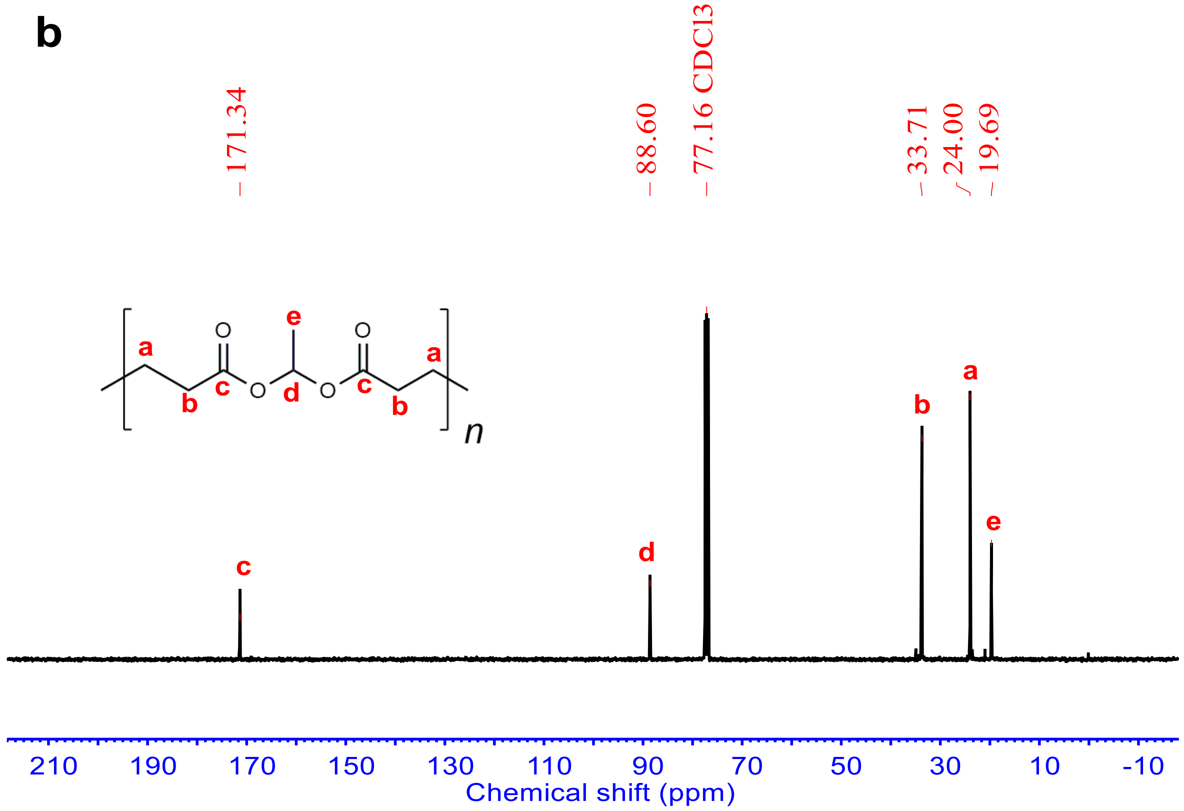

Supplementary Fig. 61 (a) ^{1}H and (b) ^{13}C NMR spectra of the obtained copolymer of **P1E** in $CDCl_3$.

Supplementary Table 3. Determination of equilibrium monomer concentration for the copolymerization of **A** and **1** [a]

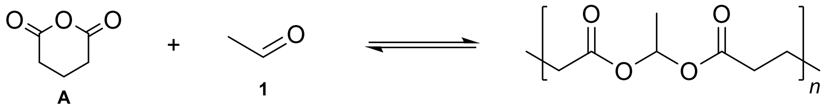

entry	T (°C)	conversion of **1** (%) [b]	conversion of **A** (%) [b]	[**1**]$_{eq}$ [c]	[**A**]$_{eq}$ [c]
1	60	93	93	0.31	0.31
2	80	88	84	0.54	0.72
3	100	74	71	1.16	1.30
4	120	45	45	2.46	2.46

[a] With [**1**]$_0$:[**A**]$_0$:[BF_3•Et_2O] = 100:100:1, [**1**]$_0$ = [**A**]$_0$ = 4.475 M, in CH_2Cl_2, for 1.5 h; [b] Conversion of the monomer, determined by ^{1}H NMR spectroscopy of the crude products; In entries 2 and 3, the conversion of **1** is slightly higher than that of **A**, which may be due to the strong volatility of **1**; [c] According to Figure 4a, the copolymerization is believed to reach equilibrium after 3 h, thus $[M]_{eq} = [M]_0$ (1-Conv.).

Supplementary Table 4. Determination of equilibrium monomer concentration for the copolymerization of **A** and **21** [a]

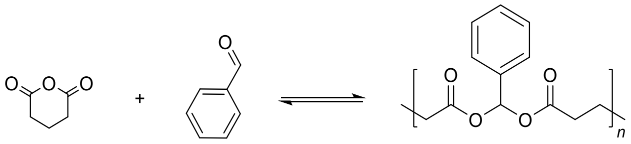

entry	T (°C)	conversion of **A** (%) [b]	[**A**]$_{eq}$ [c]
1	0	76	0.92
2	20	56	1.69
3	40	28	2.76

[a] With [**21**]$_0$:[**A**]$_0$:[BF_3•Et_2O] = 100:100:1, [**21**]$_0$ = [**A**]$_0$ = 3.833 M, in CH_2Cl_2, for 3 h; [b] Conversion of the monomer **A**, determined by ^{1}H NMR spectroscopy of the crude products; [c] According to Figure S62, the copolymerization is believed to reach equilibrium after 3 h, thus $[M]_{eq} = [M]_0$ (1-Conv.).

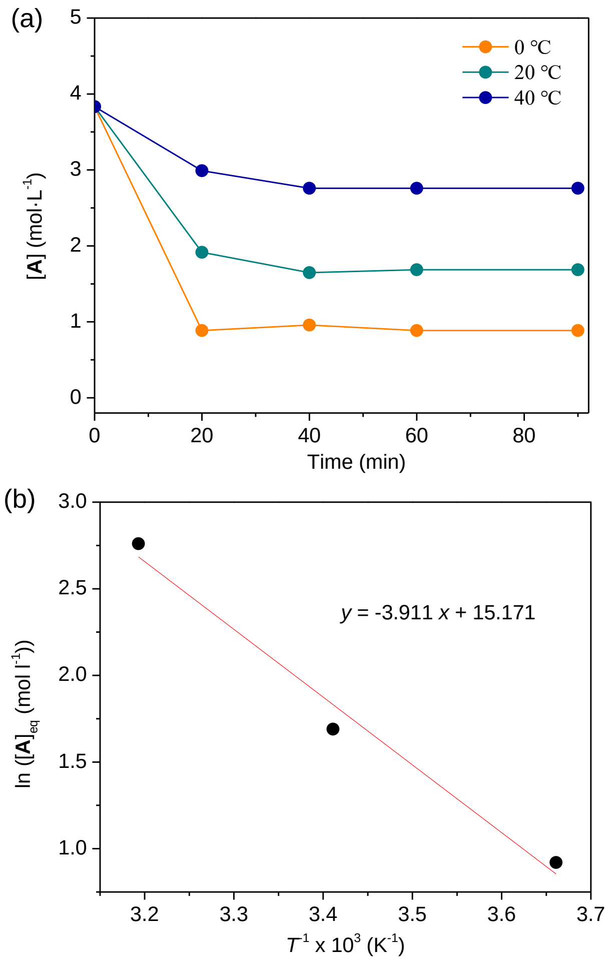

Supplementary Fig. 62 Thermodynamics of the **21** and **A** copolymerization. (a) Plot of **A** concentration as a function of time during polymerization at different temperatures. (b) Van't Hoff plot of ln[**A**]$_{eq}$ versus the reciprocal of the absolute temperature (T^{-1}).

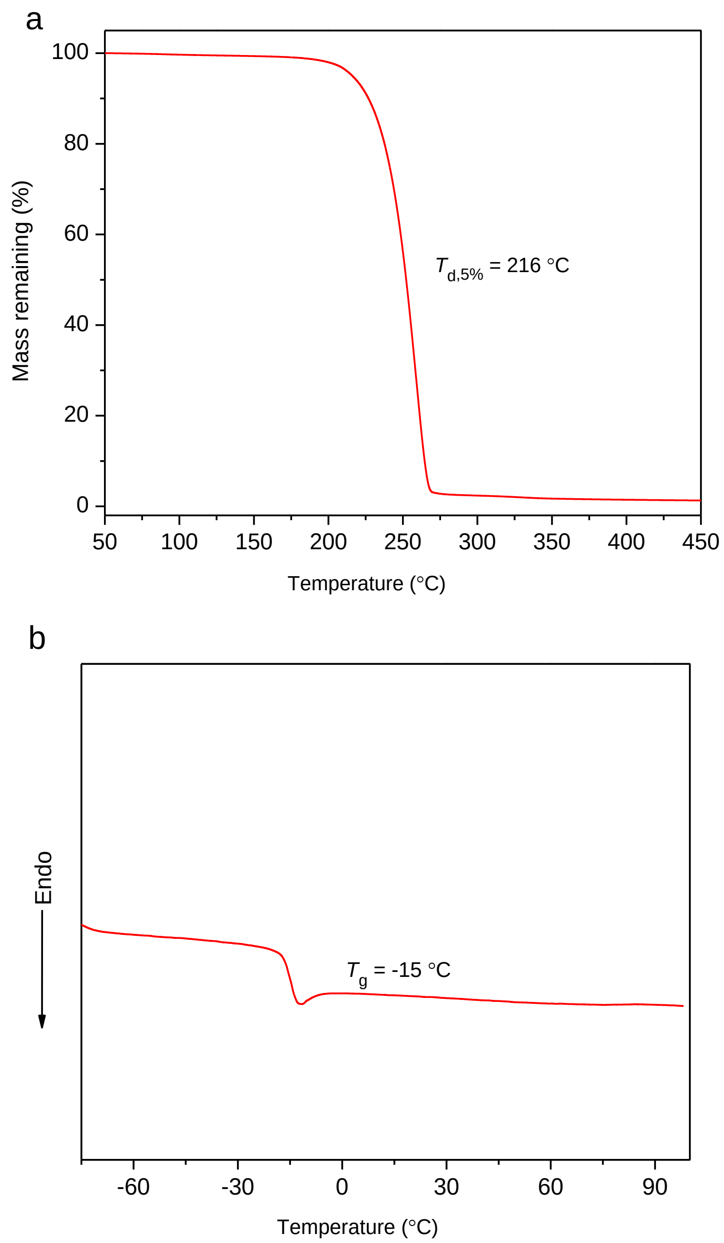

Supplementary Fig. 63 (a) TGA and (b) DSC curves of the obtained copolymer **P1A**.

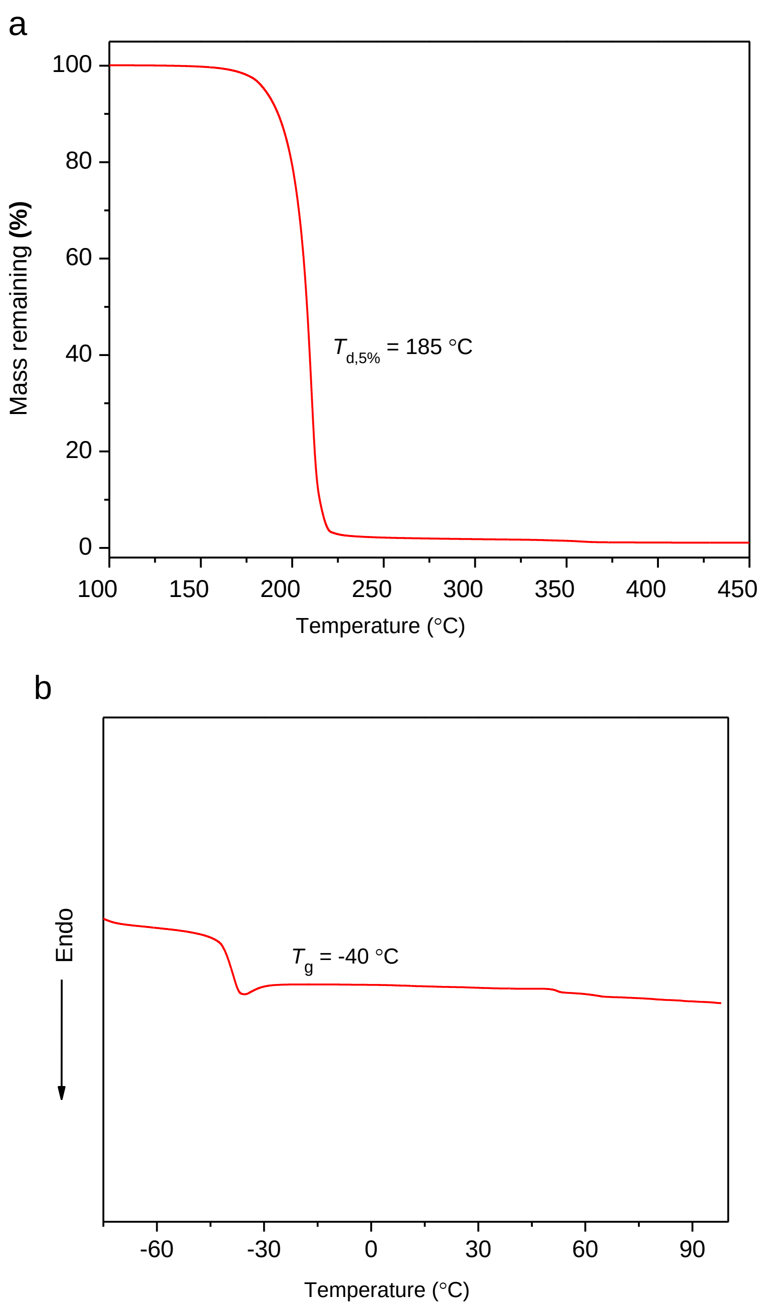

Supplementary Fig. 64 (a) TGA and (b) DSC curves of the obtained copolymer **P2A**.

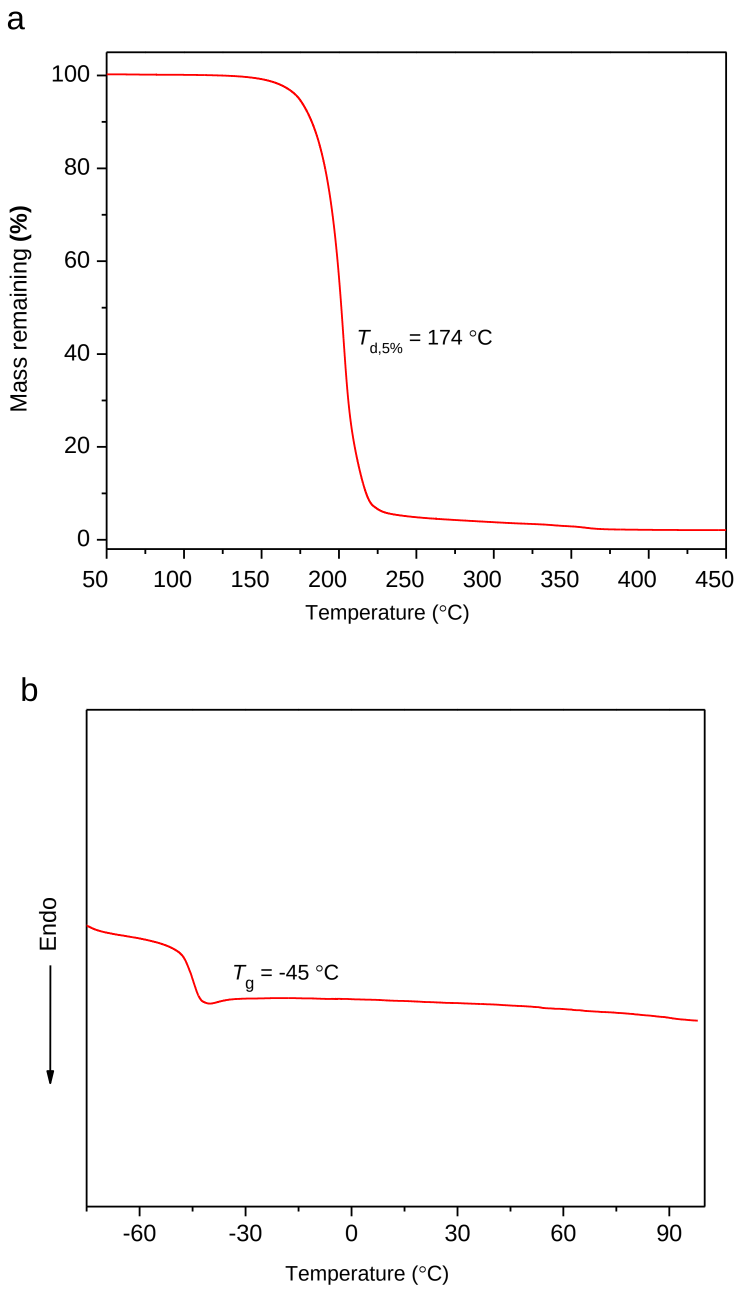

Supplementary Fig. 65 (a) TGA and (b) DSC curves of the obtained copolymer **P3A**.

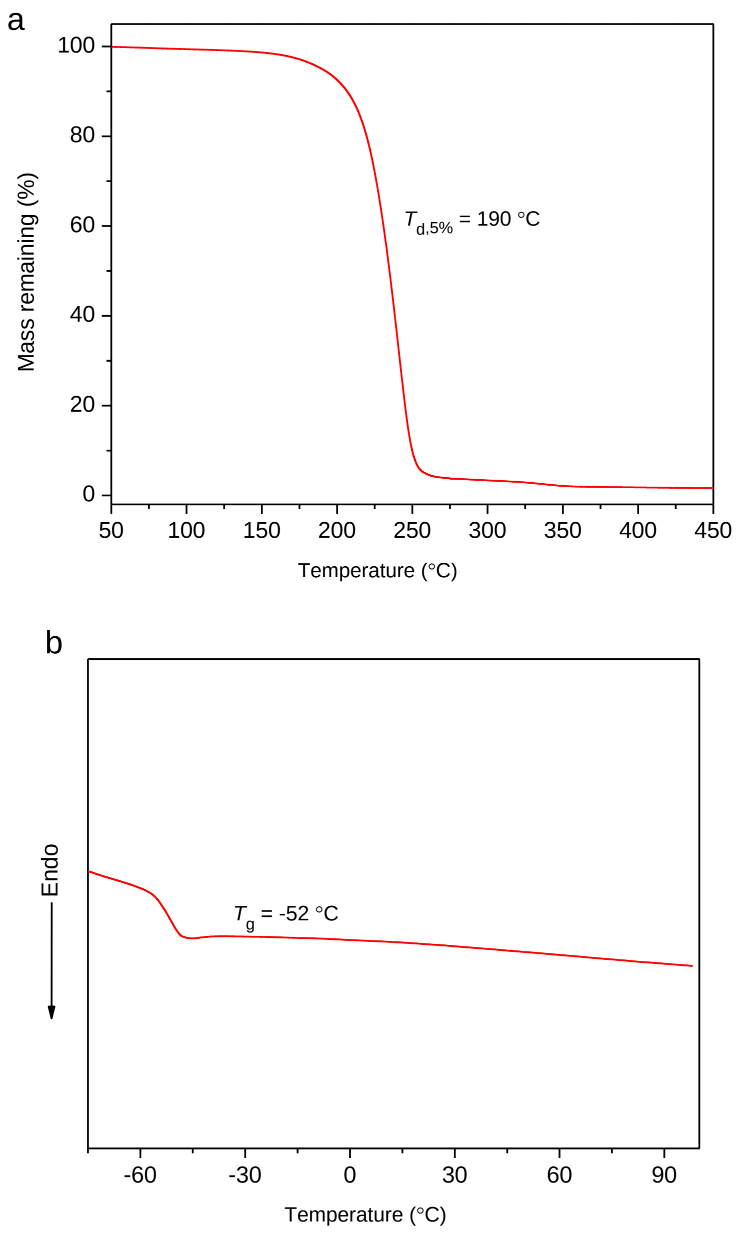

Supplementary Fig. 66 (a) TGA and (b) DSC curves of the obtained copolymer **P4A**.

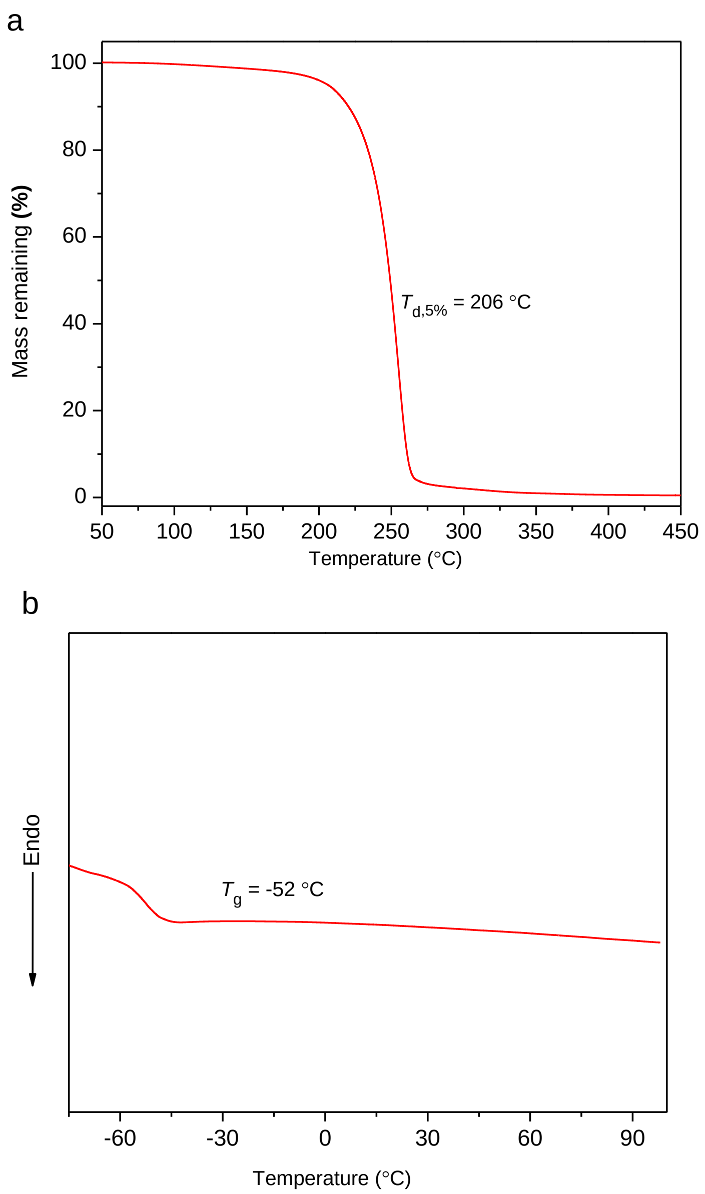

Supplementary Fig. 67 (a) TGA and (b) DSC curves of the obtained copolymer **P5A**.

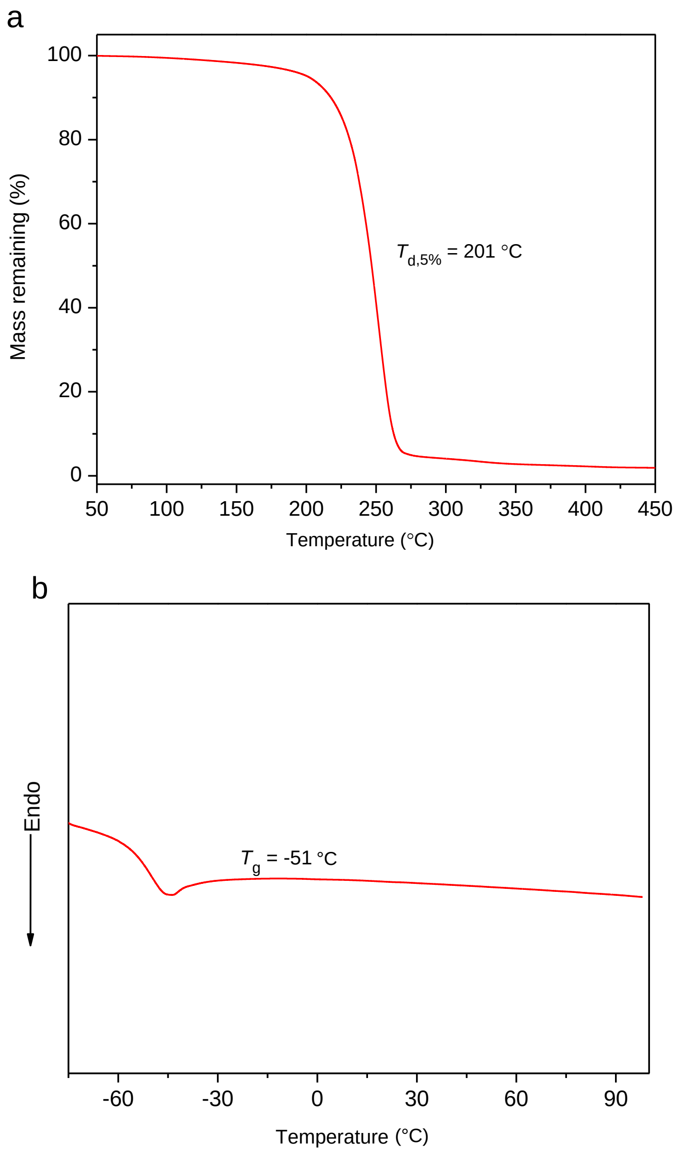

Supplementary Fig. 68 (a) TGA and (b) DSC curves of the obtained copolymer **P6A**.

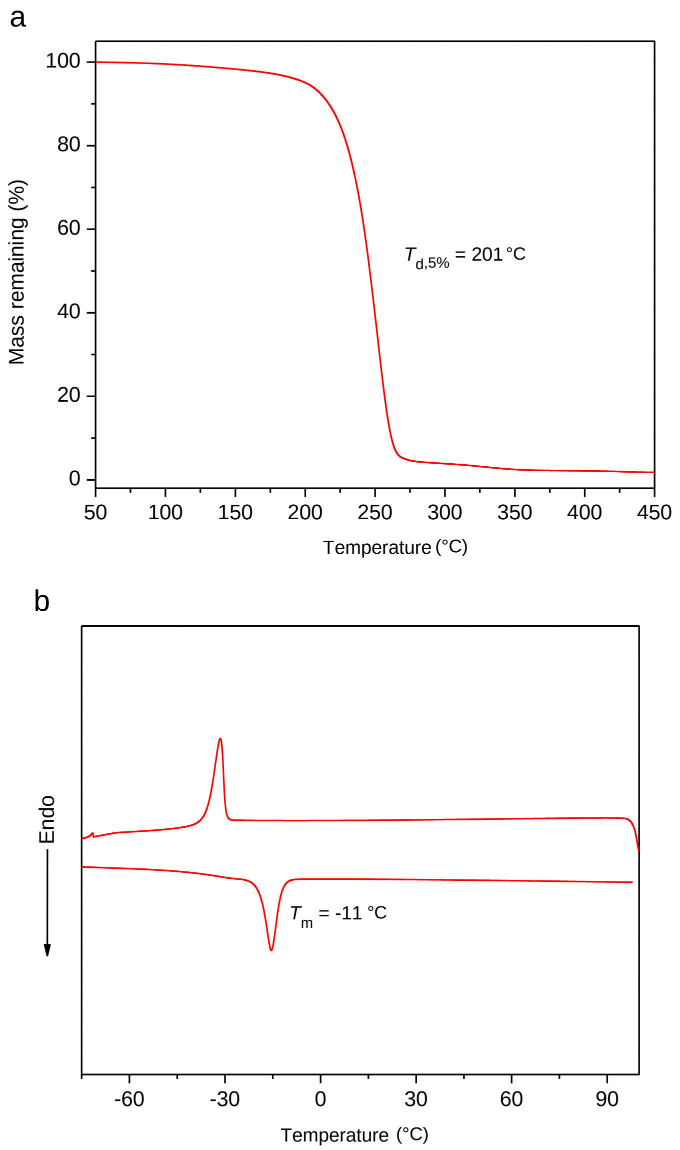

Supplementary Fig. 69 (a) TGA and (b) DSC curves of the obtained copolymer **P7A**.

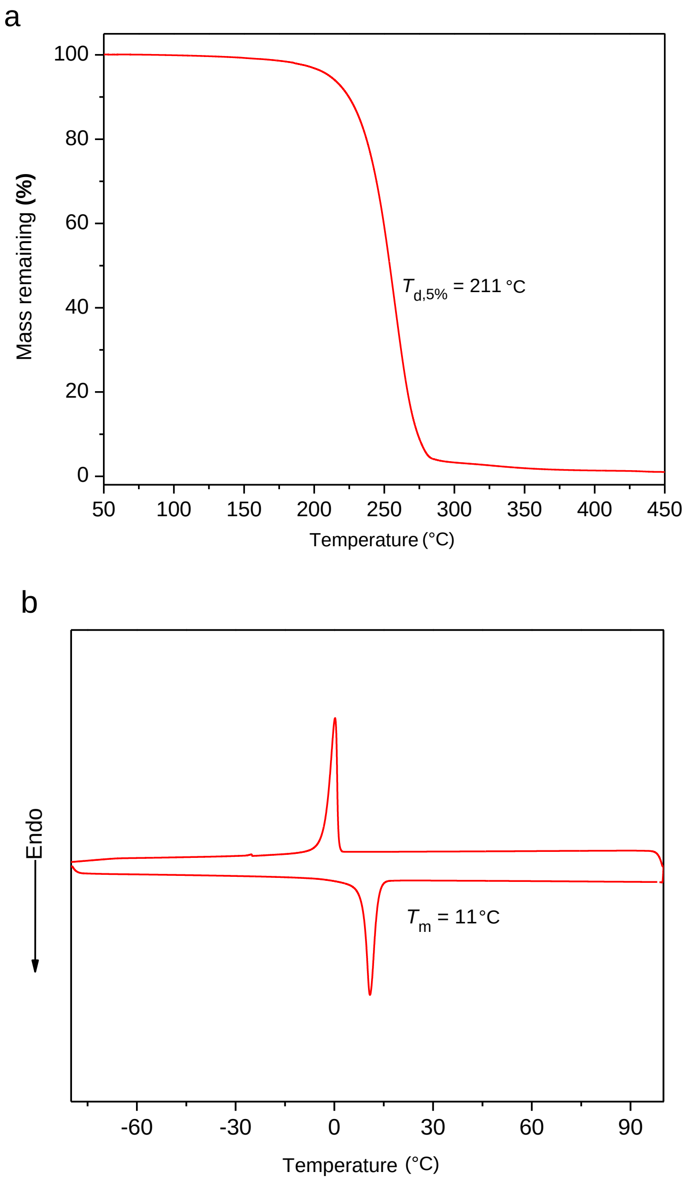

Supplementary Fig. 70 (a) TGA and (b) DSC curves of the obtained copolymer **P8A**.

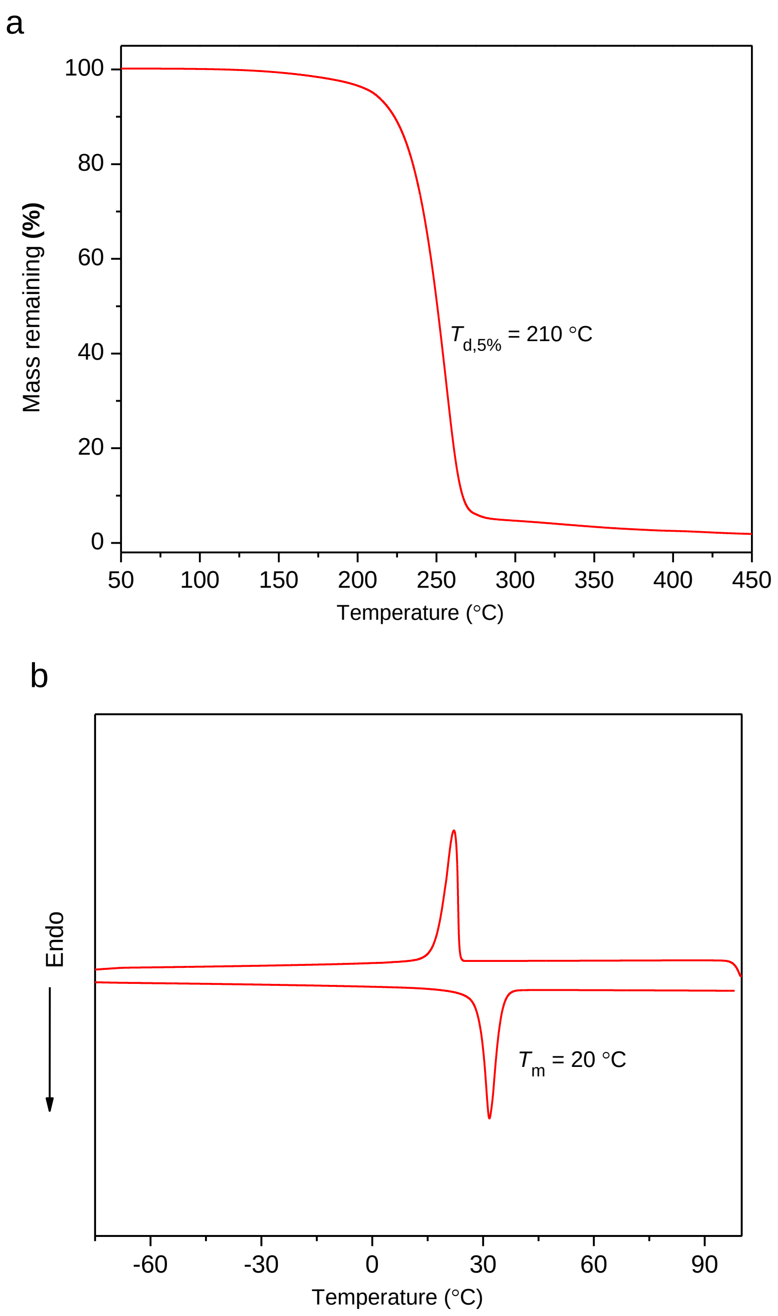

Supplementary Fig. 71 (a) TGA and (b) DSC curves of the obtained copolymer of **P9A**.

a

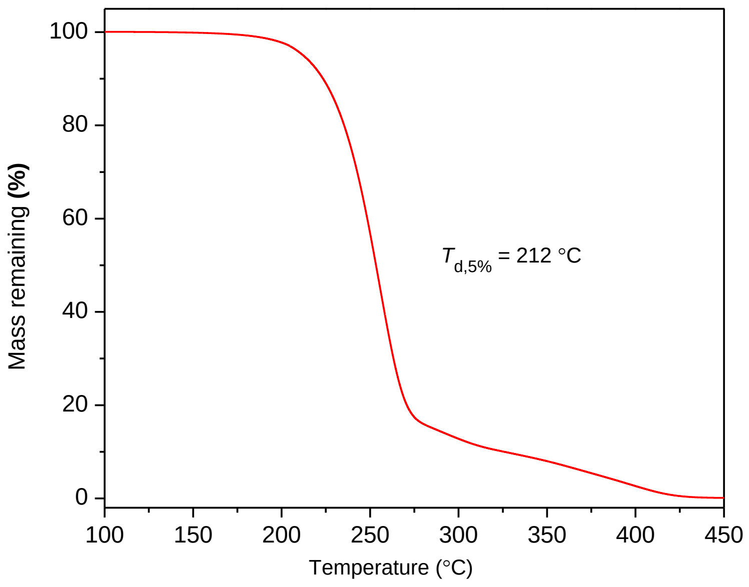

b

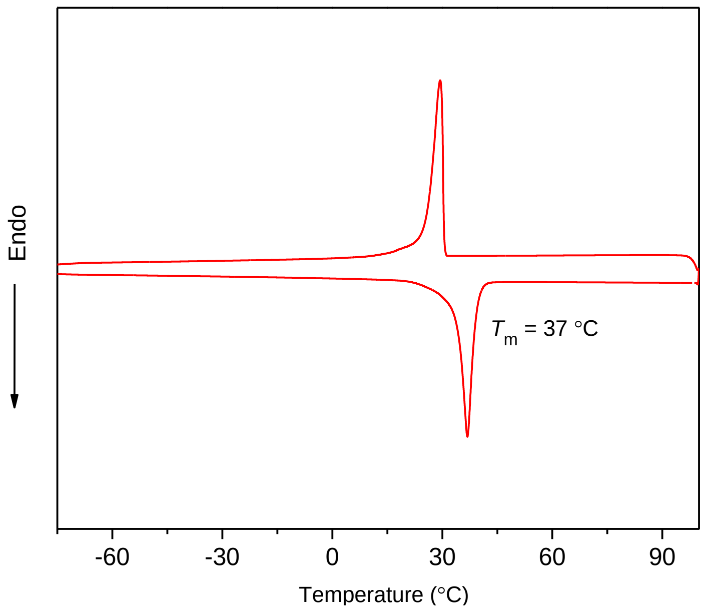

Supplementary Fig. 72 (a) TGA and (b) DSC curves of the obtained copolymer of **P10A**.

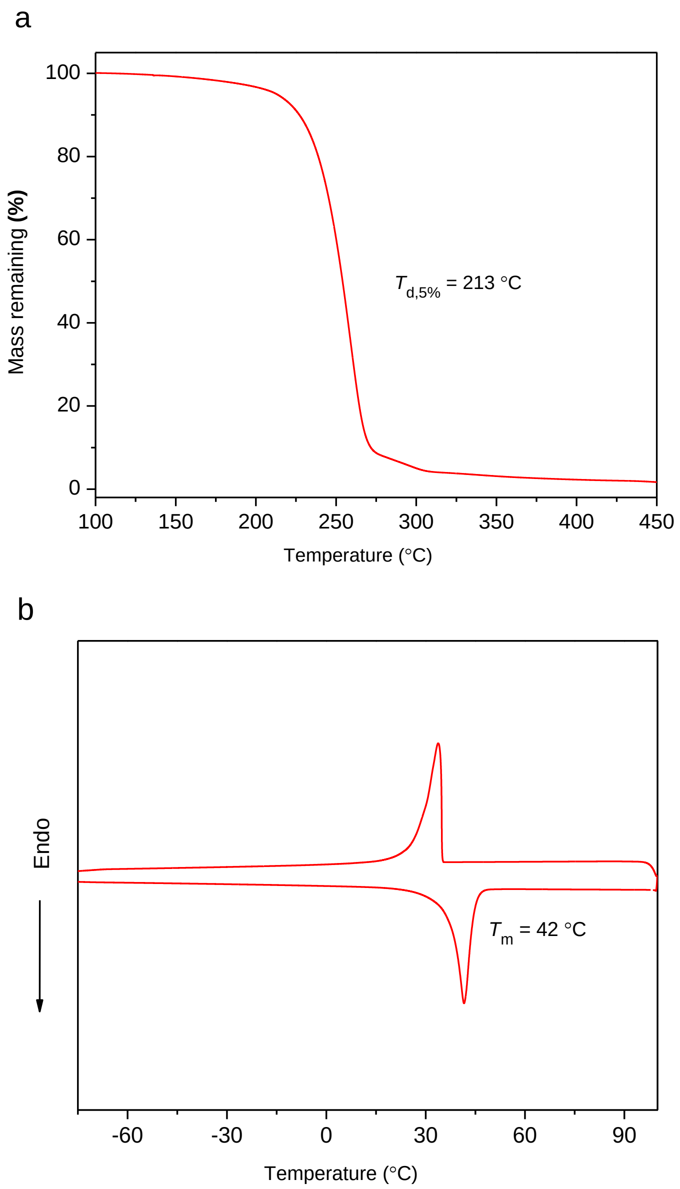

Supplementary Fig. 73 (a) TGA and (b) DSC curves of the obtained copolymer of **P11A**.

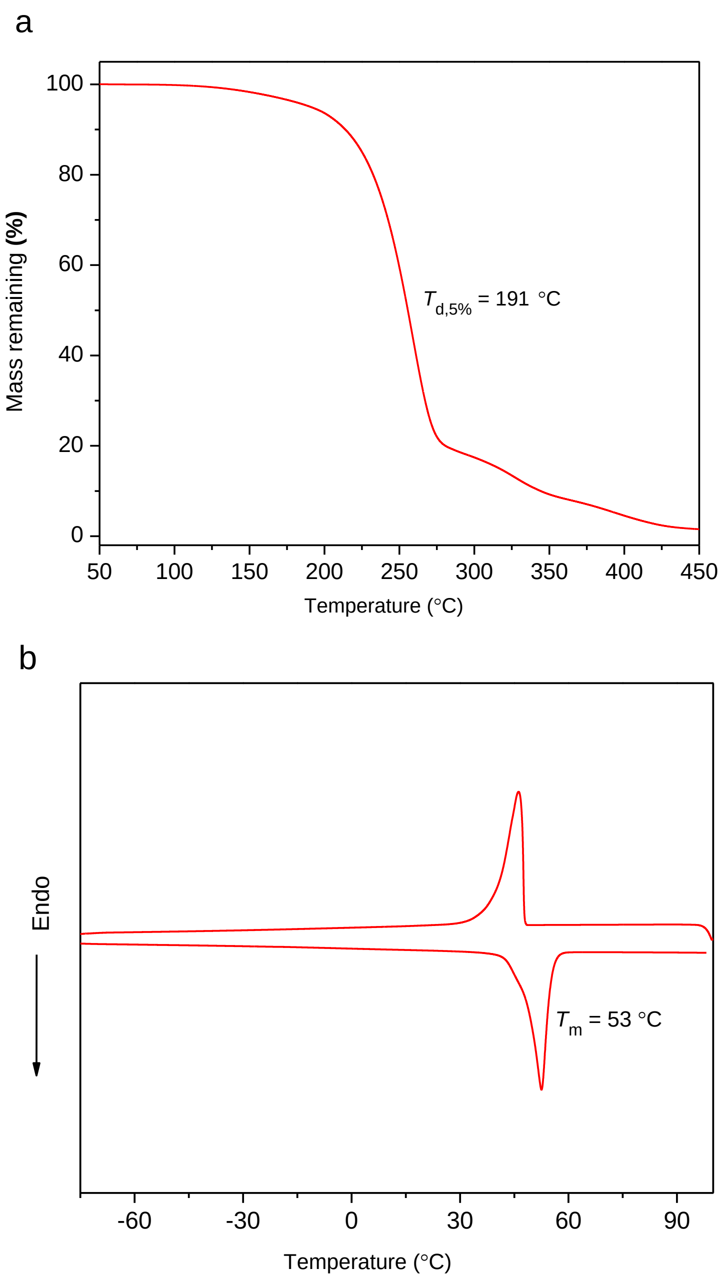

Supplementary Fig. 74 (a) TGA and (b) DSC curves of the obtained copolymer of **P12A**.

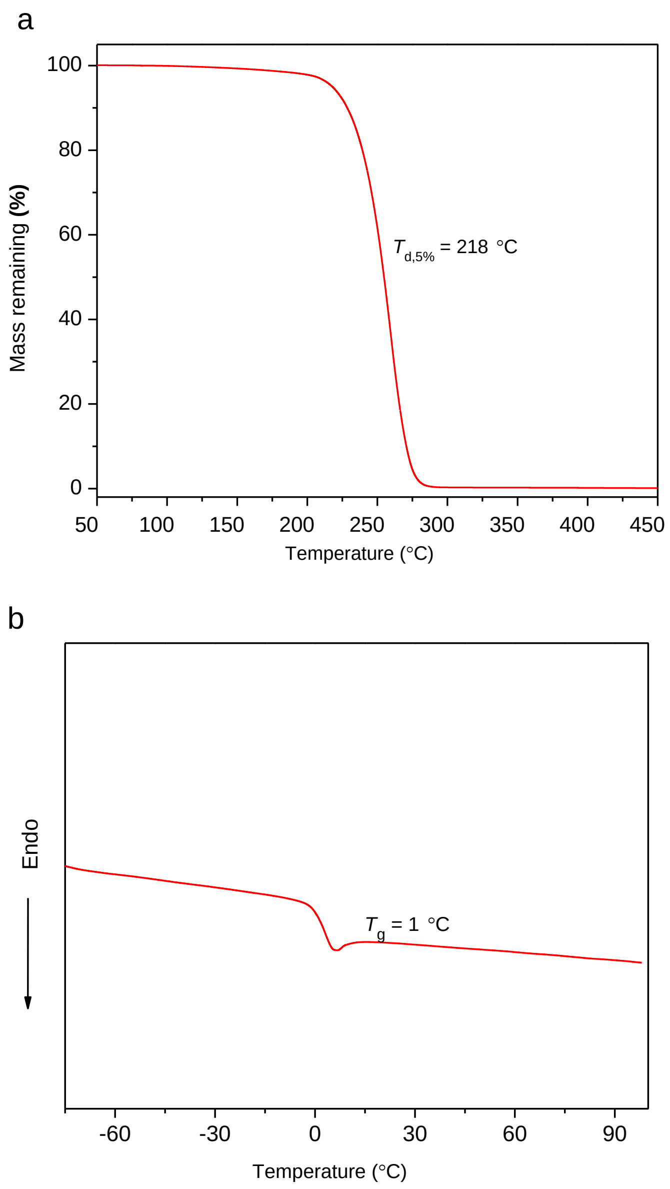

Supplementary Fig. 75 (a) TGA and (b) DSC curves of the obtained copolymer of **P13A**.

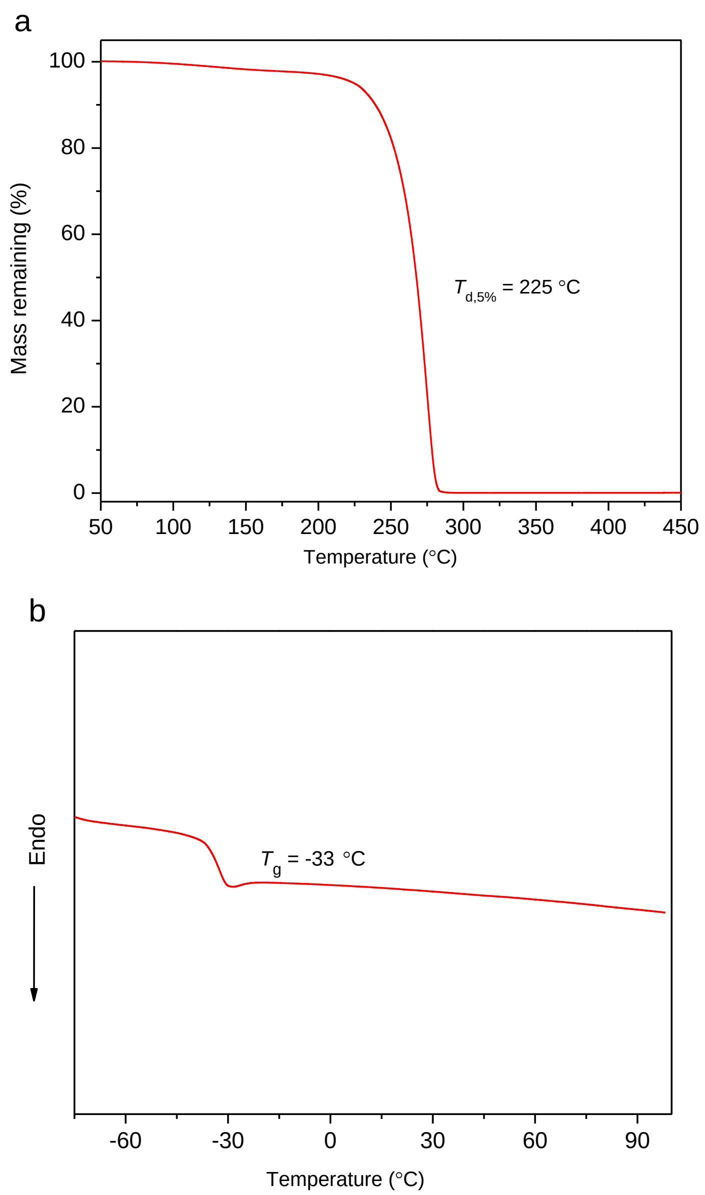

Supplementary Fig. 76 (a) TGA and (b) DSC curves of the obtained copolymer of **P14A**.

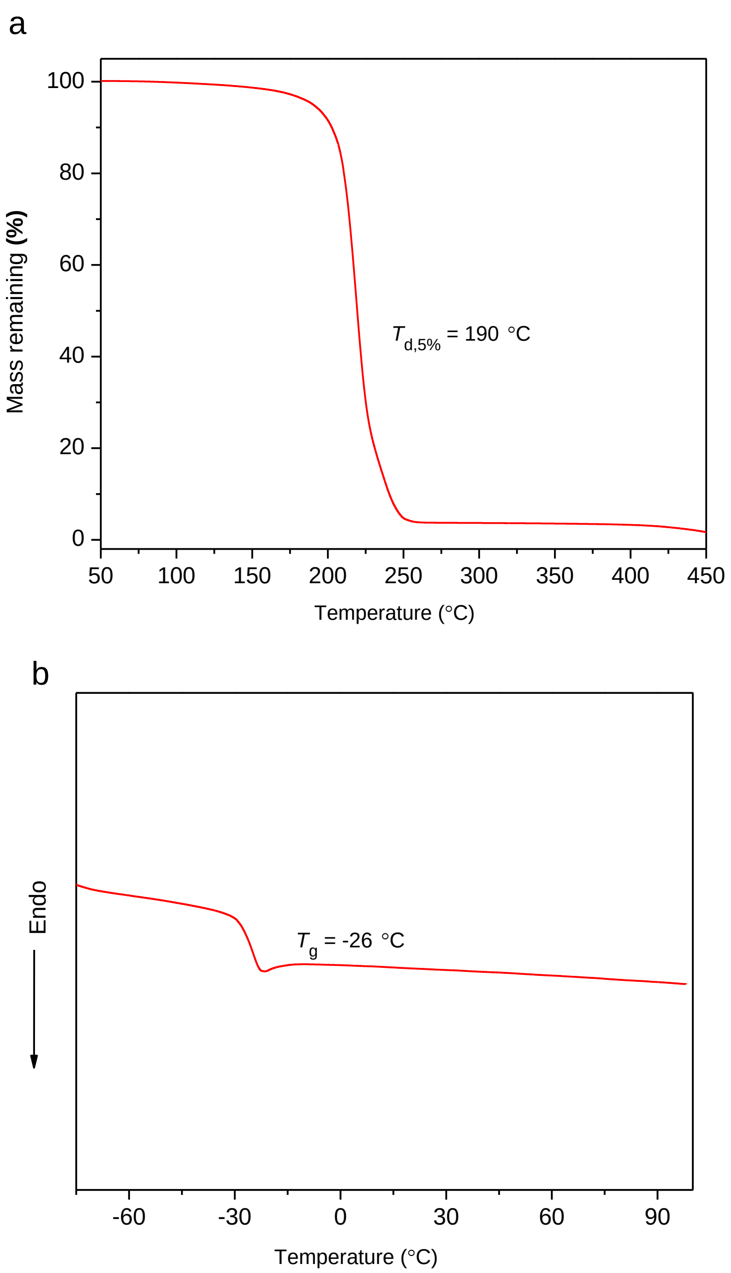

Supplementary Fig. 77 (a) TGA and (b) DSC curves of the obtained copolymer of **P15A**.

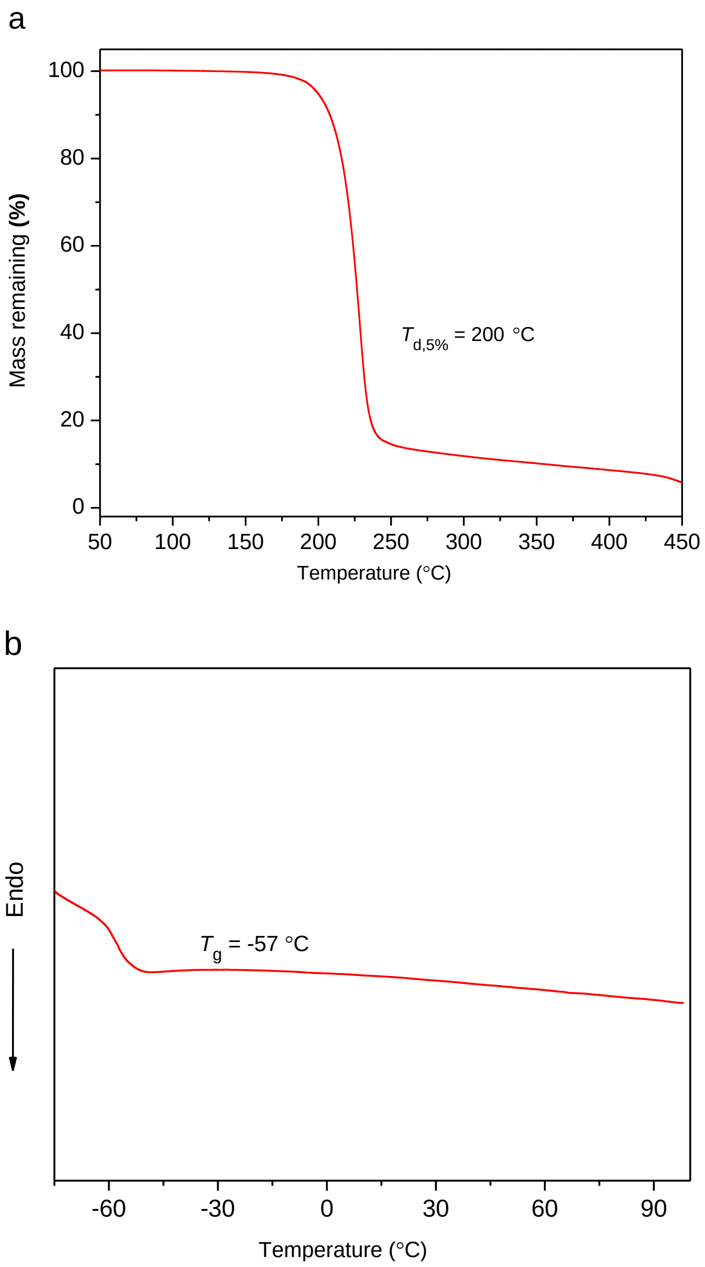

Supplementary Fig. 78 (a) TGA and (b) DSC curves of the obtained copolymer of **P16A**.

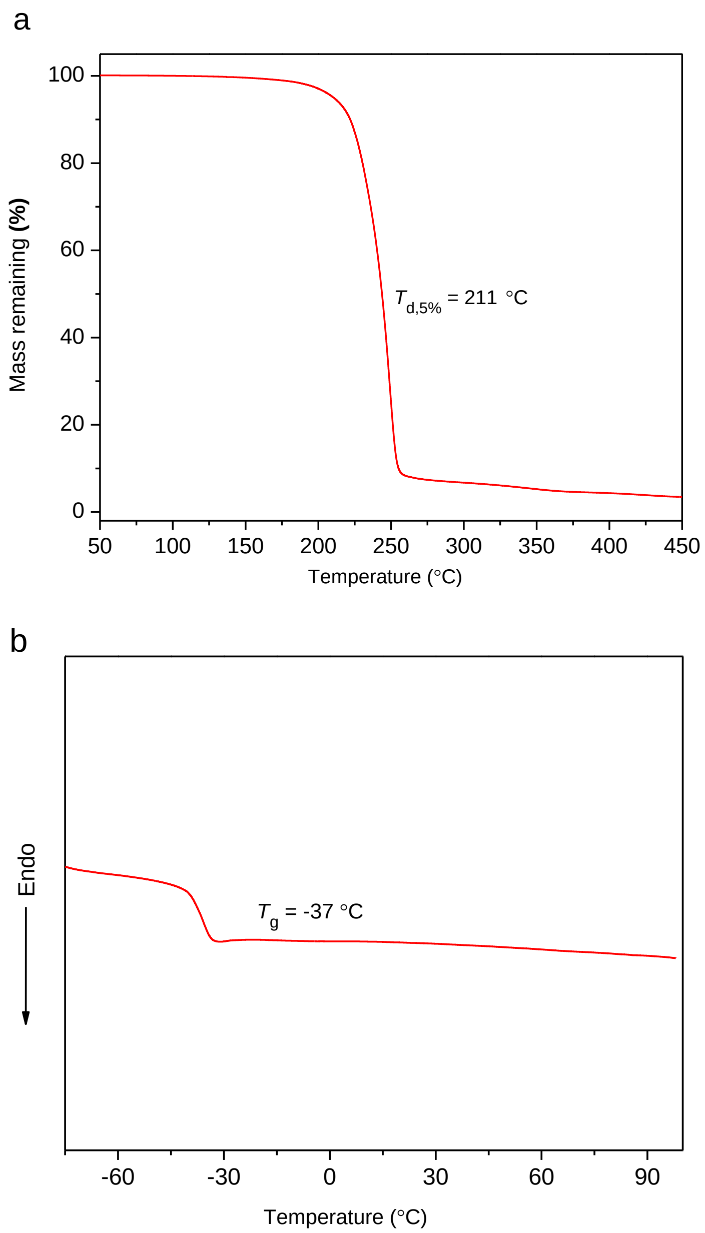

Supplementary Fig. 79 (a) TGA and (b) DSC curves of the obtained copolymer of **P17A**.

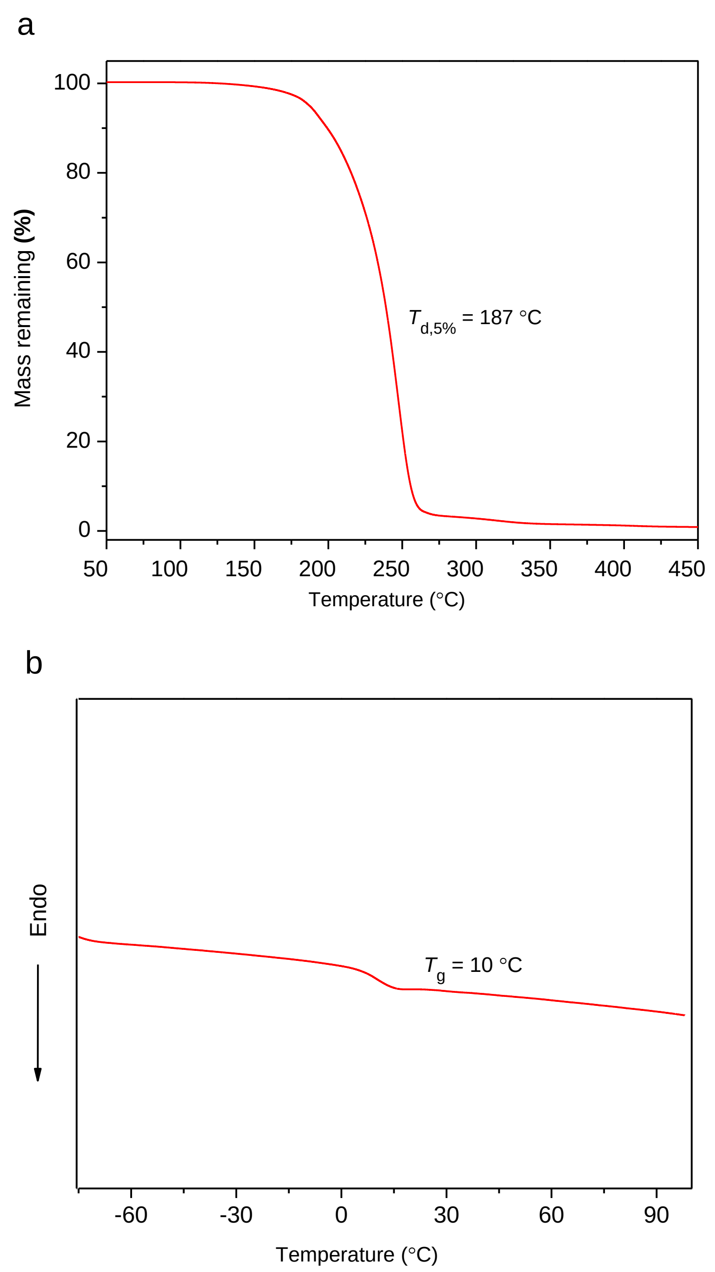

Supplementary Fig. 80 (a) TGA and (b) DSC curves of the obtained copolymer of **P18A**.

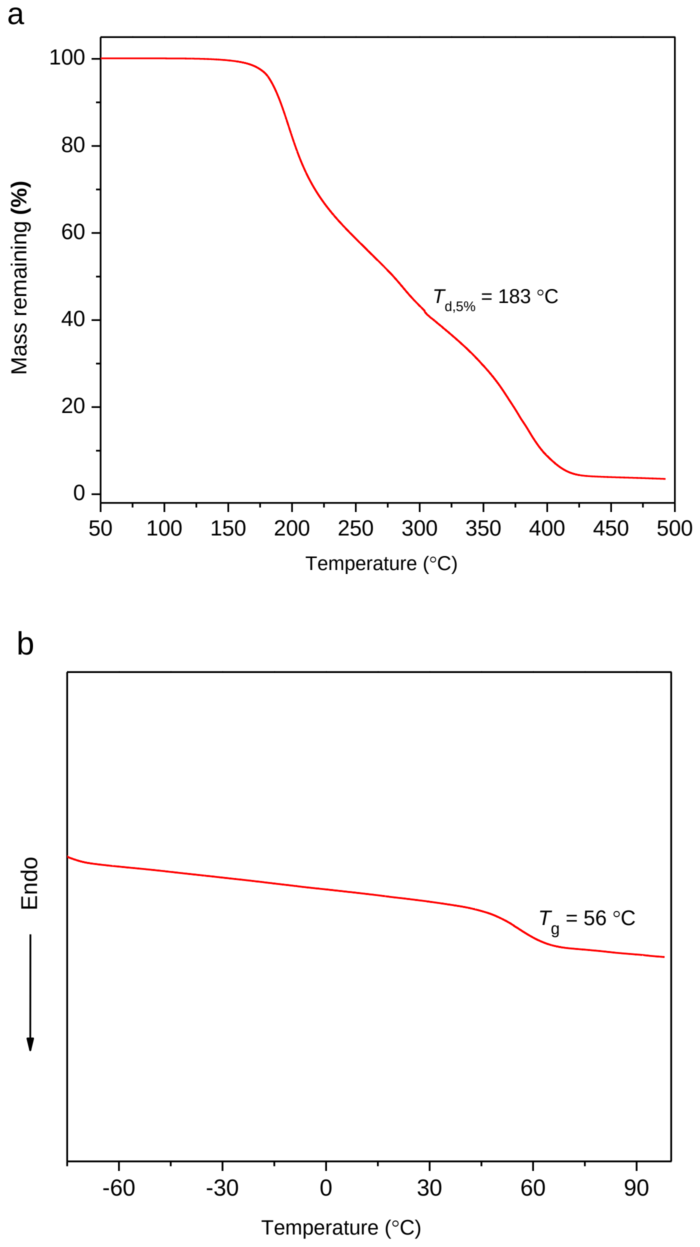

Supplementary Fig. 81 (a) TGA and (b) DSC curves of the obtained copolymer of **P19A**.

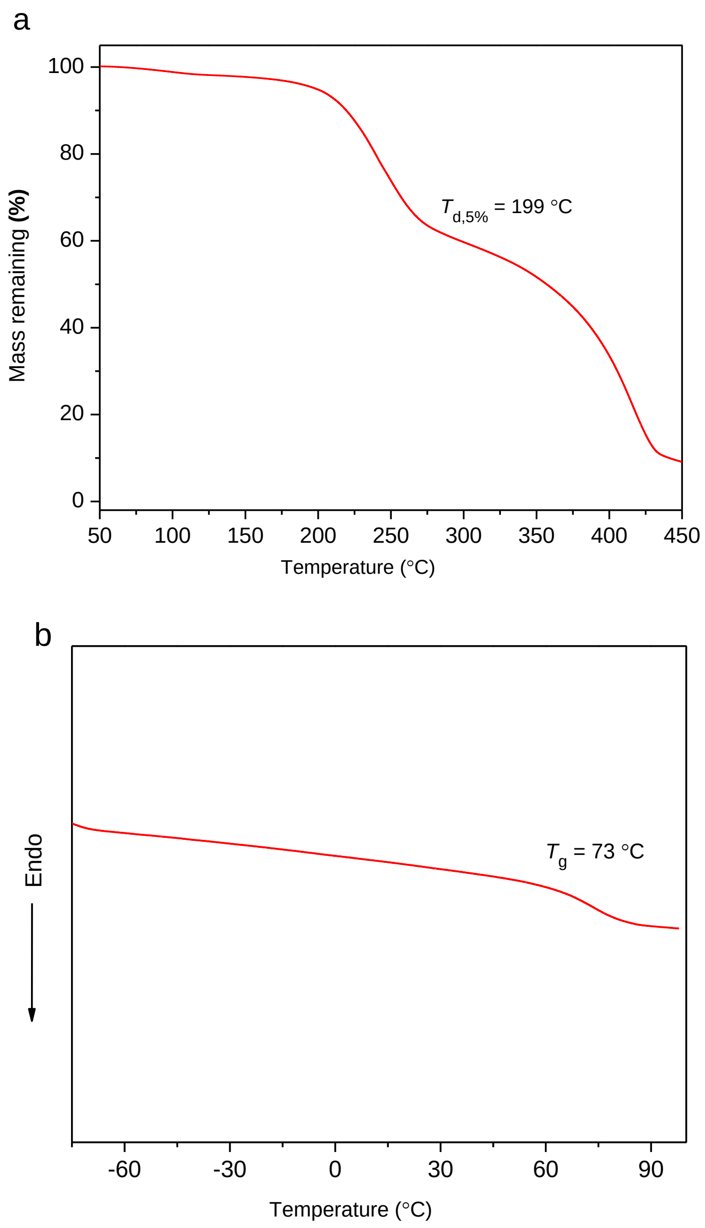

Supplementary Fig. 82 (a) TGA and (b) DSC curves of the obtained copolymer of **P20A**.

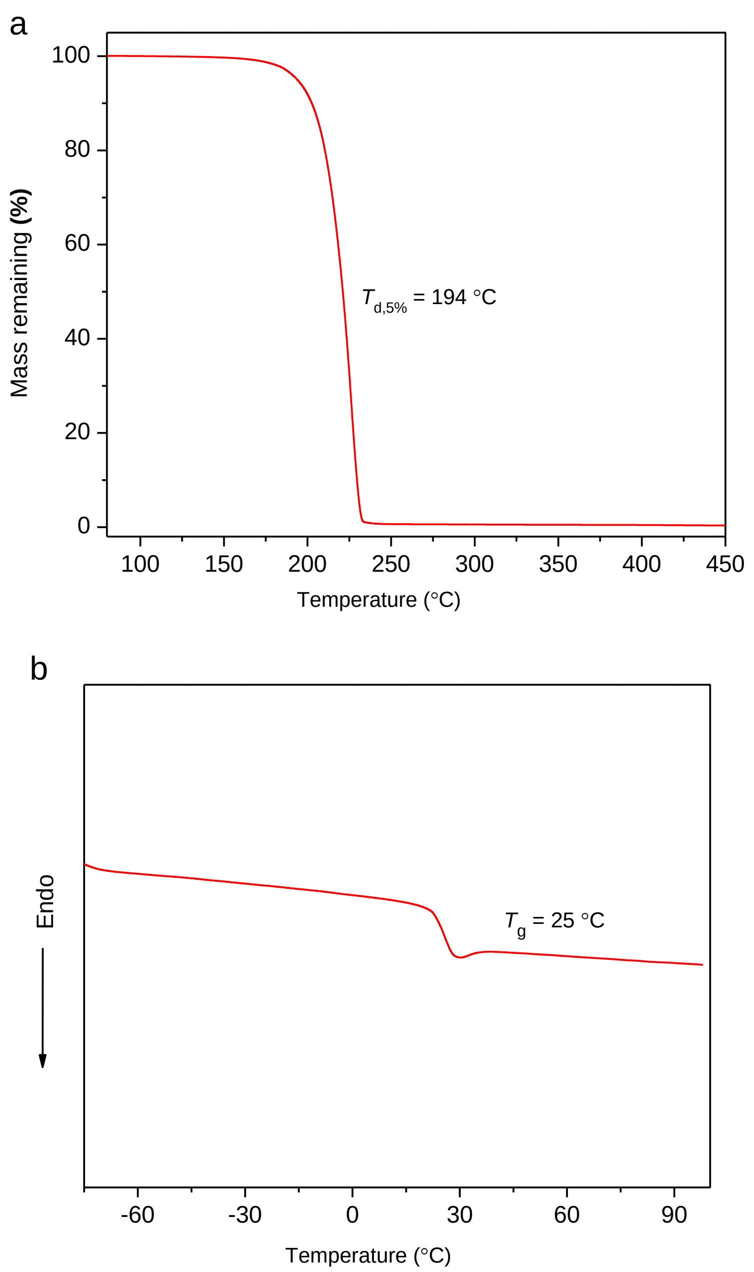

Supplementary Fig. 83 (a) TGA and (b) DSC curves of the obtained copolymer of **P21A.**

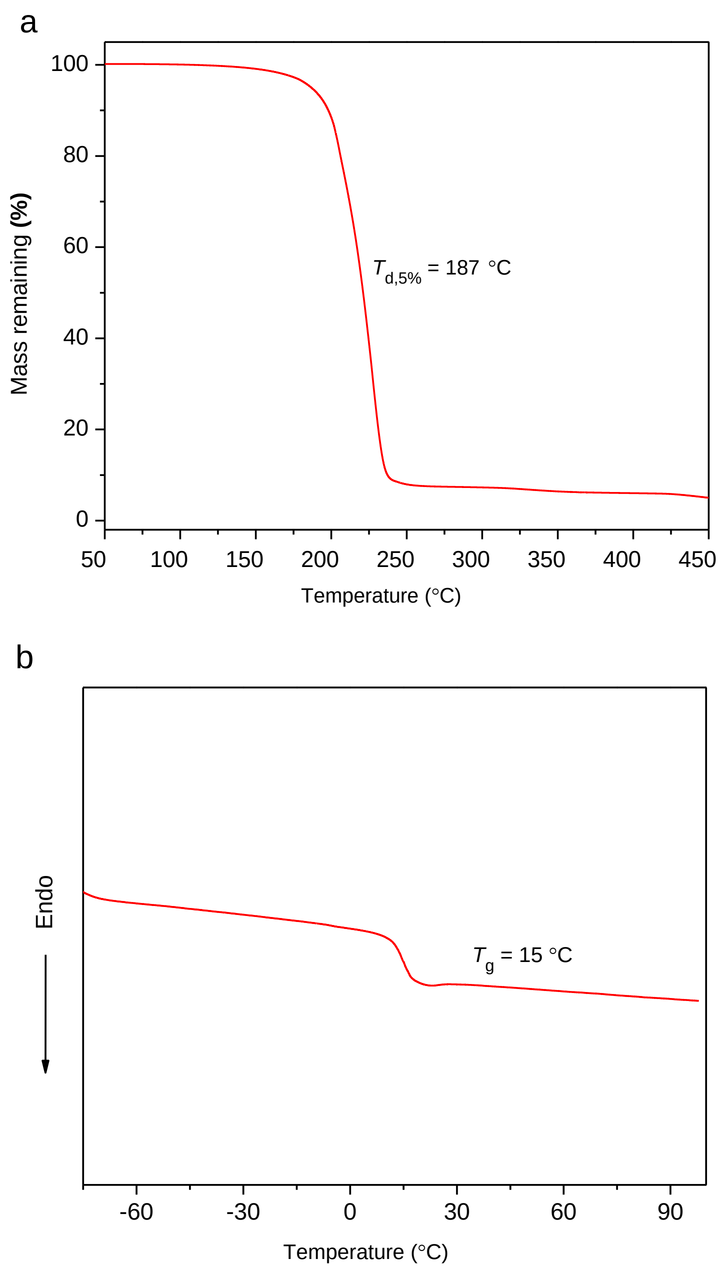

Supplementary Fig. 84 (a) TGA and (b) DSC curves of the obtained copolymer of **P22A**.

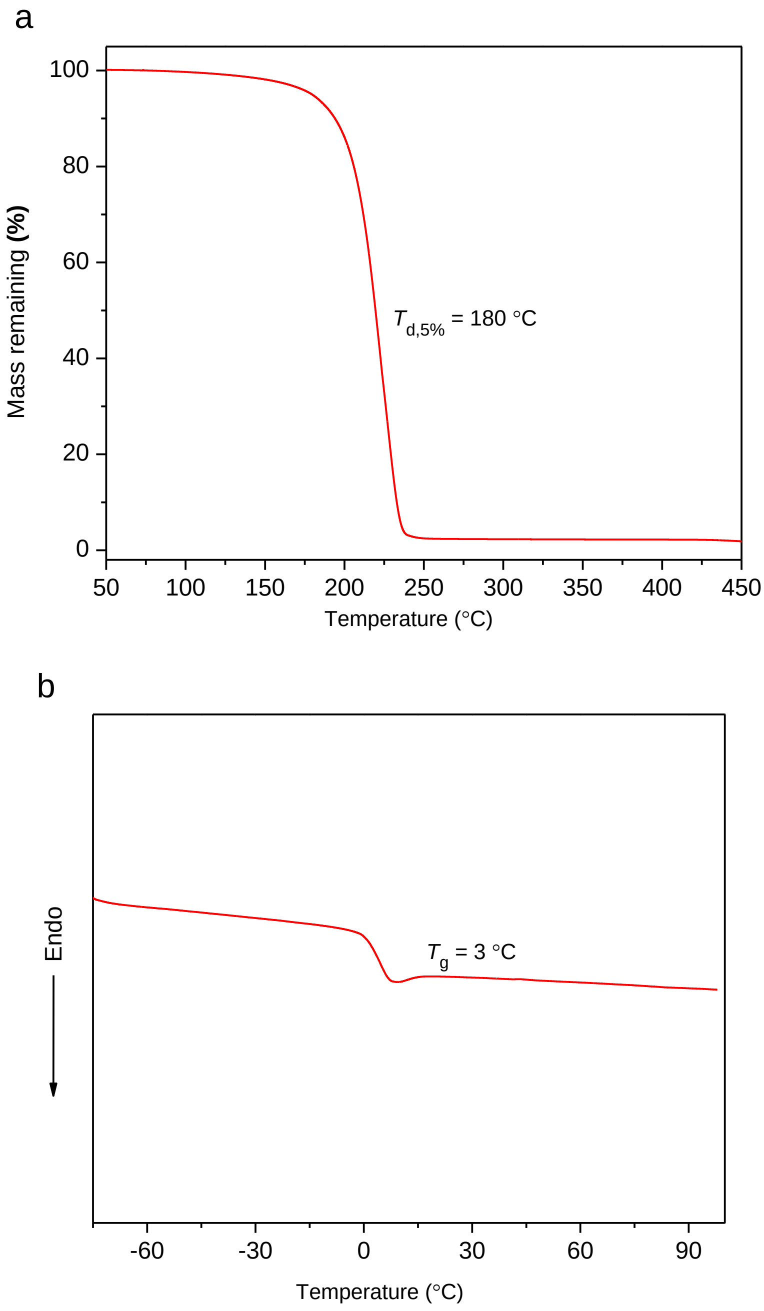

Supplementary Fig. 85 (a) TGA and (b) DSC curves of the obtained copolymer of **P23A**.

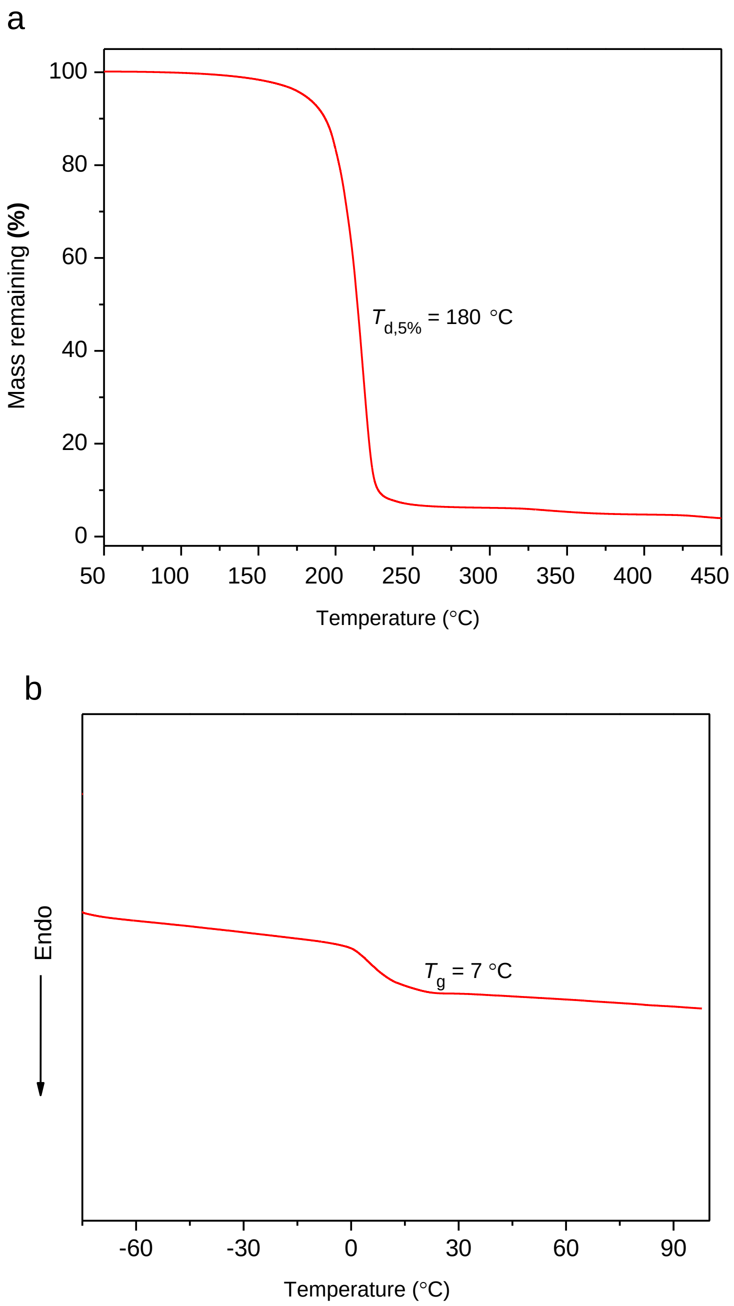

Supplementary Fig. 86 (a) TGA and (b) DSC curves of the obtained copolymer of **P24A**.

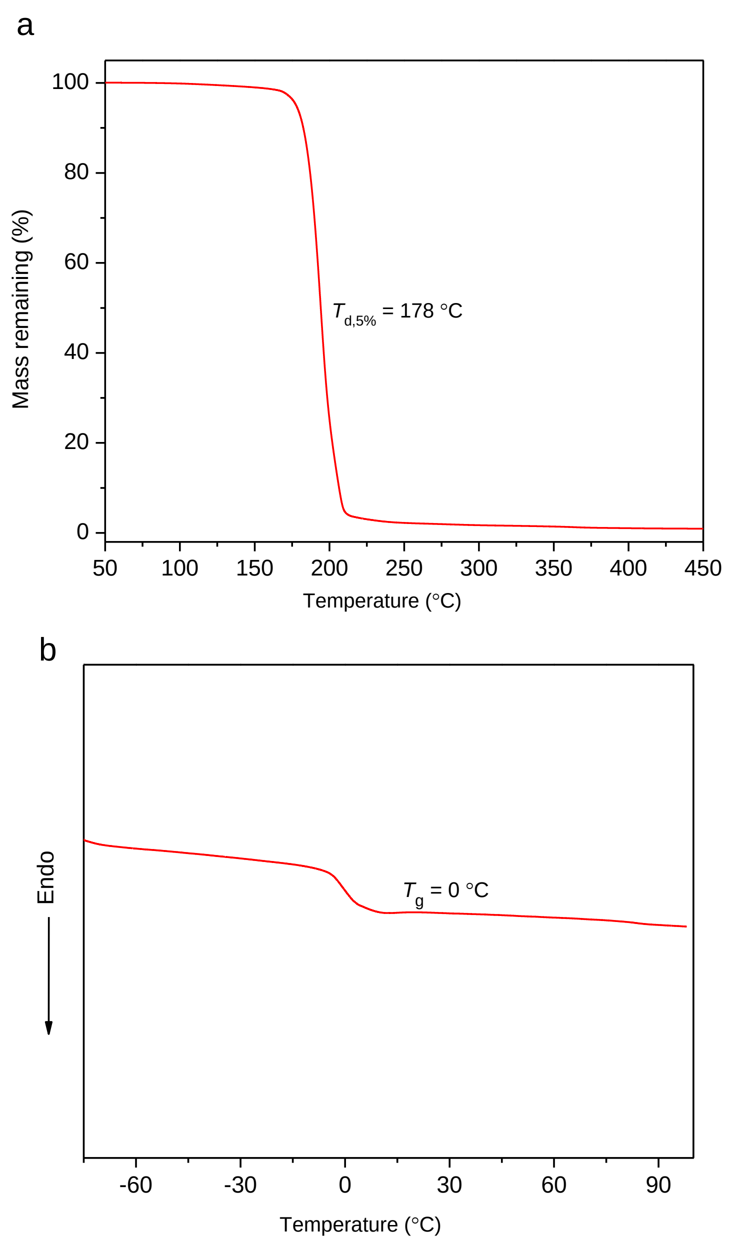

Supplementary Fig. 87 (a) TGA and (b) DSC curves of the obtained copolymer of **P25A**.

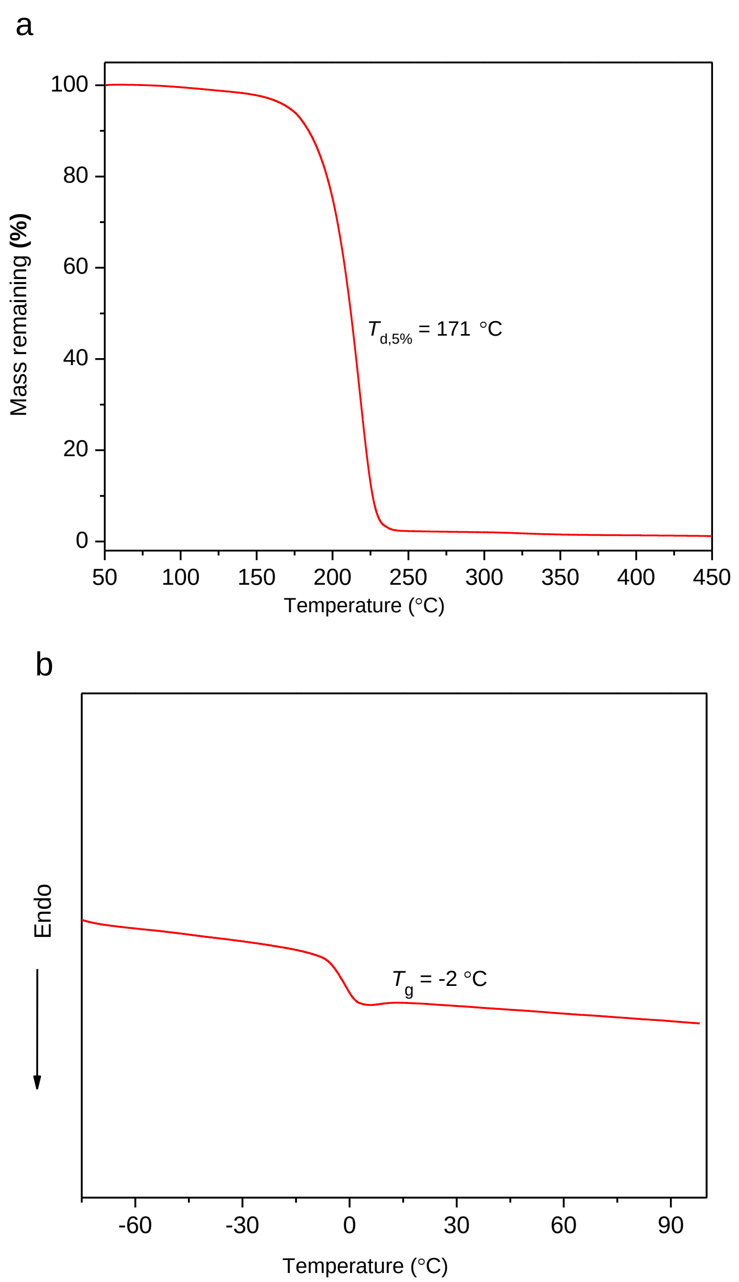

Supplementary Fig. 88 (a) TGA and (b) DSC curves of the obtained copolymer of **P26A**.

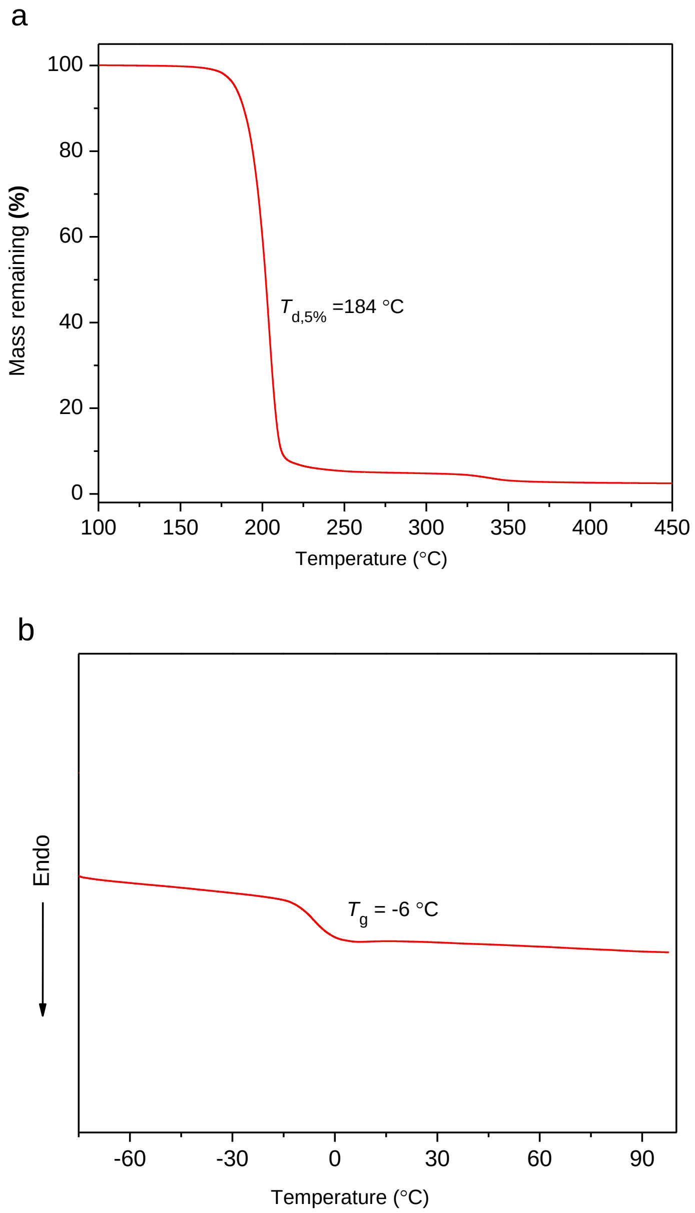

Supplementary Fig. 89 (a) TGA and (b) DSC curves of the obtained copolymer of **P27A**.

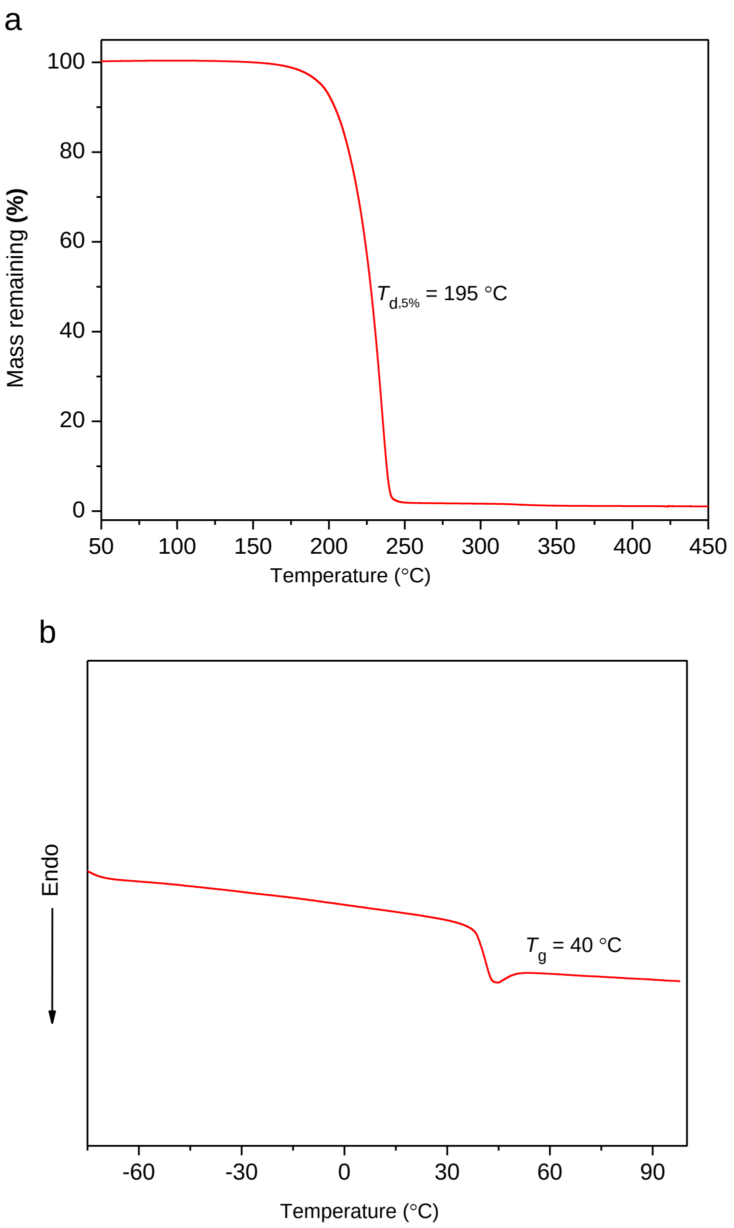

Supplementary Fig. 90 (a) TGA and (b) DSC curves of the obtained copolymer of **P28A**.

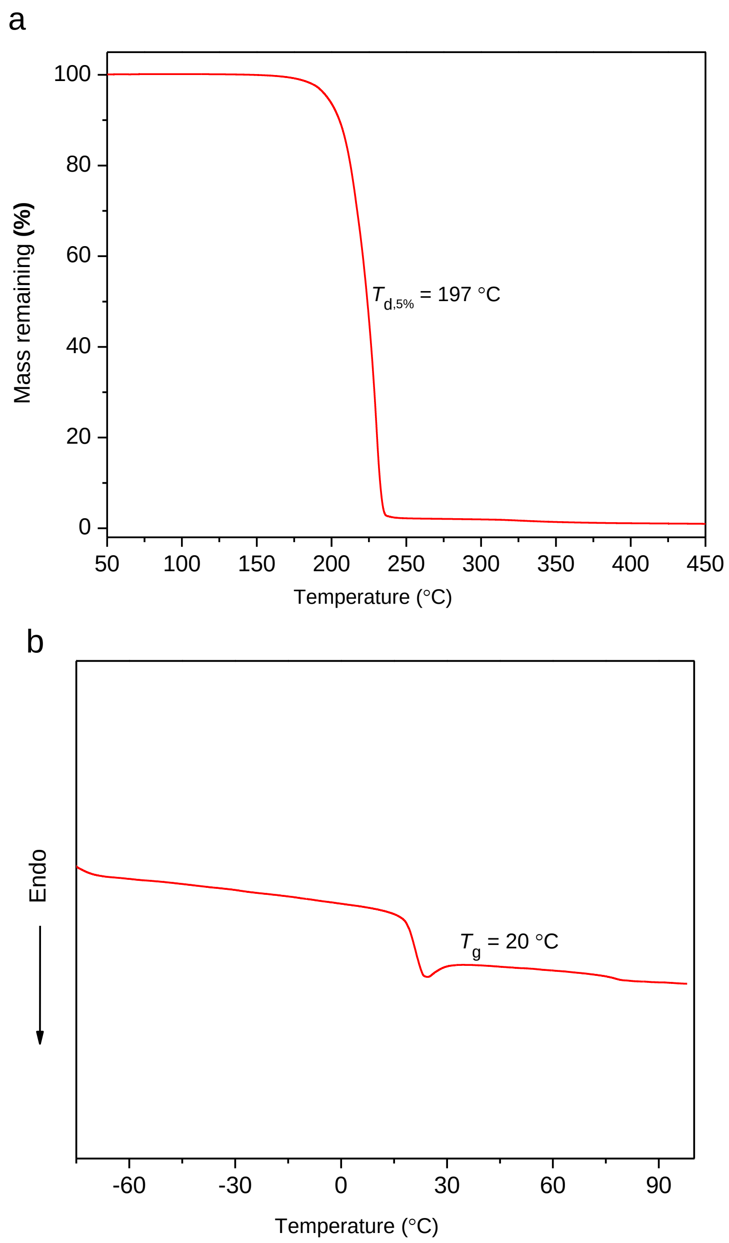

Supplementary Fig. 91 (a) TGA and (b) DSC curves of the obtained copolymer of **P29A**.

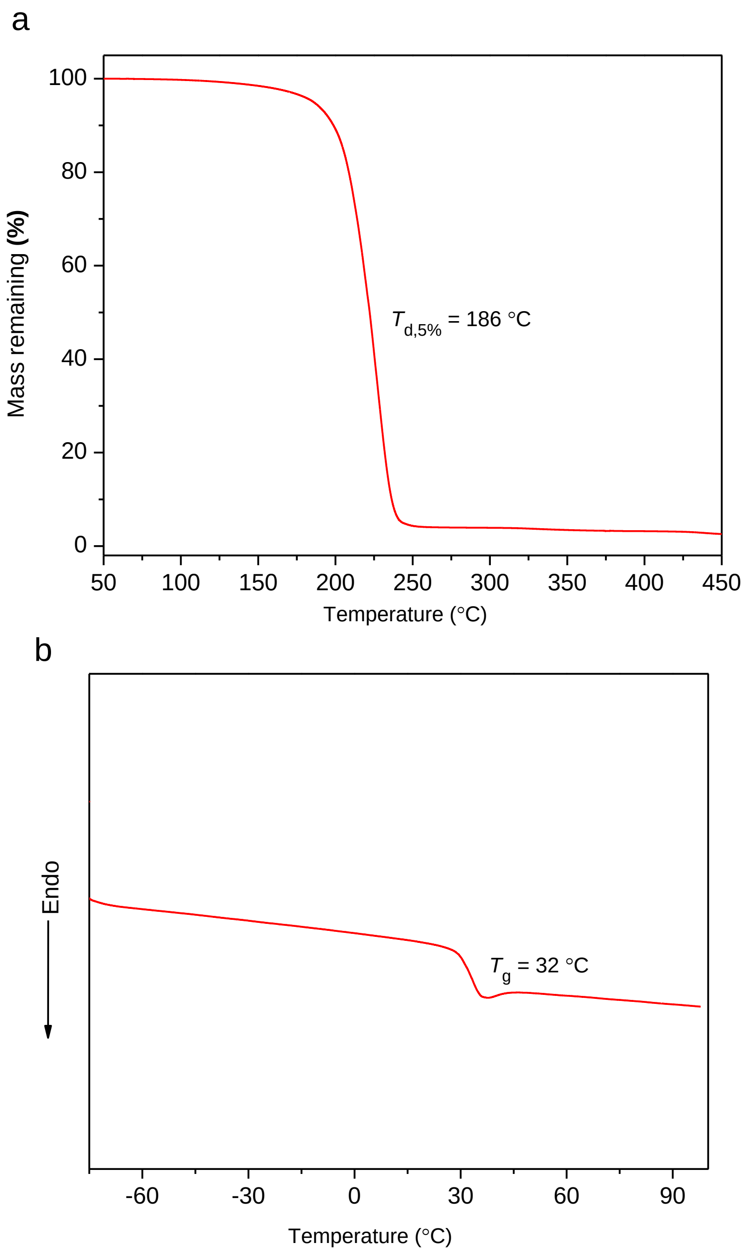

Supplementary Fig. 92 (a) TGA and (b) DSC curves of the obtained copolymer of **P30A**.

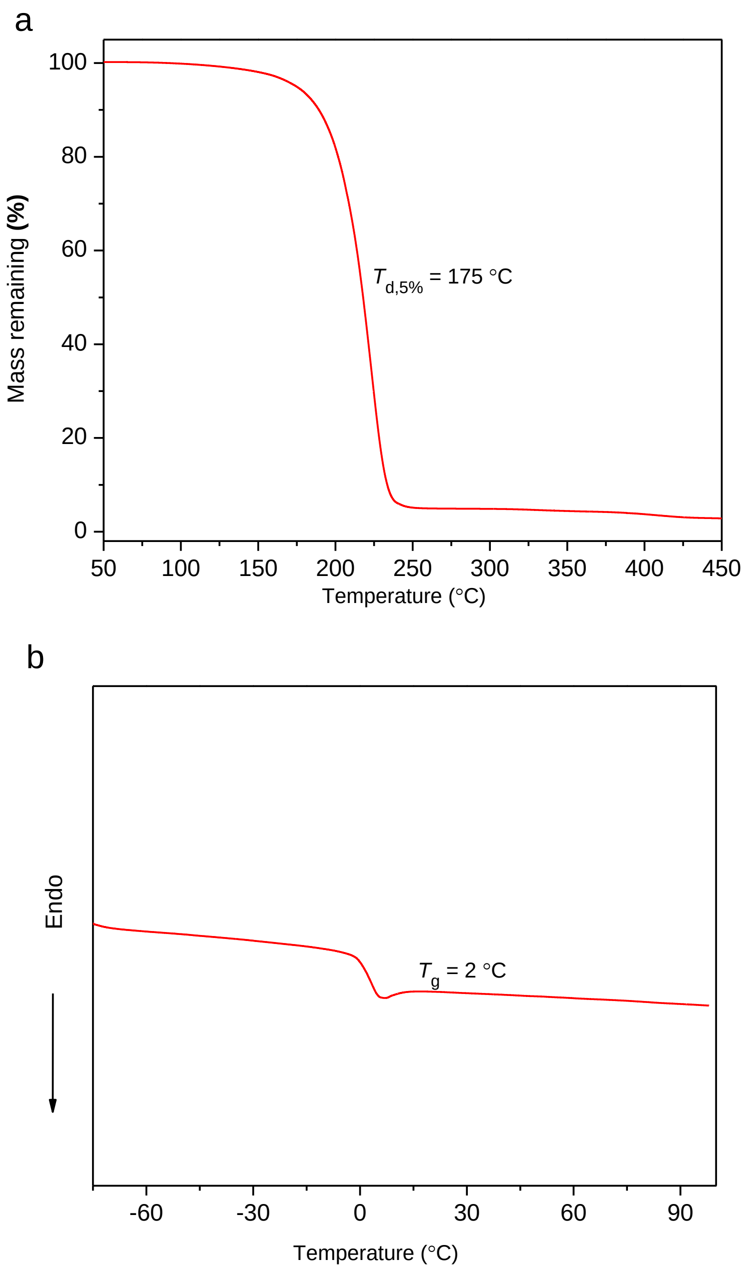

Supplementary Fig. 93 (a) TGA and (b) DSC curves of the obtained copolymer of **P31A**.

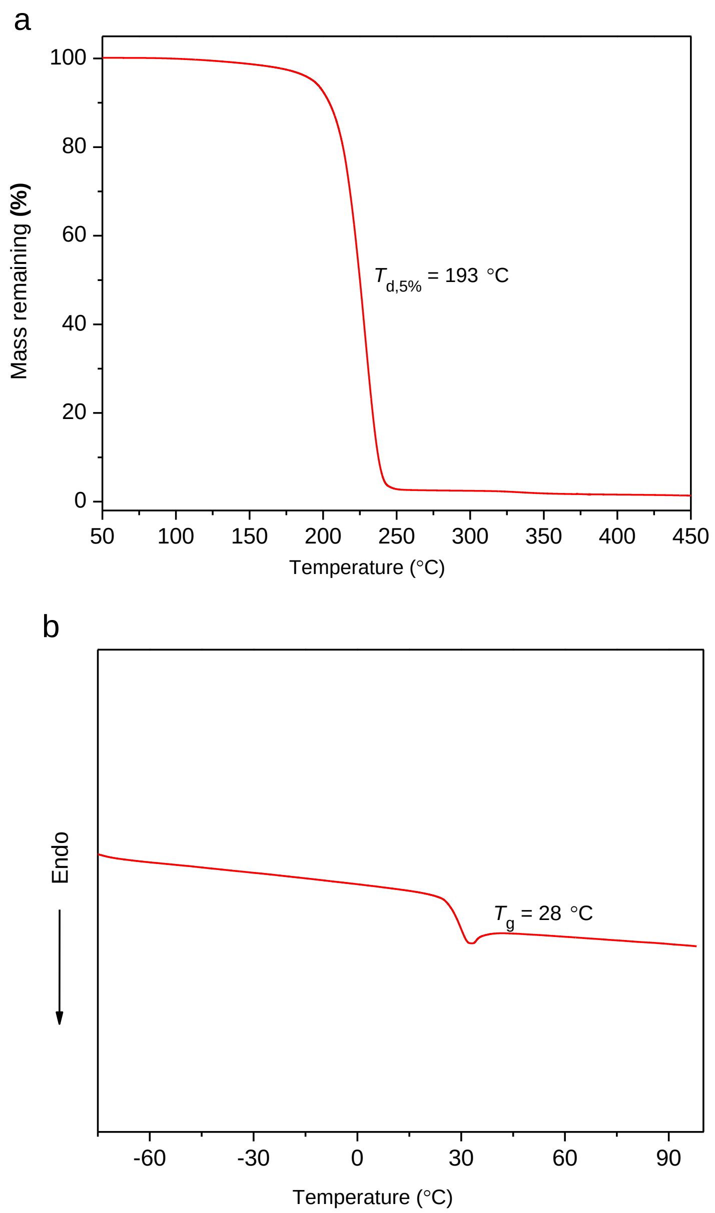

Supplementary Fig. 94 (a) TGA and (b) DSC curves of the obtained copolymer of **P32A**.

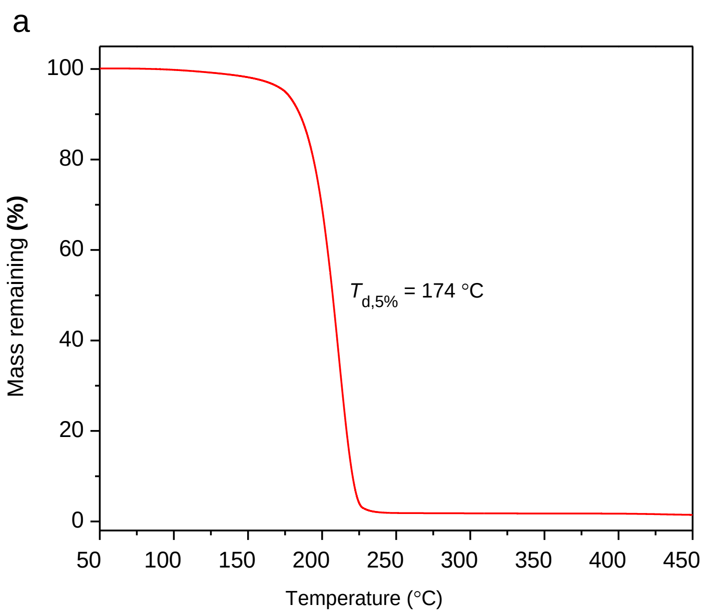

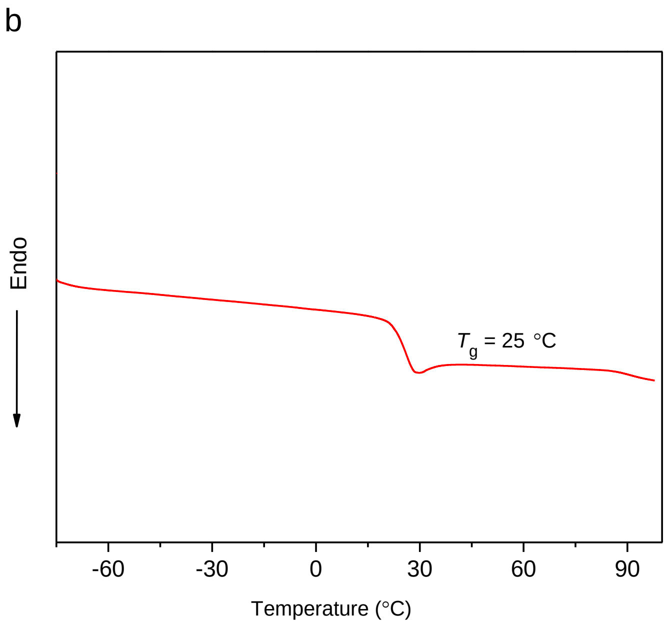

Supplementary Fig. 95 (a) TGA and (b) DSC curves of the obtained copolymer of **P33A**.

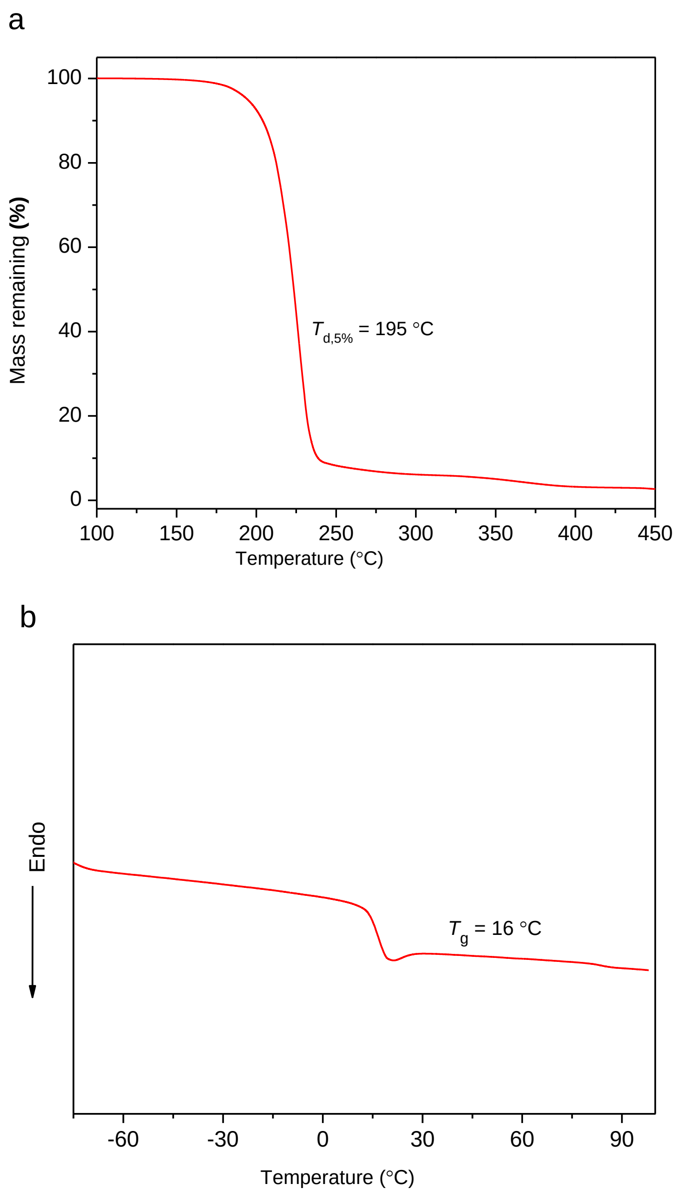

Supplementary Fig. 96 (a) TGA and (b) DSC curves of the obtained copolymer of **P34A**.

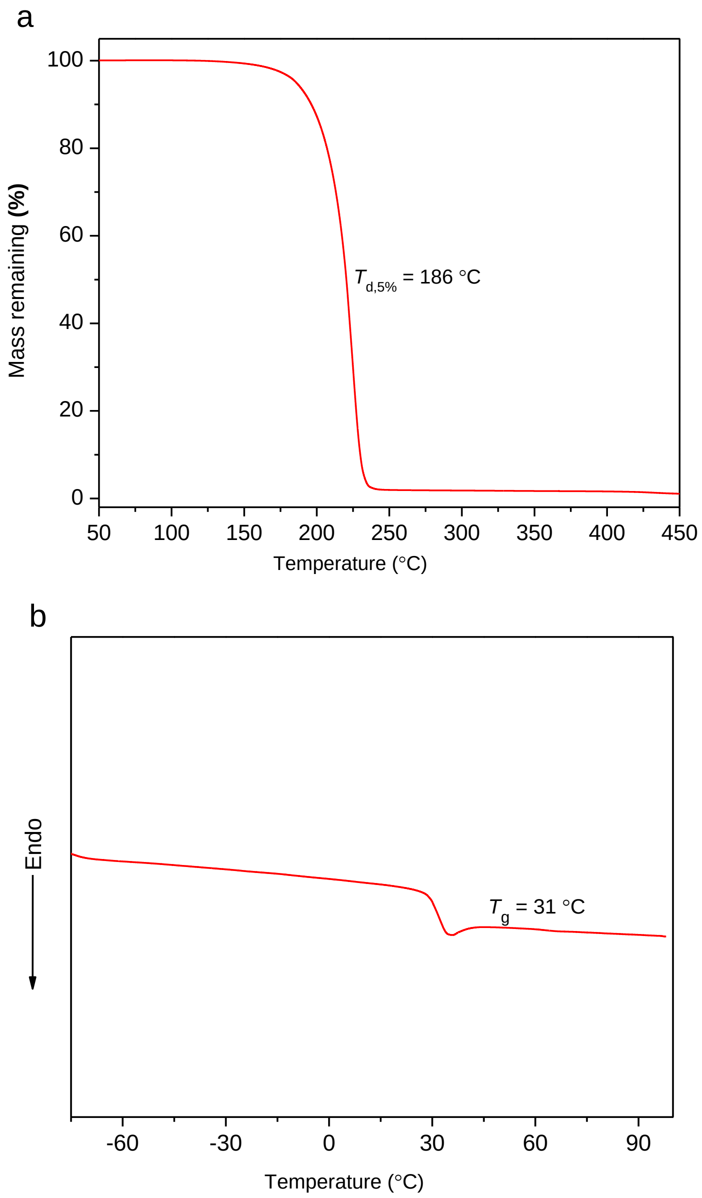

Supplementary Fig. 97 (a) TGA and (b) DSC curves of the obtained copolymer of **P35A**.

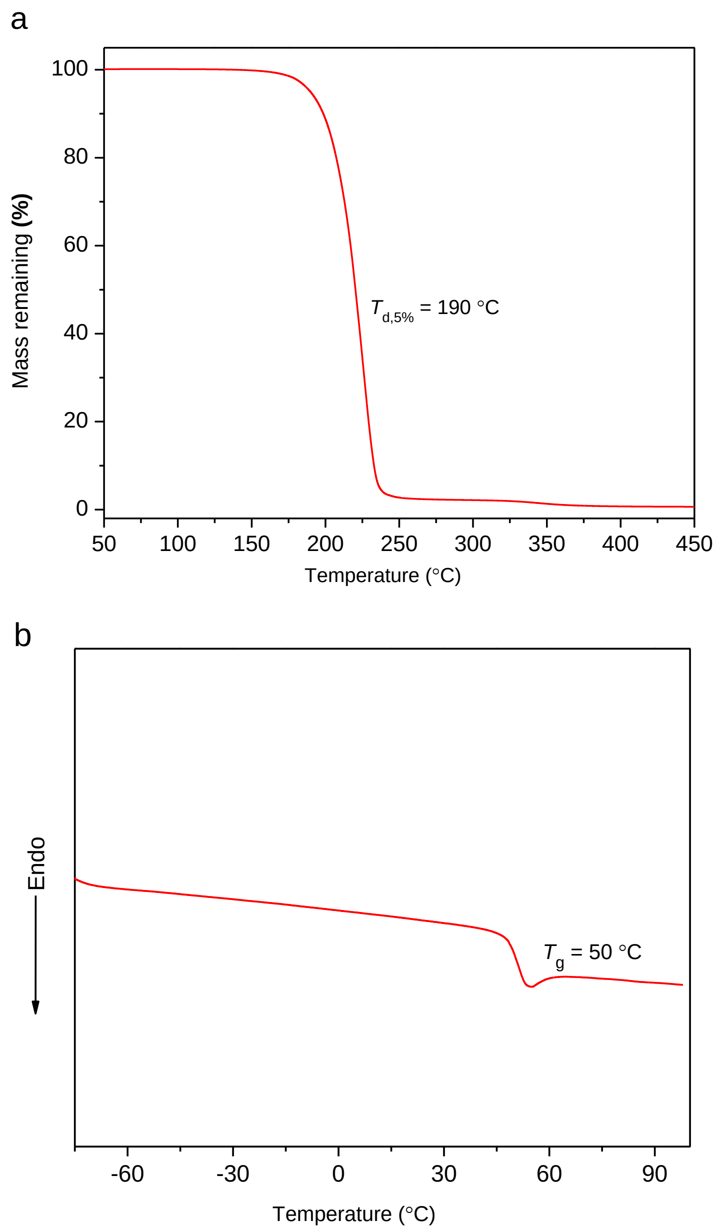

Supplementary Fig. 98 (a) TGA and (b) DSC curves of the obtained copolymer of **P36A**.

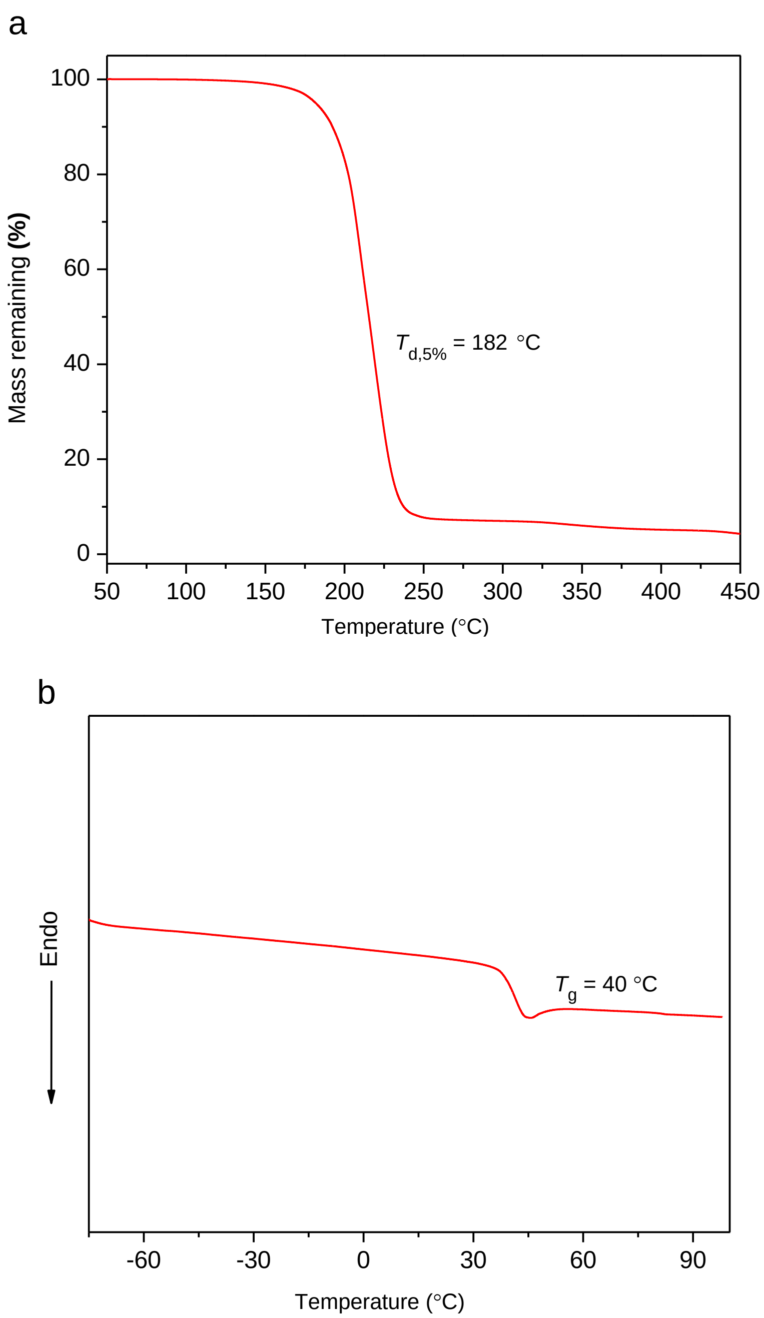

Supplementary Fig. 99 (a) TGA and (b) DSC curves of the obtained copolymer of **P37A**.

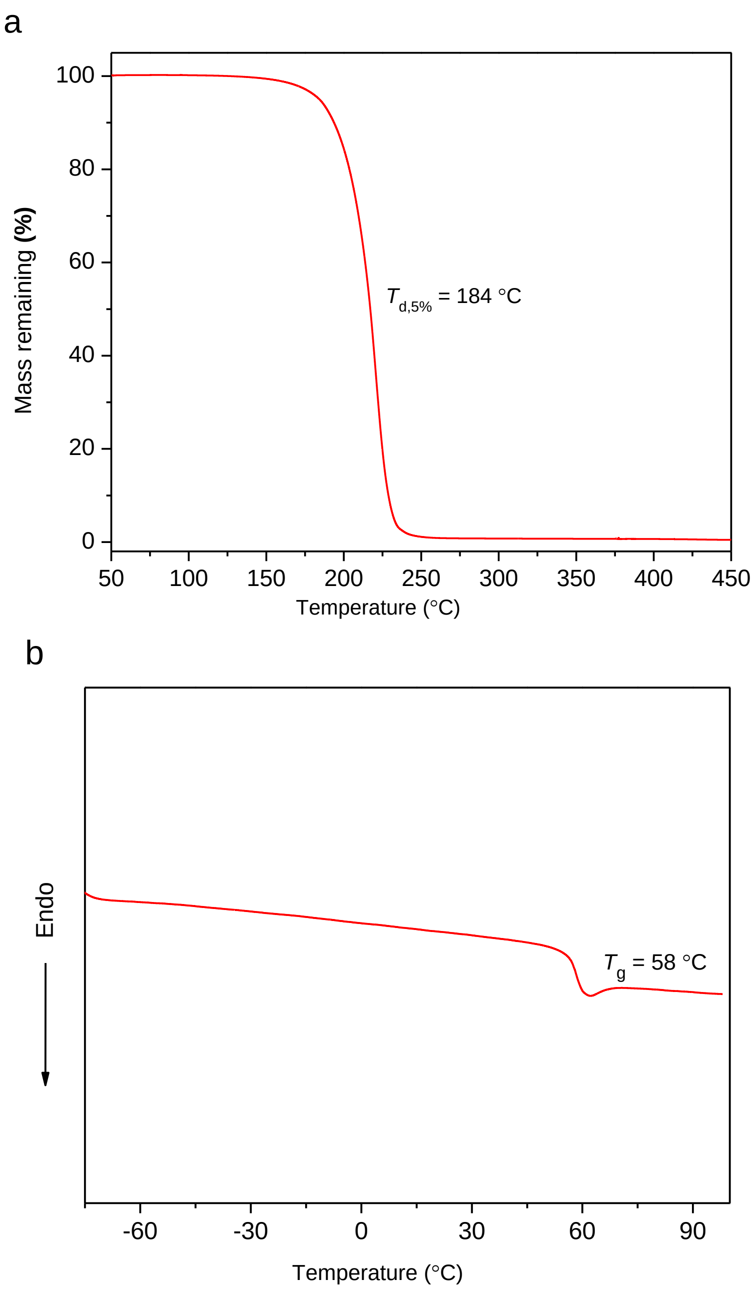

Supplementary Fig. 100 (a) TGA and (b) DSC curves of the obtained copolymer of **P38A**.

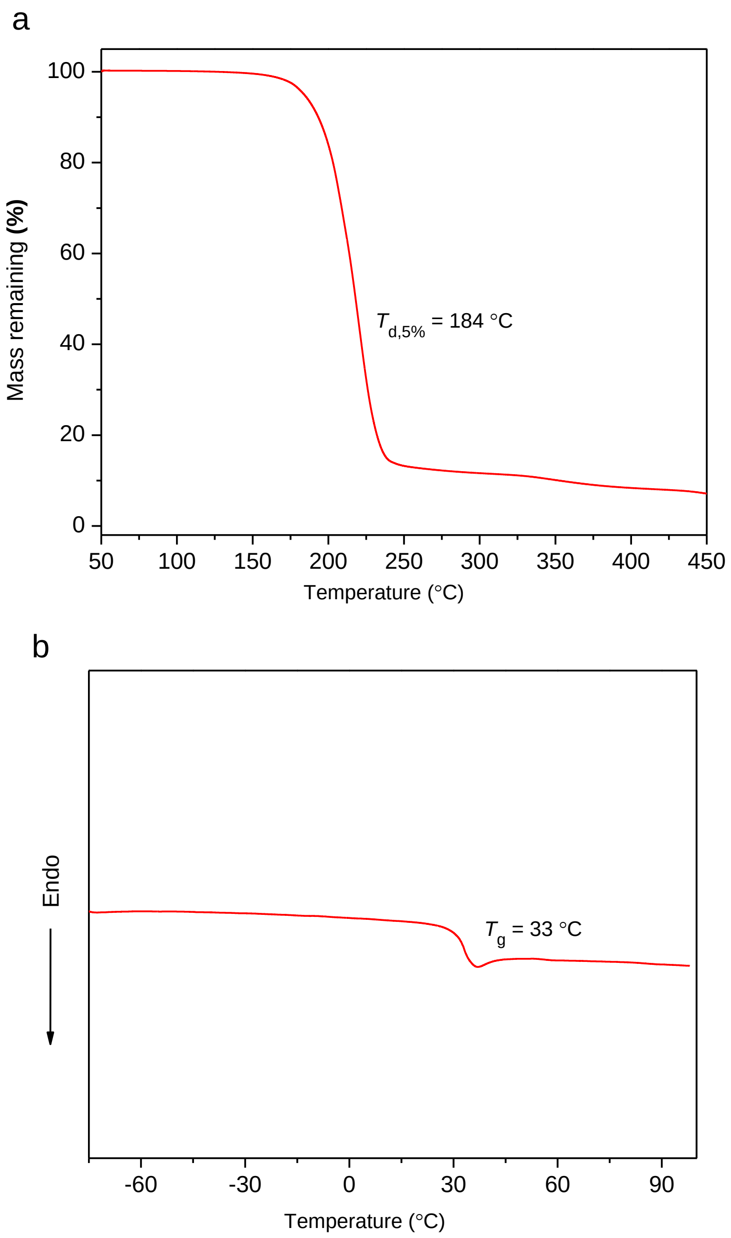

Supplementary Fig. 101 (a) TGA and (b) DSC curves of the obtained copolymer of **P39A**.

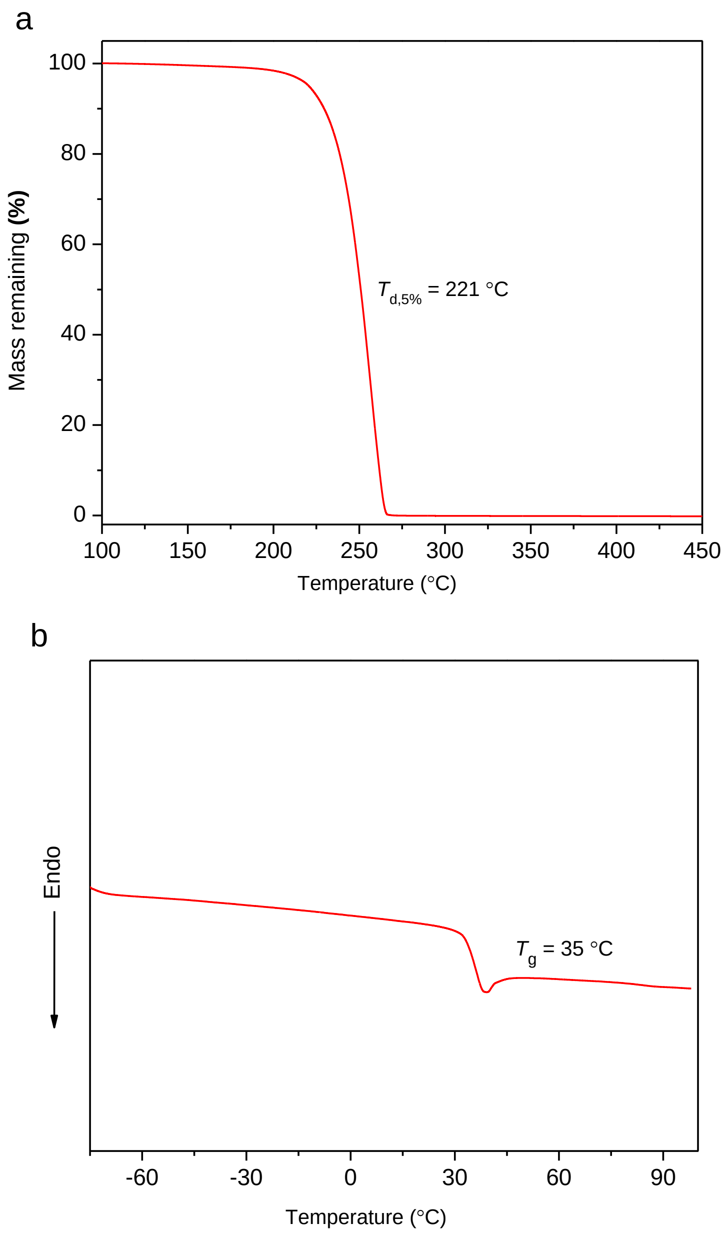

Supplementary Fig. 102 (a) TGA and (b) DSC curves of the obtained copolymer of **P40A**.

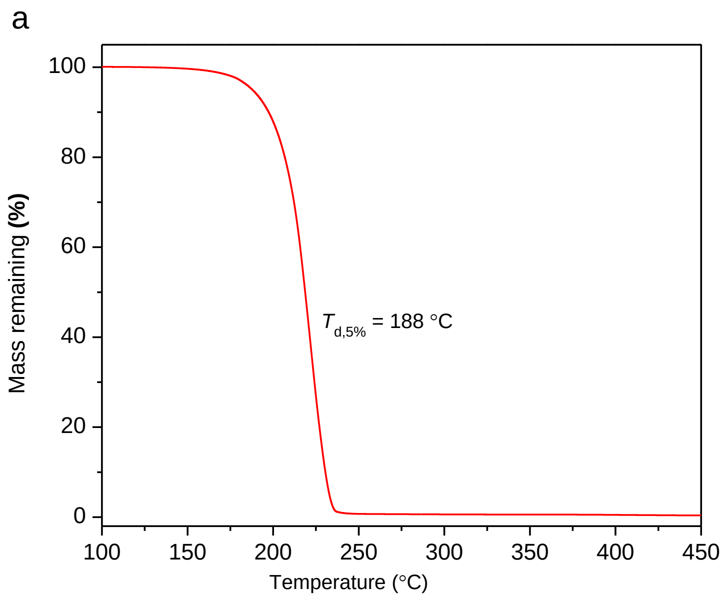

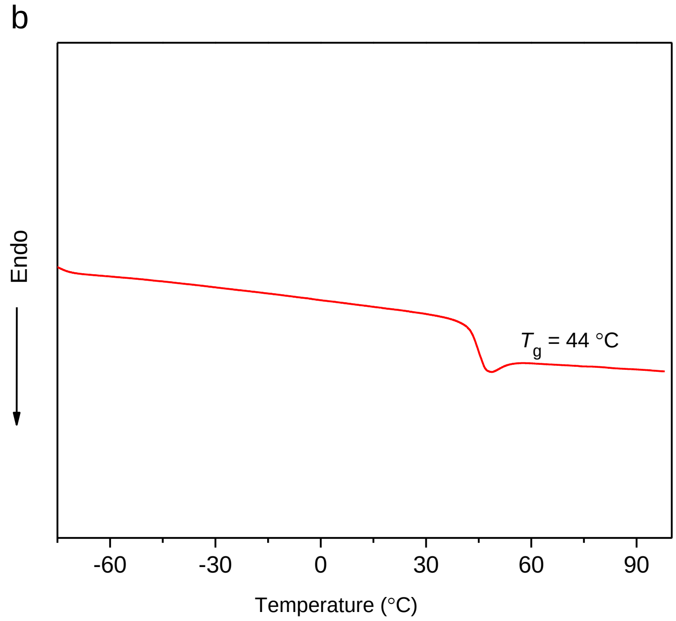

Supplementary Fig. 103 (a) TGA and (b) DSC curves of the obtained copolymer of **P41A**.

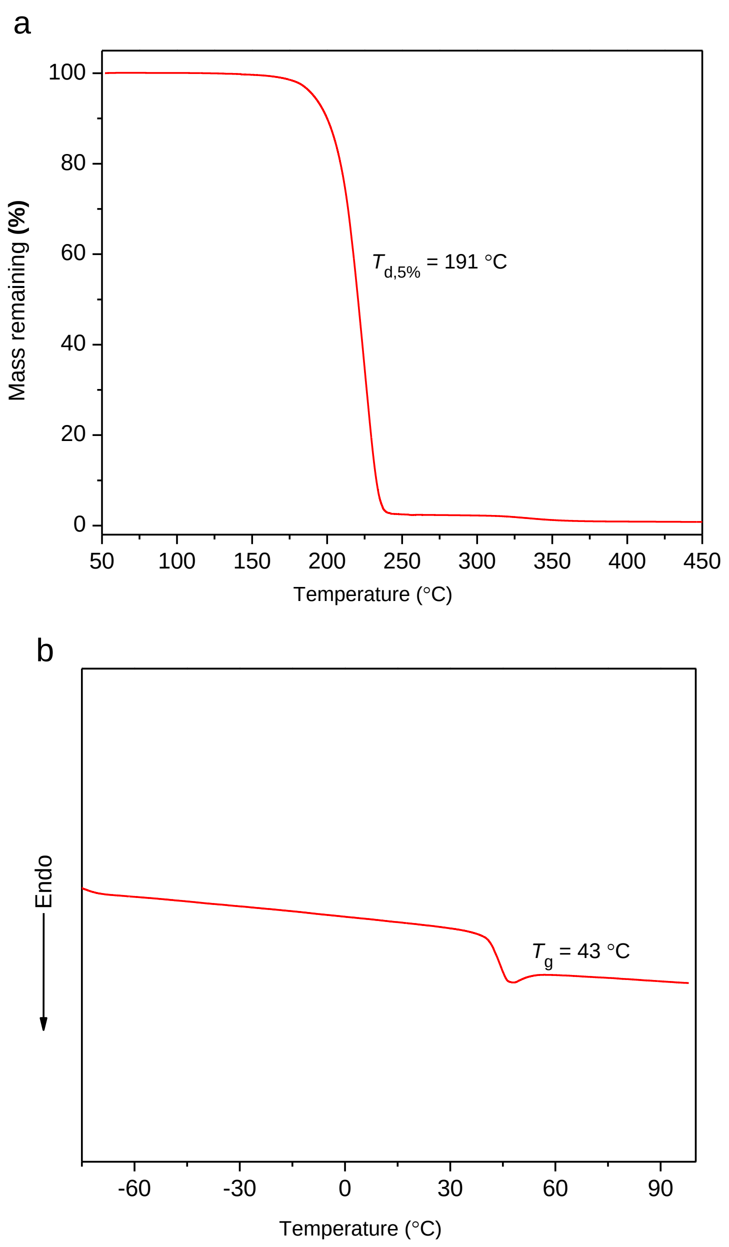

Supplementary Fig. 104 (a) TGA and (b) DSC curves of the obtained copolymer of **P42A**.

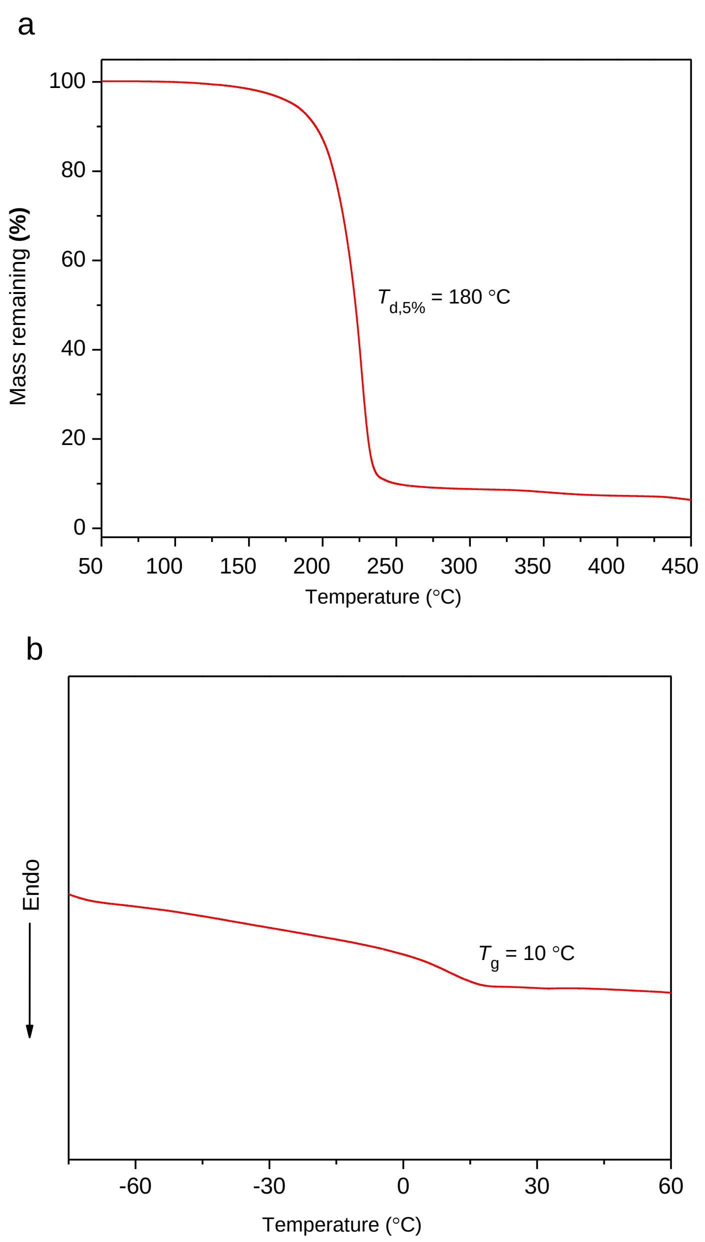

Supplementary Fig. 105 (a) TGA and (b) DSC curves of the obtained copolymer of **P43A**.

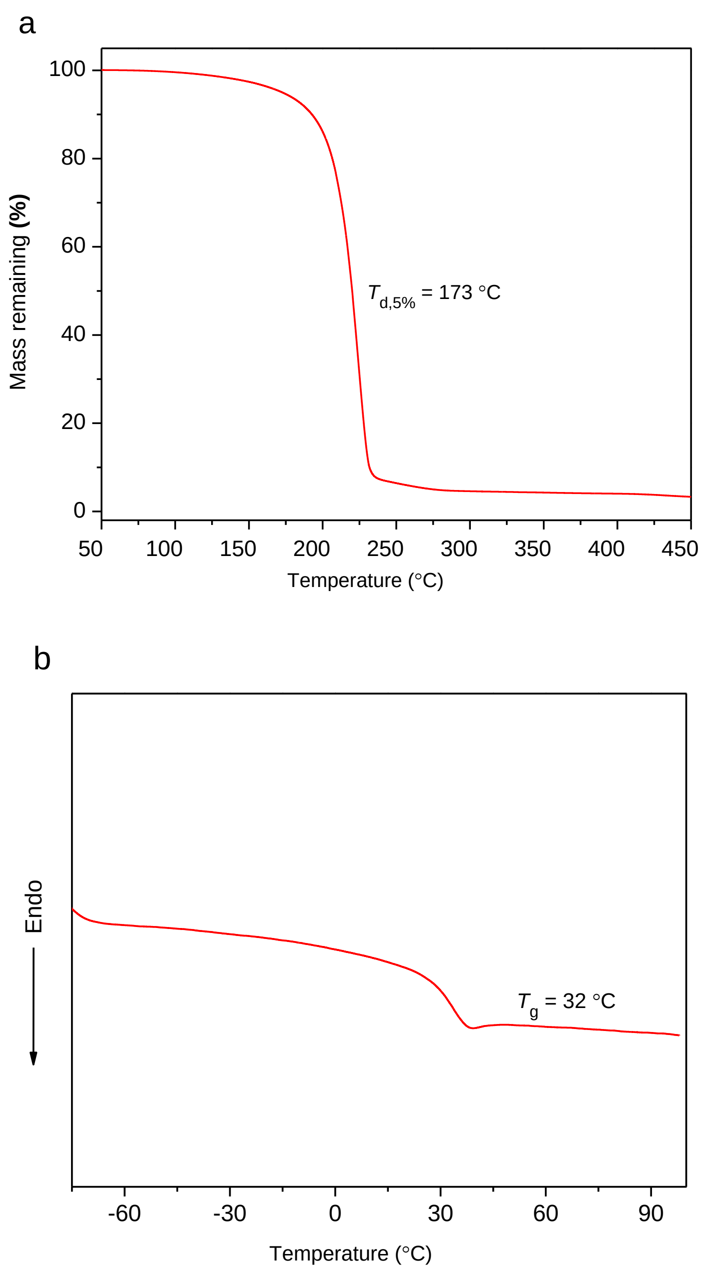

Supplementary Fig. 106 (a) TGA and (b) DSC curves of the obtained copolymer of **P44A**.

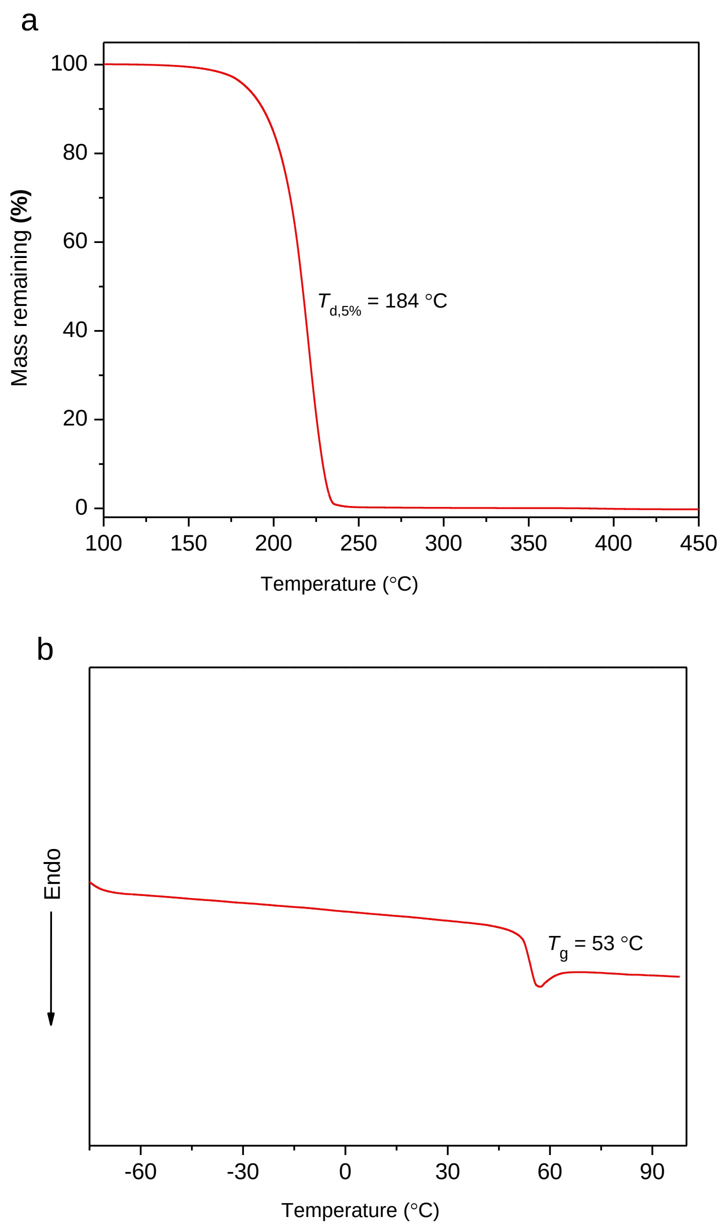

Supplementary Fig. 107 (a) TGA and (b) DSC curves of the obtained copolymer of **P45A**.

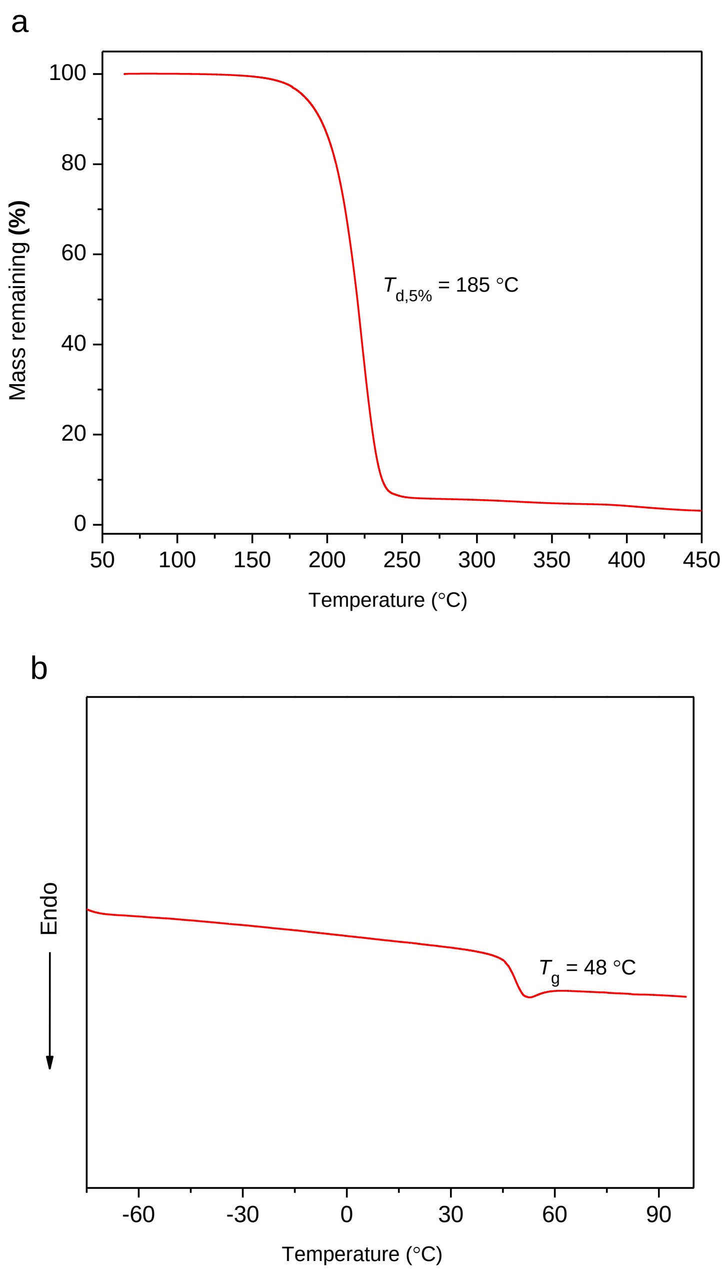

Supplementary Fig. 108 (a) TGA and (b) DSC curves of the obtained copolymer of **P46A**.

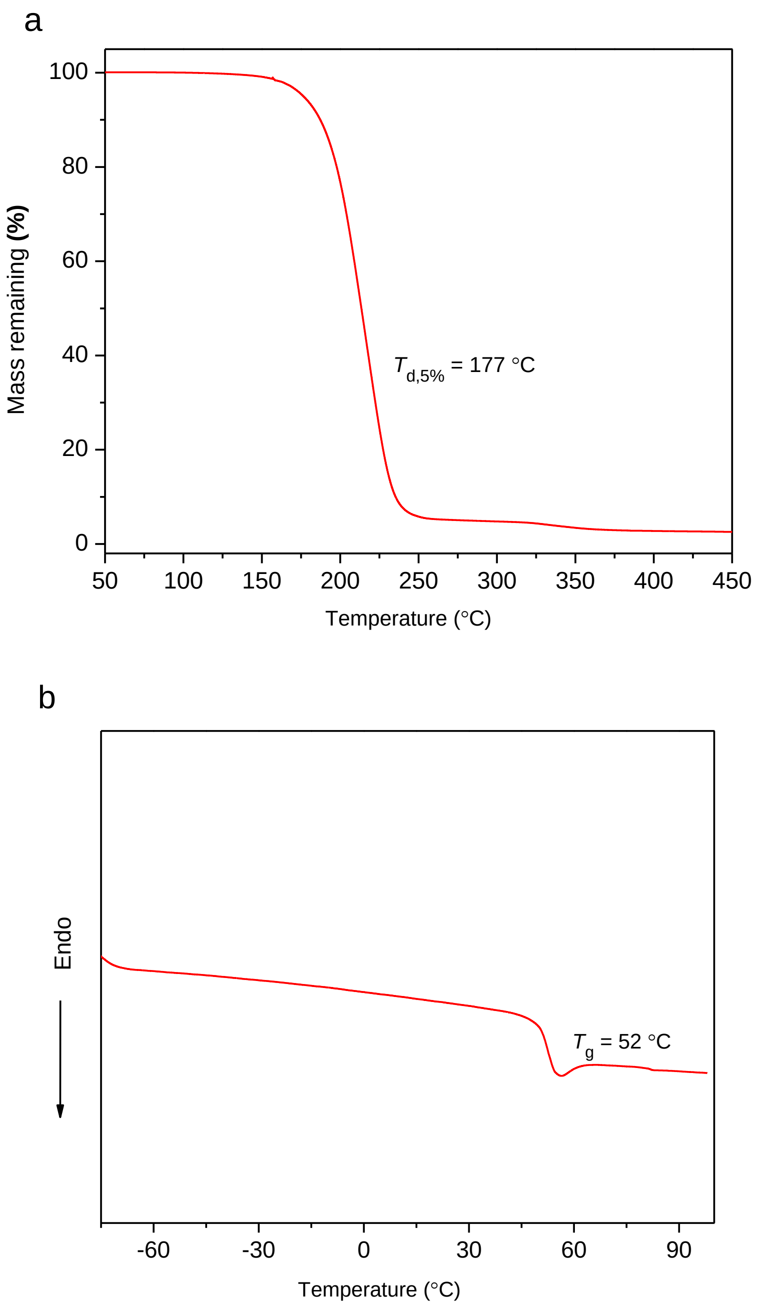

Supplementary Fig. 109 (a) TGA and (b) DSC curves of the obtained copolymer of **P47A**.

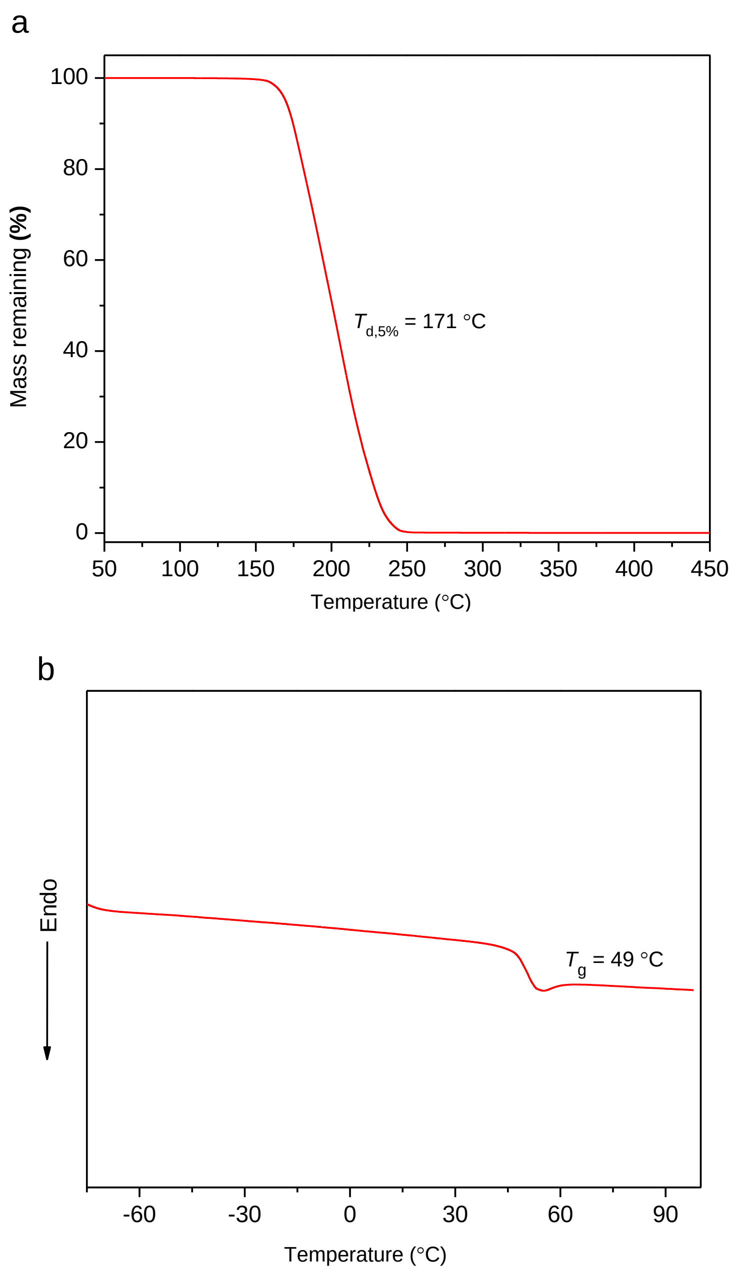

Supplementary Fig. 110 (a) TGA and (b) DSC curves of the obtained copolymer of **P48A**.

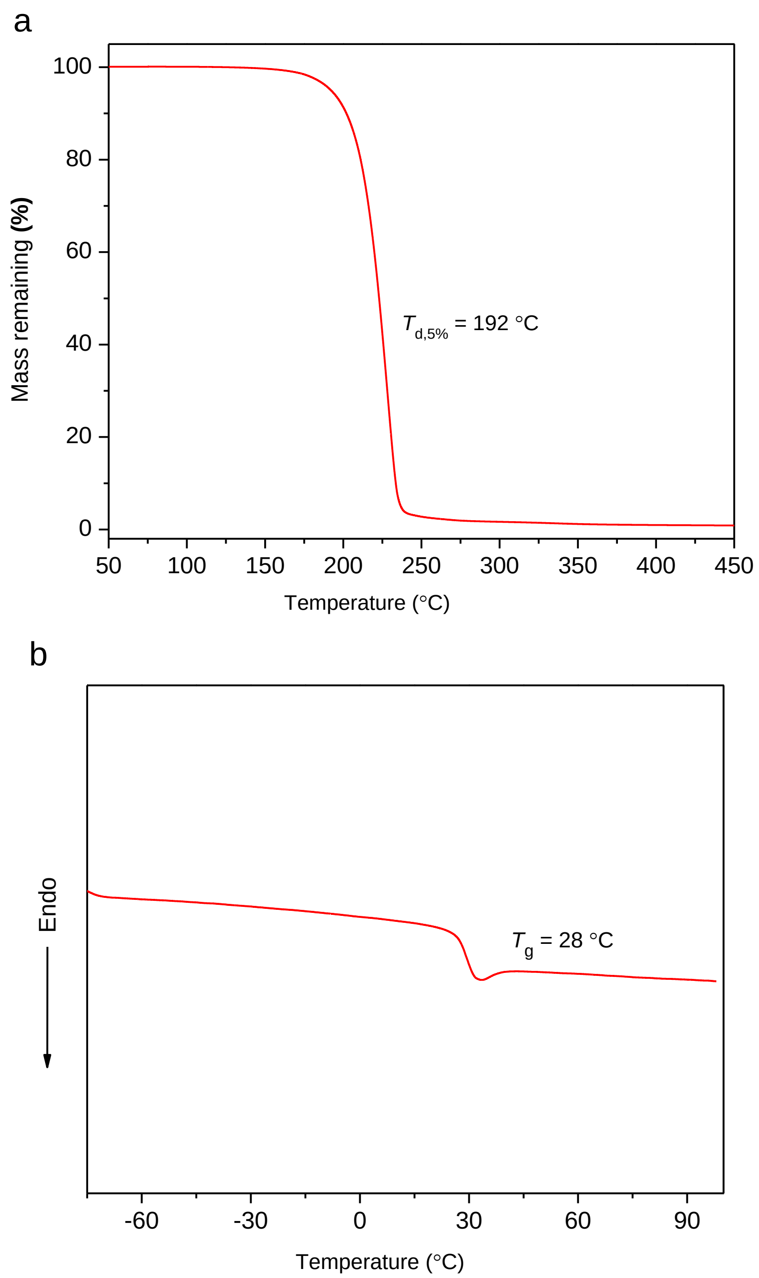

Supplementary Fig. 111 (a) TGA and (b) DSC curves of the obtained copolymer of **P49A**.

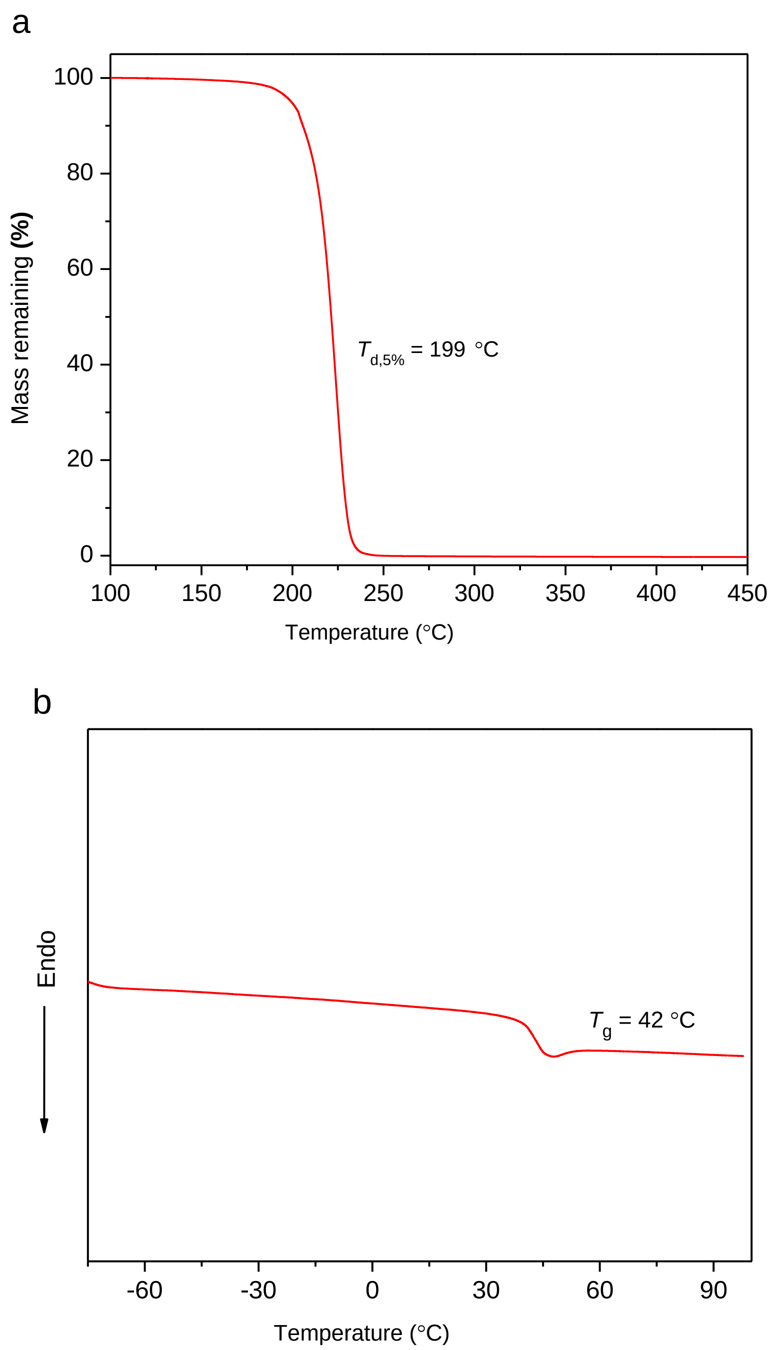

Supplementary Fig. 112 (a) TGA and (b) DSC curves of the obtained copolymer of **P50A**.

a

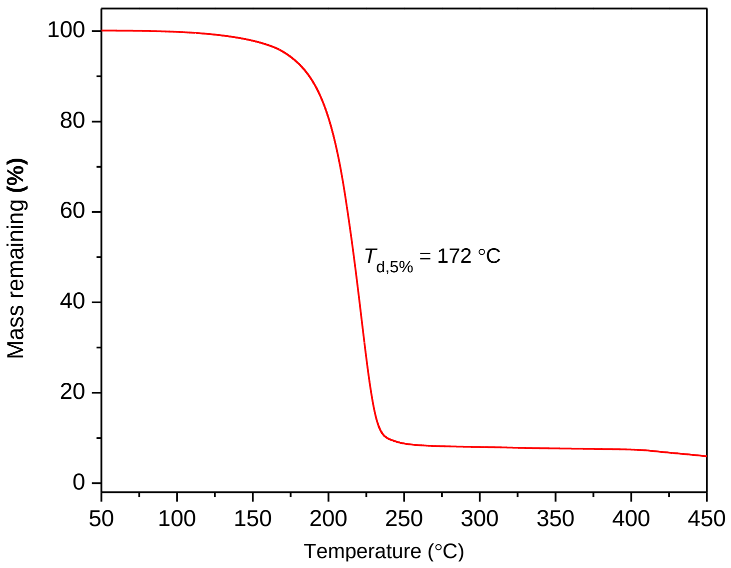

b

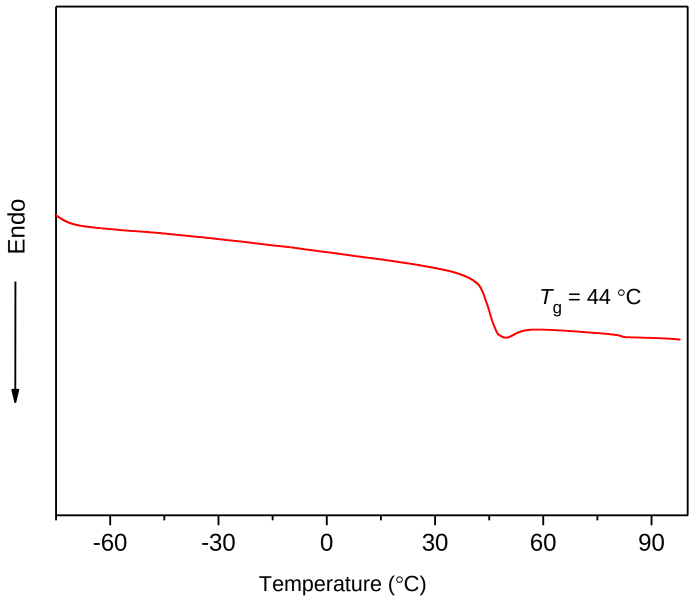

Supplementary Fig. 113 (a) TGA and (b) DSC curves of the obtained copolymer of **P51A**.

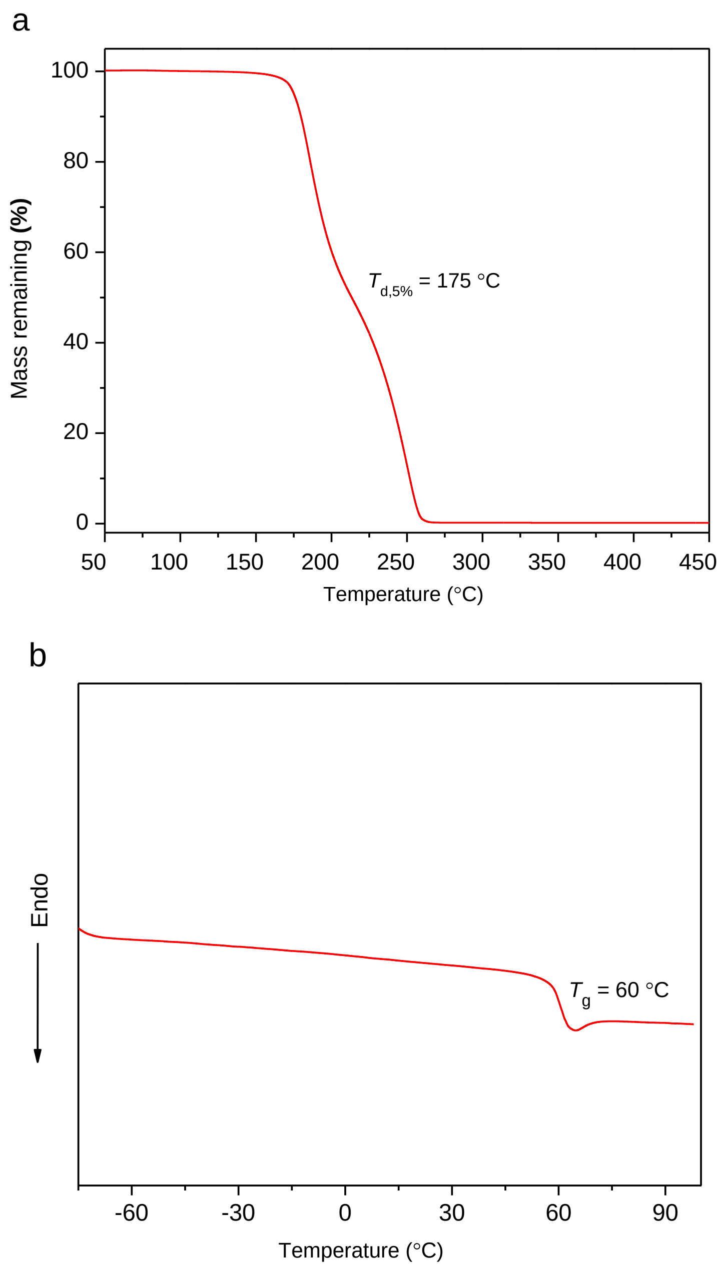

Supplementary Fig. 114 (a) TGA and (b) DSC curves of the obtained copolymer of **P52A**.

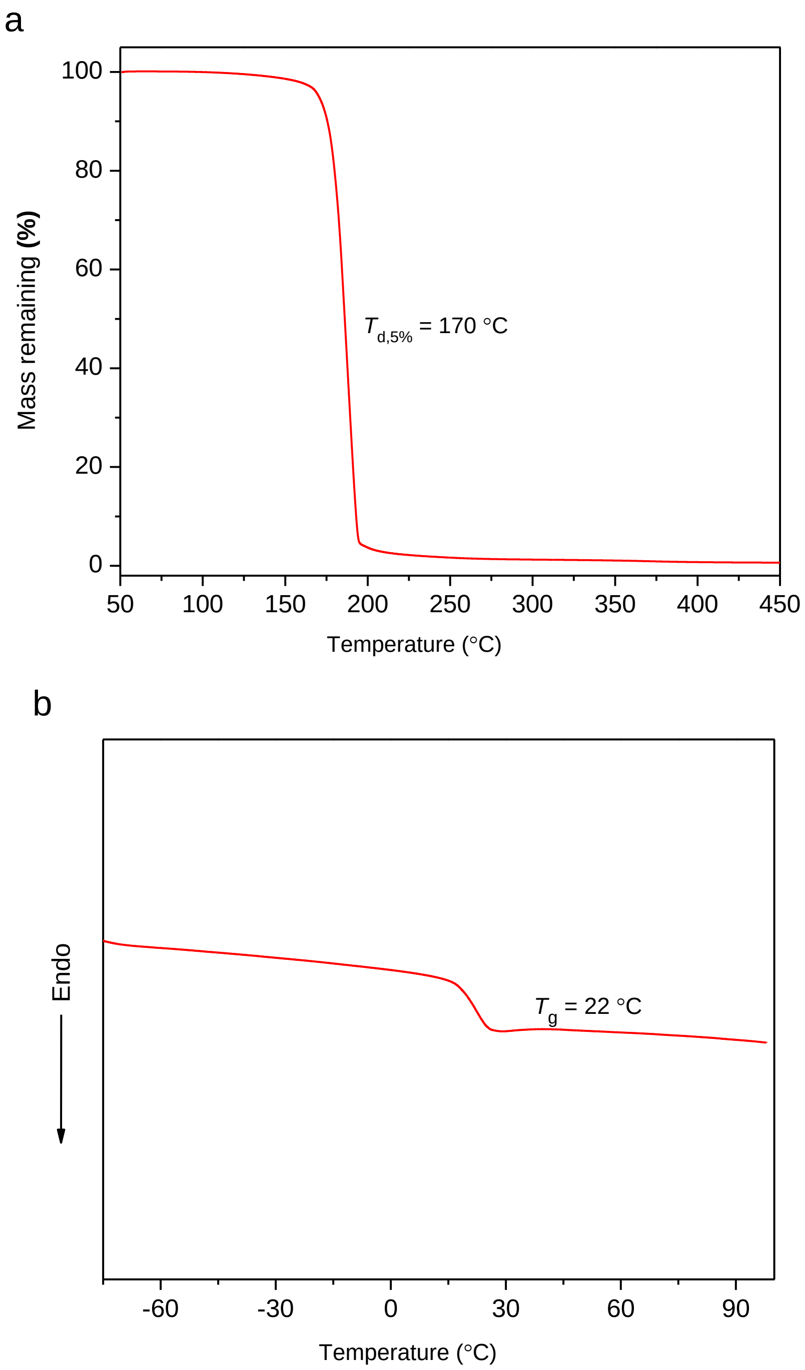

Supplementary Fig. 115 (a) TGA and (b) DSC curves of the obtained copolymer of **P53A**.

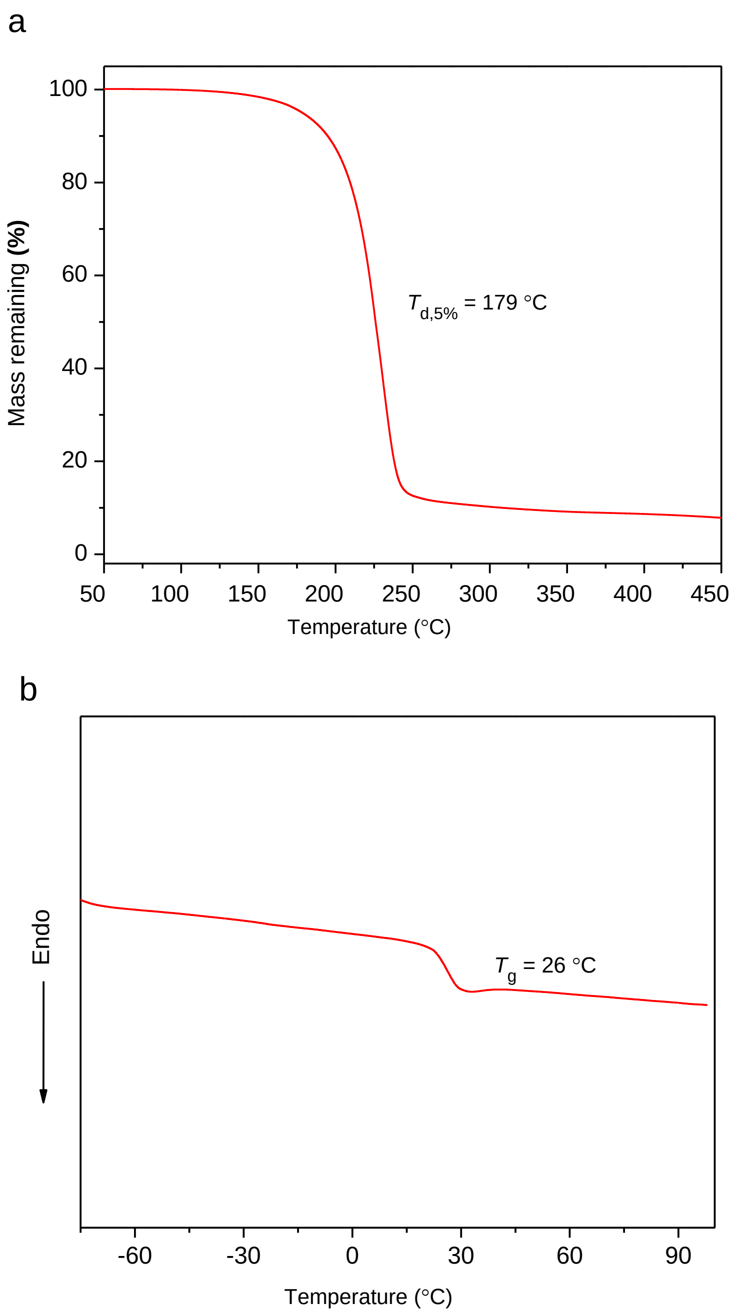

Supplementary Fig. 116 (a) TGA and (b) DSC curves of the obtained copolymer of **P54A**.

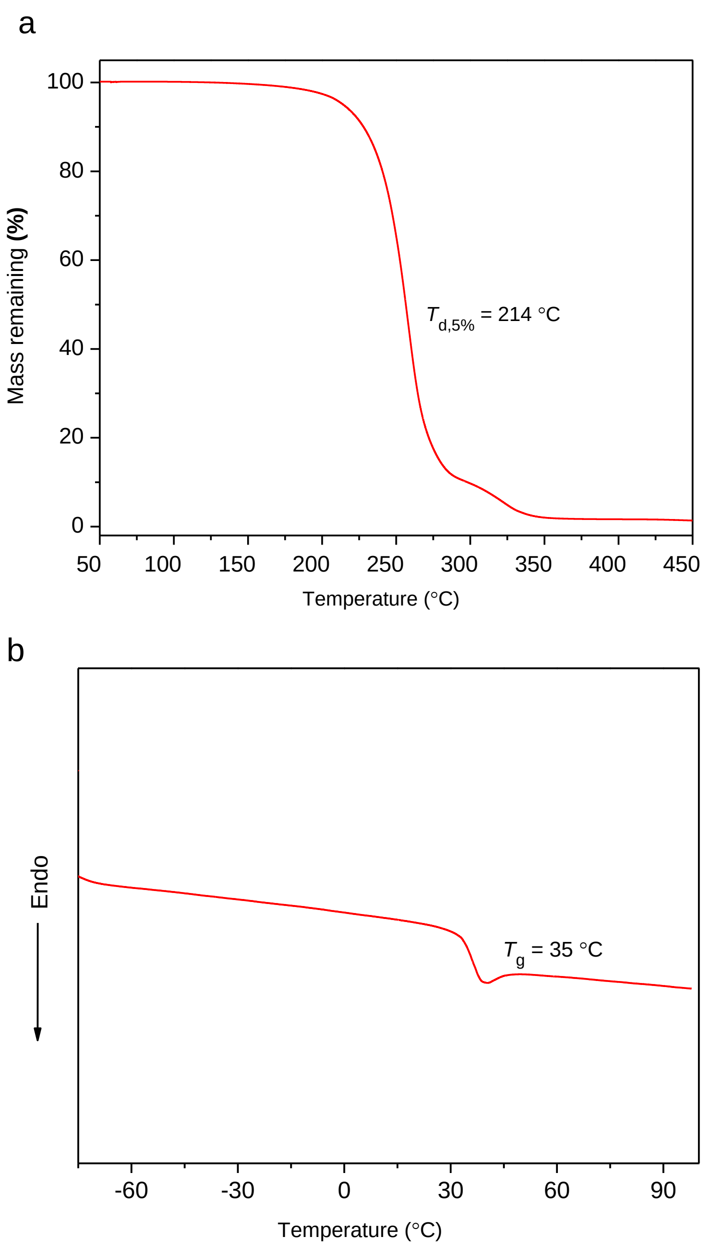

Supplementary Fig. 117 (a) TGA and (b) DSC curves of the obtained copolymer of **P55A**.

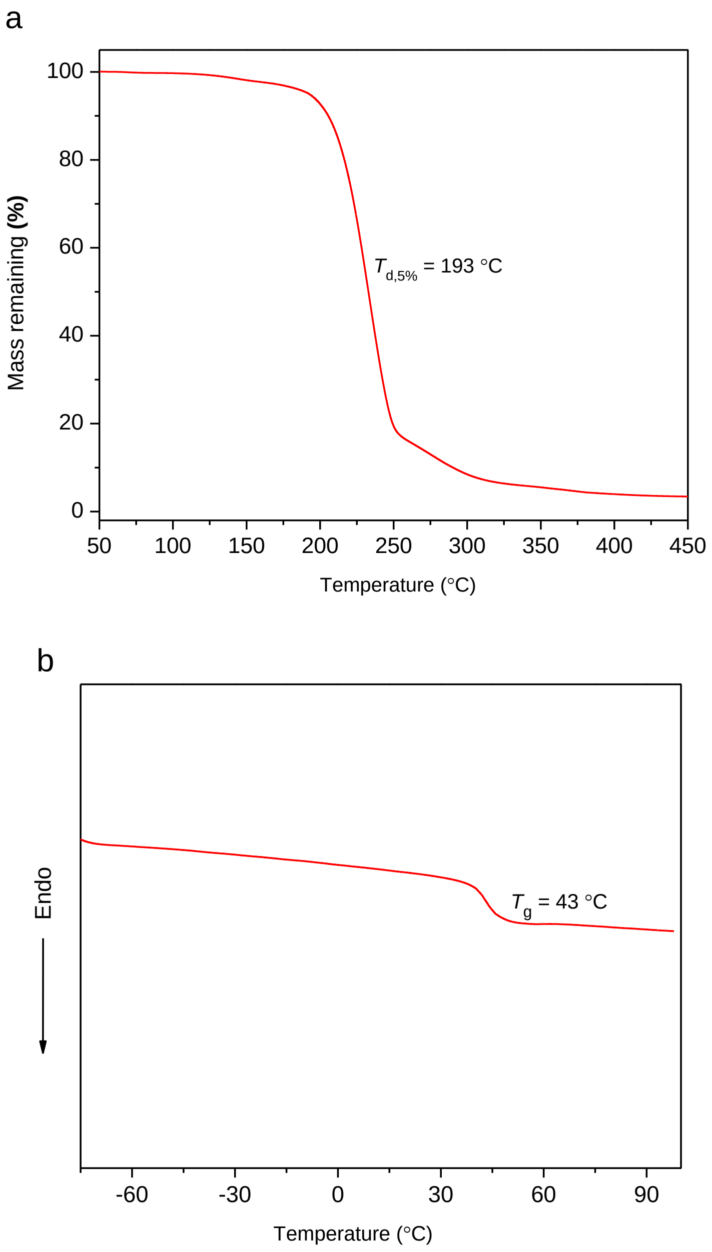

Supplementary Fig. 118 (a) TGA and (b) DSC curves of the obtained copolymer of **P56A**.

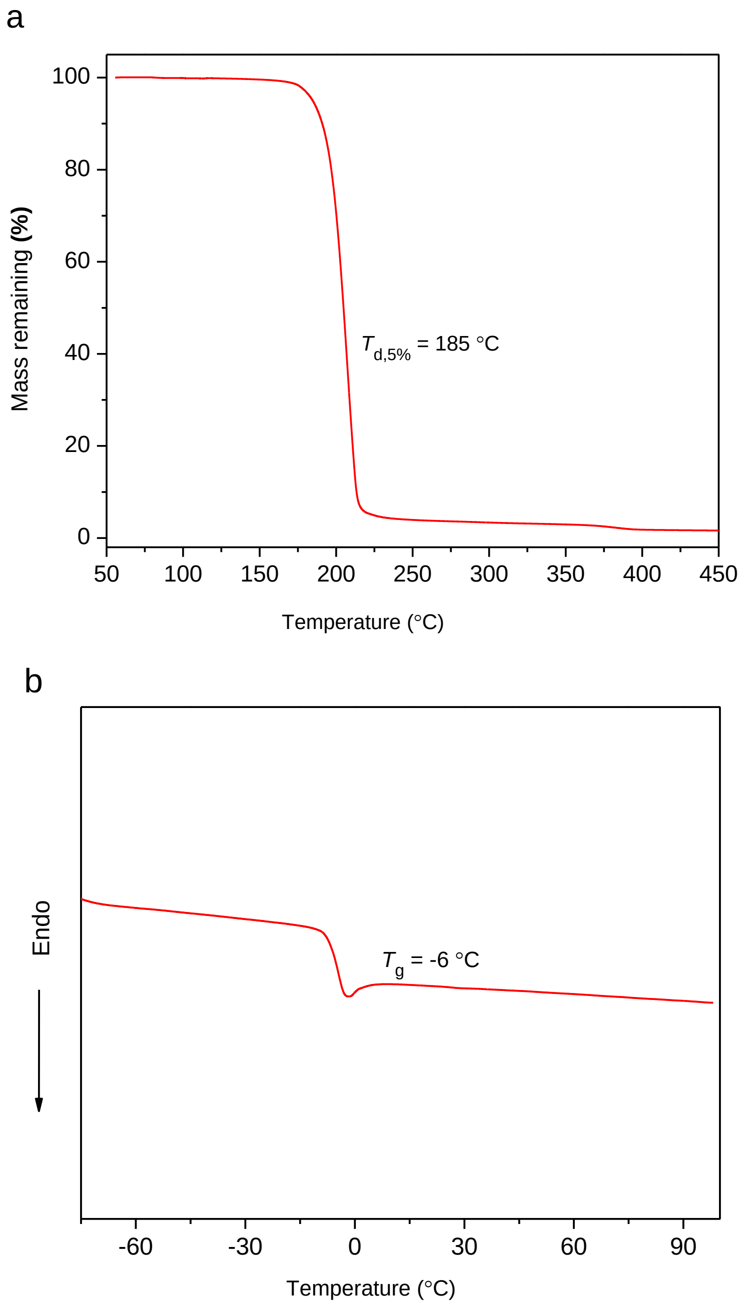

Supplementary Fig. 119 (a) TGA and (b) DSC curves of the obtained copolymer of **P1B**.

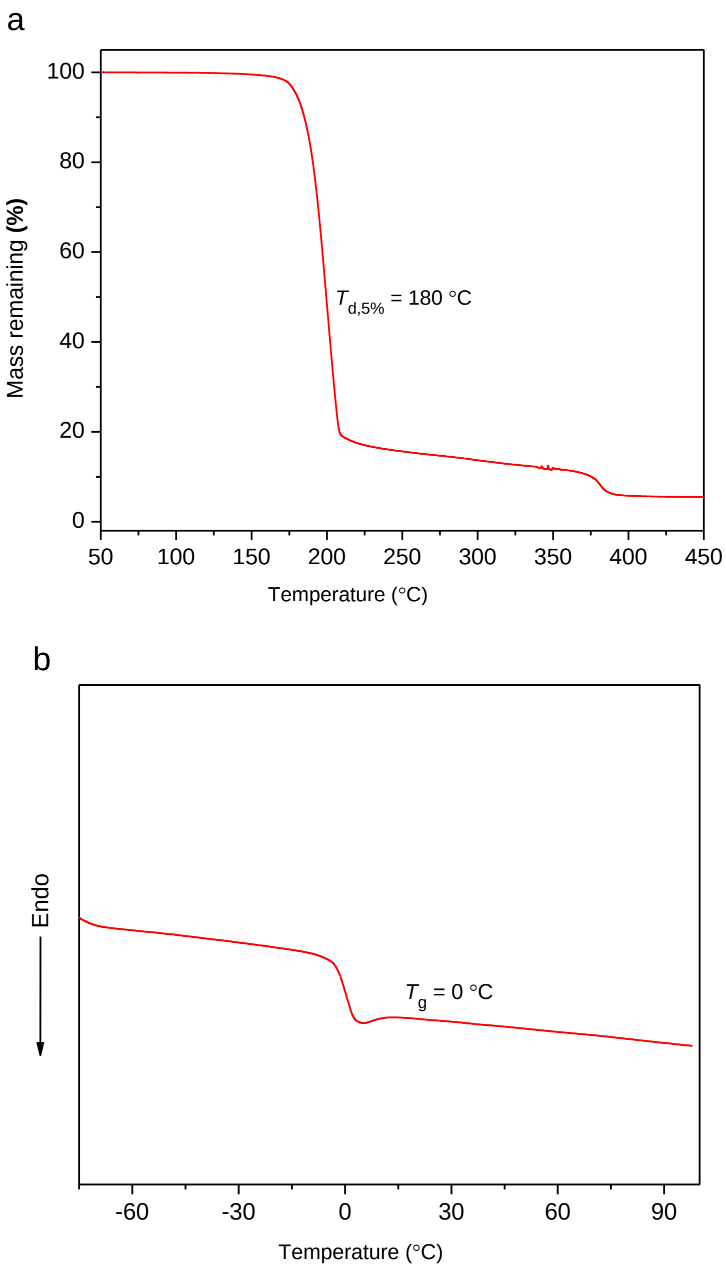

Supplementary Fig. 120 (a) TGA and (b) DSC curves of the obtained copolymer of **P1C**.

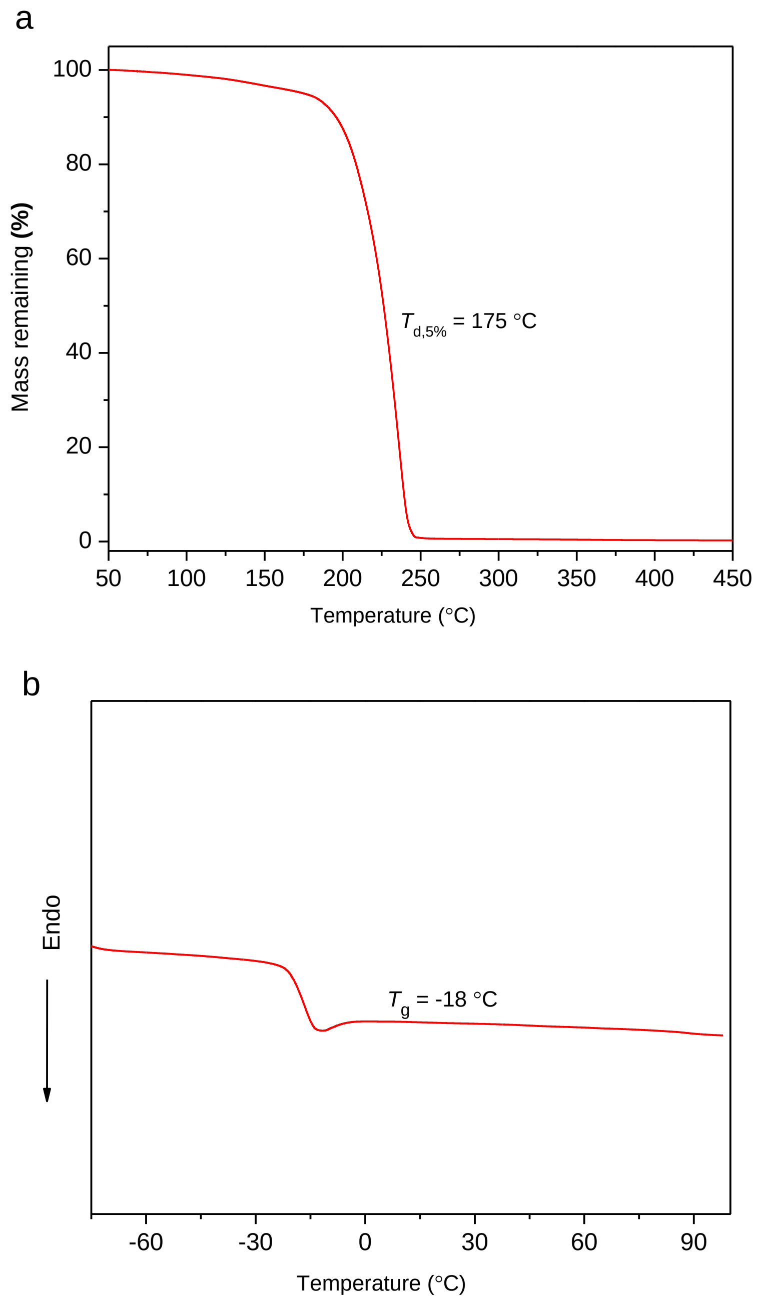

Supplementary Fig. 121 (a) TGA and (b) DSC curves of the obtained copolymer of **P1D**.

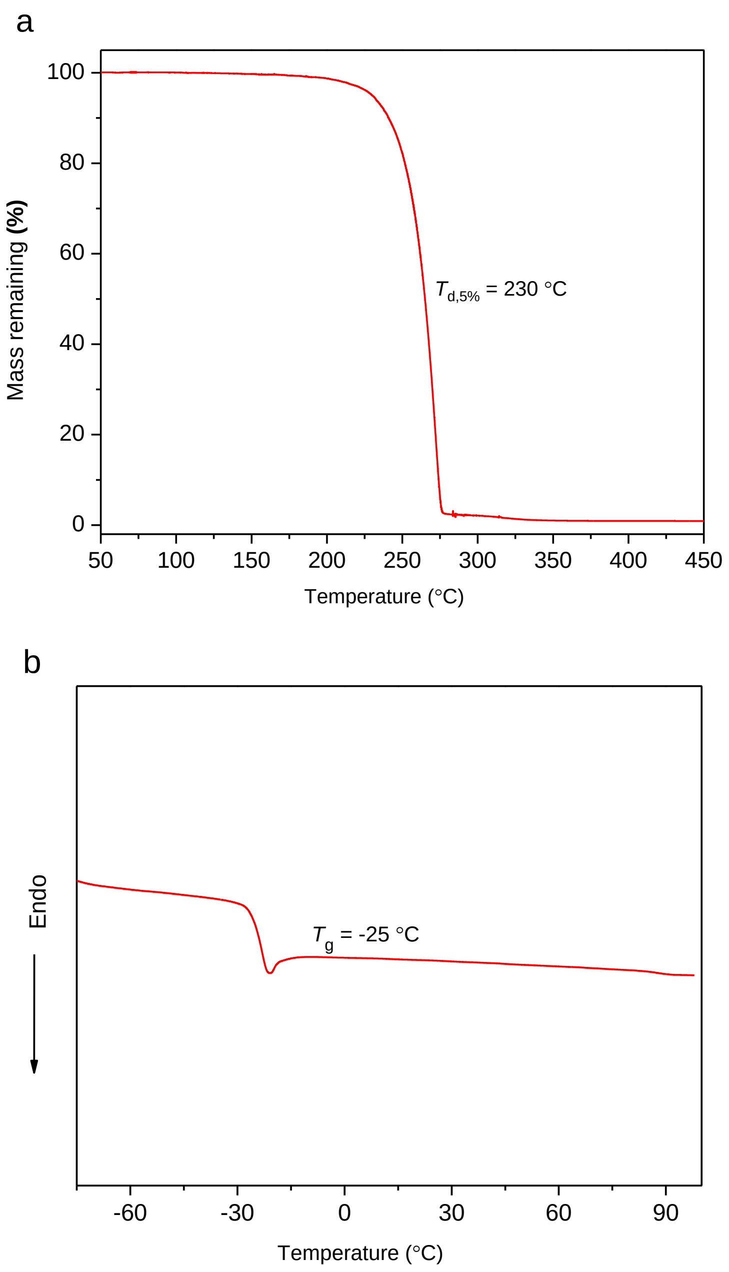

Supplementary Fig. 122 (a) TGA and (b) DSC curves of the obtained copolymer of **P1E**.

Supplementary Table 5. Copolymerization of **36** with **A** with $InBr_3$ [a]

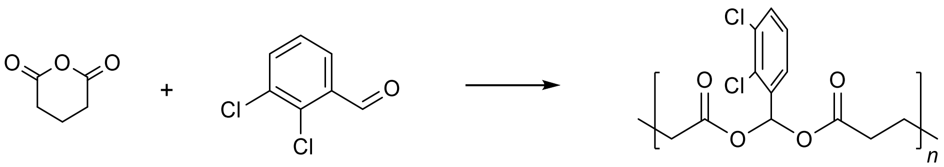

entry	[**36**]:[**A**]:[$InBr_3$]	*T* (°C)	t (h)	Conv.[b]	yield (g)[c]	M_n (kDa)[d]	*Đ*[d]
1	4000:4000:1	-10	5	90	13.9	18.0	1.5

[a] The copolymerization was performed in 7.0 ml CH_2Cl_2, 10 g of **36**, 6.5 g of **A**; [b] Conversion of **A**, determined by ^{1}H NMR spectroscopy; [c] Purified **P36A**, determined by weighing; [d] Determined by GPC in THF, calibrated with polystyrene standards.

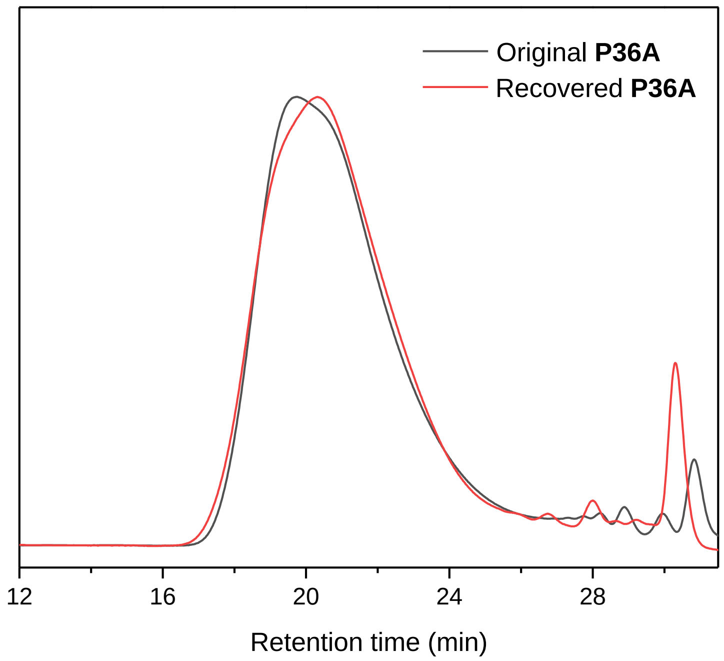

Supplementary Fig. 123 GPC curves of the original and recovered **P36A**.

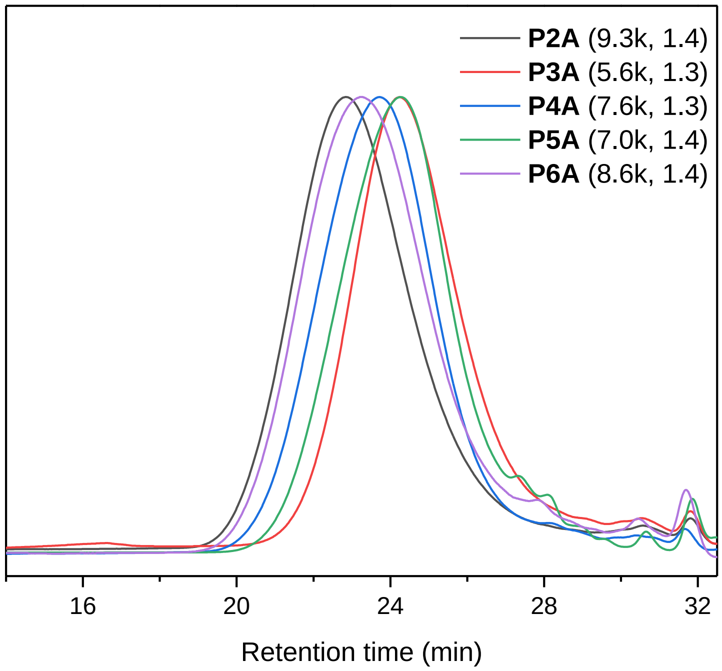

Supplementary Fig. 124 Representative GPC curves of the obtained copolymers of **P2A**, **P3A**, **P4A**, **P5A**, **P6A**.

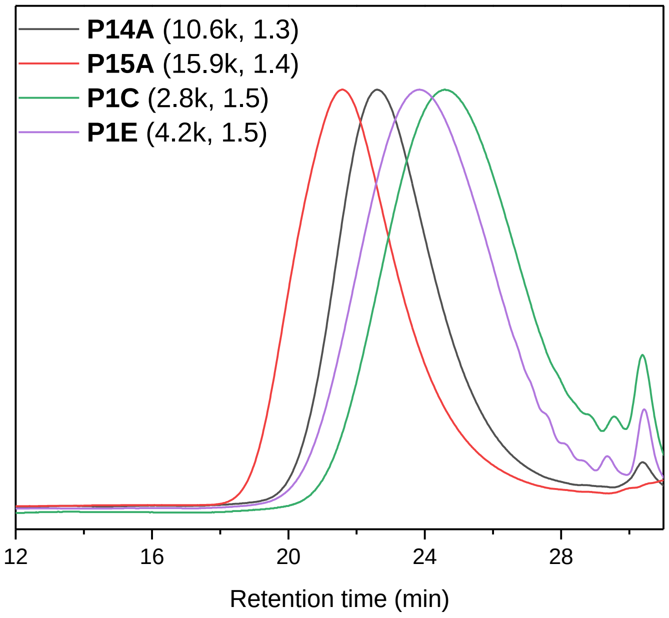

Supplementary Fig. 125 Representative GPC curves of the obtained copolymers of **P14A**, **P15A**, **P1C**, **P1E**.

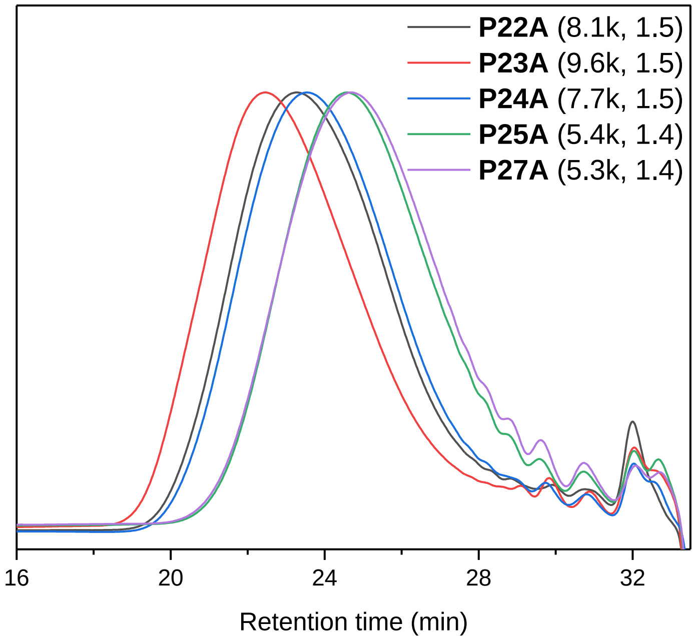

Supplementary Fig. 126 Representative GPC curves of the obtained copolymers of **P22A**, **P23A**, **P24A**, **P25A**, **P27A**.

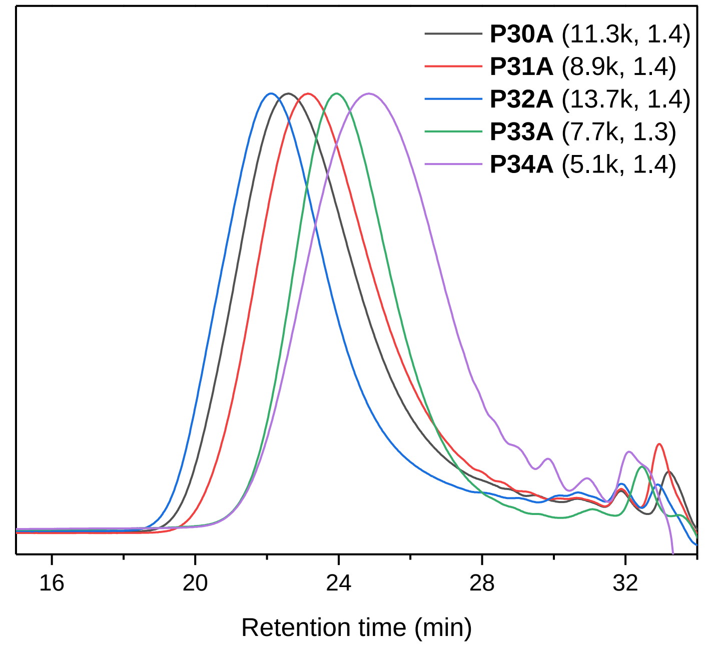

Supplementary Fig. 127 Representative GPC curves of the obtained copolymers of **P30A**, **P31A**, **P32A**, **P33A**, **P34A**.

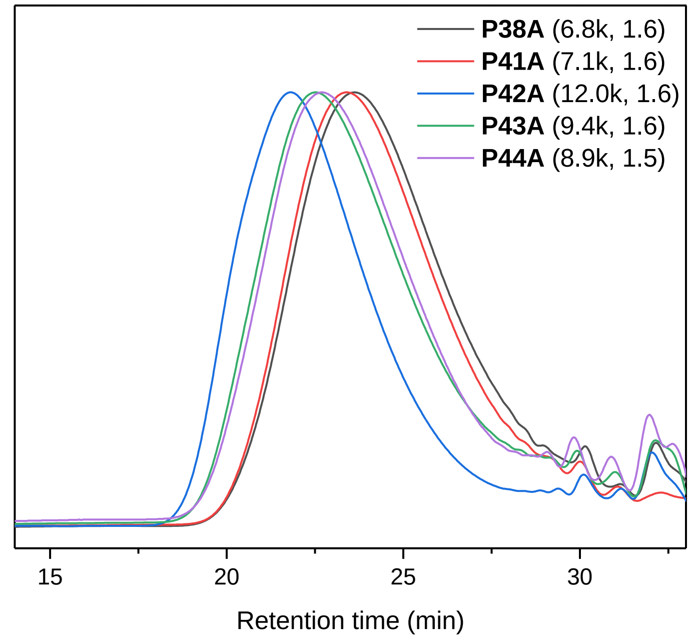

Supplementary Fig. 128 Representative GPC curves of the obtained copolymers of **P38A**, **P41A**, **P42A**, **P43A**, **P44A**.

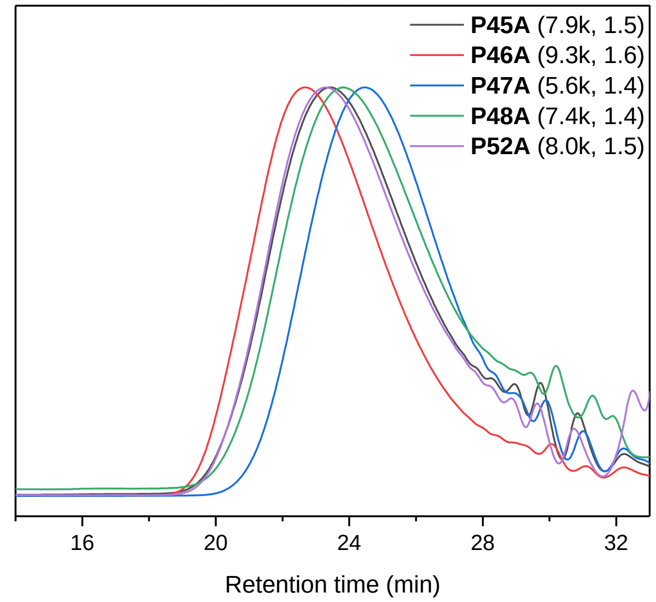

Supplementary Fig. 129 Representative GPC curves of the obtained copolymers of **P45A**, **P46A**, **P47A**, **P48A**, **P52A**.
